# SAGE: Spatially Aware Gene Selection and Dual‐View Embedding Fusion for Domain Identification in Spatial Transcriptomics

**DOI:** 10.1002/advs.202520333

**Published:** 2026-01-04

**Authors:** Yi He, Yunpei Xu, Liqing Ding, Hong‐Dong Li, Yaohang Li, Shaokai Wang

**Affiliations:** ^1^ School of Computer Science and Engineering Central South University Changsha China; ^2^ Xiangjiang Laboratory Changsha China; ^3^ Hunan Provincial Key Lab on Bioinformatics Central South University Changsha China; ^4^ Department of Computer Science Old Dominion University Norfolk VA USA; ^5^ Hong Kong University of Science and Technology Hong Kong SAR China

**Keywords:** clustering, contrastive learning, graph neural networks, spatial transcriptomics

## Abstract

Despite enabling high‐resolution mapping of gene expression within tissues, spatial transcriptomics (ST) still faces challenges in accurately segmenting spatial domains due to complex tissue architecture and limitations of current methods. Most approaches rely on local spatial priors, lack gene‐level interpretability, and fall short in capturing structure‐discriminative genes or long‐range functional relationships, limiting their ability to resolve biologically meaningful architectures. We present Spatially Aware Gene selection and dual‐view Embedding fusion (SAGE), a unified and reproducible framework for domain identification in spatial transcriptomics that combines topic‐driven gene selection with dual‐view embedding fusion to address these gaps. SAGE integrates non‐negative matrix factorization (NMF)‐based topic modeling with classifier‐based importance scoring to identify highly spatially informative genes, and fuses a local expression graph with a topic‐driven non‐local graph via consensus refinement and contrastive graph representation learning to jointly learn spatial and functional embeddings. Evaluated on 34 real‐world datasets, SAGE not only outperforms existing methods in clustering accuracy but also reveals functionally coherent regions and interpretable gene expression patterns. In case studies, SAGE reveals spatial heterogeneity associated with a pre‐malignant activation state in human breast cancer. Moreover, in zebrafish melanoma, it refines the tumor–muscle interface into transcriptionally distinct subdomains and uncovers shared vascular signatures between anatomically separate tissues. Together, these results demonstrate that SAGE can be used not only for accurate spatial domain delineation across diverse ST platforms, but also for dissecting microenvironmental niches and long‐range tissue interactions underlying disease progression.

## Graphical abstract

SAGE is a unified framework for spatial domain identification in spatial transcriptomics that jointly models tissue architecture and gene programs. Topic‐driven gene selection (NMF plus classifier‐based scoring) highlights spatially informative genes, while dual‐view graph embedding fuses local expression and non‐local functional relations. Across 34 datasets, SAGE improves clustering and yields interpretable domains, revealing microenvironmental niches and long‐range tissue interactions in cancer and melanoma.

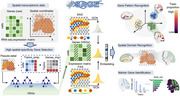

## 1 Introduction

Spatial transcriptomics (ST) technologies have revolutionized our understanding of the relationship between tissue architecture and function by enabling the in situ resolution of gene expression [1]. With continuous advances in imaging, sequencing (e.g., Seq‐Scope [2], Slide‐tags [3], MERFISH [4], Slide‐seq [5]), and tailored computational methods [6, 7], ST data have rapidly increased in both resolution and scale, driving progress in fields such as developmental biology [8], neuroscience [9], and the tumor microenvironment [10].

Spatial domain segmentation aims to identify regions within tissues that exhibit coherent structure and function, representing a central task in ST [11]. However, due to the intrinsic complexity of tissues and the heterogeneity of gene expression, achieving high‐precision delineation remains challenging [12]. Traditional single‐cell clustering methods (e.g., Seurat [13], Louvain [14]) disregard spatial context, which frequently leads to fragmented domain assignments. To address this issue, researchers have developed spatial‐aware clustering methods. Statistical modeling‐based approaches (e.g., HMRF [15], BayesSpace [16], and BASS [17]) incorporate spatial priors to enforce clustering continuity, but their underlying assumptions can be easily violated in highly heterogeneous tissues [18], compromising both generalizability and accuracy [19]. In contrast, deep learning‐enhanced methods [20, 21, 22, 23, 24, 25, 26, 27, 28, 29, 30] leverage the powerful nonlinear modeling capacity of neural networks, demonstrating greater potential in learning complex relationships between gene expression and spatial context.

Despite these advances, three fundamental challenges persist in spatial domain segmentation. First, capturing functionally relevant but spatially distant region relationships remains difficult [20]. In many biological contexts, such as tissue development or disease progression, functionally related cells or tissue regions are not necessarily adjacent. This non‐locality poses a fundamental difficulty for spatial domain segmentation. However, most methods, including representative graph‐based approaches such as STAGATE [22], GraphST [23] and SpaGCN [25], rely on single‐view or integrated graphs constructed primarily from spatial proximity, which effectively encodes local spatial continuity but limits the ability to model long‐range functional associations [21, 22]. As a result, functionally coherent but spatially dispersed regions may be incorrectly partitioned. Furthermore, the over‐smoothing effect introduced by local spatial information aggregation tends to blur spatial heterogeneity, thereby limiting the accuracy of boundary delineation [23, 24].

Second, decoupling gene selection from spatial domain identification hinders the identification of structure‐discriminative genes, including low‐abundance but biologically essential markers that define spatially distinct regions [31, 32]. Traditional gene selection strategies often rely on a single global variability metric, which fails to capture expression patterns tightly linked to spatial architecture. While highly variable genes (HVGs) reflect global expression variation, they ignore spatial context; spatially variable genes (SVGs) consider spatial location but are prone to noise and sampling bias, risking the omission of critical markers. As a result, key structure‐discriminative genes may be missed, compromising both spatial domain delineation and downstream biological interpretation.

Third, most methods [20, 21, 22, 23, 24, 25, 26, 27, 28, 29, 30] such as graph neural networks (GNNs) and variational autoencoders, lack sufficient interpretability in identifying spatial domains within tissues. Due to their inherently ‘black‐box’ nature, these models typically require post hoc analyses (e.g., differential gene expression profiling) to derive biological insights from the latent embeddings. Such indirect strategies obscure the contribution of individual genes to spatial patterning and cellular heterogeneity, limiting the ability to uncover gene regulatory mechanisms or cell–cell interactions in situ. Consequently, the biological relevance and mechanistic insight of such analyses are often diminished, underscoring the pressing need for inherently interpretable models in spatial transcriptomics.

Recent advancements in model design for optimizing interpretability have shown great promise. For example, GASTON [33] introduces interpretable hidden layers (isodepth) combined with gradient‐based analysis to highlight gene‐level contributions to spatial domains, while STAMP [34] incorporates interpretable modules to identify key genes driving spatial heterogeneity. However, these designs neither address the challenge of modeling spatially distant but functionally related regions, nor consider the limitations introduced by conventional gene selection strategies, which often fail to capture the complexity of spatially dispersed gene expression.

To address these challenges, we propose SAGE (Spatially Aware Gene selection and dual‐view Embedding fusion), a unified framework that combines task‐directed gene selection with dual‐view graph representation learning for spatial domain identification. Rather than relying solely on global variability metrics (e.g., HVGs or SVGs), SAGE emphasizes the contribution of structure‐discriminative genes to enhance spatial segmentation accuracy. SAGE introduces an innovative non‐negative matrix factorization (NMF)‐based topic modeling stage coupled with classifier‐driven importance scoring to identify topic‐specific genes (TSGs) that are both interpretable and highly discriminative of spatial domains. To capture complementary spatial and functional relationships, SAGE constructs a dual‐view graph consisting of a local expression *k*‐NN graph and a topic‐driven non‐local graph and introduces a consensus‐frequency filtering mechanism to refine graph topology, enhancing long‐range connectivity and reducing boundary noise. Finally, SAGE introduces a tailored contrastive graph representation learning scheme [35] that performs dual‐view embedding fusion, strengthens cross‐view consistency, and mitigates false positives arising from naive negative sampling. By learning joint spatial–functional representations, SAGE maps each domain to topic‐specific gene programs, directly uncovering the gene‐level signatures and functional drivers of spatial organization.

Through comprehensive benchmarking on 34 real‐world spatial transcriptomics datasets [36], SAGE demonstrates superior performance in both spatial domain identification accuracy and biological interpretability, outperforming existing methods. In various biological contexts such as the human dorsolateral prefrontal cortex (DLPFC), mouse brain, and tumor tissue, SAGE accurately reconstructs spatial tissue architecture. In particular, in human breast cancer dataset, SAGE identifies a spatial transcriptomic signature of a “pre‐malignant activation” state within histologically normal tissue. In zebrafish melanoma, SAGE deconstruct the tumor‐invasive front into two functionally distinct domains, revealing a structured interplay between pro‐invasive and host‐response programs. These findings establish that SAGE is not merely a clustering tool but a discovery engine, capable of revealing new biological principles of spatial organization in development and disease.

## 2 Results

### 2.1 Overview of SAGE Workflow

**FIGURE 1:**
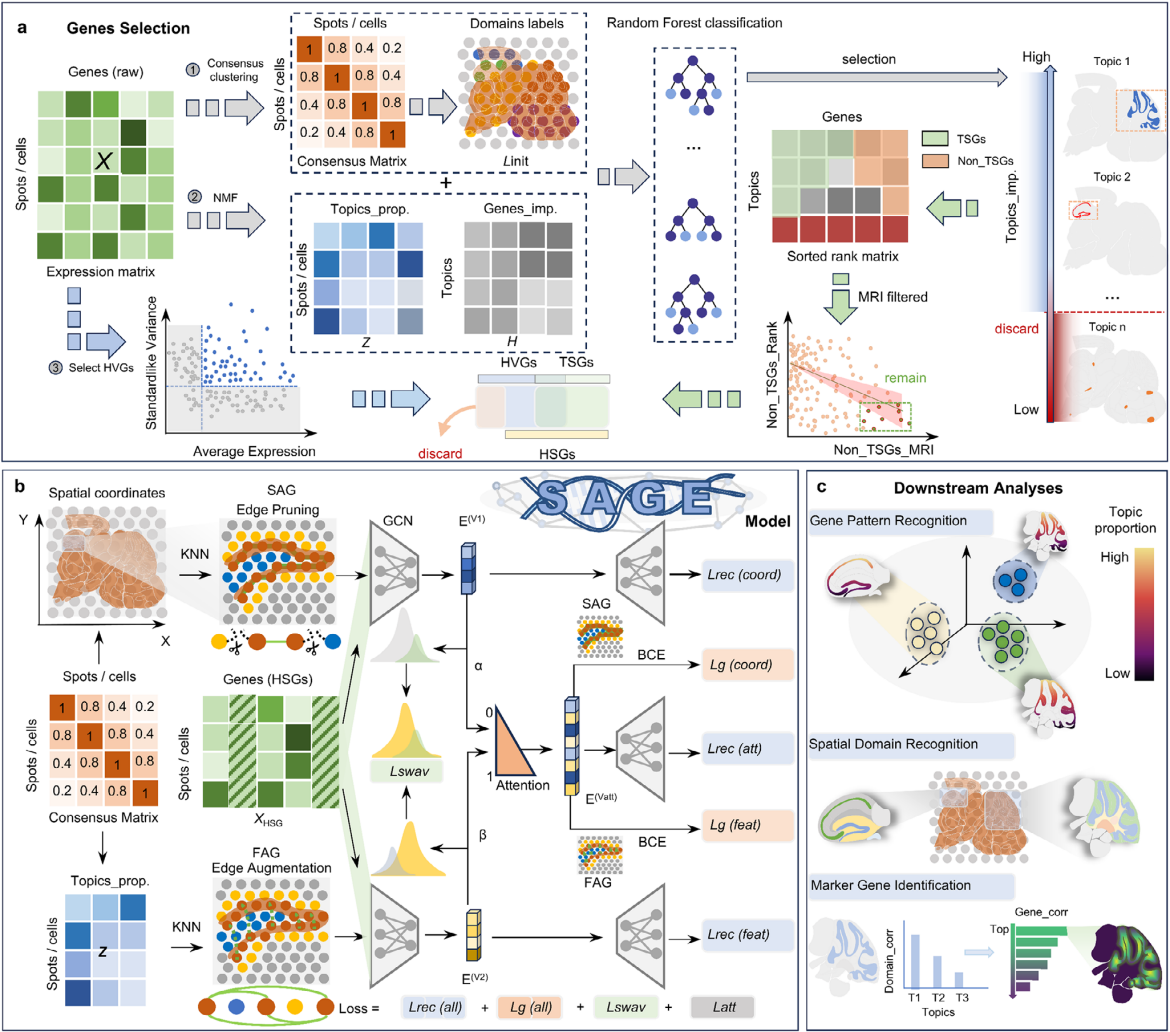
Overview of the SAGE framework. (a) SAGE takes spatial transcriptomics (ST) data as input, integrating gene expression profiles with spatial coordinates. It performs consensus clustering to generate initial pseudo‐labels *L_init_* and applies non‐negative matrix factorization (NMF) to extract topic distributions *Z* and topic‐specific gene weights *H*. Spatially informative topics are then identified using a random forest classifier, and the corresponding topic‐specific genes (TSGs) are selected. These are combined with highly variable genes (HVGs) to form the final set of high spatial‐specificity genes (HSGs). (b) Using spatial coordinates, the consensus matrix (encoding spot–spot similarity across clustering iterations), and the expression matrix *X_HSG_*, SAGE constructs two complementary graphs: a spatial‐based adjacency graph (SAG) and a feature‐based adjacency graph (FAG). These graphs are used to learn dual‐view embeddings, which are integrated via a self‐attention mechanism to generate a biologically meaningful low‐dimensional representation. (c) The final embedding supports various downstream analyses, including spatial domain recognition, gene pattern recognition, and marker gene identification.

As illustrated in Figure 1, SAGE comprises two tightly integrated components: a gene selection module for identifying spatially informative features, and a dual‐view graph neural network (GNN) for spatial representation learning. By integrating spatial statistics with topic modeling, SAGE captures both local spatial continuity and long‐range transcriptomic similarity, enabling accurate domain segmentation and functional gene discovery.

In the gene selection stage, SAGE adopts a hybrid filtering strategy that combines globally variable genes (HVGs) with topic‐specific genes (TSGs) derived from NMF. NMF decomposes the gene expression matrix *X* into a spot‐topic matrix *Z* and a topic‐gene matrix *H*, representing spatial topic distributions and gene contributions, respectively. SAGE first performs multi‐resolution clustering on the gene expression matrix *X* to generate preliminary spatial labels *L*_init_. Then, it evaluates each topic's spatial relevance by training a random forest classifier on these preliminary labels and using its feature‐importance scores to rank topics. Topics with high importance scores are considered spatially informative, and their top‐ranked genes in the topic‐gene matrix *H* are selected as TSGs. In parallel, HVGs are identified through mean–variance analysis and subsequently refined by dual filtering based on their topic‐ranking positions and spatial autocorrelation metrics (e.g., Moran's *I*), thereby excluding genes that are non‐topic‐specific or noisy. The final set of high spatial‐specificity genes (HSGs) is constructed by merging high‐confidence HVGs with TSGs (Figure 1a).

To integrate spatial and functional relationships, SAGE builds two complementary graphs: a spatial‐based adjacency graph (SAG) based on *k*‐nearest neighbors in physical space, and a feature‐based adjacency graph (FAG) based on cosine similarity between spots’ topic distributions (Figure 1b). Both graphs are further refined through a consensus‐based optimization to eliminate unreliable edges and enhance consistent ones, ensuring robust graph structure. The spatial and feature graphs, together with the HSG‐derived feature matrix *X_HSG_* extracted from the original topic matrix *X*, are fed into a dual‐branch graph neural network (DGNN). Each branch processes one graph view using dedicated GCN layers, yielding two embeddings $E^{\left(v_{1}\right)}$ and $E^{\left(v_{2}\right)}$, which are then fused via trainable attention weights (α ∈ [0,1]) to produce the final representation $E^{\left(v_{att}\right)}$. A joint objective combines reconstruction losses for both graphs with a SwAV [35] (Swapping Assignments between multiple Views) ‐based contrastive loss to enhance clustering consistency across views and improve representation discriminability. SAGE supports multiple downstream analyses, including spatial domain segmentation, gene co‐expression pattern detection, and marker gene identification (Figure 1c). The complete SAGE pipeline is illustrated in Figure 1, with detailed methodological descriptions provided in the “Methods” section.

### 2.2 Benchmarking Domain Segmentation Performance

**FIGURE 2:**
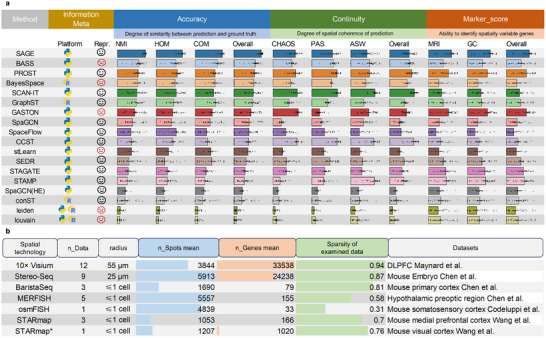
Benchmarking SAGE against 16 representative methods across 34 ST datasets. (a) Overview of the 16 evaluated methods, including their implementation platforms (Python or R) and whether they support context‐aware representations (“Repr.”: A smiling face for supported, a neutral face for not). Evaluation includes eight metrics across three categories: accuracy (NMI, HOM, COM), continuity (CHAOS, PAS, ASW), and marker identification (Moran's I, Geary's C). Each bar represents the median rank‐based score across 34 datasets; dots indicate individual dataset scores. Methods are ordered by descending *Overall_accuracy_*, computed as the average rank score of NMI, HOM, and COM. Details of the rank‐based scores calculations are provided in the “Methods” section. (b) Summary of datasets used in the benchmark, including the number of subsets, spatial resolution, average spots/cells count, average gene count, and matrix sparsity.

To comprehensively evaluate the performance of SAGE, we benchmarked it against 16 representative methods, building on benchmarking results previously reported by Yuan et al. [36]. and further including 3 recently proposed approaches, such as GASTON [33], STAMP [34] and PROST [37], These competing methods span traditional clustering algorithms (e.g., Leiden, Louvain), statistical modeling‐based methods (e.g., BayesSpace, BASS), and deep learning‐enhanced methods (e.g., GraphST, STAGATE, PROST, GASTON), as well as a topic model–based method (STAMP). All methods aim to identify biologically meaningful spatial domains across 34 real‐world ST datasets encompassing seven major ST technologies (Figure 2b), with dataset details provided in Tables S1 and S2.

This study conducted a comprehensive evaluation using eight metrics across three categories: accuracy (NMI, HOM, COM), continuity (CHAOS, PAS, ASW), and marker score (Moran's *I*, Geary's C). Instead of using raw metric values, we adopted a rank‐based scoring scheme, in which each method's performance was ranked across all methods for each metric, and the ranks were used to compute aggregated scores—higher scores indicate better relative performance. This approach is consistent with previous benchmark analyses [36].

For a given metric *i*, we define *rank_i_* as the rank‐based score of a method on a single dataset. Then, $\bar{rank_{i}}$ denotes the average of *rank_i_* across all datasets (e.g., *rank_NMI_*, $\bar{rank_{\mathrm{NMI}\;}}$). The final ranking was determined by averaging the rank‐based scores across the three accuracy‐related metrics (NMI, HOM, and COM). We refer to this average score as *Overall_accuracy_*. Similarly, we compute *Overall_continuity_* by averaging the rank‐based scores of CHAOS, PAS, and ASW, and *Overall_marker_* by averaging those of Moran's *I* and Geary's C. Details of the rank‐based score calculations are provided in the “Methods” section. Figures S1–S14 provide a comprehensive overview of the performance across all datasets based on eight evaluation metrics, as well as the spatial domain identification results produced by SAGE for each dataset.

The overall evaluation demonstrates that SAGE achieves more accurate segmentation compared to other methods (ranked 1 in *Overall_accuracy_*: 0.94), outperforming the second‐best (BASS: 0.72) and third‐best (PROST: 0.69) by 30.56% and 36.23%, respectively (Figure 2a; Tables S3–S9). Among the 34 datasets, SAGE ranks first in *Overall_accuracy_* in 24 datasets. For instance, On the DLPFC dataset [38] (10x Visium, Figures S1 and S2), SAGE accurately recapitulates the expected cortical lamination architecture and achieves the highest performance in accuracy metrics ($\bar{rank_{NMI}}$= 0.97, $\bar{rank_{HOM}}$= 0.91, $\bar{rank_{COM}}$= 0.95). Compared to the second‐best method (GraphST: $\bar{rank_{NMI}}$= 0.73, $\bar{rank_{HOM}}$= 0.67, $\bar{rank_{COM}}$= 0.66), SAGE demonstrates improvements of 33.09%, 35.00%, and 23.16%, respectively. In terms of marker gene detection, SAGE identifies spatially informative marker genes with stronger spatial specificity (ranked 1 in *Overall_marker_*: 0.83), outperforming the second‐best (PROST: 0.69) and third‐best (STAGATE: 0.62) by 20.29% and 33.87%, respectively. Specifically, SAGE ranks within the top three in *Overall_marker_* in 26 datasets. In continuity evaluation, SAGE ranks fifth (*Overall_continuity_*: 0.69), and places within the top 5 in 24 out of 34 datasets, indicating stable spatial coherence despite its emphasis on boundary.

During the evaluation, we observe that certain methods exhibit biases in performances across datasets generated by different ST technologies and lack generalizability. Although GraphST performs well on the DLPFC dataset (10x Visium, ranked 2 in *rank*_NMI_), its performance drops significantly on the imaging‐based mouse hypothalamus dataset (MERFISH, ranked 6 in *rank*_NMI_). On the mouse embryo dataset (Stereo‐Seq), SCAN‐IT ranks first in continuity assessment (ranked 1 in *rank*_ASW_) but performs poorly in accuracy (ranked 7 in *rank*_NMI_). This may be attributed to the heavy reliance of SCAN‐IT on spatial information, which makes it difficult to accurately model the heterogeneous tissue structures of the mouse embryo (Figures S3 and S4). Methods like CCST and SpaGCN show similar inconsistencies, performing well on certain datasets but dropping steeply on others.

By contrast, SAGE maintains a more consistent balance between accuracy, continuity, and marker‐based performance across all datasets. It consistently achieves high accuracy, particularly excelling in topologically complex tissues such as the mouse embryo (ranked 1 in *rank*_NMI_). Consistent with this behavior, SAGE attains the highest Total score (2.45) among all 17 methods, where the Total score is defined as the sum of the three category‐wise mean rank‐based scores (Total  = Overall_accuracy_  + Overall_continuity_ + Overall_marker_, Table S22). Although SAGE does not rank first in Overall_continuity_, it remains within the top five while clearly leading in accuracy and marker identification, reflecting the intended trade‐off between preserving sharp, biologically meaningful boundaries and maintaining spatial coherence. To quantify performance stability, we calculate the standard deviation of ranking scores for each method across different ST platforms (Figure S15; Table S23). Lower standard deviations indicate more consistent performance across platforms. SAGE demonstrates the highest robustness, with low variance across accuracy‐related metrics (σ (*NMI*)= 0.04, σ (*HOM*)= 0.10, σ (*COM*)= 0.08; where σ denotes the standard deviation). In comparison, methods such as BASS (σ (*NMI*)= 0.18, σ (*HOM*)= 0.17, σ (*COM*)= 0.18) and PROST (σ (*NMI*)= 0.15, σ (*HOM*)= 0.15, σ (*COM*)= 0.15) show much higher standard deviations, indicating inconsistent cross‐platform performance. For instance, BASS performs well on the mouse primary cortex dataset (BaristaSeq) but poorly on the mouse embryo dataset (Stereo‐seq), conversely, while PROST achieves better performance on the mouse somatosensory cortex dataset (osmFISH) but ranks low on hypothalamic preoptic region dataset (MERFISH, Figure S7). Overall, SAGE exhibits both high accuracy and strong generalizability across diverse spatial transcriptomics platforms, highlighting its suitability for broad biological applications.

### 2.3 SAGE Effectively Resolves Cortical Layer Organization in DLPFC With High Accuracy

To further investigate the performance of SAGE in spatial domain segmentation, we use the DLPFC dataset as a representative example for detailed visualization and comparative analysis. The dataset comprises 12 tissue sections from three donors and is widely recognized as the gold standard for evaluating spatial clustering methods. We use the manual annotations provided by Maynard et al. [38]. as ground truth, which are based on tissue morphology and marker gene expression.

**FIGURE 3:**
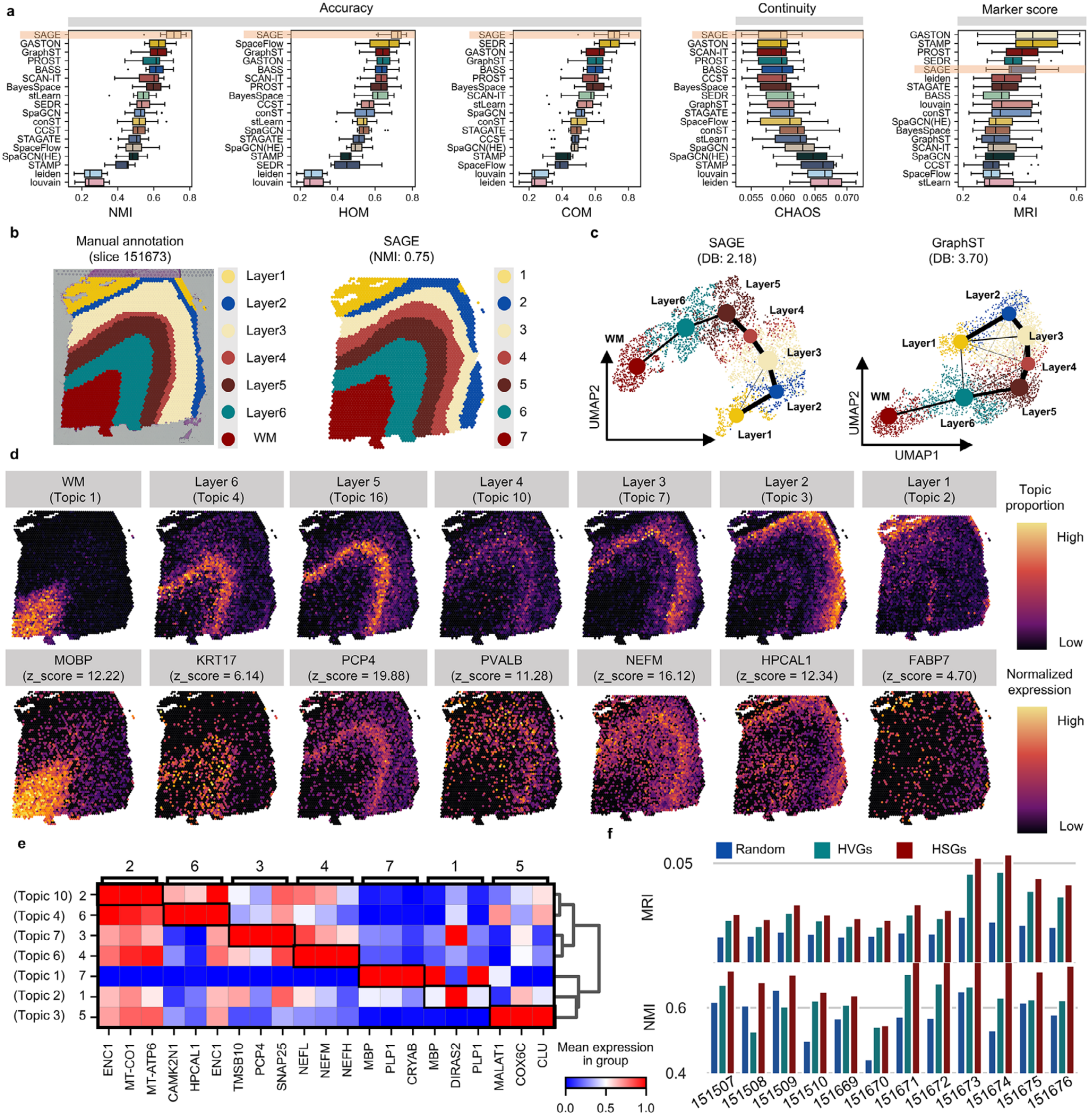
Enhanced identification of spatial domains and HSGs in DLPFC using SAGE. (a) The boxplot summarizes the benchmark performance of SAGE (highlighted with a yellow background) compared to 16 representative methods, including metrics for accuracy (NMI, HOM, COM), continuity (CHAO), and marker score (MRI). The boxplot's center line, box limits, and whiskers denote the median, upper and lower quartiles, and 1.5× interquartile range, respectively. (b) Comparison of manual annotations (left, provided by Maynard et al.) and SAGE‐inferred spatial domains (right) for section 151673. (c) Comparison of UMAP visualizations and trajectory inference by SAGE (left, DB = 2.18) and GraphST (right, DB = 3.7) for section 151673. (d) Spatial gradient of seven expression topics from WM to L1, with top marker genes and their significance (z‐scores) shown below. (e) The gene expression heatmap identified by SAGE highlights distinct spatial localization features. (e.g., MBP in WM). (f) Bar plots comparing three gene selection strategies (random, HVGs and HSGs), where SAGE‐derived HSGs significantly improve downstream performance (*p* < 0.01, two‐sided *t*‐test).

As shown in Figure 3a and Figures S1 and S2, SAGE achieves the highest accuracy performance across the 12 tissue slices (median NMI = 0.71, median HOM = 0.72, median COM = 0.71), outperforming the second‐best methods GraphST (median NMI = 0.62), SpaceFlow (median HOM = 0.67), and SEDR (median COM = 0.69) by 14.75%, 7.46%, and 2.9%, respectively (Figure 3a; Table S24). Additionally, SAGE achieves top performance in continuity and marker gene detection metrics (ranked 1 in median CHAOS, ranked 3 in median MRI), further demonstrating its broad applicability in resolving the structured laminar organization of the cerebral cortex across multiple tissue slices.

To further compare the spatial domain interpretation capabilities of different methods, we select the top 5 methods supporting embedded visualization based on NMI ranking (GraphST, PROST, SCAN‐IT, stLearn, SEDR). The results reveal (Figures S16–S27) that the spatial domains identified by SAGE are highly consistent with both the manual annotations and the established anatomical definitions of cortical layers in neuroscience. We use the representative tissue slice #151673 as an example. Figure 3b compares manual annotation with the results produced by SAGE and results from other methods are provided in Figure S24. In terms of cortical layer boundary segmentation, SEDR (NMI = 0.57) and PROST (NMI = 0.46) identify only partial cortical layers and white matter (WM), without distinguishing clear boundaries between layers L1–L6. stLearn (NMI = 0.40) detects only limited boundaries, such as between L1–L2 and WM–L6, while other domain boundaries remain poorly resolved. SCAN‐IT (NMI = 0.68) identifies relatively smooth cortical layer boundaries but shows notable discrepancies compared with manual annotations. GraphST (NMI = 0.73) erroneously merges L3 and L4. In contrast, SAGE (NMI = 0.75) not only achieves precise delineation of cortical layers L1–L6 and WM but also accurately reconstructs the boundary structures between these layers. Its results are highly consistent with manual annotations and demonstrate superior performance in preserving boundary continuity and spatial proportions compared to other methods [39].

We further evaluate the quality and interpretability of the low‐dimensional embeddings using Uniform Manifold Approximation and Projection (UMAP) and Partition‐based Graph Abstraction (PAGA) trajectory inference (Figure 3c; Figure S24d,e). To assess cluster separability, we calculate the Davies‐Bouldin (DB) index on the UMAP embeddings, where lower values indicate better‐defined and more distinct cortical layers. SAGE (DB = 2.18) demonstrates superior inter‐layer separation compared to GraphST (DB = 3.7), which can be attributed to its improved modeling of topological structures. We then assess biological interpretability by projecting PAGA‐inferred pseudotime onto the original spatial coordinates. The pseudotime ordering derived from SAGE closely aligns with known cortical anatomy, progressing from the inner L6 layer near the white matter (WM) to the outer L1 layer adjacent to the pia mater. This spatial trajectory reflects the classical “inside‐out” pattern of cortical development, highlighting SAGE's capacity to capture biologically meaningful spatiotemporal organization.

To assess the biological interpretability of the spatial domains identified by SAGE, we analyze the gene co‐expression topics uncovered by the model. SAGE identifies seven distinct topics that exhibit strong correspondence with the known laminar structure of the DLPFC, as confirmed by manual annotations from the original study (Figure 3d). To further validate the biological relevance of these topics, we examine the spatial expression of well‐established cortical layer marker genes. Here, the z‐score quantifies the importance of each gene within its associated topic (Methods). Notably, genes such as *MOBP* (z‐score = 12.22), *PCP4* (z‐score = 19.88), *PVALB* (z‐score = 11.28), *NEFM* (z‐score = 11.31), and *HPCAL1* (z‐score = 12.34) display distinct laminar‐specific expression patterns. These marker–topic associations are consistent with well‐established biological roles of these genes in cortical lamination: *MOBP* [40] marks myelin structure and white matter (WM); *PCP4* [41] is enriched in deep‐layer (L5–L6) corticothalamic neurons; *PVALB* [42] is a canonical marker of fast‐spiking inhibitory interneurons; *NEFM* [43] labels mature cortical projection neurons with prominent expression in deep layers in our data; and *HPCAL1* [44] is enriched in superficial (L2/3) and L6b excitatory neurons (Data S1).

In addition, functional enrichment analysis of the slice shows that different spatial domains exhibit distinct enrichment patterns in KEGG and GO pathways, with domain 5 being highly enriched in protein synthesis, energy metabolism, neurodegenerative disease pathways, and synaptic signaling, supporting its correspondence to deep‐layer neurons (L5–L6) in the DLPFC (Figures S28 and S29; Datas S2–S4). Most of these genes rank among the top genes within their respective topics (marker list in Table S25), with 6 genes ranking in the top 10, underscoring the biological validity of the learned topics. In addition, the heatmap in Figure 3e highlights the accuracy of SAGE in resolving anatomically meaningful cortical domains.

Finally, we perform an ablation study to compare clustering performance using HSGs, HVGs, or random genes as input. The evaluation is carried out on 12 tissue slices from the DLPFC dataset, using NMI and MRI as the evaluation metrics. HSGs achieve the best clustering results (mean NMI = 0.70, mean MRI = 0.03), outperforming HVGs (mean NMI = 0.62, mean MRI = 0.02) by 12.9% and 18.5%, respectively (Figure 3f; Table S26), and cross‐slice analysis (Figures S30–S32; Table S27; Data S5) shows that SAGE‐selected TSGs maintain consistent spatial patterns across adjacent slices. These results highlight both the superior structural consistency and spatial resolution provided by HSGs, reinforcing their biological relevance and functional significance within the SAGE framework.

### 2.4 SAGE Precisely Identifies the Hierarchical Structure and Functional Regions of the Mouse Brain

**FIGURE 4:**
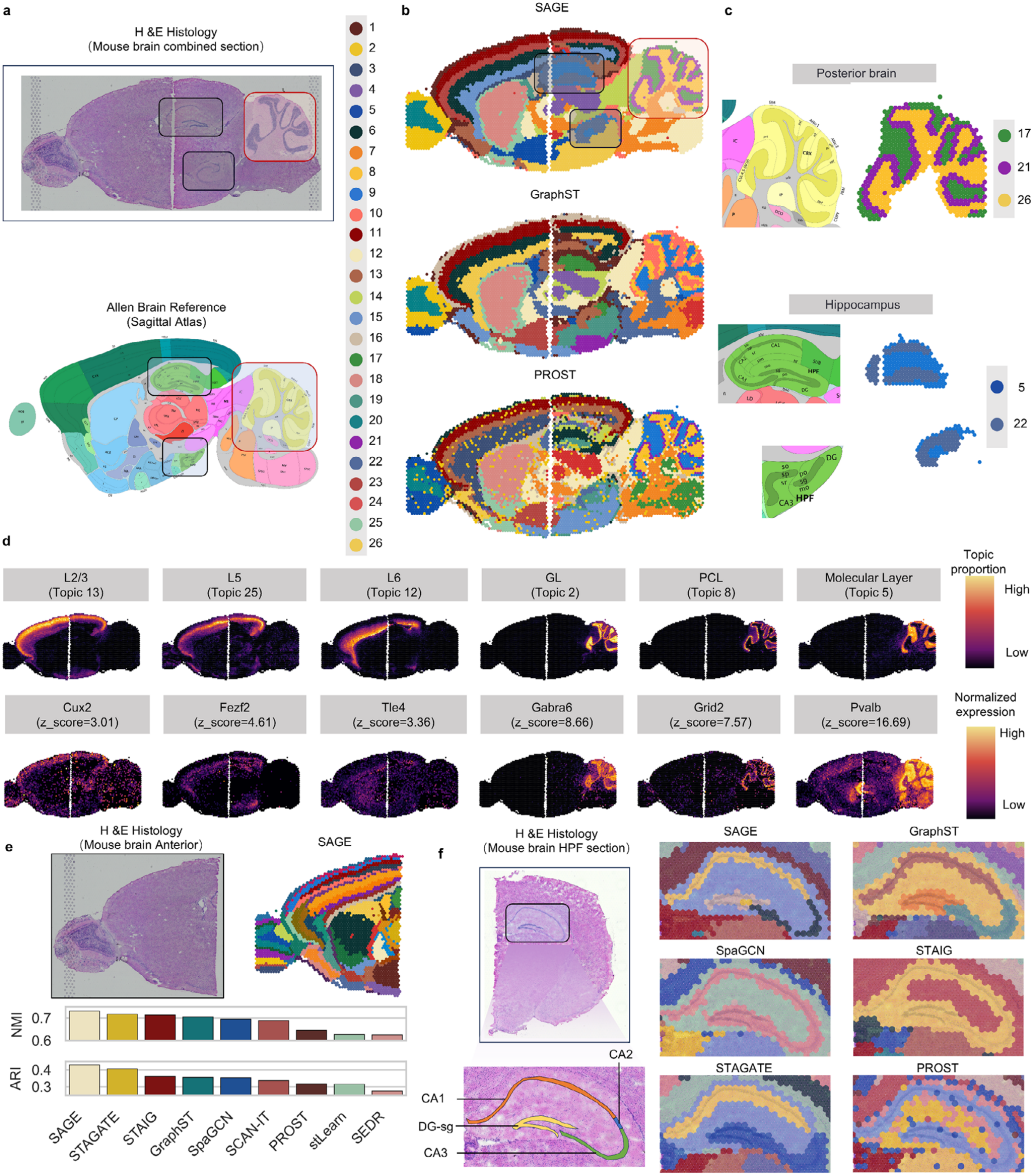
Accurate identification of the mouse brain cortical and hippocampal spatial structures using SAGE. (a) H&E‐stained images of the mouse forebrain and hindbrain. The black box marks the hippocampal region (CA and DG), and the red box marks the cerebellar cortical layers. Anatomical annotations from the Allen Brain Atlas are shown below. (b) Spatial structure identification by SAGE, GraphST, and PROST on the corresponding brain sections (right), with the structural category legend (left). (c) SAGE accurately identifies the three‐layer cortical structure of the cerebellum (domains 17, 21, and 26), and the dorsal and ventral hippocampal regions (domains 5 and 22). (d) Six representative spatial expression patterns identified by SAGE (top), including three from cortical regions (CTX) and three from cerebellar regions (CBX), along with top‐weight marker genes and their z‐scores for each topic (bottom). (e) Sagittal section of the mouse forebrain, showing H&E‐stained images (upper left), spatial structure identification by SAGE (upper right), and comparison with eight other methods using ARI and NMI metrics (bottom). (f) Coronal section of the mouse hippocampus stained with H&E, annotated with CA1–CA3, and DG‐sg regions (left). The clustering results of SAGE and five comparison methods in the hippocampal region (right).

The mouse brain presents a more complex and fine‐grained anatomical structure. To assess the generalization capability and robustness of SAGE in capturing such intricate tissue structures [45], we validate its output against the Allen Mouse Brain Atlas [46] (Figure 4a). The experiment includes three tissue slices: an anterior brain sagittal slice, a posterior brain sagittal slice, and a coronal slice. First, we integrate the anterior and posterior sagittal slices to assess the continuity of gene expression and structural consistency, particularly at slice junctions. We then compare SAGE with eight methods (PROST, GraphST, SEDR, stLearn, SpaGCN, SCAN‐IT, STAGATE, and STAIG), using a fixed number of clusters (*n* = 26) for all approaches, based on prior knowledge of the tissue structure and to match the settings used in the SpaGCN and GraphST [23, 25].

The results (Figure 4b,c) show that SAGE accurately identifies the hierarchical structure of the cerebellar cortex (CBX), including the granular layer, Purkinje cell layer, and molecular layer, which closely aligns with the Allen atlas [46]. Notably, SAGE leverages its long‐range enhancement capability to accurately resolve the dorsal and ventral hippocampal formations. It assigns CA1–CA3 to domain 22 and DG to domain 5, maintaining anatomical continuity despite their discontinuity in 2D sections [47]. In contrast, methods such as SEDR, stLearn, SpaGCN, and PROST suffer from cluster mixing near the CA–DG interface, failing to resolve blurred anatomical boundaries. While SCAN‐IT, STAGATE, and STAIG produce smoother domains, their results deviate from the actual tissue architecture (Figure 4b; Figure S33). GraphST achieves reasonable alignment at the junction of anterior and posterior slices; however, it fails to accurately delineate the layered architecture of the CBX.

Additionally, SAGE further supports and interprets the domain segmentation results via gene co‐expression topics (Figure 4d; Figure S34). It generates a clear embedding structure that effectively identifies the layered architecture of the cerebral cortex (CTX), hippocampus regions (CA and DG), and the three‐layer structure of CBX, each aligned with specific topics. By analyzing gene contributions to each topic (Data S6), SAGE detects multiple established marker genes. For instance, in the cerebellum, it identifies *Gabra6* (GCL, z‐score = 8.66), which encodes a GABA_A receptor subunit specifically expressed in granule cells; *Grid2* (PCL, z‐score = 7.57), a glutamate receptor subtype widely used to label Purkinje cells; and *Pvalb* (ML, z‐score = 16.69), which encodes a calcium‐binding protein predominantly expressed in inhibitory interneurons of the molecular layer.

In the CTX, SAGE also identifies layer‐specific genes, including *Cux2* (L2/3, z‐score = 3.01), a known marker of superficial excitatory neurons; *Fezf2* (L5, z‐score = 4.61), a transcription factor critical for pyramidal neuron development and predominantly expressed in layer 5; and *Tle4* (L6, z‐score = 8.66), a well‐established marker of deep‐layer neurons in layer 6. Notably, SAGE identifies two atypical specific expression genes for the Purkinje layer: *Car8* (z‐score = 16.8) and *Fam107b* (z‐score = 7.59). Although *Car8* lacks enzymatic activity, it is highly expressed in Purkinje cells and functions as a calcium signaling modulator, suggesting a potential role in synaptic regulation [48]. *Fam107b* shows restricted spatial enrichment in the Purkinje layer, and its expression pattern reflects underlying molecular heterogeneity within this neuronal population [49]. These results demonstrate SAGE's ability to resolve both anatomical boundaries and molecular heterogeneity. (Figure S34d; Data S6)

Finally, as a complementary analysis, we perform spatial domain analysis separately on the mouse forebrain and hindbrain slices, aiming to resolve finer anatomical structures by increasing the number of clusters. For the forebrain slice, we use the 52 manually annotated labels provided by Long et al. [23]. as reference (Figure 4e; Figure S35c), and evaluate performance using ARI and NMI. SAGE achieves the highest scores on both metrics (ARI = 0.429, NMI = 0.731), accurately delineating layered cortical structures in both the forebrain and the hindbrain (Figure S35d,c). It also faithfully identifies key regions such as the main olfactory bulb (MOB), caudoputamen (CP), and nucleus accumbens (ACB) [50]. Compared with the Allen Brain Atlas, the spatial domains inferred by SAGE show greater anatomical consistency.

We further evaluate the capacity of each method to resolve fine‐grained hippocampal architecture in the coronal slice, using a fixed number of clusters (*n* = 15), guided by anatomical atlas‐based annotations (Figure 4f). This choice ensures consistency with prior anatomical knowledge while allowing fair comparison across methods. SEDR fails to recover a coherent hippocampal structure, and SCAN‐IT only outlines coarse boundaries. SpaGCN, PROST, and STAIG all merge the CA and DG into a single domain. STAGATE identifies CA1 but merges CA2, CA3, and DG. Both SAGE and GraphST distinguish CA1, CA2&CA3, and DG; however, SAGE achieves finer‐grained delineation, with thinner, more precise domain boundaries that closely match histological structures, whereas GraphST produces coarser boundaries (Figure 4f). These results further highlight SAGE's superiority in resolving complex brain architectures.

### 2.5 SAGE Reveals the Spatial Heterogeneity Associated with Early Oncogenesis in Breast Cancer Tissue

**FIGURE 5:**
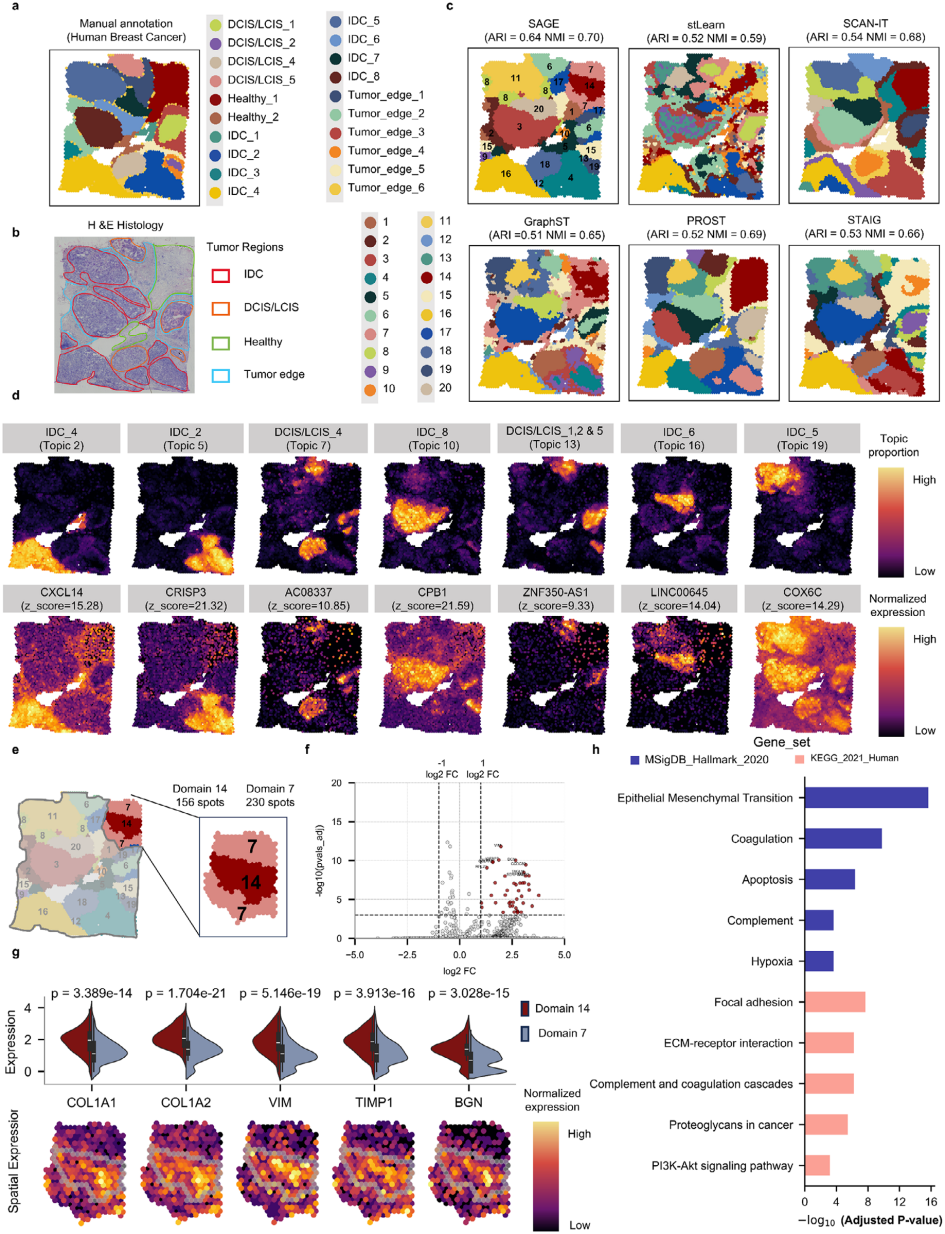
Uncovering intra‐tumoral heterogeneity in human breast cancer tissue using SAGE. (a) 10x Visium ST data of breast cancer sample with pathologist annotations, including IDC, DCIS/LCIS, tumor margins, and healthy tissue. (b) H&E‐stained image with manually labeled regions. (c) Clustering results from SAGE, stLearn, SCAN‐IT, GraphST, and PROST, evaluated by ARI and NMI. Additional comparisons are provided in Figure S37. (d) Seven spatial gene expression topics identified by SAGE (top), along with representative marker genes and their corresponding z‐scores (bottom). Additional patterns are shown in Figure S38. (e) SAGE further resolves the annotated Healthy_1 region into two spatial subclusters, corresponding to domain 14 (156 spots) and domain 7 (230 spots). (f) Volcano plot showing differential gene expression (DGE) analysis between domain 14 and domain 7. Each dot represents a gene, with the x‐axis indicating log_2_ fold change and the y‐axis representing –log_10_ *p*vals_adj. (|log2FC| > 1 and *FDR* < 0.001, Wilcoxon test). (g) Violin plots and spatial expression of selected DEGs (*COL1A1*, *COL1A2*, *VIM*, *TIMP1*, *BGN*). (h) Functional enrichment analysis of DEGs using MSigDB_Hallmark_2020 (blue) and KEGG_2021_Human (pink). The y‐axis denotes enriched pathways, and the x‐axis indicates –log_10_ *p*‐values (Benjamini–Hochberg).

The progression of ductal carcinoma in situ (DCIS) to invasive ductal carcinoma (IDC) is a critical event in breast cancer, yet the spatial transcriptomic programs and microenvironmental context that orchestrate this transition remain poorly resolved [51]. A key challenge lies in the intrinsic heterogeneity of DCIS and the difficulty in identifying subtle, pre‐malignant changes within histologically normal‐appearing tissue [52]. Conventional histology, while foundational, offers limited molecular insight. To uncover the hidden spatial heterogeneity and molecular dynamics at the tumor‐normal interface, we applied SAGE to a human breast cancer dataset [23]. We adopt the manual annotation provided by Fu et al. [19] to support evaluation. Based on H&E‐stained images, pathologists delineate 20 tissue domains (Figure 5a), further grouped into four main morphology categories (Figure 5b): ductal/lobular carcinoma in situ (DCIS/LCIS), invasive ductal carcinoma (IDC), normal tissue (Healthy), and tumor edge. These annotations serve as spatial priors essential for evaluating tumor heterogeneity and method performance.

We compare SAGE with eight benchmark methods, evaluate clustering accuracy using ARI and NMI, and visualize the performance of each method (Figure 5c; Figure S37c). Among all methods, SAGE achieves the best performance (ARI = 0.64, NMI = 0.70), improving by approximately 14.33% over the second‐best SpaGCN (ARI = 0.56) and 1.48% over PROST (NMI = 0.69), respectively. SAGE accurately delineates regions of IDC and DCIS/LCIS, aligning well with expert annotations. In contrast, the clustering results of stLearn, SpaGCN, STAGATE, and GraphST exhibit spatial fragmentation and fail to resolve the tumor edge. While SEDR, SCAN‐IT, and PROST exhibit relatively high spatial consistency, their results still show considerable deviations from the manually annotated tumor boundaries. For example, SCAN‐IT shows over‐clustering in IDC_4, while PROST displays a similar issue in IDC_6.

Using SAGE's integrated analysis, we identify two key layers of spatial heterogeneity with potential biological and clinical significance (Figure 5d; Figure S38c; Data S7). First, within DCIS/LCIS regions, a distinct transcriptomic module (Topic 13) is active in subtypes 1, 2, and 5 but absent in subtype 4. This module is defined by concurrent upregulation of *TFF1* (luminal), *KRT6B* (basal), and *SERPINA3* (microenvironmental regulator), revealing a luminal–basal hybrid phenotype that has not previously been resolved in situ. The presence of this hybrid program, together with pro‐invasive signals, suggests that these DCIS subtypes represent a transcriptionally “primed” state with higher propensity for progression [53], whereas subtype 4 appears more indolent. Second, SAGE dissects the pathologist‐annotated “Healthy_1” tissue into two sub‐clusters (Cluster 14: 156 spots; Cluster 7: 230 spots; Figure 4e), one of which (Cluster 14) shows pronounced signatures of extracellular matrix (ECM) remodeling (*COL1A1*, *COL1A2*, *BGN*) and mesenchymal transition—hallmarks of an activated precancerous niche embedded within morphologically normal tissue [54] (Datas S8–S10). This cryptic structure is uniquely detectable by SAGE due to its sensitivity to subtle, coordinated gene‐expression patterns that other methods obscure. Together, these findings provide spatial evidence of hybrid phenotypes within DCIS and reveal a hidden precancerous microenvironment in histologically normal regions, offering fresh insight into the earliest stages of breast cancer evolution.

### 2.6 SAGE Unveils Fine‐Grained Tumor‐Microenvironment Interactions in Zebrafish Melanoma

**FIGURE 6:**
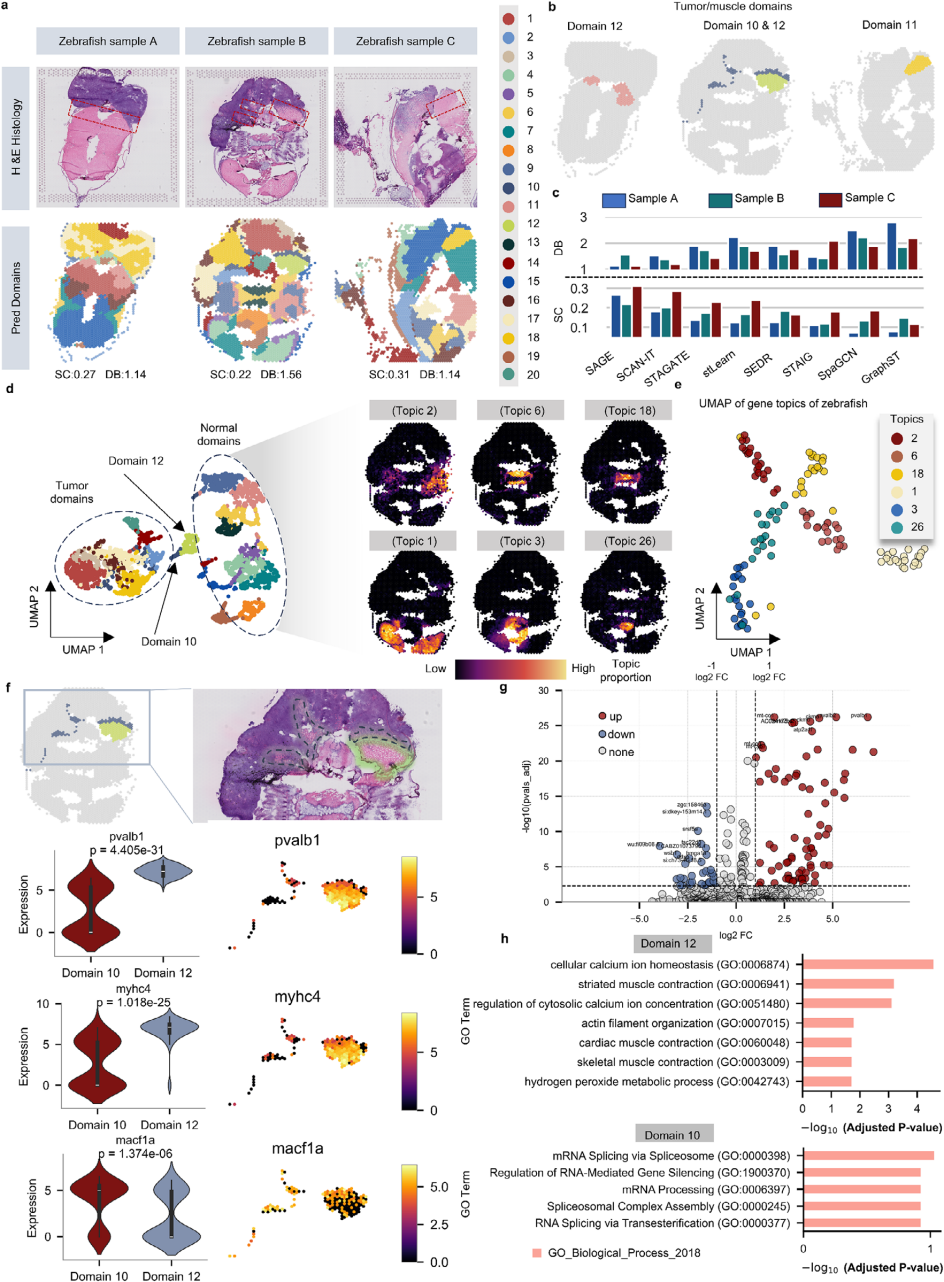
Fine‐grained segmentation of the tumor–muscle interface domain in zebrafish melanoma dataset using SAGE. (a) H&E‐stained image of zebrafish melanoma (Hunter et al., 2021), with tumor–muscle interface marked by red dashed box (top). Domains identified by SAGE on three samples (bottom). Baseline methods results are shown in Figures S39–S41 c. (b) Interface domains identified by SAGE across three samples. (c) Clustering performance (SC, DB) of SAGE and eight baselines on three samples (samples A and C contain 13 clusters each; sample B contains 20 clusters). (d) UMAP visualization of sample B showing tumor (left), normal (right), and interface domains (domains 10, 12). Related topics and marker genes are provided in Figures S42–S44. (e) UMAP of the top 20 genes (z‐score > 1.96) from six normal tissue topics. (f) Spatial maps and violin plots of DEGs (pvalb1, myhc4, macf1a) in domains 10 and 12. (g) DGE analysis between domains 10 and 12. Each point represents a gene; the x‐axis indicating log2 fold change and the y‐axis representing –log10 (*p*vals_adj). (|log2FC| > 1 and *p*vals_adj < 0.005, Wilcoxon test). (h) GO enrichment of DEGs between domains 10 and 12 (GO_Biological_Process_ 2018). The y‐axis denotes GO terms, and the x‐axis indicates –log10 *p*vals_adj (Benjamini–Hochberg).

The interface between a tumor and its surrounding normal tissue is the critical frontline of cancer invasion. However, the precise spatial transcriptomic architecture of this interface—whether it represents a simple, graded transition or is composed of distinct functional compartments—remains a fundamental open question. To address this, we apply SAGE to a zebrafish melanoma model [53], a powerful system for studying tumor–microenvironment interactions. We analyze three samples A, B, and C (Hunter et al., 2021), each associated with H&E‐stained images and corresponding manual annotations (Figure 6a), which delineate tumor tissue, normal tissue, and the tumor–muscle interface domain. This interface domain, highlighted by red boxes, corresponds to the transition region at the tumor's invasive front.

We first assess the ability of various methods to resolve the complex structure at the tumor‐invasive front. The spatial domains inferred by SAGE exhibit the closest alignment with manual annotations across all samples (Figure 6a,b; Figures S39–S41a). In samples A and C (13 clusters each), most methods approximate the interface, with slight over‐clustering or inclusion of extra muscle cells. More importantly, as tissue complexity increases (Sample B; 20 clusters), benchmark methods (SEDR, STAGATE, GraphST, SCAN‐IT, and STAIG) either oversimplify the interface as a single region or generate fragmented clusters. By contrast, SAGE consistently recovers a coherent but subdivided structure. UMAP embeddings further show that SAGE clearly separates tumor and normal tissue regions (Figure 6b; Figures S39–S41c). Moreover, we quantitatively assess clustering performance across the three slides using Silhouette Coefficient (SC) and Davies–Bouldin (DB) indices. SAGE achieves the highest clustering consistency (SC = 0.26; DB = 1.28), corresponding to improvements of 18.18% and 6.57%, respectively, over the second‐best method, SCAN‐IT (SC = 0.22; DB = 1.37) (Figure 6c; Figures S39–S41b; Table S28).

To evaluate how well the learned embeddings preserve spatial structure, we apply UMAP for low‐dimensional visualization. In sample B, SAGE clearly separates tumor and normal tissue regions, with strong agreement to manual annotations, especially at the tumor–normal interface (Figure 6d). To molecularly decipher the spatial organization revealed by SAGE's embeddings, we analyze the learned gene co‐expression topics. These topics map to broad anatomical regions such as the skin, heart, and pancreas (Figures S42–S44b), and their top‐associated genes form distinct clusters in transcriptomic space, confirming their specificity. Notably, we also observe shared signatures between functionally related tissues; for instance, the angiogenesis marker *VWF* is co‐enriched in topics corresponding to the heart and intestine, reflecting common vascular programs [55, 56]. This ability to capture both tissue‐specific and shared transcriptional modules provides a robust foundation for interpreting finer‐scale spatial domains.

Notably, in sample B, SAGE reveals that the tumor–muscle interface is not a unitary transition zone but is composed of two functionally distinct spatial domains—a tumor‐proximal “leading edge” (Domain 10) and a muscle‐proximal “stress zone” (Domain 12), which refines the interface domain manually annotated by Hunter et al. [53]. H&E‐stained images (Figure 6f) show that Domain 10 is located closer to the tumor region, while Domain 12 lies nearer to the normal muscle tissue. This segmentation more accurately captures the spatial complexity of the interface domains. To investigate the transcriptional heterogeneity, we analyze differentially expressed genes and enriched GO biological processes in Domains 10 and 12 (Figure 6f,g; Figure S45; Datas S11–S13). Domain 12 upregulates genes such as *pvalb1*, *myhc4*, *hspb6*, and *trim63a*, linked to skeletal muscle contraction, cellular stress response, and protein turnover. GO enrichment highlights muscle contraction, actin filament organization, and oxidative stress responses. In contrast, Domain 10 upregulates genes like *macf1a*, *pmela*, *wsb1*, and *ptprc*, indicating cytoskeletal remodeling, tumor‐specific gene activity, and immune infiltration [57, 58]. Enriched GO terms related to mRNA splicing, RNA processing, and post‐transcriptional gene silencing suggest active regulation in the tumor microenvironment. These findings reveal distinct transcriptional states in close proximity, offering a refined view of the tumor–muscle interface. This interplay between tumor‐driven “push” and the host tissue “response” provides a more nuanced understanding of invasion.

Collectively, these results establish a bipartite organizational principle at the tumor‐invasive front, moving beyond the concept of a simple transition zone. The discovery of functionally distinct “leading edge” and “stress response” domains provides a new spatial framework for understanding cancer invasion as a structured interplay between aggressive progression and host tissue defense (Figure S54).

## 3 Discussion

Although spatial transcriptomics (ST) has greatly advanced our understanding of tissue architecture and cellular heterogeneity, existing methods fall short in resolving complex spatial patterns, modeling long‐range functionally related regions, and interpreting spatial domains in a biologically meaningful manner. In particular, while methods like SpaGCN, STAGATE, and GraphST have made significant progress, they are often limited by their reliance on a single integrated graph, which can obscure the modeling of non‐local functional relationships and hinder biological interpretability. In this study, we introduce SAGE, a unified spatial domain analysis framework that integrates multi‐resolution clustering, topic modeling, and GNNs. SAGE aims to overcome existing limitations by selecting cluster‐relevant feature genes and learning embeddings through dual‐view feature fusion. This approach enables more accurate identification and interpretation of complex spatial tissue structures and cellular functional states, thereby enhancing the interpretability of ST data.

SAGE is designed to emphasize the critical role of feature (gene) selection in guiding spatial domain segmentation. Unlike existing methods that rely on single features (e.g., HVGs or SVGs) or static graph structures, SAGE identifies high spatial‐specificity genes (HSGs) through the integration of multi‐dimensional features, improving both the accuracy and biological interpretability of domain segmentation. Furthermore, through topological optimization and dual‐view graph embedding learning, SAGE provides a more comprehensive perspective for dissecting tissue heterogeneity.

Through benchmarking on diverse ST datasets against other representative methods, SAGE consistently demonstrates outstanding performance in both algorithmic accuracy and biological interpretability. The framework enables precise spatial domain segmentation and reveals interpretable gene co‐expression patterns, establishing it as a powerful tool for dissecting complex spatial architectures and tissue heterogeneity. SAGE's successful application in cross‐platform spatial technologies (e.g., MERFISH [4], Slide‐seq [5]) and complex biological contexts (such as tumor microenvironments) highlights its potential as a universal and efficient tool for spatial domain analysis.

The spatial domain segmentation results produced by SAGE not only recapitulate known tissue annotations but also uncover previously unrecognized heterogeneity. SAGE accurately identifies the trilaminar organization of the cerebellar cortex and distinguishes the hippocampus in mouse brain slides, uncovering hidden heterogeneity in human breast tumor tissue. In rice embryonic spatial transcriptomics, using the spatiotemporal transcriptomic atlas of rice embryonic cells during seed germination (Yao et al., 2024 [59]), SAGE recapitulates major embryonic compartments and their reorganization across germination stages and achieves higher agreement with anatomical annotations than competing methods, underscoring its applicability to plant developmental biology

Moreover, the application of SAGE in complex biological systems such as zebrafish melanoma sections demonstrates its practical utility in identifying tumor‐normal transition zones, spatial heterogeneity, and differences in cellular states. It also uncovers shared vascular signatures between anatomically distinct regions like the heart and intestine, highlighting functional interactions across tissues. These findings further underscore the broad applicability and strengths of SAGE in elucidating diverse biological processes.

In conclusion, SAGE provides a powerful and precise tool for analyzing multi‐scale functional structures and tissue heterogeneity within ST. By deepening our systematic understanding of functional regions and tissue heterogeneity, SAGE is poised to advance studies of tissue microenvironment mechanisms and disease progression, offering potential guidance for disease diagnosis, prognosis, and precision therapy strategies. Moreover, the framework demonstrates strong adaptability across scales and species, making it suitable for a wide range of biological systems, from normal tissue homeostasis to pathological transformations. Future work can further refine the algorithm to handle larger, more complex datasets and explore its application in emerging fields such as single‐cell transcriptomics and multi‐omics integration.

## 4 Methods

### 4.1 Data Preparation

SAGE takes a gene expression matrix $X\in R^{N\times G}$ as input, where *N* is the number of spatial locations (e.g., spots or cells depending on the platform) and *G* is the number of genes, with *X_ij_* denoting the expression (UMI count) of gene *j* at location *i*. The spatial coordinate matrix $S\in R^{N\times 2}$ records the 2D position of each location. Together, D=(X,S) defines the spatial transcriptomics dataset. SAGE does not rely on species‐specific assumptions and can therefore be directly applied to both animal and plant spatial transcriptomics. In this study, all datasets—including human, mouse and the rice embryonic ST data during seed germination—are processed with the same pipeline to construct *X* and *S*, and SAGE shows accurate recovery of major embryonic compartments and their reorganization across stages on the rice datasets (Figure S53; Data S14), illustrating its cross‐species applicability.

### 4.2 Data Preprocessing

Raw gene expression counts are preprocessed using the SCANPY package [60]. Genes detected in fewer than 5 spatial locations or with total counts ≤20 are removed. Highly variable genes (HVGs) are identified using the Seurat v3 method (as implemented in SCANPY), and the top 3,000 HVGs (optional) are selected for downstream analysis. Expression vectors are library‐size normalized to 10 000 counts per spot and subsequently log‐transformed using the natural logarithm. Specifically, let $X_{ij}^{\left(\mathrm{norm}\right)}$ denote the normalized expression level of gene *j* in spot *i*. The log‐transformed expression is then computed as $X_{ij}^{\left(\log\right)}=\log\left(X_{ij}^{\left(\mathrm{norm}\right)}+1\right)$, which stabilizes variance and reduces the impact of outliers.

### 4.3 Multi‐Resolution Consensus Clustering

We select the top 3 000 HVGs from the preprocessed matrix and apply Principal Component Analysis (PCA) to obtain the top 200 principal components (optional). We perform multi‐resolution clustering using Leiden algorithms (default) to capture spatial domains with varying structural characteristics. Specifically, Leiden clustering is applied across a range of resolutions to detect modular and well‐separated domains through resolution tuning. In contrast, for tissues exhibiting continuous gradients or layered architectures (e.g., DLPFC and Mouse Brain), we additionally employ a Gaussian mixture model‐based clustering (Mclust), which is better suited for identifying smooth spatial transitions and subtle domain boundaries. In the Mclust setting, we set the number of clusters based on prior biological knowledge or baseline clustering results, and explore a small range of values around this target to identify the most consistent clustering solution. Each clustering result is represented by a label vector $l^{\left(t\right)}=\left(l_{1}^{\left(t\right)},l_{2}^{\left(t\right)},\text{…},l_{N}^{\left(t\right)}\right)$, where t∈{1,2,…,r} indexes the clustering result obtained from the *t*‐th run. Here *r* denotes the total number of clustering results used to build the consensus matrix. To measure the co‐occurrence frequency of cell pairs across these clustering results, we compute the consensus matrix $C\in {\left[0,1\right]}^{N\times N}$, where each element is given by:

(1) $$C_{ij}=\frac{1}{r}\;\sum_{t=1}^{r}I\left(\;l_{i}^{\left(t\right)}=l_{j}^{\left(t\right)}\;\right)$$

where $I\;(\;l_{i}^{\left(t\right)}=l_{j}^{\left(t\right)}\;)=1$ if location *i* and *j* are assigned to the same cluster in clustering result *t* and 0 otherwise. *C_ij_* measures the consensus frequency between locations *i* and *j*. Finally, to obtain the preliminary spatial domain labels, we apply the Leiden clustering algorithm to a graph with edge weights derived from the consensus matrix *C*. The resulting cluster labels $L_{\mathrm{init}}\in {\left\{1,\text{…},N_{\mathrm{cluster}}\right\}}^{N}$ are used as the initial domain assignments for downstream topic selection and topological refinement, where *N*_cluster_ is the number of inferred spatial domains. Each element *L*_init_ indicates the domain assignment of location *i*.

### 4.4 NMF Topic Modeling

SAGE applies non‐negative matrix factorization (NMF) using the scikit‐learn package to decompose the preprocessed gene expression matrix $X\in R^{N\times G}$. The number of latent topics *N_topics_* is set to 30 by default. We empirically validate the robustness of SAGE to this hyperparameter (Figure S46b). Given the inherent sparsity of ST data, particularly the prevalence of zero expression values, we adopt non‐negative double singular value decomposition (NNDSVD) for initialization to improve stability and convergence. The decomposition yields two non‐negative matrices: (1) $W_{\mathrm{nmf}}\in R^{N\times N_{\mathrm{topics}}}$, representing topic weights per spot/cell; (2) $H_{\mathrm{nmf}}\in R^{N_{\mathrm{topics}}\times G}$, capturing gene contributions to each topic. To simplify notation, we refer to *W*_nmf_ as *Z* and the transpose of *H*_nmf_ as *H*, where $Z\in R^{N\times N_{\mathrm{topics}}}$ and $H\in R^{G\times N_{\mathrm{topics}}}$. To facilitate downstream comparison, the columns of *Z* are normalized to the range [0, 1] using column‐wise min‐max normalization. This factorization enables the separation of the original gene expression matrix into latent spatial topic patterns (*Z*) and their associated gene‐level contributions (*H*).

### 4.5 Topic Selection via Supervised Learning

To enhance spatial coherence and discriminability of extracted topics, SAGE proposes a two‐step filtering procedure on the NMF‐derived topic matrix $Z\in R^{N\times N_{\mathrm{topics}}}$. Given the spatial structure inherent of gene expression, we first assess the spatial autocorrelation of each topic using Moran's *I* statistic. Let $S\in R^{N\times 2}$ denote spatial coordinates. For each topic vector $z_{i}\in R^{N}$(i.e., the *i*‐th column of *Z*), Moran's *I* is computed as:

(2) $$MRI_{i}=\frac{N}{W}\;\cdot \frac{\sum_{j=1}^{N}\sum_{l=1}^{N}w_{jl}\left(z_{ij}-{\bar{z}}_{i}\right)\left(z_{il}-{\bar{z}}_{i}\right)}{\sum_{j=1}^{N}{\left(z_{ij}-{\bar{z}}_{i}\right)}^{2}}$$

where ${\bar{z}}_{i}=\frac{1}{N}\;\sum_{j=1}^{N}Z_{ij}$ is the mean of topic vector *z_i_*, *w_jl_* denotes the spatial weight between positions *j* and *l*, computed based on Euclidean distance. $W=\sum_{j,l}z_{jl}$ is the total weight.

Topics with *MRI_i_* ≥  *T*_MRI_ =  0.2 are retained to form the filtered topic matrix $Z_{\mathrm{filtered}}\in R^{G\times N_{\mathrm{filtered}}}$. We empirically observe that topics with low Moran's *I* values tend to lack spatial structure and biological interpretability.

Although topics are initially filtered using the MRI value, some high‐MRI topics may result from local noise. To remove these spurious components, we further evaluate each topic's discriminative power using a random forest (RF) classifier trained on domain labels. RF is chosen because it provides feature importance scores, allowing us to directly evaluate each topic's contribution to spatial domain discrimination. Topics with low importance scores are discarded, ensuring the final set is both spatially coherent and biologically relevant. Specifically, we assess the discriminative power of each topic in the filtered matrix $Z_{\mathrm{filtered}}\in R^{N\times N_{\mathrm{filt}\_t}}$ using a random forest classifier, where $N_{\mathrm{filt}\_t}$ is the number of retained topics. The preliminary domain labels $L_{\mathrm{init}}\in {\left\{1,\text{…},N_{\mathrm{cluster}}\right\}}^{N}$ serve as classification targets. A classifier $f:R^{N\times N_{\mathrm{filt}\_t}}\to {\left\{1,\text{…},N_{\mathrm{cluster}}\right\}}^{N}$ is trained using the default configuration in scikit‐learn (v1.7.0), with the number of decision trees set to (T = 100). After training, the importance of each topic is computed based on the total Gini impurity reduction it contributes across all trees. Let splits(*tr*) denote the set of split nodes in a tree *tr*, and let the Gini importance of topic *i* be defined as:

(3) Impi=1T∑t=1T∑s∈splitstδis·ΔGinis

where T = 100 is the number of decision trees, ΔGini_*s*_ is the decrease in Gini impurity at split node *s*, and $\delta_{i}^{\left(s\right)}=I\left(i\in \mathrm{topic}s_{s}\right)$, with features_*s*_ denoting the set of topics available at node *s*. The indicator function $\delta_{i}^{\left(s\right)}$ equals 1 if topic *i* is used in split *s*, and 0 otherwise. To retain only the most informative topics, we discard the bottom fraction (here *p*  =  0.2) of topics based on importance scores and select the top (1 − *p*) × 100% to form the final topic matrix $Z_{\mathrm{final}}\in R^{N\times N_{\mathrm{sel}\_t}}$, where $N_{\mathrm{sel}\_t}=\left\lfloor \left(1-p\right)\cdot N_{\mathrm{filt}\_t}\right\rfloor$. This refined matrix is used in subsequent gene selection and graph‐based spatial refinement. Notably, the RF classifier here is used solely as an internal topic‐importance estimator and is trained on the full dataset, without train/validation/test splitting or supervised performance evaluation, and we further show in Figure S47 that the RF‐based topic ranking is robust to perturbations of the preliminary labels *L*_init_.

### 4.6 Construction of High Spatial‐Specificity Genes (HSGs)

The identification of HSGs in SAGE comprises four major steps: 1) selecting topic‐specific genes based on their contributions to latent topics; 2) preliminary filtering to remove non‐topic‐specific genes; 3) spatial consensus‐driven joint optimization; and 4) final assembly of highly specific genes. Specifically, given the topic representation matrix *Z*_final_, SAGE proceeds through the following detailed steps.

1. Given $Z_{\mathrm{final}}\in R^{N\times N_{\mathrm{sel}\_t}}$, we refer back to the original gene–topic weight $H\in R^{G\times N_{\mathrm{topics}}}$ derived from the NMF decomposition, where *G* denotes the total number of genes and *N*_topics_ is the total number of topics. Based on the selected topic indices $T\subset \{1,\text{…},\;N_{\mathrm{topics}}\}$, which corresponds to the columns of *Z*_final_, we extract the corresponding submatrix $H_{T}\in R^{G\times N_{\mathrm{sel}\_t}}$ from the original gene–topic weight matrix *H*. For each topic t∈T, column‐wise normalization is performed as follows: $\tilde{H_{g,t}}=\frac{H_{g,t}}{\sum_{g^{\prime }=1}^{G}H_{g^{\prime },t}}\;,\;\forall g\in \left\{1,\;2,\text{…},\;G\right\}$ yielding the normalized gene–topic contribution matrix $\tilde{H_{T}}\in R^{G\times N_{\mathrm{sel}\_t}}$. Each column of $\tilde{H_{T}}$ is then sorted in descending order to determine a rank of each gene's contribution within its corresponding topic, forming a rank matrix: *R*_*g*,*t*_ = *rank_t_* (*g*), where *rank_t_* (*g*)= 1 indicates the gene *g* with the highest normalized contribution in topic *t*, and larger rank values indicate lower contributions.
2. For each topic *t*, SAGE selects genes based on their contribution ranks, and then defines the lower and upper rank thresholds $r_{t}^{\left(l\right)}$ and $r_{t}^{\left(u\right)}$ as the smallest integers such that the cumulative contributions exceed the specified quantile thresholds τ_*l*_ and τ_*u*_, respectively:
(4) $$r_{t}^{\left(l\right)}=\min\left\{r\in \left\{1,\text{…},G\right\}\;|\;\sum_{g=1}^{r}\tilde{H_{g,t}}\geq \tau_{l}\right\}$$
(5) $$r_{t}^{\left(u\right)}=\min\left\{r\in \left\{1,\text{…},G\right\}\;|\;\sum_{g=1}^{r}\tilde{H_{g,t}}\geq \tau_{u}\right\}$$

(6) $$Z_{g,t}=\left|\frac{H_{g,t}-\mu_{t}}{\sigma_{t}}\right|,\;\mu_{t}=E\left[H_{\cdot ,t}\right],\;\sigma_{t}=\;\mathrm{Std}\left[H_{\cdot ,t}\right]$$

where *r* ranges from 1 to *G*. These quantile thresholds are empirically chosen to balance sensitivity and specificity in gene selection and have demonstrated stable performance across a range of datasets. Genes within the top $r_{t}^{\left(l\right)}$ ranks are further evaluated through z‐score normalization based on the final gene–topic weight matrix $H_{T}$:

We define the set of topic‐specific genes (TSGs) as:

(7) $$G_{\mathrm{TSG}}=\left\{g\in G\;|\;R_{g,t}\langle r_{t}^{\left(l\right)}\wedge Z_{g,t}\rangle \theta \right\}\;,\;t\in T$$

where *R*_*g*,*t*_ denotes the rank of gene *g* in topic *t*. We set θ  =  1.96, corresponding to the 95% two‐tailed cutoff under standard normal assumption, to filter statistically enriched genes. A gene is considered non‐topic‐specific if it consistently ranks below the upper threshold $r_{t}^{\left(u\right)}$ across all topics. Formally, the preliminary set of Non‐TSGs is defined as:

(8) $$G_{\mathrm{Non}-\mathrm{TSG}}^{\mathrm{Init}}=\left\{g\in G_{\mathrm{HVG}}\;|\;R_{g,t}>r_{t}^{\left(u\right)},\forall t\in T\right\}\;$$

1. Although NMF‐based topic decomposition effectively captures dominant spatial patterns, it can potentially overlook genes with strong spatial autocorrelation due to rank inconsistency or decomposition bias. To address this limitation, SAGE further refines the candidate gene set using a joint filtering strategy that incorporates both spatial autocorrelation and topic rank consistency. This step aims to recover spatially coherent genes that are underrepresented in the initial decomposition. Specifically, for each gene *g*, we compute its total topic rank sum $S_{g}=\sum_{t=1}^{T}R_{g,t}$, where T=|T| is the number of selected topics, and *R*_*g*,*t*_ denotes the rank of gene *g* within topic *t* (lower values indicating higher topic relevance). We then identify genes with high spatial autocorrelation but low topic relevance by selecting those that satisfy:
(9) $$G_{\mathrm{Non}-\mathrm{TSG}}^{\mathrm{Del}}=\left\{g\in G_{\mathrm{Non}-\mathrm{TSG}}^{\mathrm{Init}}\;|\;MRI_{g}>\theta_{\mathrm{mri}}\wedge S_{g}<\theta_{s}\right\}\;$$

1. The final set of non‐topic‐specific genes is defined as: $G_{\mathrm{Non}-\mathrm{TSG}}=G_{\mathrm{Non}-\mathrm{TSG}}^{\mathrm{Init}}\;\setminus G_{\mathrm{Non}-\mathrm{TSG}}^{\mathrm{Del}}$. To construct the final set of high spatial‐specificity genes (HSGs), we begin with the union of highly variable genes (HVGs) and topic‐specific genes (TSGs):
(10) $$G_{\mathrm{Add}}=G_{\mathrm{HVG}}\cup G_{\mathrm{TSG}}$$

where θ_*mri*_ and θ_*s*_ are empirically defined thresholds based on the distribution of Moran's *I* scores *MRI_g_* and *S_g_* values, respectively. These genes are removed from the non‐topic‐specific set $G_{\mathrm{Non}-\mathrm{TSG}}^{\mathrm{Init}}$ to ensure that spatially informative genes are not excluded due to model bias.

This unified candidate set incorporates both statistically variable and topic‐relevant genes. However, a subset of genes within this union may exhibit low spatial specificity—particularly those from HVG that are not topic‐specific. To address this, we define a refinement set $G_{\mathrm{De}l^{*}}\subseteq G_{\mathrm{HVG}}\cap G_{\mathrm{Non}-\mathrm{TSG}}$, where gene $g\in \;G_{\mathrm{De}l^{*}}$ is selectively removed based on a high value in *R*_*g*,*t*_.

Here *R*_*g*,*t*_ denotes the rank of gene *g* in topic *t*, with smaller values indicating higher topic contribution. These genes are excluded from the candidate set to ensure biological specificity:

(11) $$G_{\mathrm{HSG}}=G_{Add}\;\setminus G_{\mathrm{De}l^{*}}.$$

To preserve interpretability and ensure that gene filtering does not remove more information than it adds, we impose the following constraint on the size of the removal set:

(12) $$\left|G_{\mathrm{De}l^{*}}\right|\leq \left|G_{\mathrm{Add}}\right|-\left|G_{\mathrm{HVG}}\right|,$$

where (|*G*_Add_|) denotes the number of genes in unified candidate set ($G_{\mathrm{HVG}}\cup \;G_{\mathrm{TSG}}$) and (|*G*_HVG_|) is the number of HVGs. This condition ensures that the number of genes removed never exceeds the number of informative topic‐specific genes added, guaranteeing a net gain in spatially relevant gene content.

### 4.7 Consensus‐Driven Graph Construction

SAGE introduces a consensus‐driven graph construction strategy that integrates spatial proximity and feature consensus. This process involves two main steps:

1. Construction of the spatial‐based adjacency graph (SAG): SAGE first constructs a *k*‐nearest neighbor (*k*‐NN) graph based on spatial coordinates. Let $x_{i}\in R^{2}$ denote the 2D spatial coordinates of spot *i* and define $N_{k}\left(i\right)$ as the set of *k* nearest neighbors of *i* in Euclidean space. The initial adjacency matrix is constructed as: $A_{\mathrm{coord}}^{\mathrm{init}}\left(i,j\right)=\{\begin{array}{cc} 1, & \mathrm{if}\;j\in N_{k}\left(i\right)\\ 0, & \mathrm{Otherwise} \end{array}$. Additional, to support effective spatial information propagation in downstream graph‐based operations, the adjacency matrix $A_{\mathrm{coord}}^{\mathrm{init}}\left(i,j\right)$ is symmetrized via element‐wise maximum with its transpose to enable undirected connections. To reduce redundant edges across tissue boundaries, SAGE introduces an edge‐pruning strategy. Given the consensus matrix $C\in R^{N\times N},\;C\left(i,\;j\right)\in \left[0,1\right]$, derived from multi‐resolution consensus clustering, we define the pruned spatial adjacency matrix *A*_coord_ by thresholding the initial spatial adjacency matrix $A_{\mathrm{coord}}^{\mathrm{init}}$ as follows:
(13) $$A_{\mathrm{coord}}\left(i,j\right)=\{\begin{array}{cc} A_{\mathrm{coord}}^{\mathrm{init}}\left(i,j\right), & \mathrm{if}\;C\left(i,j\right)\geq \tau \\ 0, & otherwise \end{array}$$

1. Construction of the feature‐based adjacency graph (FAG): To complement the spatial adjacency structure, SAGE constructs a feature‐based adjacency graph $A_{\mathrm{feat}}\in {\left\{0,1\right\}}^{N\times N}$, integrating both consensus co‐assignment probability and expression‐level similarity.

where τ  =  0.2 is a confidence threshold, empirically selected based on performance in Figure S50. Here, *C*(*i*, *j*) denotes the frequency with which spots/cells *i* and *j* are co‐assigned to the same cluster across different resolutions or cluster numbers during consensus clustering. A low consensus score indicates that the two spots/cells are rarely grouped together across clustering outcomes, suggesting potential transcriptional divergence or boundary‐like properties. Removing such low‐confidence edges, this step helps eliminate noise and spurious links, thereby preserving only spatially coherent relationships for downstream graph‐based modeling.

For each spot *i*, the consensus‐based neighbor set is initially defined as: $N_{\mathrm{cons}}\left(i\right)=\left\{j\neq i|C(i,j)=1\right\}$. If $|N_{\mathrm{cons}}\left(i\right)|<n_{\mathrm{neighbors}}$, additional spots with the next highest consensus scores *C*(*i*, *j*) < 1 are appended until $|N_{\mathrm{cons}}\left(i\right)|\geq n_{\mathrm{neighbors}}$. Here, *n*_neighbors_ is a predefined hyperparameter that controls the maximum number of candidate neighbors per spot. We investigate the effect of this parameter choice in Figure S50. Simultaneously, a similarity‐based neighbor set $N_{\mathrm{feat}}\left(i\right)$ is constructed by selecting the top *n*_neighbors_ spots (j≠i) with the highest cosine similarity to spot *i*’s feature vector *x_i_*, where $x_{i}\in \;R^{N_{\mathrm{sel}\_t}}$ denotes the feature vector of spot *i*. The final candidate set is given by:

(14) $$N_{\mathrm{final}}\;\left(i\right)=N_{\mathrm{cons}}\;\left(i\right)\cap N_{\mathrm{feat}}\left(i\right)$$

For each node pair (*i*, *j*) in $N_{\mathrm{final}}\left(i\right)$, if the following conditions hold:

(15) $$i\neq j,\;\mathrm{De}g_{\mathrm{add}}\left(i\right)<n_{\mathrm{neighbors}},\;\mathrm{De}g_{\mathrm{add}}\left(j\right)<n_{\mathrm{neighbors}}$$

then set *A*_feat_(*i*, *j*) = 1 and *A*_feat_(*j*, *i*) = 1, otherwise 0. Here,  Deg_add_(*i*) denotes the number of newly added (non‐local) edges for node *i*. This construction strategy ensures that only transcriptionally similar and structurally plausible connections are retained, enriching the graph with biologically meaningful non‐spatial relationships that are absent in the spatial adjacency.

### 4.8 Dual‐View Graph Neural Network

We propose a dual‐view graph convolutional network that jointly learns from the SAG and FAG. As shown in Figure 1b, the dual‐view graph convolutional network comprises two GCN encoders, a self‐attention fusion module, and three symmetric decoders. The encoders extract view‐specific embeddings, which are fused into a unified representation and decoded to reconstruct the input.

After constructing the SAG, FAG, and the feature matrix $X_{\mathrm{HSG}}\in R^{N\;\times \;d_{\mathrm{in}}}$, where *d*_in_ denotes the number of high spatial‐specificity genes (HSGs) selected through the prior gene selection pipeline, SAGE employs two separate graph convolutional network (GCN) encoders to learn spot representations from spatial and feature perspectives. Each encoder consists of two layers: a GCN layer with *d*_hid_ hidden units, followed by a linear transformation projecting the output to a latent embedding of dimension *d*_out_. The GCN operation for each view is defined as:

(16) $$\begin{array}{c} \;H_{1}^{\left(v_{k}\right)}=\sigma \left({\tilde{A}}^{\left(v_{k}\right)}X_{\mathrm{HSG}}W_{1}\right),\;k=1,\;2 \end{array}$$

where ${\tilde{A}}^{\left(v_{k}\right)}$ is the normalized adjacency matrix for view (A_coord_ or A_feat_), $W_{1}\;\in R^{d_{\mathrm{in}}\;\times \;d_{\mathrm{hid}}}$ is a learnable weight matrix, and σ(·) denotes the ReLU activation function. The final view‐specific embeddings are computed as:

(17) $$\begin{array}{c} \;E^{\left(v_{k}\right)}=H_{1}^{\left(v_{k}\right)}\;W_{2}+b_{2},\;k=1,\;2 \end{array}$$

where $W_{2}\;\in R^{d_{\mathrm{hid}}\;\times \;d_{\mathrm{out}}}$ and $b_{2}\in$ $R^{d_{\mathrm{out}}}$ are shared learnable parameters across views. As a result, this yields two node embeddings: $E^{\left(v_{1}\right)}$ from SAG and $E^{\left(v_{2}\right)}$ from FAG, both in $R^{N\times d_{\mathrm{out}}}$. To integrate the two views, SAGE applies a self‐attention fusion layer. The two spot embeddings from the spatial and feature views are stacked into a tensor $E_{stack}\in R^{N\times 2\times d_{\mathrm{out}}}$. For each spot *i*, the view‐specific embeddings are passed through a nonlinear transformation:

(18) $$v_{i}=tanh\left(E_{\mathrm{stack},i}\cdot W_{\omega }\right)\;,\;\alpha_{i}=softmax\left(v_{i}\cdot u_{\omega }\right)$$

where $W_{\omega }\in R^{d_{\mathrm{out}}\times d_{\mathrm{out}}}$, $u_{\omega }\in R^{d_{\mathrm{out}}\times 1}$ and $\alpha_{i}\in R^{2}$ denotes the attention weights across the two views for spot/cell *i*. The final fused embedding for each spot/cell *i* is computed as:

(19) $$E_{i}^{\left(v_{att}\right)}=\alpha_{i}^{1}\;\cdot E_{i}^{\left(v_{1}\right)}+\alpha_{i}^{2}\cdot E_{i}^{\left(v_{2}\right)}$$

Given the view‐specific embeddings $E^{\left(v_{1}\right)},E^{\left(v_{2}\right)}$, and the fused embedding $E^{\left(v_{att}\right)}$, SAGE employs three symmetric decoders to reconstruct the feature matrix $X_{\mathrm{HSG}}\in R^{N\times d_{\mathrm{in}}}$. Each decoder mirrors the encoder with two linear layers, using the transposed encoder weights:

(20) $$H^{\left(1\right)}=\sigma \left(E^{\left(k\right)}W^{{\left(2\right)}^{⊤}}\right),\;X_{HSG}^{\left(k\right)}=H^{\left(1\right)}\;W^{{\left(1\right)}^{⊤}},k\in \left\{v_{1},v_{2},v_{att}\right\}$$

where σ(·) is a nonlinear activation function ReLU, and *W* ^(1)^,*W* ^(2)^ are the encoder weights. All decoders aim to minimize the mean squared error (MSE) between reconstructed and original features.

### 4.9 Joint Optimization Objectives

To enable multi‐view embedding learning and feature reconstruction, the overall loss function comprises four components. The first component is the reconstruction loss. Each decoder reconstructs *X*_HSG_ from $E^{\left(v_{1}\right)}$, $E^{\left(v_{2}\right)}$, and the fused embedding $E^{\left(v_{att}\right)}$, yielding three reconstructions $X_{\mathrm{rec}}^{\left(k\right)}$, where *k* ∈ {*v*_1_,*v*_2_, *v_att_*}. The reconstruction loss is defined as the sum of MSE between reconstructed features and the original input:

(21) $$L_{\mathrm{recon}}=\sum_{k\in \{v_{1},v_{2},v_{att}\}}MSE\;\left(X_{\mathrm{HSG}},X_{\mathrm{rec}}^{\left(k\right)}\right)$$

To preserve the graph structure within the learned embeddings, SAGE compares the fused embedding $E^{\left(v_{att}\right)}$ with both the spatial and feature adjacency matrices. The reconstruction probabilities are estimated via a sigmoid function applied to the similarity matrix $E^{\left(v_{att}\right)}{E^{\left(v_{att}\right)}}^{⊤}$, and binary cross‐entropy (BCE) losses are computed:

(22) $$L_{\mathrm{graph}}=\;\mathrm{BCE}\left(A_{\mathrm{coord}},\sigma \left(E^{\left(v_{att}\right)}{E^{\left(v_{att}\right)}}^{⊤}\right)\right)+\mathrm{BCE}\left(A_{\mathrm{feat}},\sigma \left(E^{\left(v_{att}\right)}{E^{\left(v_{att}\right)}}^{⊤}\right)\right)$$

Here, loss $L_{\mathrm{graph}}$ encourages the fused embedding to preserve the original graph connectivity: connected nodes in the original graphs are embedded with high similarity, while unconnected nodes have low similarity.

To encourage cross‐view consistency, SAGE incorporates the SwAV mechanism for contrastive clustering between $E^{\left(v_{1}\right)}$ and $E^{\left(v_{2}\right)}$. After deriving cluster centers from $E^{\left(v_{att}\right)}$ via K‐Means, each view's embedding is assigned to these centers using softmax. The assignment matrices $Q^{\left(v_{1}\right)}$ and $Q^{\left(v_{2}\right)}$ are normalized using the Sinkhorn‐Knopp algorithm [35]. The SwAV loss is defined as the symmetric Kullback‐Leibler (KL) divergence between these distributions:

(23) $$L_{\mathrm{swav}}=\;\mathrm{KL}\left(Q^{\left(v_{1}\right)}|Q^{\left(v_{2}\right)}\right)+\mathrm{KL}\left(Q^{\left(v_{2}\right)}|Q^{\left(v_{1}\right)}\right)$$

To prevent dominance by a single view and enforce balanced attention, we introduce an attention regularization term. This term imposes a regularization constraint on the attention weights α learned during the fusion process, defined as:

(24) $$L_{\mathrm{att}}=E\left[{\left(\alpha -0.5\right)}^{2}\right]$$

The overall loss function is defined as a weighted sum of the above components:

(25) $$L_{\mathrm{total}}=\lambda_{1}\;L_{\mathrm{recon}}+\lambda_{2}L_{\mathrm{graph}}+\lambda_{3}L_{\mathrm{swav}}+\lambda_{4}L_{\mathrm{att}},$$

where λ_1_,λ_2_,λ_3_,λ_4_ are loss weights initialized to {10, 10, 5, 1}. SAGE adopts a staged dynamic weighting strategy, adjusting these coefficients during training to improve convergence and model performance.

### 4.10 Spatial Domain Identification

After model training, we perform spatial domain clustering analysis based on the latent embeddings $E^{\left(v_{att}\right)}$ generated by SAGE. Specifically, we use the Mclust algorithm (from the R package) or the Leiden algorithm (from the SCANPY package) to cluster each spot/cell. When the ground truth is known, we set the number of clusters to match the ground truth. In cases where the number of clusters is unknown, we conduct a grid search within the resolution range [0.1, 2.5] with a step size of 0.001, calculating the Calinski‐Harabasz (CH) score for each resolution and selecting the resolution with the highest score as the optimal value for clustering.

Although dual‐view embedding learning enhances feature expressiveness and clustering separability, we still observe that a small number of spots can be misassigned to incorrect spatial domains. We treat these cases as outliers arising from data noise or model uncertainty, which may affect downstream biological interpretation. To mitigate this, we introduce an optional post‐processing step in which suspected outliers are relabeled according to the most frequent cluster within their local spatial neighborhood, defined as the 25 nearest spatial neighbors (n = 25), i.e., the cluster with the highest local “purity”. This local reassignment can, however, introduce over‐smoothing in datasets with fine‐grained spatial structure or complex micro‐architecture. Therefore, we only apply this step to relatively coarse‐resolution, spot‐based Visium datasets (e.g., human DLPFC, human breast cancer, zebrafish melanoma), and omit it for high‐resolution molecule‐ or pixel‐based datasets(e.g., MERFISH, osmFISH, Stereo‐seq). A dataset‐level summary of whether local reassignment is used is provided in Supplementary Table S1. In practice, we recommend enabling this optional local reassignment only for coarse‐resolution, spot‐based datasets with robust macroscale domains, and disabling it for high‐resolution or highly complex tissues where preserving fine‐grained boundaries is a priority.

### 4.11 The Ablation Studies of SAGE

To systematically evaluate the contribution of key components in the SAGE framework, particularly the proposed high spatial‐specificity genes (HSG) selection strategy and the dual‐view neural architecture, we conduct comprehensive ablation experiments on the DLPFC dataset. Four experimental settings are tested: (1) a baseline model without HSG‐based gene selection and using only a single spatial view; (2) a model incorporating HSG‐based gene selection while still using a single view; (3) a model applying conventional HVG selection with a dual‐view architecture; and (4) the complete SAGE framework, which combined HSG‐based gene selection with the dual‐view design. As GraphST ranks second in clustering performance on the benchmark DLPFC dataset, it is used as the baseline method for comparison. The results show that both the HSG strategy and the dual‐view architecture contribute independently to substantial improvements in clustering performance, as evaluated by ARI and NMI. The complete SAGE model consistently outperforms all other configurations, demonstrating its superiority in spatial transcriptomics clustering tasks.

To further elucidate the role of the proposed HSG selection, we conducted detailed comparative analyses between HVGs and HSG‐based gene sets across all DLPFC slices. As shown in Figure S49, HSGs capture more spatially coherent patterns with higher Moran's I and lower Geary's C values compared to HVGs. Moreover, a cross‐method evaluation presented in Figure S50 demonstrates that integrating SAGE‐based feature selection universally improves clustering accuracy across multiple spatial analysis frameworks. In addition, to validate the parameter robustness of the topic extraction and topology optimization processes, we conduct extensive multi‐parameter robustness studies on these two modules within the SAGE framework. Detailed results and analyses can be found in Figures S46–S52.

### 4.12 Visualization of Dual‐View Embeddings and Attention Weights

To further illustrate the effect of the dual‐view fusion mechanism, we visualize the embeddings learned from each graph view and from their fused representation. For four representative datasets (DLPFC slice 151673, mouse brain anterior section, human breast cancer, and zebrafish melanoma sample B), we extract three types of embeddings from the trained SAGE model: the spatial‐view embedding from the spatial adjacency graph (SAG_emb), the feature‐view embedding from the feature adjacency graph (FAG_emb), and the fused embedding produced by the dual‐view attention module (Fuse_emb).

Each embedding is projected into two dimensions using UMAP (as implemented in SCANPY), with the same parameter settings across datasets to ensure comparability. For the dual‐view attention module, we record the learned attention weights assigned to the spatial and feature views, denoted as α_coord_and α_feat_, respectively, with α_coord_ +  α_feat_ =  1 for each spot. These weights are visualized both in the UMAP space and mapped back onto the spatial coordinates of the tissue sections. The resulting panels summarizing SAG_emb, FAG_emb, Fuse_emb and the corresponding attention‐weight distributions are presented in Figure S54.

### 4.13 Usage of Comparative Methods

To comprehensively evaluate the clustering performance of SAGE in spatial domain identification, we conduct a systematic benchmark comparison with several representative spatial transcriptomics methods, including stLearn (https://github.com/BiomedicalMachineLearning/stLearn), SEDR (https://github.com/JinmiaoChenLab/SEDR/), STAGATE (https://github.com/zhanglabtools/STAGATE), STAIG (https://github.com/y‐itao/STAIG), GraphST (https://github.com/JinmiaoChenLab/GraphST), PROST (https://github.com/Tang‐Lab‐super/PROST), SCAN‐IT (https://github.com/zcang/SCAN‐IT), and SpaGCN (https://github.com/jianhuupenn/SpaGCN). In all comparative experiments, to ensure the comparability of clustering results, we set the target number of clusters for each method to match the cluster number in manual annotations and strictly follow the recommended configurations and default preprocessing pipelines in each method's official code repository. Additionally, to ensure the reproducibility of the experiments, we set a fixed random seed across all experiments.

### 4.14 Performance Evaluation Metrics

During benchmarking, we evaluate clustering performance across eight complementary metrics, each reflecting different aspects of segmentation quality: NMI, HOM, and COM assess consistency with manual annotations; CHAOS, PAS, and ASW measure spatial coherence and structural compactness; while Moran's *I* and Geary's C evaluate the biological relevance of clustering based on marker gene spatial expression.

To facilitate cross‐dataset and cross‐metric comparison, we refer to the unified index designed by Yuan et al. [36]. Specifically, for each metric $\ell \in \{1,\text{…},N_{\mathrm{metric}}\}$, a score matrix $V^{\left(\ell \right)}\in R^{N_{\mathrm{dataset}}\times N_{\mathrm{method}}}$ is constructed, where each element $V_{ij}^{\left(\ell \right)}$ records the performance score of method *j* on dataset *i* under metric ℓ. *N*_dataset_ and *N*_method_ denote the number of datasets and methods, respectively. Each row of $V^{\left(\ell \right)}$ is sorted to produce a rank matrix $R^{\left(\ell \right)}\in {\left\{1,\text{…},N_{\mathrm{method}}\right\}}^{N_{\mathrm{dataset}}\times N_{\mathrm{method}}}$, where smaller values indicate better ranks. Ranks are normalized to the range [0,1] as follows:

(26) $${\tilde{R}}_{ij}^{\left(\ell \right)}=\frac{N_{\mathrm{method}}-R_{ij}^{\left(\ell \right)}}{N_{\mathrm{method}}-1}$$

The rank score (Figure 2c) for each method *j* under metric ℓ is then computed as the average normalized score across datasets:

(27) $${\mathrm{rank}}_{\left(m\right)}^{j}=\frac{1}{N_{\mathrm{dataset}}}\;\sum_{i=1}^{N_{\mathrm{dataset}}}{\tilde{R}}_{ij}^{\left(\ell \right)}$$

Based on these scores, we define three aggregated overall metrics to summarize method performance:

(28) $$\mathrm{Overal}l_{\mathrm{accuracy}}=\frac{1}{3}\;\left(\mathrm{ran}k_{\mathrm{NMI}}+\mathrm{ran}k_{\mathrm{HOM}}+\mathrm{ran}k_{\mathrm{COM}}\;\right)$$

(29) $$\mathrm{Overal}l_{\mathrm{continuity}}=\frac{1}{3}\;\left(\mathrm{ran}k_{\mathrm{CHAOS}}+\mathrm{ran}k_{\mathrm{PAS}}+\mathrm{ran}k_{\mathrm{ASW}}\right)$$

(30) $$\mathrm{Overal}l_{\mathrm{marker}}=\frac{1}{2}\;\left(\mathrm{ran}k_{{\mathrm{Moran}}^{\prime }s\;I}+\mathrm{ran}k_{{\mathrm{Geary}}^{\prime }s\;C}\right)$$

Additionally, Adjusted Rand Index (ARI) is introduced in tissue‐specific analyses to further assess clustering consistency across slices. To quantify the importance of each gene within its associated topic, we adopt the z‐score computation as previously described in “Construction of high spatial‐specificity genes (HSGs)” section based on the gene–topic weight matrix $H_{T}$. A higher z‐score indicates that a gene is more specifically and strongly associated with the given topic, thus contributing more significantly to the biological interpretation of that topic.

### 4.15 Identification of Differential Genes

Differentially expressed genes (DEGs) are identified using the *rank_genes_groups* function from the SCANPY Python package (version 1.10.2), which implements a two‐sided Wilcoxon rank‐sum test. For most analyses in this study, we apply an FDR cutoff of 0.001 to control for multiple hypothesis testing using the Benjamini–Hochberg procedure, and we require an absolute log_2_ fold‐change of at least 1 (|log_2_FC| ≥ 1) between groups. Fold‐change values are calculated from the group‐wise mean expression of each gene. For the zebrafish melanoma tumor–muscle interface, we adopt a slightly relaxed threshold of FDR < 0.005 for Domains 10 and 12, because an FDR < 0.001 would yield too few DEGs for stable and interpretable enrichment analyses, while still providing stringent control of false discoveries. A gene is considered cluster‐specific if it is found to be differentially up‐regulated in a particular cluster compared to each of the remaining clusters.

In addition, SAGE implements an auxiliary strategy for domain‐specific marker gene discovery based on domain–topic correspondence. This approach exploits the correlation structure between spatial domains and latent topics to identify genes that spatially co‐vary with domain‐level transcriptional programs. The method consists of three steps: (1) Domain‐to‐topic mapping: for each spatial domain, Pearson correlation coefficients are computed between the domain assignment vector and all latent topic activity profiles. The topic with the highest correlation is selected as the representative topic for that domain. (2) Topic‐to‐gene correlation: for the selected topic, Pearson correlations are calculated between its spatial activity and the expression levels of all genes across locations. (3) Marker gene selection: genes with a correlation coefficient exceeding 0.3 are retained and ranked in descending order. The resulting genes constitute a candidate marker set for the domain. The threshold of *r* > 0.3 reflects a moderate effect size commonly used in co‐expression and spatial transcriptomics analyses, while ensuring biological relevance. This correlation‐based framework offers a biologically grounded alternative for marker gene inference, particularly in scenarios where conventional DE testing may be limited by sample heterogeneity or low statistical power. By integrating topic modeling with correlation analysis, it provides an interpretable and data‐driven means to explore the transcriptional architecture of spatial domains.

### 4.16 Gene Ontology Functional Enrichment Analysis

GO functional enrichment analysis was performed using the *enrichr* function from the gseapy Python package (version 1.1.4). The gene sets used included ‘MSigDB_Hallmark_2020’, ‘KEGG_2021_Human’, and ‘GO_Biological_Process_2018’.

### 4.17 Statistics and Reproducibility

All statistical tests and multiple‐testing corrections follow the procedures described in the corresponding Methods subsections. In particular, differentially expressed genes are defined as described in “Identification of differential genes,” with FDR‐controlled Wilcoxon rank‐sum tests and consistent fold‐change thresholds across analyses.

No statistical method is used to pre‐determine sample size. No data is excluded from the analysis, all genes in datasets are used throughout all analyses. The investigators are blinded to allocation during experiments and outcome assessment.

## Author Contributions

Y.H. and S.W. conceived and designed the project. Y.H., Y.X., and L.D. contributed to the overall design and implementation of the study. Y.H. collected the datasets and conducted the experiments. Y.H. performed the data analysis. Y.H. tested the software and validated the results. Y.H. and S.W. drafted the manuscript. H.‐D.L. and S.W. provided critical revisions and supervision. Y.L. contributed methodological guidance. All authors reviewed and approved the final version of the manuscript.

## Funding

This work was supported in part by the National Natural Science Foundation of China under Grants (Nos. 62350004, 62332020), and Hunan Provincial Natural Science Foundation of China (2025JJ20068). This work was carried out in part using computing resources at the High‐Performance Computing Center of Central South University.

## Conflicts of Interest

The authors declare no conflicts of interest.

## Supporting information




**Supporting File**: advs73676‐sup‐0001‐SuppMat.pdf.

## Data Availability

Data sharing is not applicable to this article as no new data were created or analyzed in this study.
