# Supplementary material for: SAGE: Spatially Aware Gene Selection and Dual‐View Embedding Fusion for Domain Identification in Spatial Transcriptomics
Source: Adv Sci (Weinh). 2026 Jan 4;13(16):e20333. doi: 10.1002/advs.202520333 (PMC13042484; doi:10.1002/advs.202520333)
Supplement: Supplementary file 1 — Supporting File: advs73676‐sup‐0001‐SuppMat.pdf. [file ADVS-13-e20333-s001.pdf]

Supplementary Information for

SAGE: Spatially aware Genes Selection and Dual-view Embedding Fusion for Domain Identification in Spatial Transcriptomics

He *et al.*

Supplementary Figures 1-54

Supplementary Figure 1

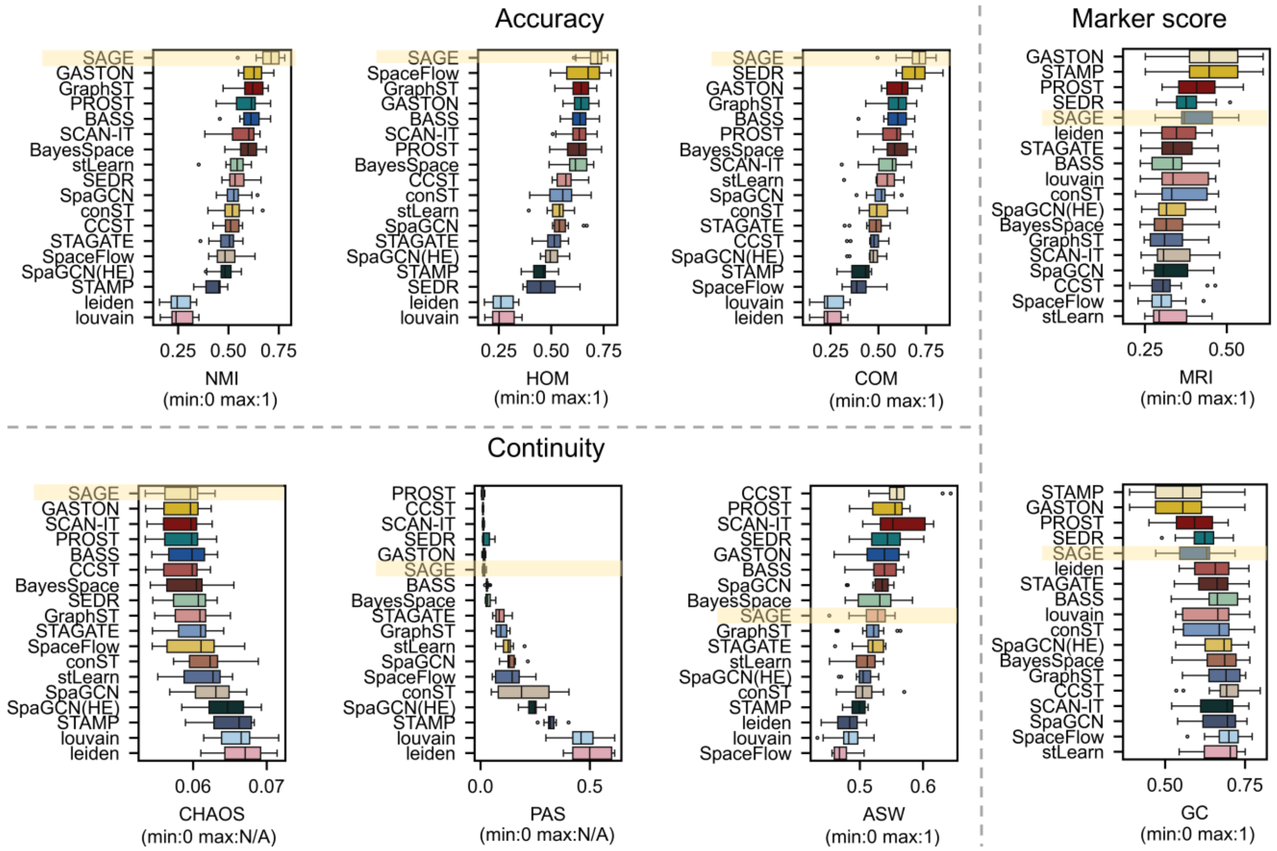

Supplementary Fig. 1 Comparison of SAGE with 14 state-of-the-art methods on the 10× Visium-based DLPFC dataset (n=12). Performance comparisons between SAGE and 14 state-of-the-art methods are presented based on three categories of evaluation metrics: accuracy (NMI, HOM, COM), continuity (CHAOS, PAS, ASW), and marker score (Moran's I, Geary's C). Each method is ranked in descending order for each metric, with SAGE highlighted in light yellow. Box plots indicate the interquartile range (IQR; Q1 to Q3), with the median shown as a central line and whiskers extending to 1.5× IQR. Source data are available in the Source Data file.

Supplementary Figure 2

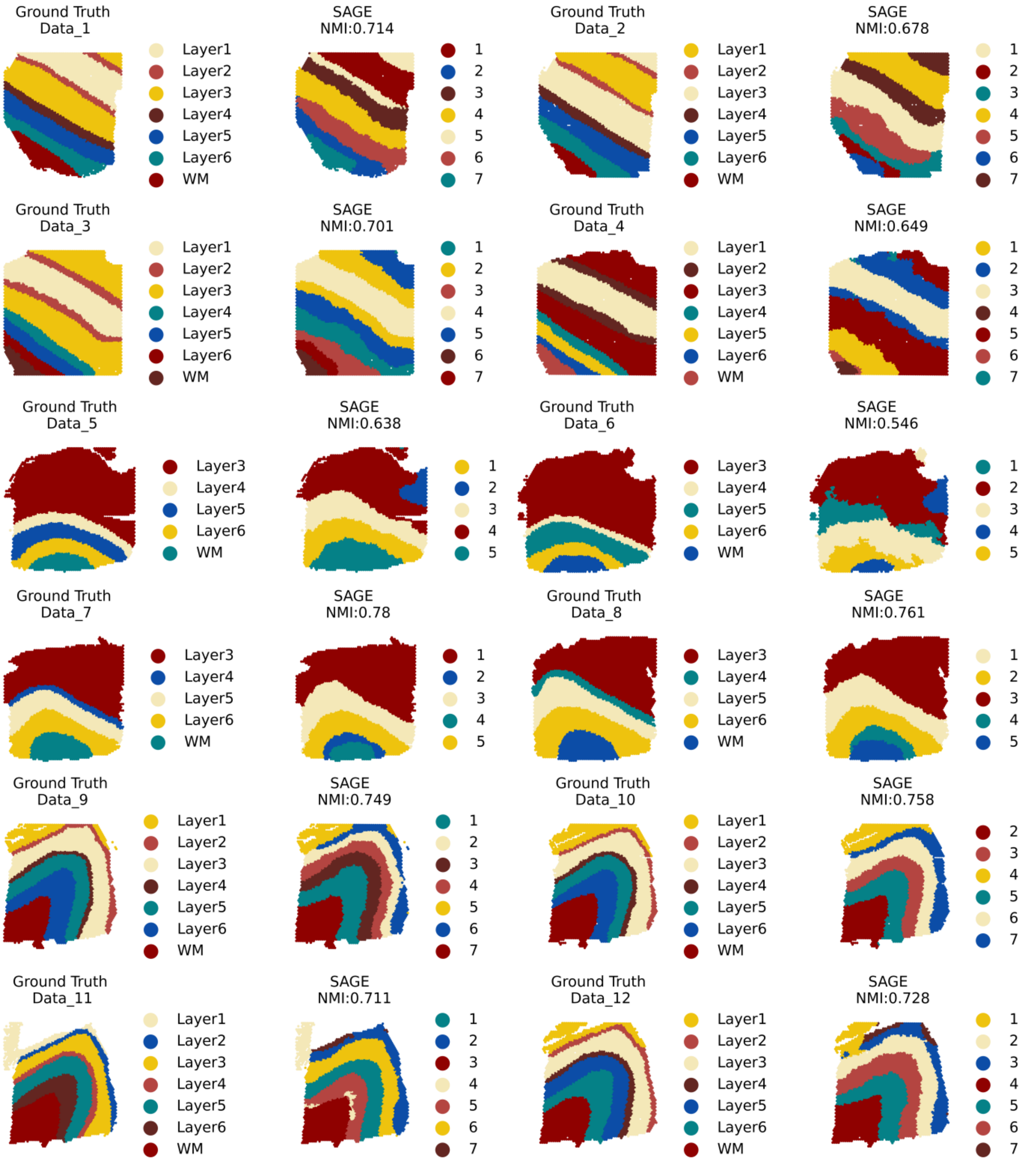

Supplementary Fig. 2 Spatial domain segmentation on the 10× Visium-based DLPFC dataset (n=12). Spatial domain segmentation results are shown for all slices of the DLPFC dataset based on 10× Visium technology, using both ground truth annotations and SAGE. Each slice is annotated with the corresponding normalized mutual information (NMI) value to assess segmentation accuracy.

Supplementary Figure 3

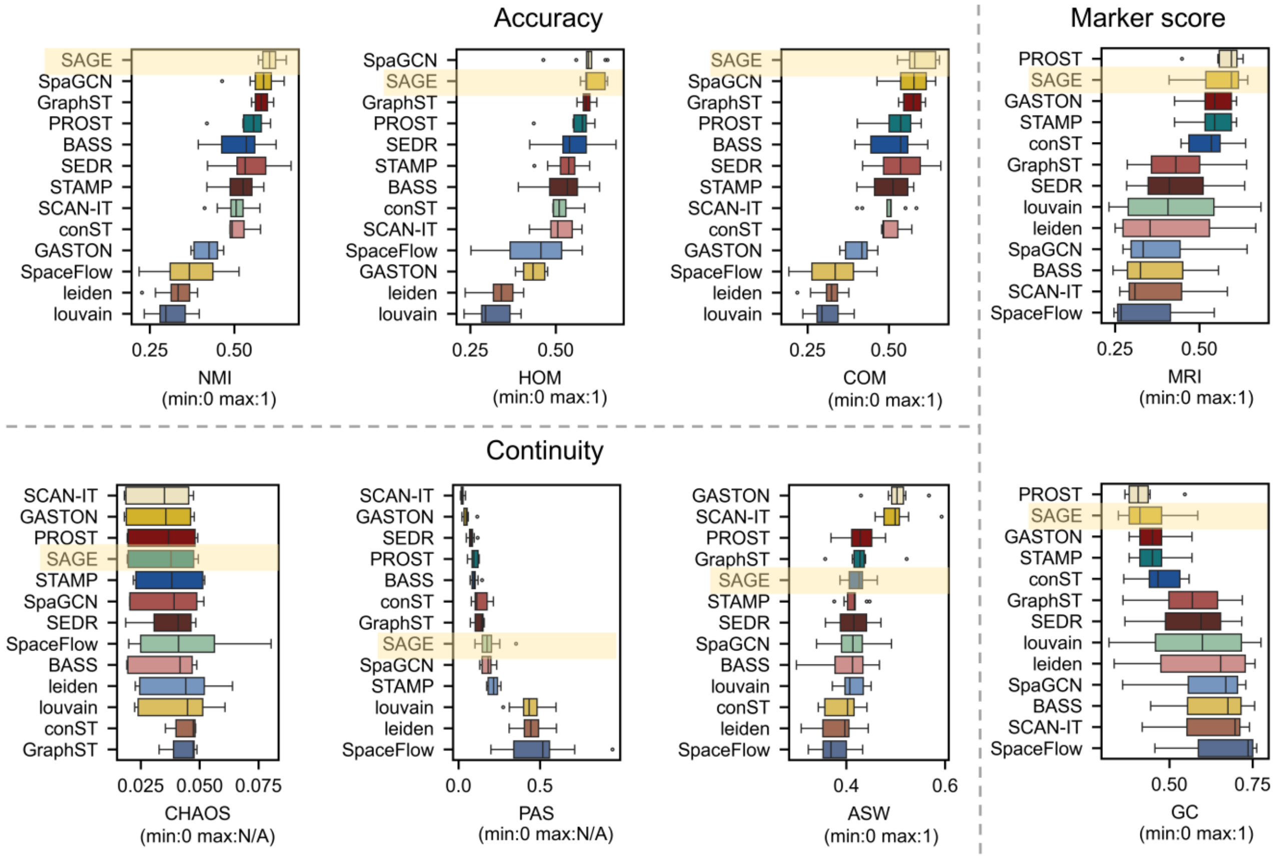

Supplementary Fig. 3 Comparison of SAGE with 11 state-of-the-art methods on the Stereo-seq-based mouse embryo dataset (n=9). Performance comparison of SAGE and 12 state-of-the-art methods across multiple evaluation metrics: accuracy (NMI, HOM, COM), continuity (CHAOS, PAS, ASW), and marker score (Moran's I, Geary's C). For each metric, methods are ranked in descending order, with SAGE highlighted in light yellow. Box plots represent interquartile range (IQR; Q1 to Q3), with the median indicated as a central line and whiskers extending to 1.5× IQR. Source data are available in the Source Data file.

Supplementary Figure 4

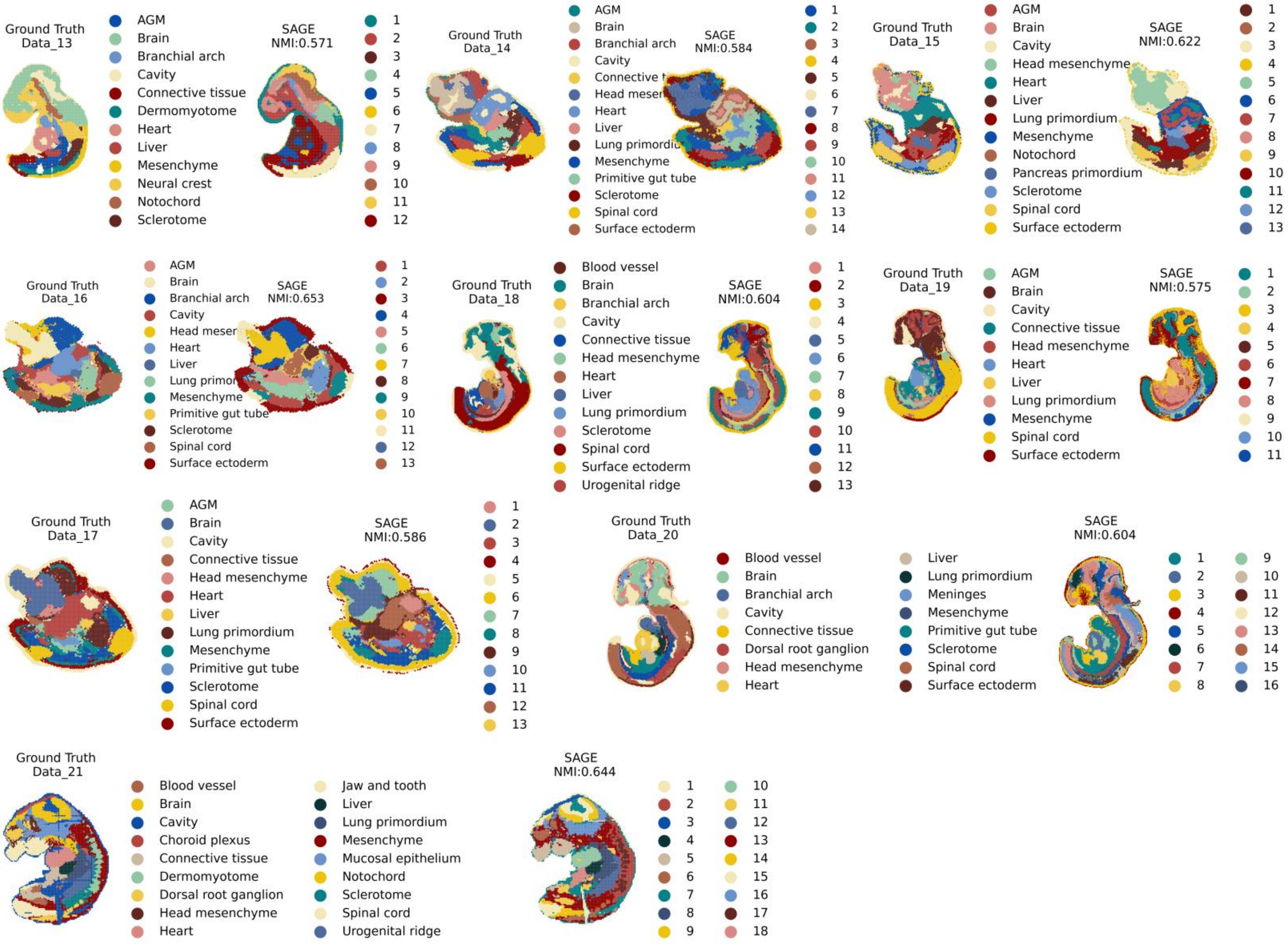

Supplementary Fig. 4 Spatial domain segmentation on the Stereo-seq-based mouse embryo dataset (n=9). Spatial domain segmentation results are shown for all slices of the Stereo-seq-based mouse embryo dataset using ground truth annotations and SAGE. Each slice is accompanied by the corresponding normalized mutual information (NMI) value, providing a quantitative measure of segmentation accuracy.

Supplementary Figure 5

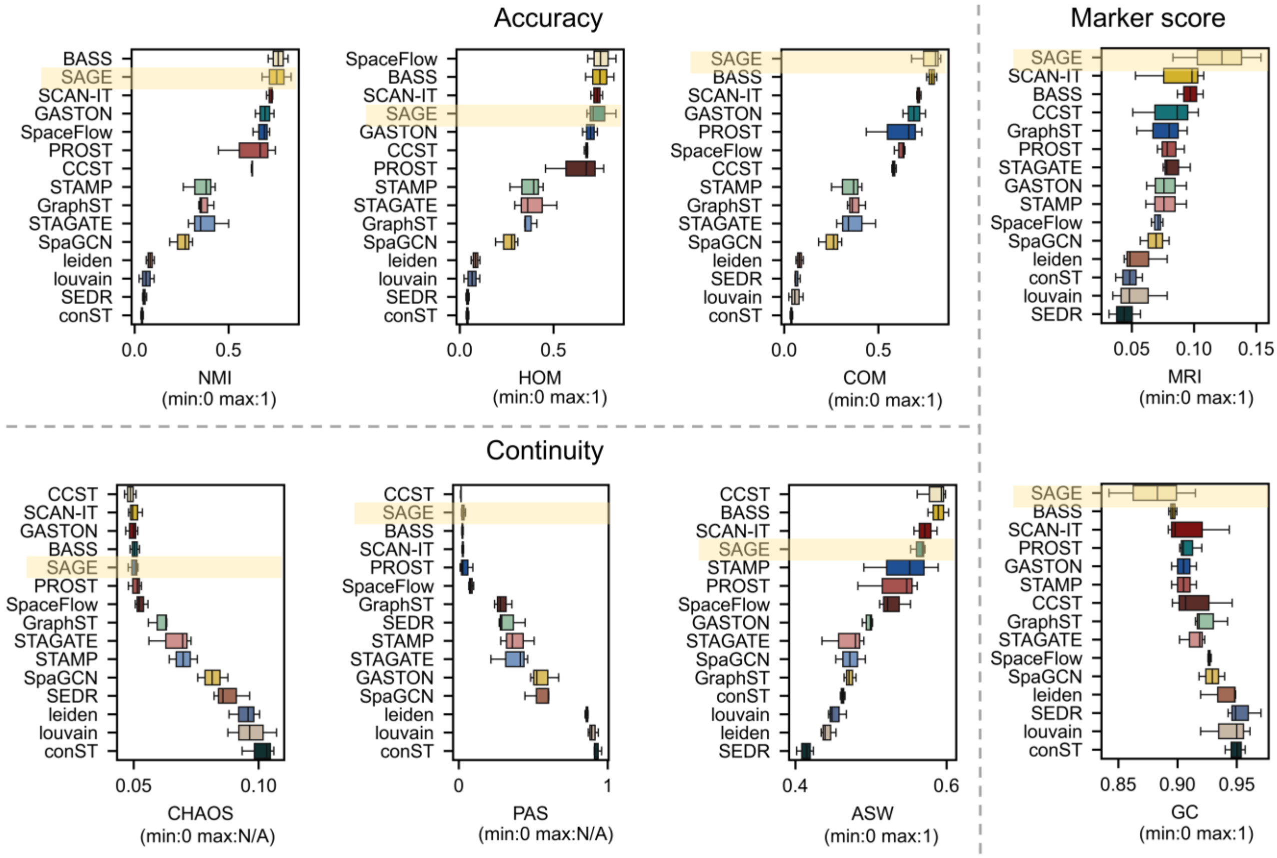

Supplementary Fig. 5 Comparison of SAGE with 12 state-of-the-art methods on the BaristaSeq-based mouse primary cortex dataset (n=3). Performance comparisons between SAGE and 12 state-of-the-art methods are presented across accuracy (NMI, HOM, COM), continuity (CHAOS, PAS, ASW), and marker score (Moran's I, Geary's C), based on quantitative analysis of 3 tissue slices. For each metric, methods are ranked in descending order of performance, with SAGE highlighted in light yellow. Box plots display the interquartile range (IQR; Q1 to Q3), with the median shown as a central line and whiskers extending to 1.5× IQR. Source data are available in the Source Data file.

Supplementary Figure 6

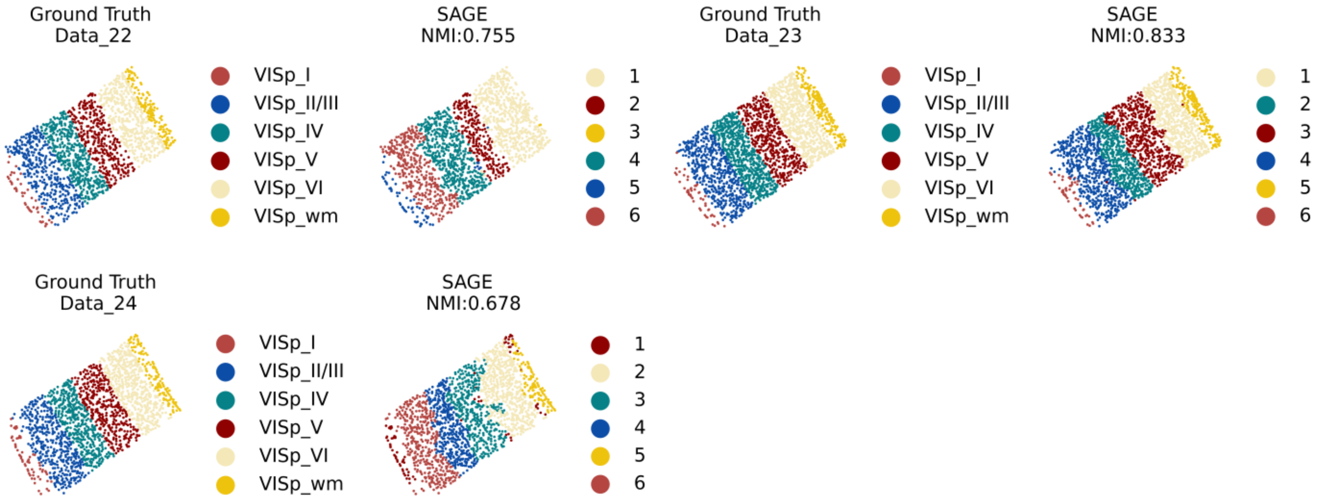

Supplementary Fig. 6 Spatial domain segmentation on the BaristaSeq-based mouse primary cortex dataset (n=3). Spatial domain segmentation results are shown for all slices of the mouse primary cortex dataset based on BaristaSeq technology, using both ground truth annotations and SAGE. Each slice is labeled with the corresponding normalized mutual information (NMI) value to indicate segmentation accuracy.

Supplementary Figure 7

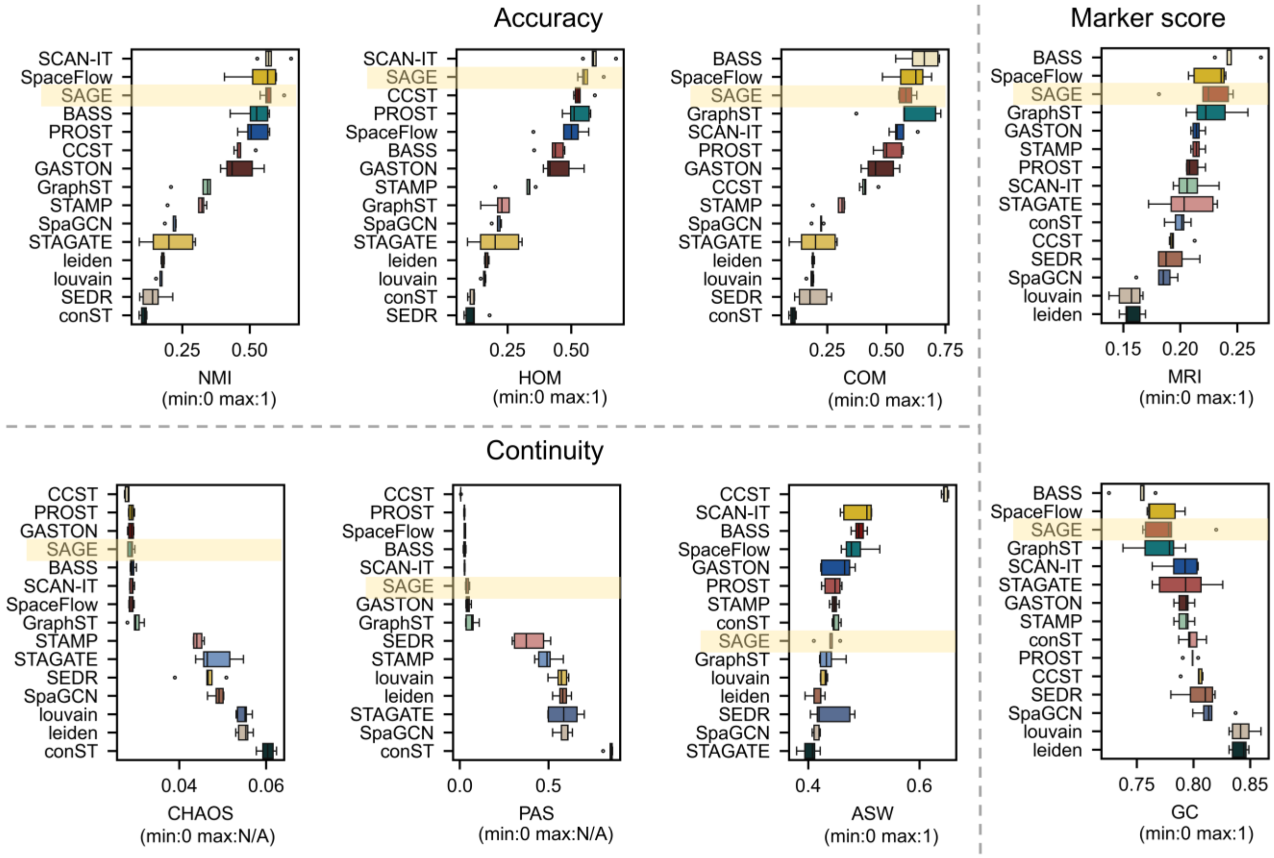

Supplementary Fig. 7 Comparison of SAGE with 12 state-of-the-art methods on the MERFISH-based hypothalamic preoptic region dataset (n=5). Performance comparisons between SAGE and 12 state-of-the-art methods are provided across three categories of evaluation metrics: accuracy (NMI, HOM, COM), continuity (CHAOS, PAS, ASW), and marker score (Moran's I, Geary's C), based on comprehensive analysis of 5 tissue slices. Each method is ranked in descending order of performance per metric, with SAGE highlighted in light yellow. Box plots indicate the interquartile range (IQR; Q1 to Q3), with the median shown as a central line and whiskers extending to 1.5× IQR. Source data are available in the Source Data file.

Supplementary Figure 8

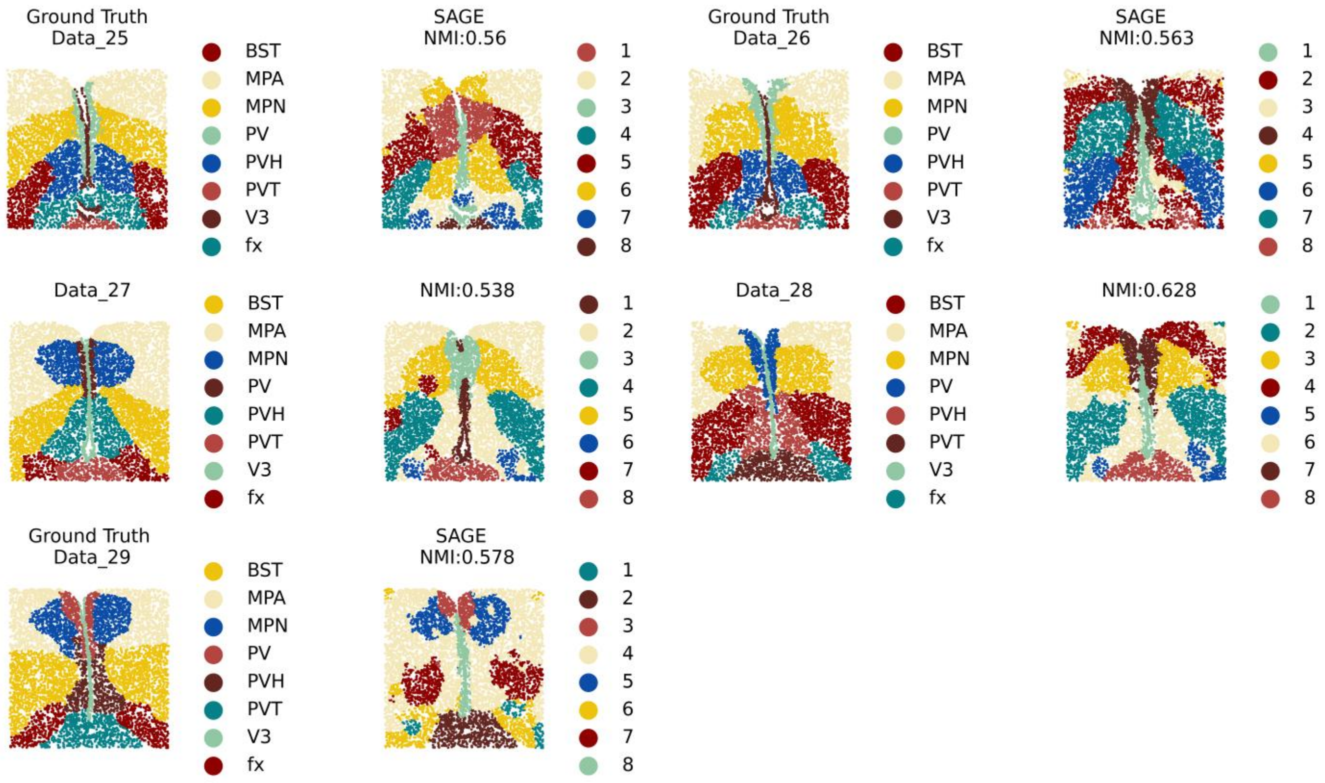

Supplementary Fig. 8 Spatial domain segmentation on the MERFISH-based hypothalamic preoptic region dataset (n=5). Spatial domain segmentation results are shown for all slices of the hypothalamic preoptic region dataset based on MERFISH technology, using both ground truth annotations and SAGE. Each slice is annotated with the corresponding normalized mutual information (NMI) value to indicate segmentation performance.

Supplementary Figure 9

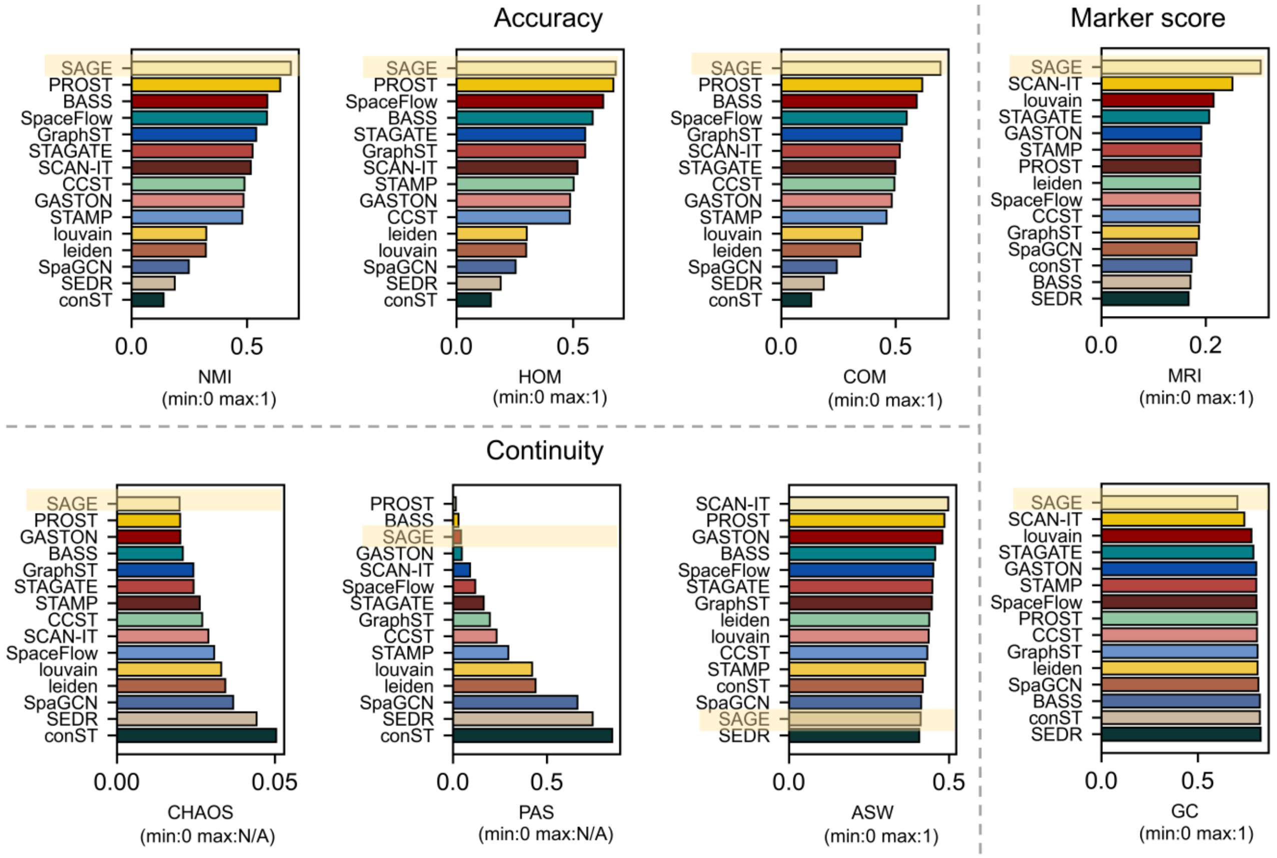

Supplementary Fig. 9 Comparison of SAGE with 12 state-of-the-art methods on the osmFISH-based mouse somatosensory cortex dataset (n=1). Performance comparisons between SAGE and 12 state-of-the-art methods are presented across accuracy (NMI, HOM, COM), continuity (CHAOS, PAS, ASW), and marker score (Moran's *I*, Geary's C), based on comprehensive analysis of the mouse somatosensory cortex slice. For each metric, methods are ranked in descending order, with SAGE highlighted in light yellow. Horizontal bar plots visualize the score distribution of each method across the different metrics. Source data are available in the Source Data file.

Supplementary Figure 10

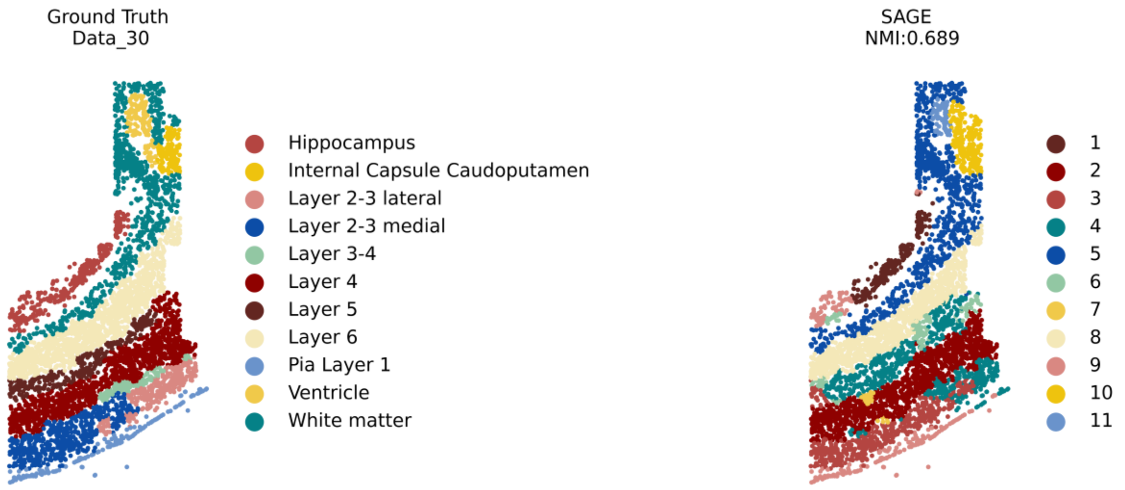

Supplementary Fig. 10 Spatial domain segmentation on the osmFISH-based mouse somatosensory cortex dataset (n=1). Spatial domain segmentation results are shown for the mouse somatosensory cortex slice based on osmFISH technology, using both ground truth annotations and SAGE. The corresponding normalized mutual information (NMI) value is displayed to evaluate segmentation accuracy.

Supplementary Figure 11

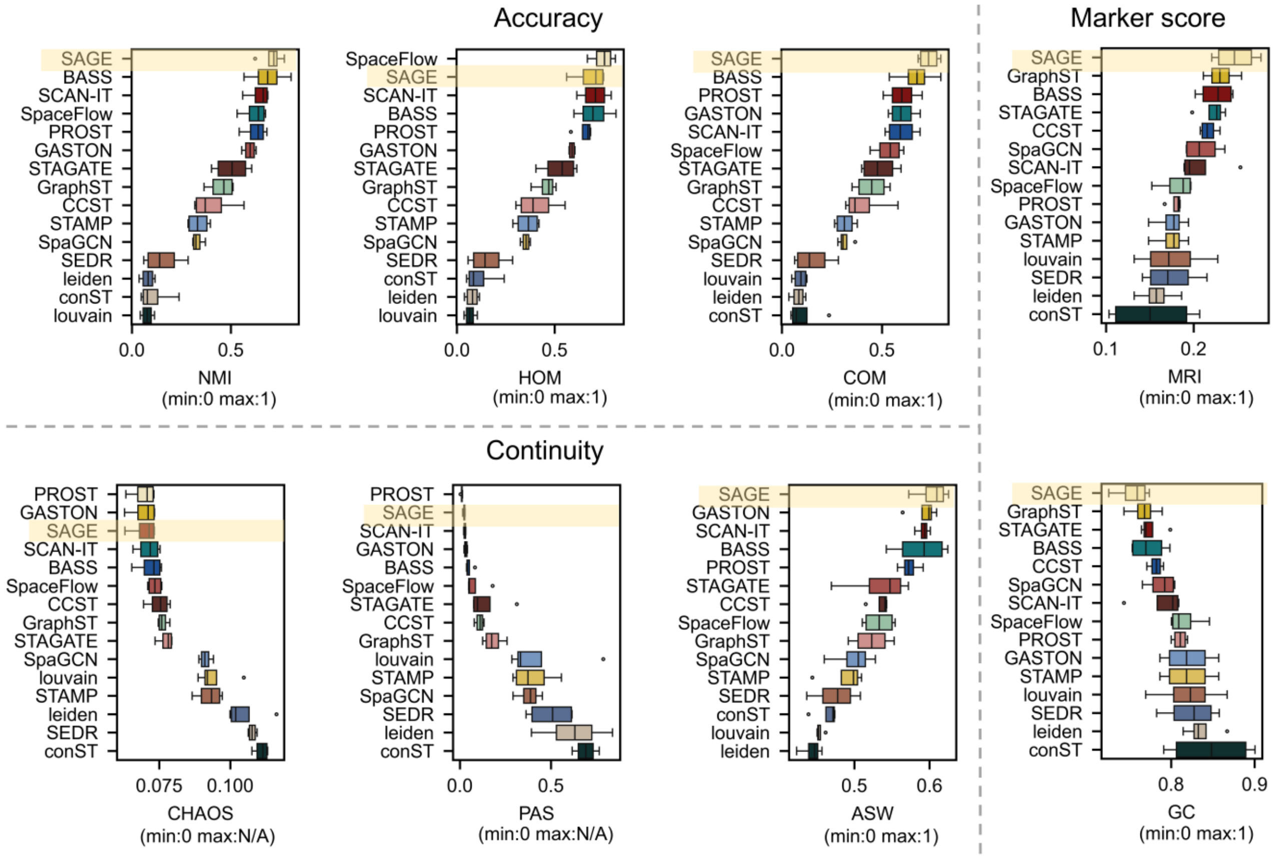

Supplementary Fig. 11 Comparison of SAGE with 12 state-of-the-art methods on STARmap and STARmap datasets (n = 4). Comprehensive performance comparisons between SAGE and 12 state-of-the-art methods are presented across accuracy (NMI, HOM, COM), continuity (CHAOS, PAS, ASW), and marker score (Moran's I, Geary's C). Analysis is based on 4 tissue slices from the STARmap* and STARmap-based mouse medial prefrontal cortex and visual cortex datasets. For each metric, methods are ranked in descending order of performance, with SAGE highlighted in light yellow. Box plots indicate the interquartile range (IQR; Q1 to Q3), with the median represented by a central line and whiskers extending to 1.5× IQR. Source data are available in the Source Data file.

Supplementary Figure 12

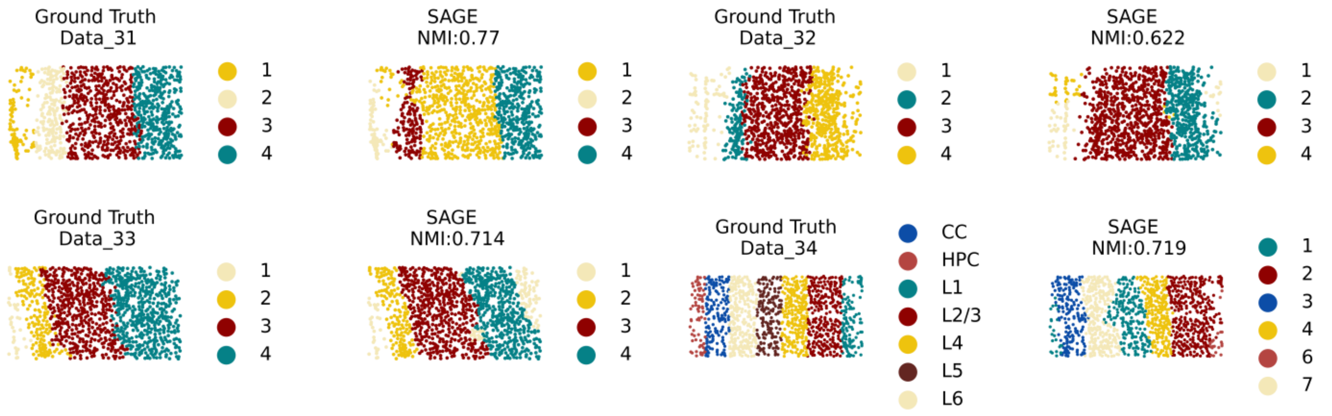

Supplementary Fig. 12 Spatial domain segmentation on the STARmap and STARmap-based mouse medial prefrontal cortex and visual cortex datasets (n = 4). Spatial domain segmentation results from SAGE and ground truth annotations are shown across 4 tissue slices derived from the STARmap* and STARmap-based mouse medial prefrontal cortex (n = 3) and visual cortex (n = 1) datasets. The corresponding normalized mutual information (NMI) values are provided for each slice to indicate segmentation performance.

Supplementary Figure 13

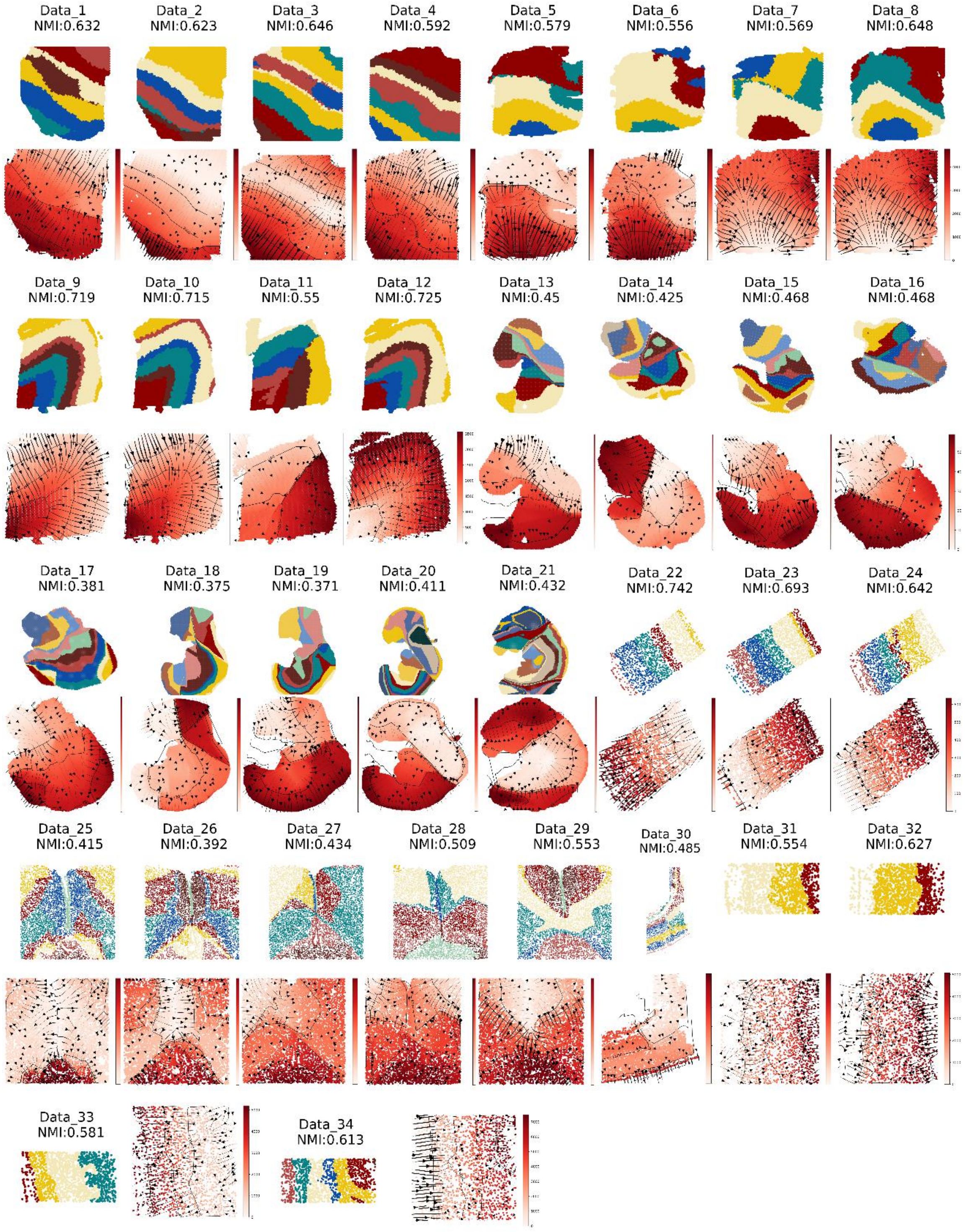

Supplementary Fig. 13 Benchmarking GASTON across 34 spatial transcriptomics datasets. From left to right and top to bottom, each panel corresponds to one dataset. For each dataset, the upper subplot shows the inferred spatial domains, with different colors indicating distinct domains. The title of the upper subplot displays the dataset identifier (Data_1 to Data_34) and the NMI score with respect to the ground-truth annotation. The lower subplot shows the gradient contour map identified by GASTON, where colors range from light to dark red and deeper red denotes higher gradient values.

Supplementary Figure 14

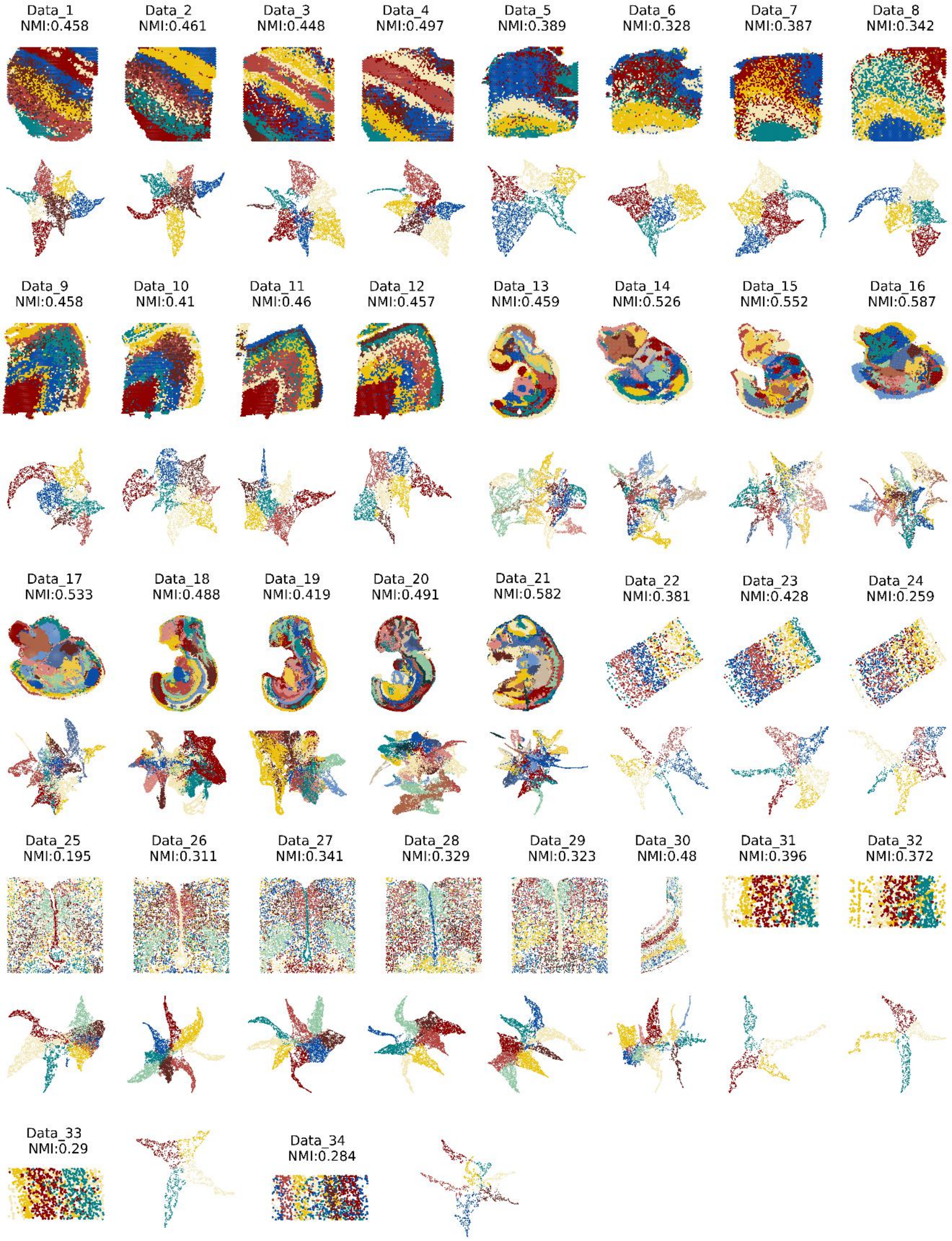

Supplementary Fig. 14 Benchmarking STAMP across 34 spatial transcriptomics datasets. From left to right and top to bottom, each panel corresponds to one dataset. For each dataset, the upper subplot shows the inferred spatial domains, with different colors indicating distinct domains. The title of the upper subplot displays the dataset identifier (Data_1 to Data_34) and the NMI score with respect to the ground-truth annotation (reported to three decimal places). The lower subplot shows the UMAP visualization of the STAMP embedding.

Supplementary Figure 15

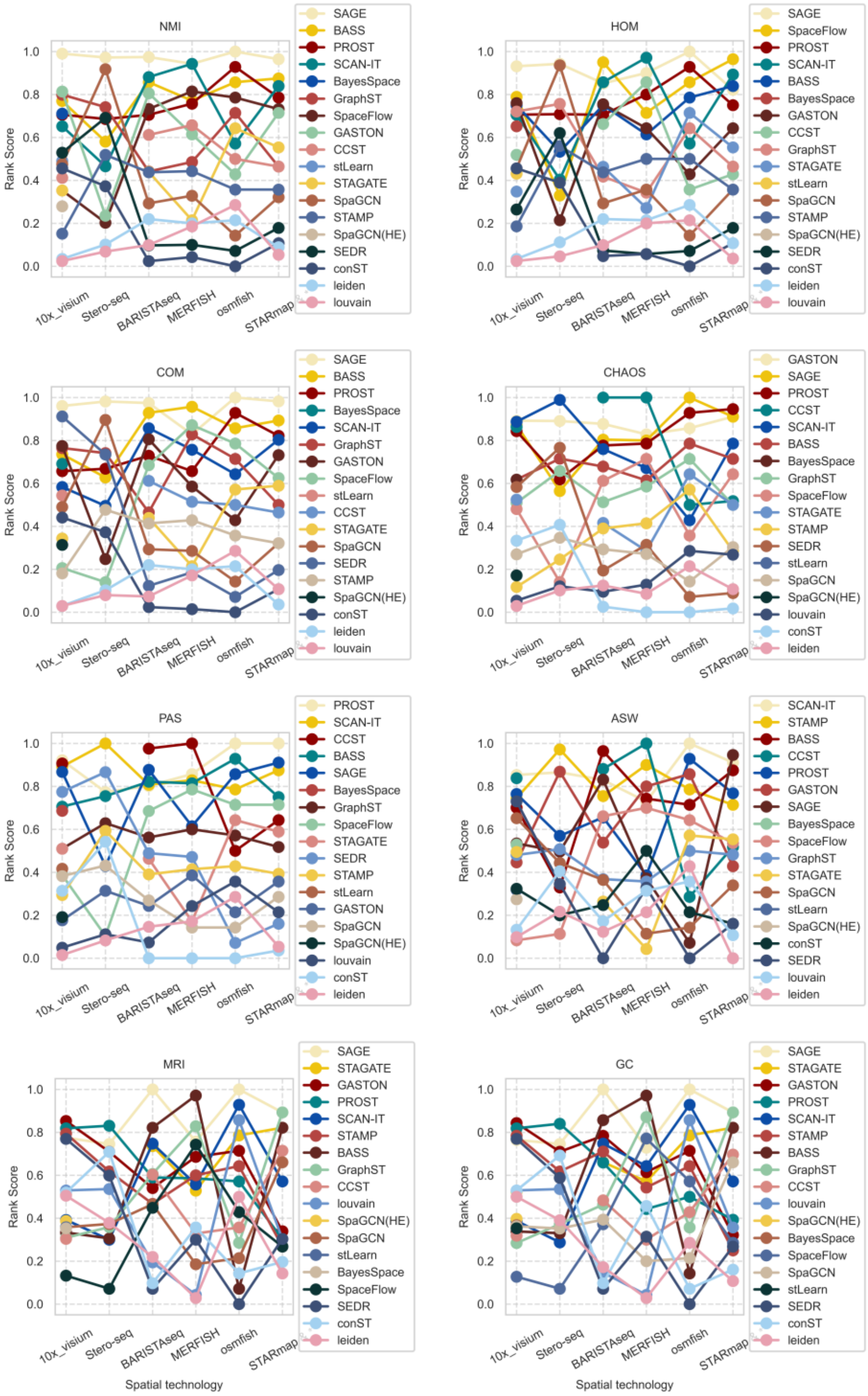

Supplementary Fig. 15 Comparative performance on various spatial transcriptomics technologies. This figure presents a comprehensive comparison of SAGE with 14 state-of-the-art spatial domain segmentation methods (including PROST, SCAN-IT, BayesSpace, GraphST, SpaGCN, and others) across multiple spatial transcriptomics platforms, including 10× Visium, Stereo-seq, BARISTA-seq, MERFISH, and STARmap. Evaluation metrics include accuracy (NMI, HOM, COM), continuity (CHAOS, PAS, ASW), and marker score (Moran's I, Geary's C). In each subplot, methods are ranked by their rank score in descending order. The x-axis denotes different spatial transcriptomics technologies, while the y-axis represents the normalized rank score (ranging from 0 to 1, with higher scores indicating better performance). Source data are provided in the Source Data file.

Supplementary Figure 16

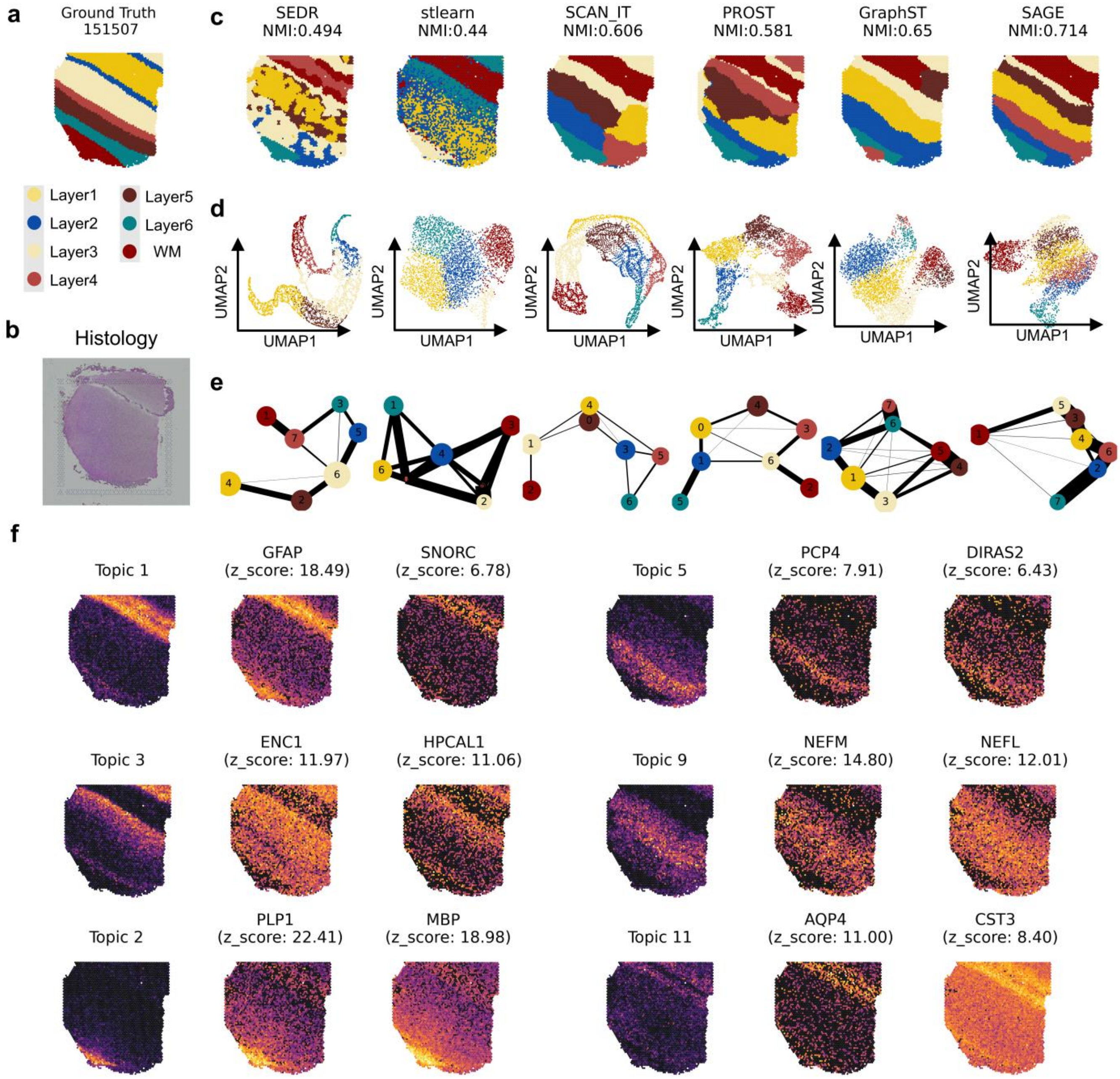

Supplementary Fig. 16 Spatial domain segmentation, UMAP visualization, PAGA graph, and identified gene co-expression topics based on the 10× Visium DLPFC dataset (Slice ID: 151507). **(a)** Manual annotations (provided by Maynard *et al.*). **(b)** H&E-stained tissue image. **(c)** Spatial domain segmentation results generated by SEDR, stLearn, SCAN-IT, PROST, GraphST, and SAGE. **(d, e)** UMAP visualizations and PAGA graphs based on low-dimensional embeddings from SEDR, stLearn, SCAN-IT, PROST, GraphST, and SAGE, with spot colors reflecting manual annotations. **(f)** Gene co-expression topics identified by SAGE exhibiting significant spatial expression patterns, along with their top contributing genes. Statistical significance was assessed using Z-scores.

Supplementary Figure 17

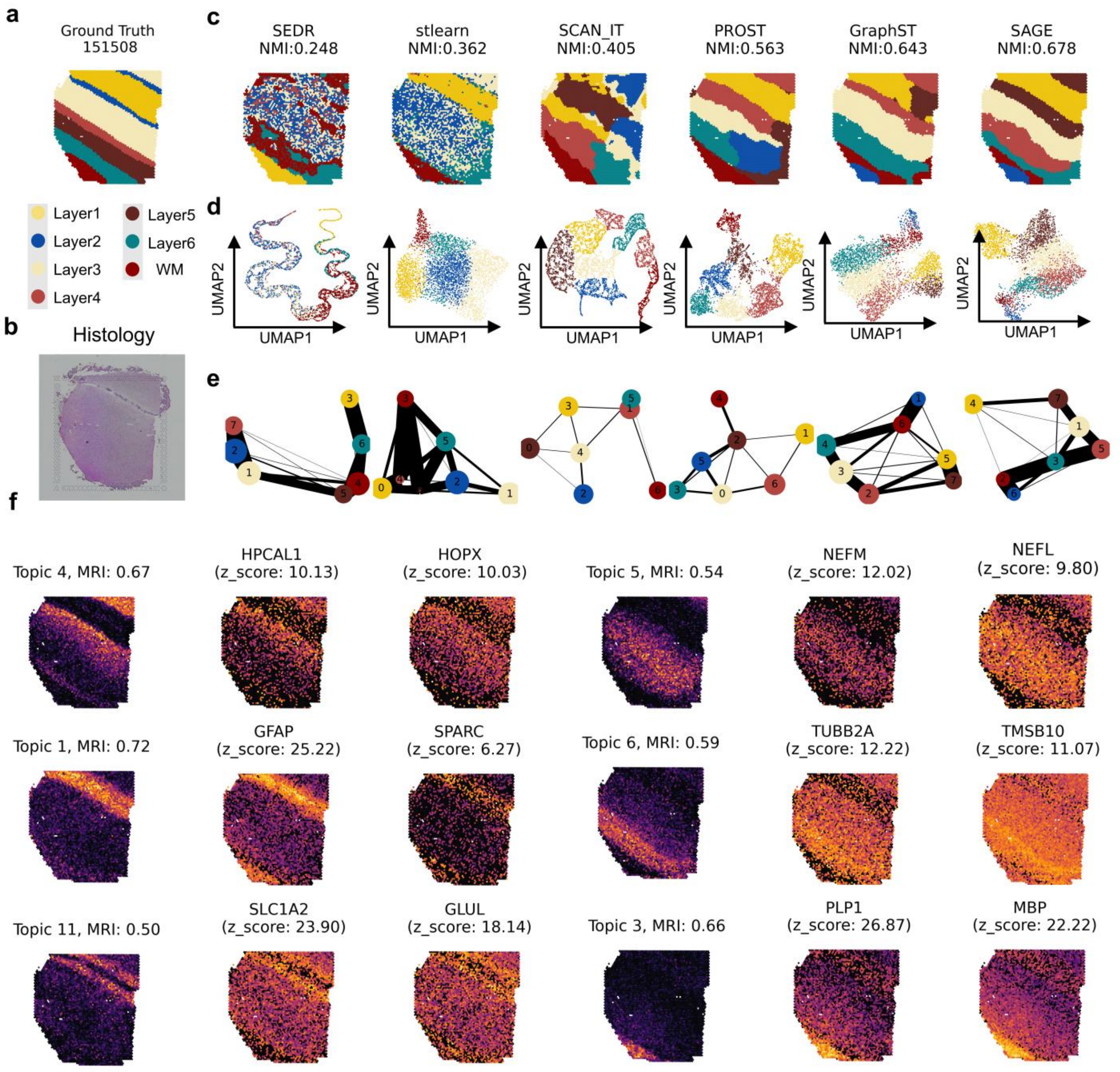

Supplementary Fig. 17 Spatial domain segmentation, UMAP visualization, PAGA graph, and identified gene co-expression topics based on the 10× Visium DLPFC dataset (Slice ID: 151508). **(a)** Manual annotations (provided by Maynard *et al.*). **(b)** H&E-stained tissue image. **(c)** Spatial domain segmentation results generated by SEDR, stLearn, SCAN-IT, PROST, GraphST, and SAGE. **(d, e**) UMAP visualizations and PAGA graphs based on low-dimensional embeddings from SEDR, stLearn, SCAN-IT, PROST, GraphST, and SAGE, with spot colors reflecting manual annotations. **(f)** Gene co-expression topics identified by SAGE exhibiting significant spatial expression patterns, along with their top contributing genes. Statistical significance was assessed using Z-scores.

Supplementary Figure 18

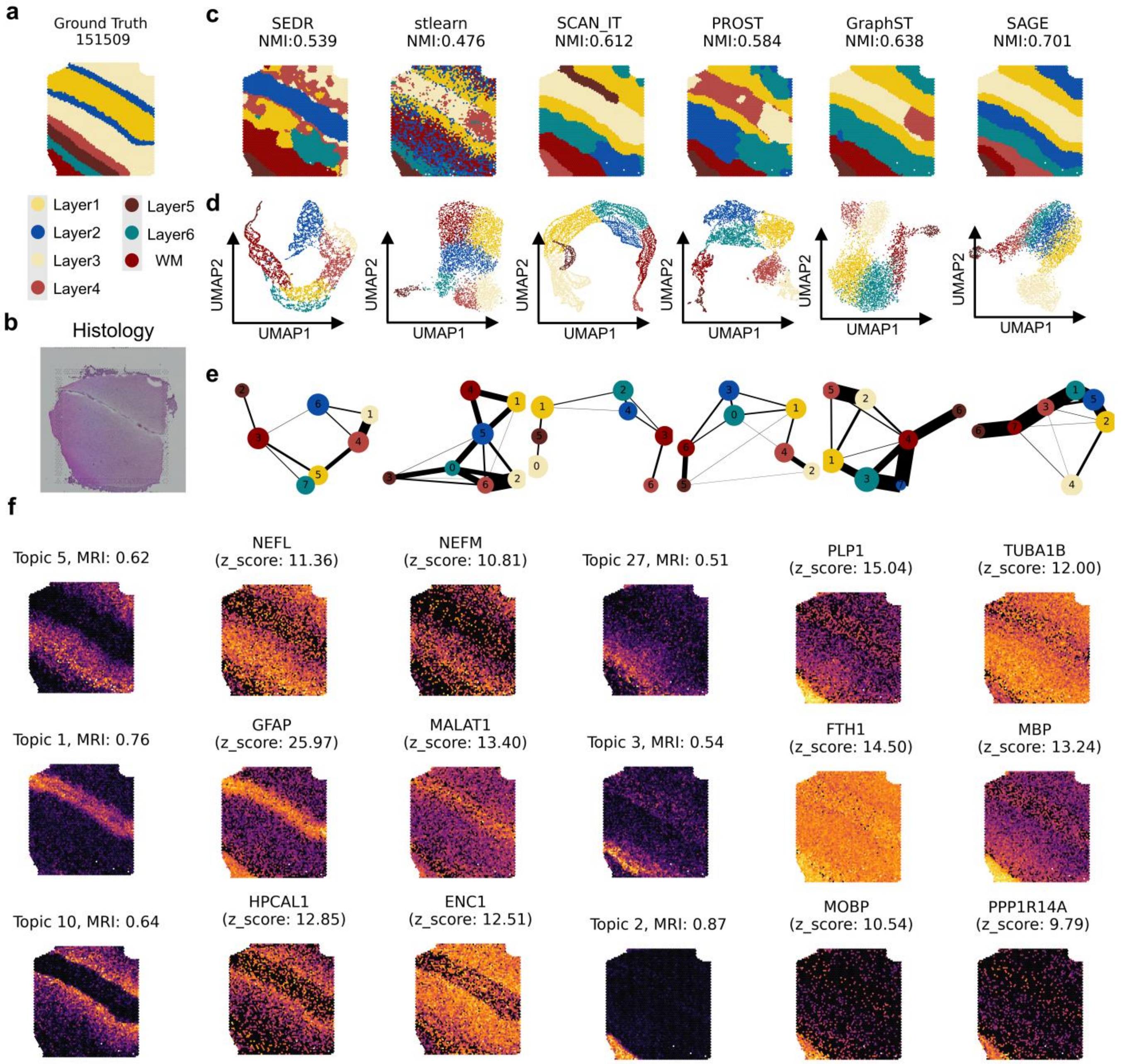

Supplementary Fig. 18 Spatial domain segmentation, UMAP visualization, PAGA graph, and identified gene co-expression topics based on the 10× Visium DLPFC dataset (Slice ID: 151509). **(a)** Manual annotations (provided by Maynard *et al.*). **(b)** H&E-stained tissue image. **(c)** Spatial domain segmentation results generated by SEDR, stLearn, SCAN-IT, PROST, GraphST, and SAGE. **(d, e)** UMAP visualizations and PAGA graphs based on low-dimensional embeddings from SEDR, stLearn, SCAN-IT, PROST, GraphST, and SAGE, with spot colors reflecting manual annotations. **(f)** Gene co-expression topics identified by SAGE exhibiting significant spatial expression patterns, along with their top contributing genes. Statistical significance was assessed using Z-scores.

Supplementary Figure 19

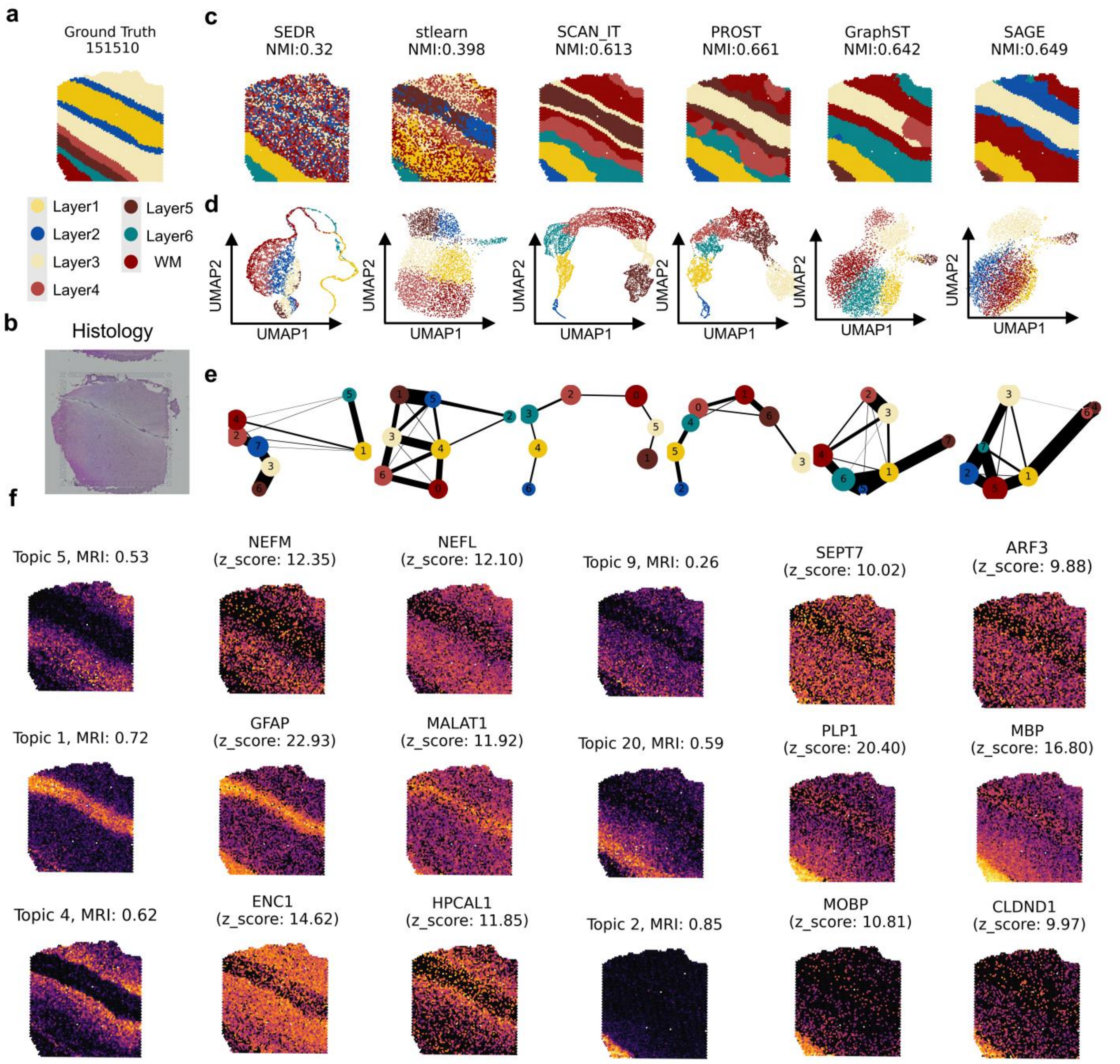

Supplementary Fig. 19 Spatial domain segmentation, UMAP visualization, PAGA graph, and identified gene co-expression topics based on the 10× Visium DLPFC dataset (Slice ID: 151510). **(a)** Manual annotations (provided by Maynard *et al.*). **(b)** H&E-stained tissue image. **(c)** Spatial domain segmentation results generated by SEDR, stLearn, SCAN-IT, PROST, GraphST, and SAGE. **(d, e)** UMAP visualizations and PAGA graphs based on low-dimensional embeddings from SEDR, stLearn, SCAN-IT, PROST, GraphST, and SAGE, with spot colors reflecting manual annotations. **(f)** Gene co-expression topics identified by SAGE exhibiting significant spatial expression patterns, along with their top contributing genes. Statistical significance was assessed using Z-scores.

Supplementary Figure 20

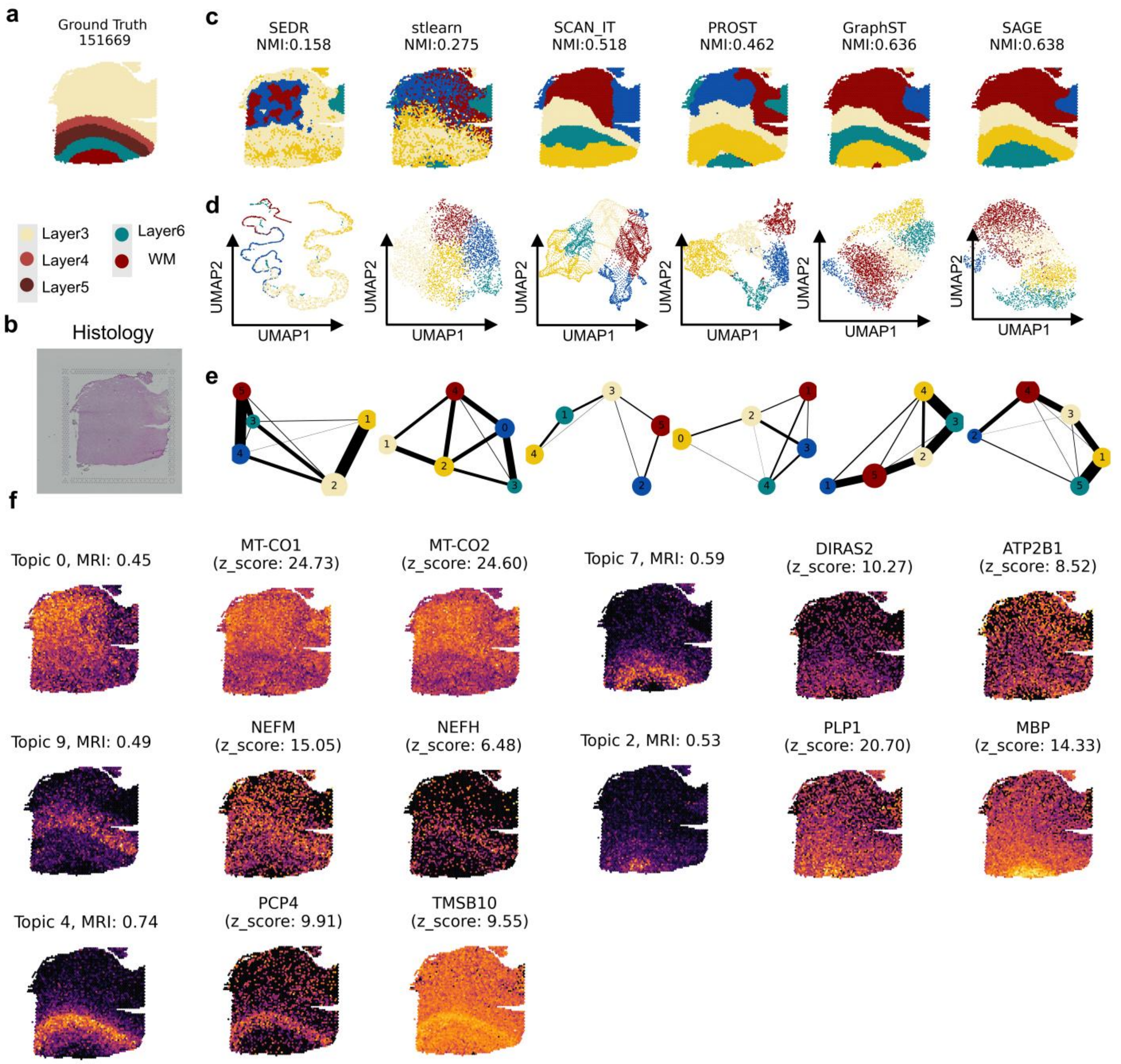

Supplementary Fig. 20 Spatial domain segmentation, UMAP visualization, PAGA graph, and identified gene co-expression topics based on the 10× Visium DLPFC dataset (Slice ID: 151669). **(a)** Manual annotations (provided by Maynard *et al.*). **(b)** H&E-stained tissue image. **(c)** Spatial domain segmentation results generated by SEDR, stLearn, SCAN-IT, PROST, GraphST, and SAGE. **(d, e)** UMAP visualizations and PAGA graphs based on low-dimensional embeddings from SEDR, stLearn, SCAN-IT, PROST, GraphST, and SAGE, with spot colors reflecting manual annotations. **(f)** Gene co-expression topics identified by SAGE exhibiting significant spatial expression patterns, along with their top contributing genes. Statistical significance was assessed using Z-scores.

Supplementary Figure 21

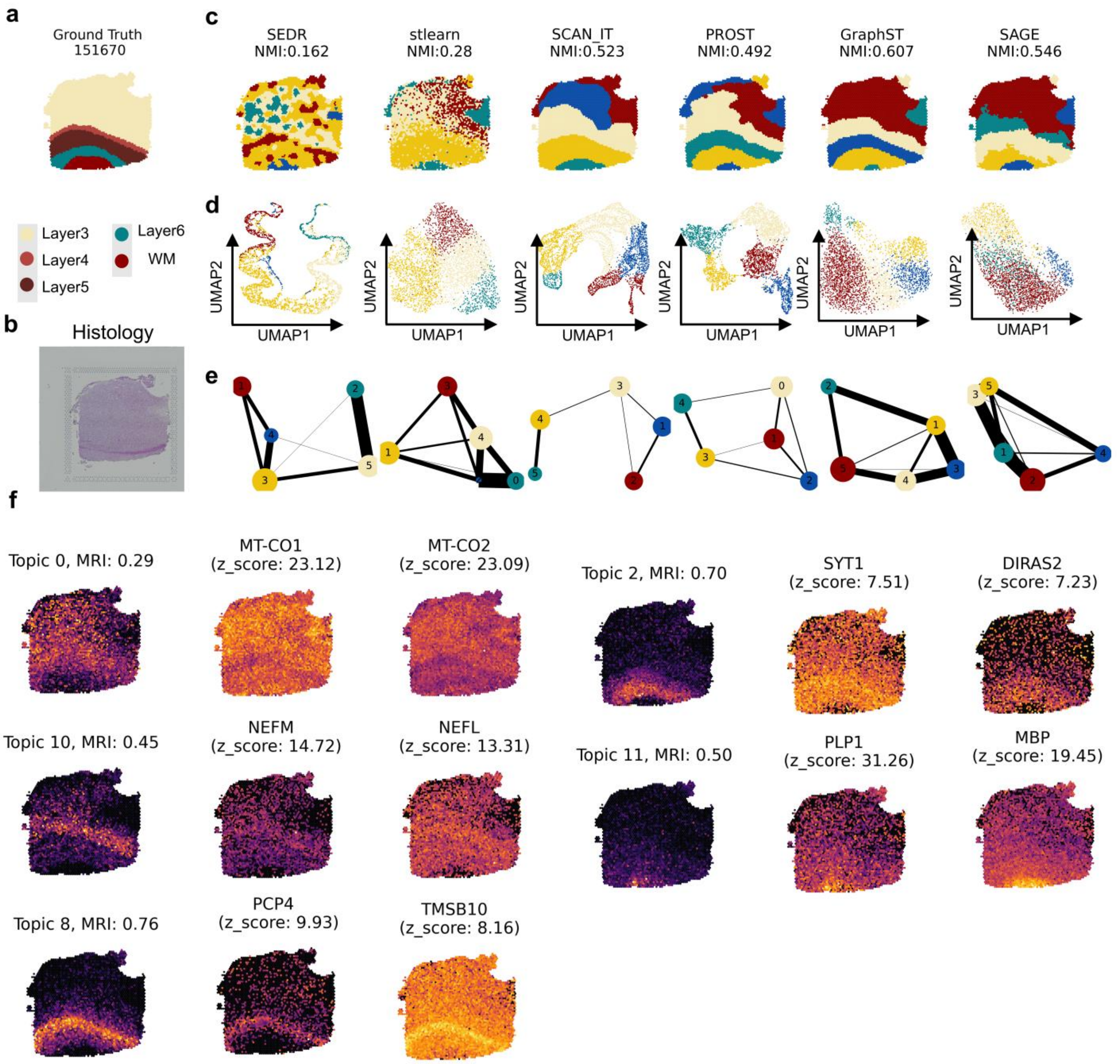

Supplementary Fig. 21 Spatial domain segmentation, UMAP visualization, PAGA graph, and identified gene co-expression topics based on the 10× Visium DLPFC dataset (Slice ID: 151670). **(a)** Manual annotations (provided by Maynard *et al.*). **(b)** H&E-stained tissue image. **(c)** Spatial domain segmentation results generated by SEDR, stLearn, SCAN-IT, PROST, GraphST, and SAGE. **(d, e)** UMAP visualizations and PAGA graphs based on low-dimensional embeddings from SEDR, stLearn, SCAN-IT, PROST, GraphST, and SAGE, with spot colors reflecting manual annotations. **(f)** Gene co-expression topics identified by SAGE exhibiting significant spatial expression patterns, along with their top contributing genes. Statistical significance was assessed using Z-scores.

Supplementary Figure 22

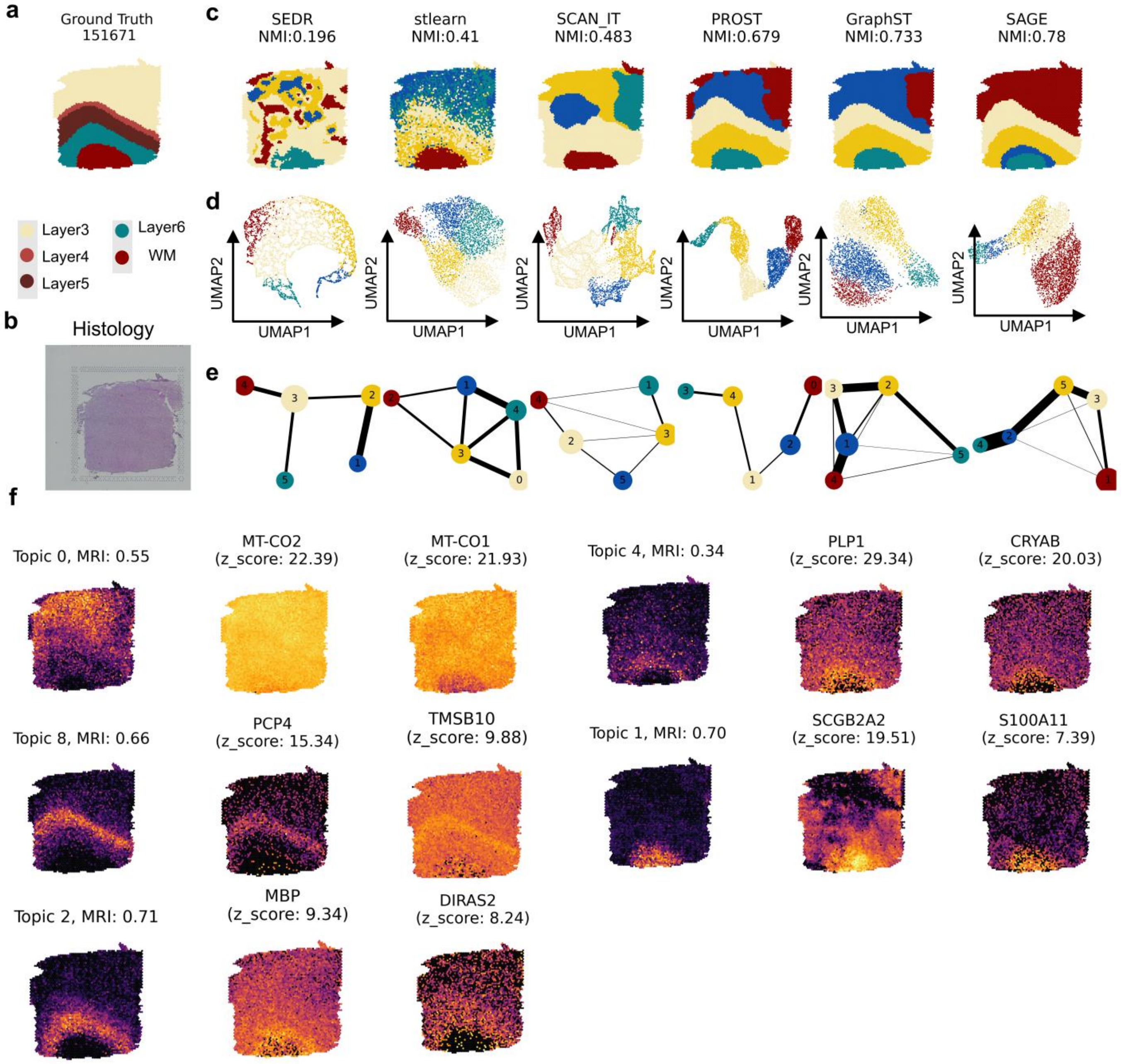

Supplementary Fig. 22 Spatial domain segmentation, UMAP visualization, PAGA graph, and identified gene co-expression topics based on the 10× Visium DLPFC dataset (Slice ID: 151671). **(a)** Manual annotations (provided by Maynard *et al.*). **(b)** H&E-stained tissue image. **(c)** Spatial domain segmentation results generated by SEDR, stLearn, SCAN-IT, PROST, GraphST, and SAGE. **(d, e)** UMAP visualizations and PAGA graphs based on low-dimensional embeddings from SEDR, stLearn, SCAN-IT, PROST, GraphST, and SAGE, with spot colors reflecting manual annotations. **(f)** Gene co-expression topics identified by SAGE exhibiting significant spatial expression patterns, along with their top contributing genes. Statistical significance was assessed using Z-scores.

Supplementary Figure 23

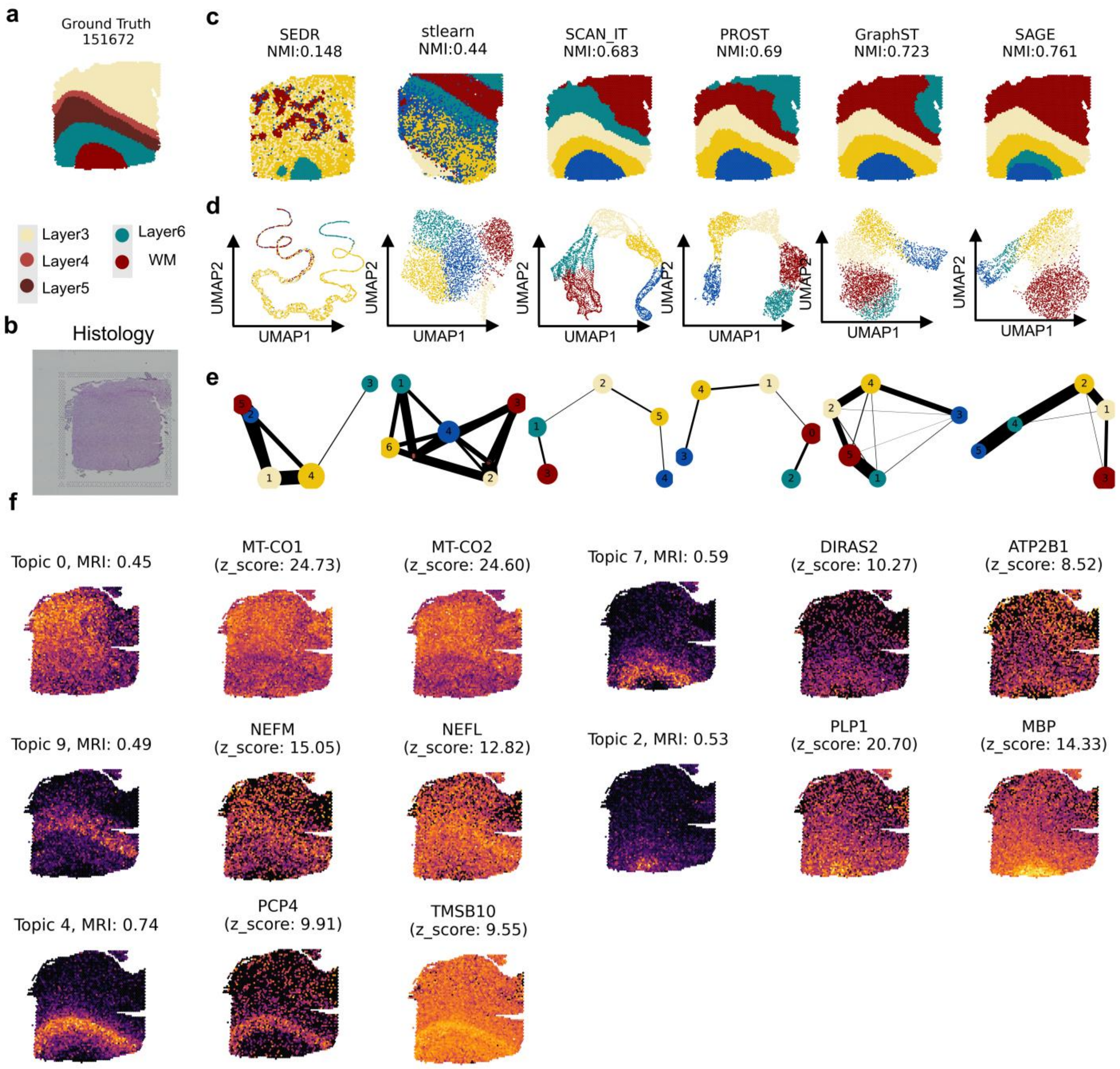

Supplementary Fig. 23 Spatial domain segmentation, UMAP visualization, PAGA graph, and identified gene co-expression topics based on the 10× Visium DLPFC dataset (Slice ID: 151672). **(a)** Manual annotations (provided by Maynard *et al.*). **(b)** H&E-stained tissue image. **(c)** Spatial domain segmentation results generated by SEDR, stLearn, SCAN-IT, PROST, GraphST, and SAGE. **(d, e)** UMAP visualizations and PAGA graphs based on low-dimensional embeddings from SEDR, stLearn, SCAN-IT, PROST, GraphST, and SAGE, with spot colors reflecting manual annotations. **(f)** Gene co-expression topics identified by SAGE exhibiting significant spatial expression patterns, along with their top contributing genes. Statistical significance was assessed using Z-scores.

Supplementary Figure 24

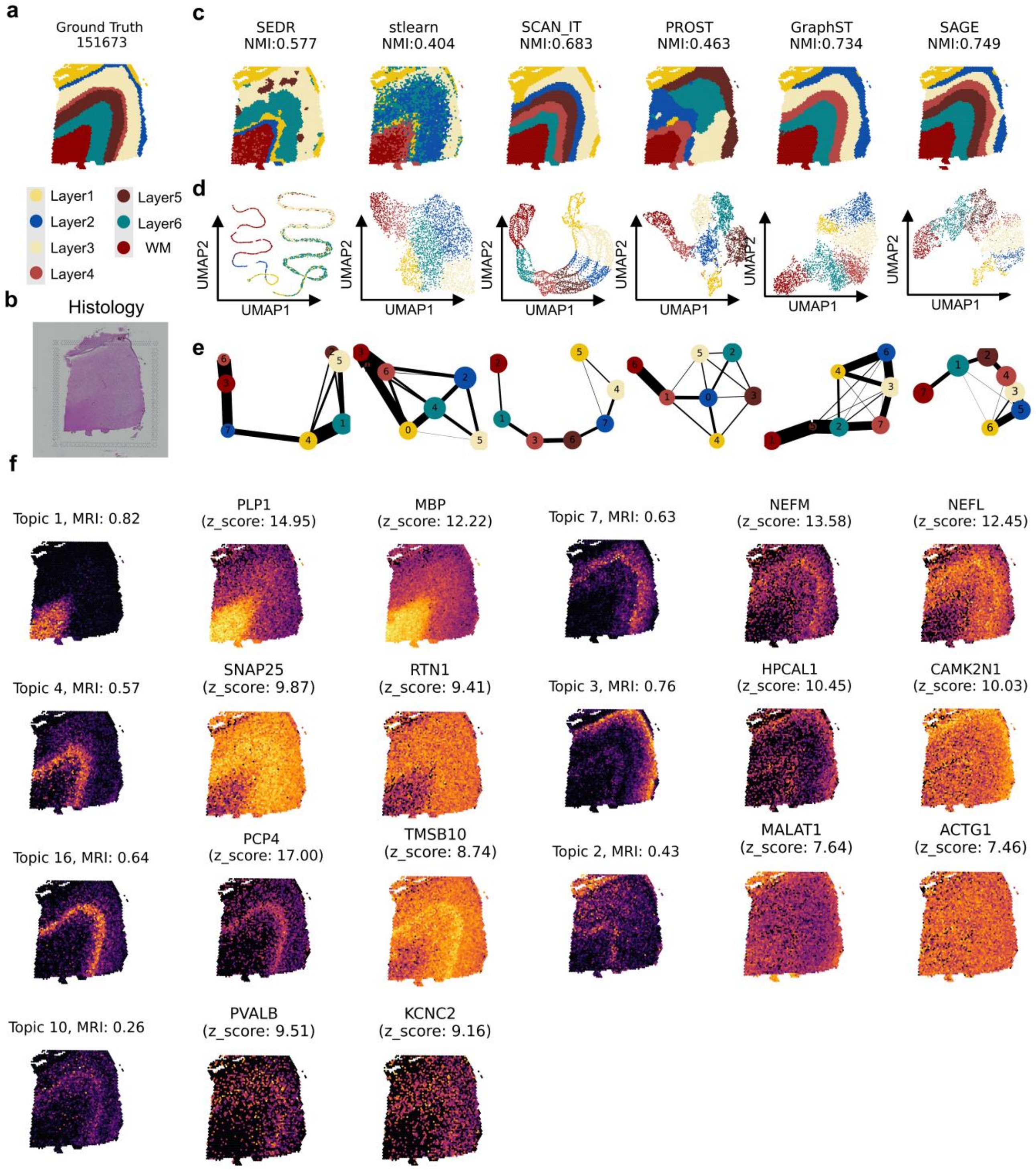

Supplementary Fig. 24 Spatial domain segmentation, UMAP visualization, PAGA graph, and identified gene co-expression topics based on the 10× Visium DLPFC dataset (Slice ID: 151673). **(a)** Manual annotations (provided by Maynard *et al.*). **(b)** H&E-stained tissue image. **(c)** Spatial domain segmentation results generated by SEDR, stLearn, SCAN-IT, PROST, GraphST, and SAGE. **(d, e)** UMAP visualizations and PAGA graphs based on low-dimensional embeddings from SEDR, stLearn, SCAN-IT, PROST, GraphST, and SAGE, with spot colors reflecting manual annotations. **(f)** Gene co-expression topics identified by SAGE exhibiting significant spatial expression patterns, along with their top contributing genes. Statistical significance was assessed using Z-scores.

Supplementary Figure 25

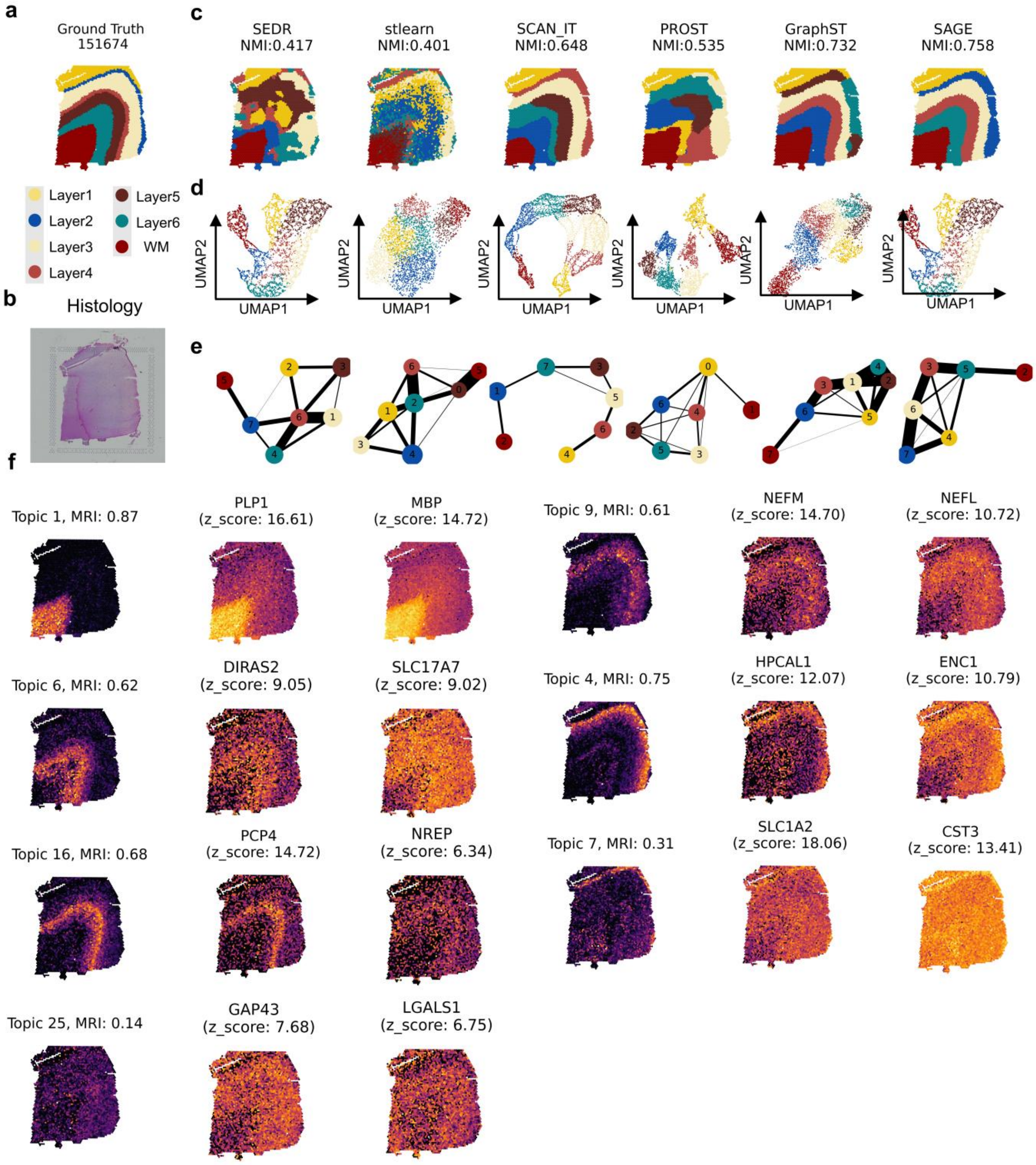

Supplementary Fig. 25 Spatial domain segmentation, UMAP visualization, PAGA graph, and identified gene co-expression topics based on the 10× Visium DLPFC dataset (Slice ID: 151674). **(a)** Manual annotations (provided by Maynard *et al.*). **(b)** H&E-stained tissue image. **(c)** Spatial domain segmentation results generated by SEDR, stLearn, SCAN-IT, PROST, GraphST, and SAGE. **(d, e)** UMAP visualizations and PAGA graphs based on low-dimensional embeddings from SEDR, stLearn, SCAN-IT, PROST, GraphST, and SAGE, with spot colors reflecting manual annotations. **(f)** Gene co-expression topics identified by SAGE exhibiting significant spatial expression patterns, along with their top contributing genes. Statistical significance was assessed using Z-scores.

Supplementary Figure 26

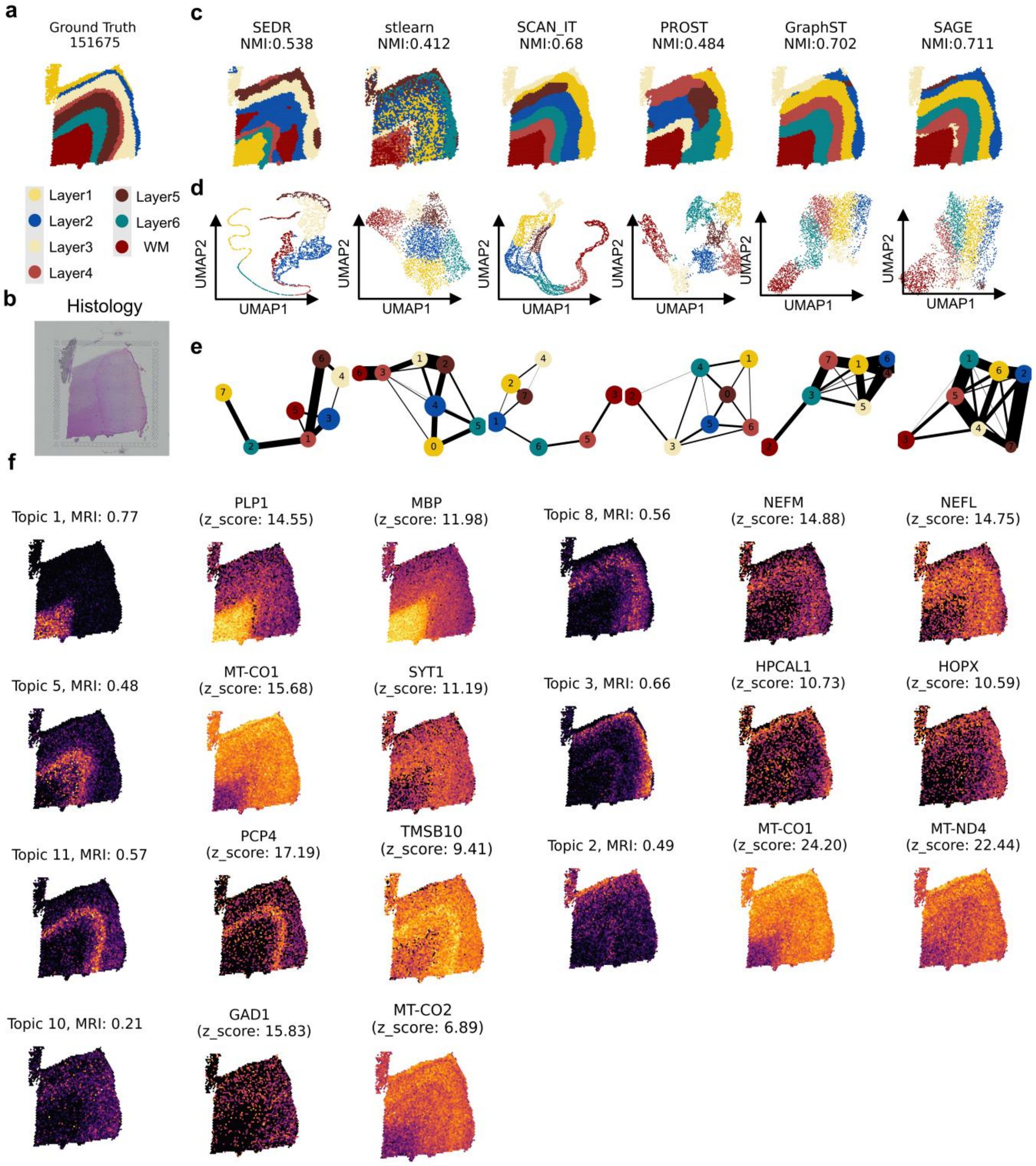

Supplementary Fig. 26 Spatial domain segmentation, UMAP visualization, PAGA graph, and identified gene co-expression topics based on the 10× Visium DLPFC dataset (Slice ID: 151675). **(a)** Manual annotations (provided by Maynard *et al.*). **(b)** H&E-stained tissue image. **(c)** Spatial domain segmentation results generated by SEDR, stLearn, SCAN-IT, PROST, GraphST, and SAGE. **(d, e)** UMAP visualizations and PAGA graphs based on low-dimensional embeddings from SEDR, stLearn, SCAN-IT, PROST, GraphST, and SAGE, with spot colors reflecting manual annotations. **(f)** Gene co-expression topics identified by SAGE exhibiting significant spatial expression patterns, along with their top contributing genes. Statistical significance was assessed using Z-scores.

Supplementary Figure 27

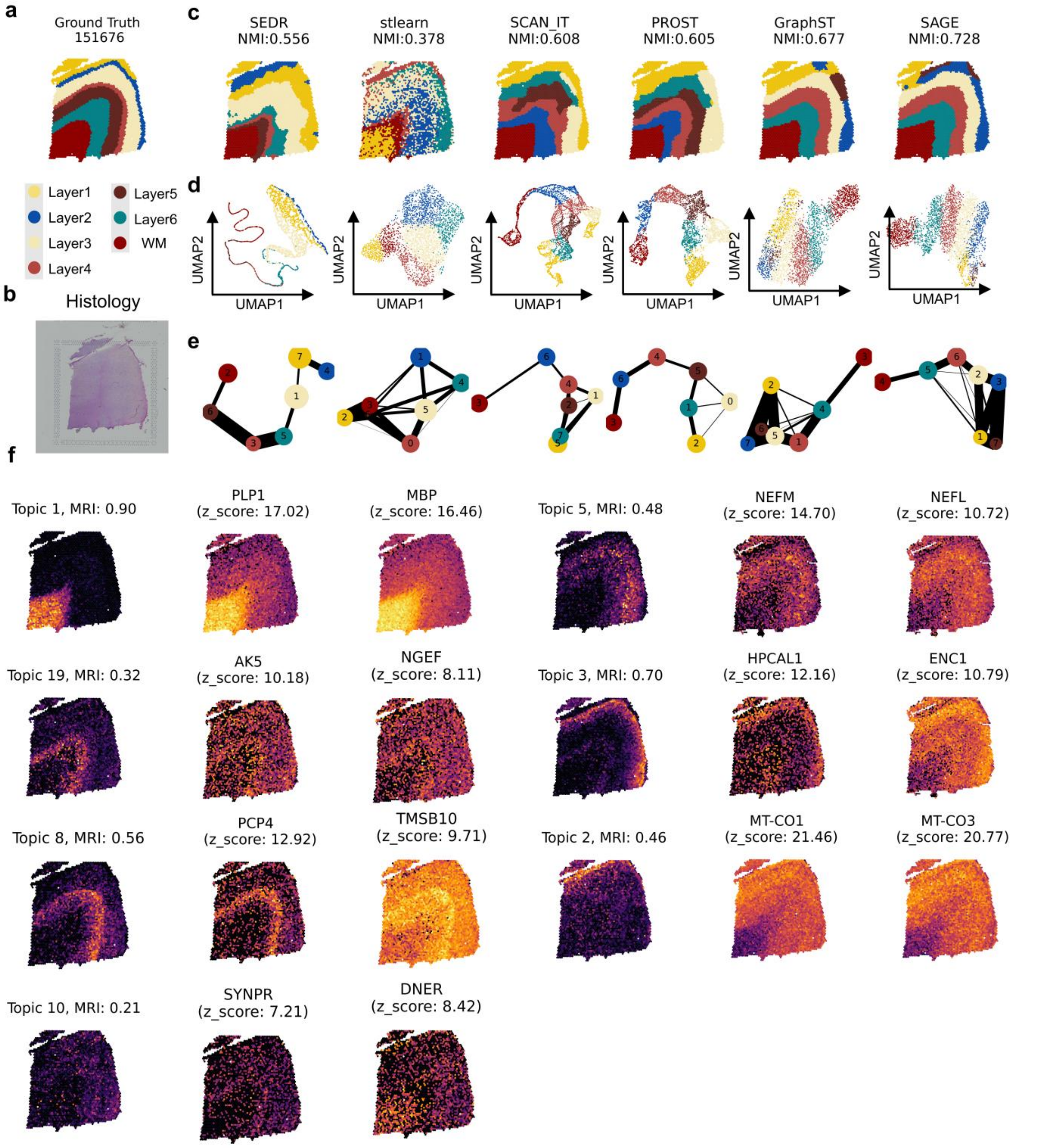

Supplementary Fig. 27 Spatial domain segmentation, UMAP visualization, PAGA graph, and identified gene co-expression topics based on the 10× Visium DLPFC dataset (Slice ID: 151676). **(a)** Manual annotations (provided by Maynard *et al.*). **(b)** H&E-stained tissue image. **(c)** Spatial domain segmentation results generated by SEDR, stLearn, SCAN-IT, PROST, GraphST, and SAGE. **(d, e)** UMAP visualizations and PAGA graphs based on low-dimensional embeddings from SEDR, stLearn, SCAN-IT, PROST, GraphST, and SAGE, with spot colors reflecting manual annotations. **(f)** Gene co-expression topics identified by SAGE exhibiting significant spatial expression patterns, along with their top contributing genes. Statistical significance was assessed using Z-scores.

Supplementary Figure 28

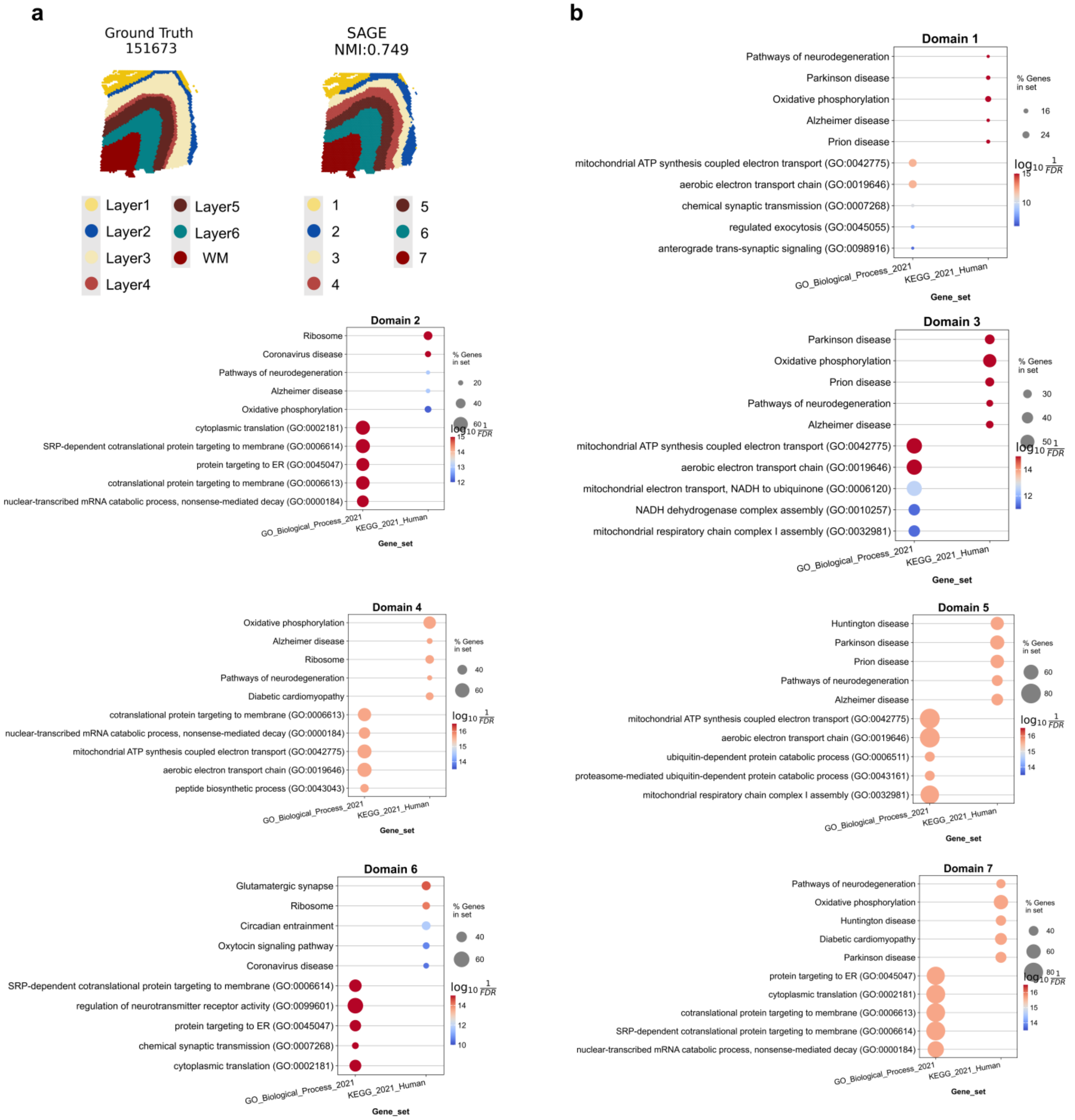

Supplementary Fig. 28 Spatial domain visualization and pathway enrichment analysis of slice 151673. **(a)** Left: ground truth spatial domains for slice 151673. Right: domain segmentation results obtained by SAGE (NMI = 0.749). The color legend below indicates the seven identified domains. **(b)** Pathway enrichment analysis for each domain (Domain 1–Domain 7, top to bottom, left to right). For each domain, the top ten enriched pathways are shown as bubble plots: the first five correspond to KEGG_2021_Human, and the last five correspond to GO_Biological_Process_2021. Each bubble represents a pathway, where the bubble size indicates the number of genes overlapping with the pathway, and the color gradient reflects the adjusted p-value (FDR-corrected). The x-axis represents the enrichment score or –log10(p-value), and pathway names are labeled next to each bubble.

Supplementary Figure 29

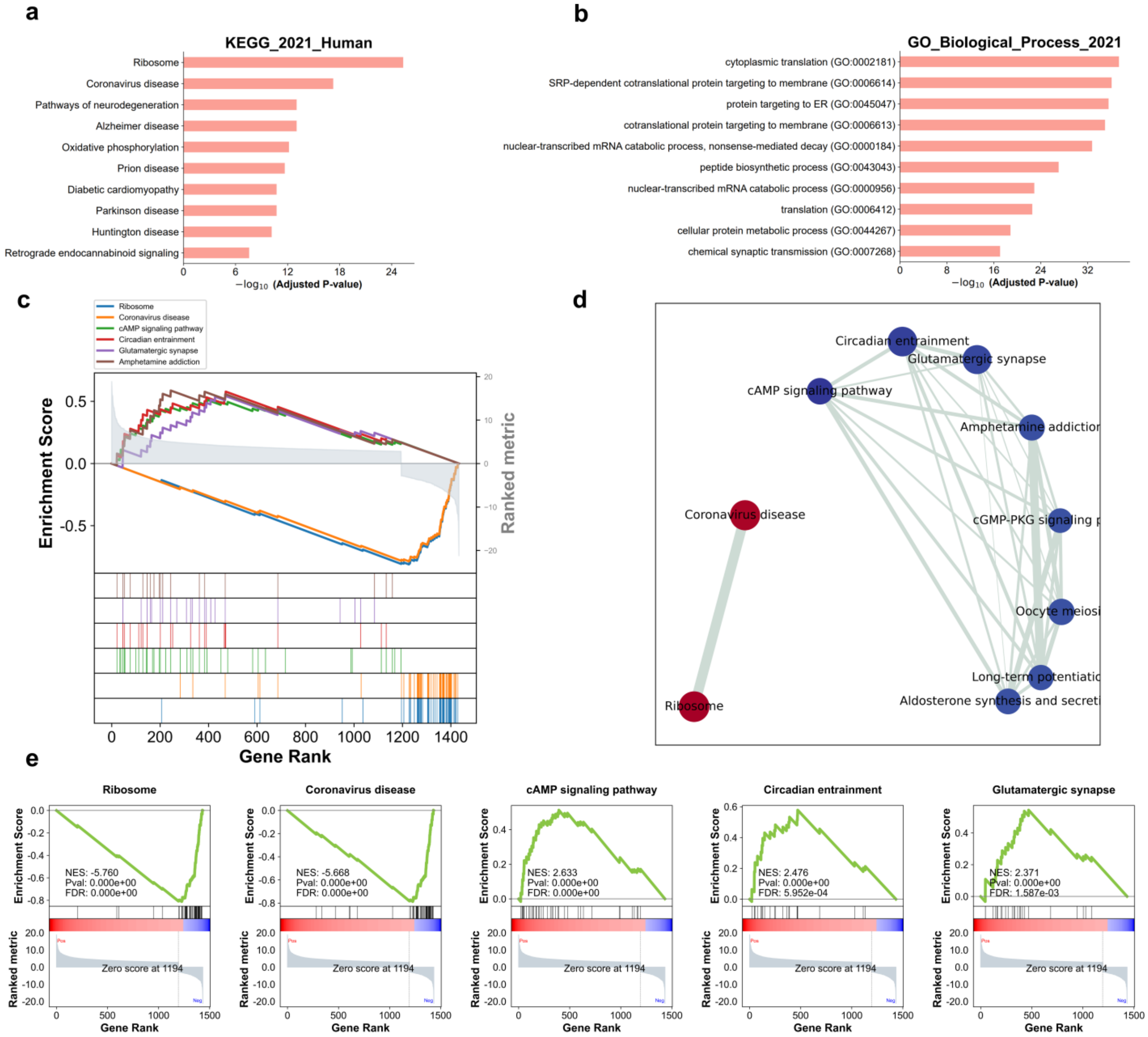

Supplementary Fig. 29 Functional characterization of Domain 5 in slice 151673. **(a)** KEGG 2021 Human pathway enrichment analysis for genes in Domain 2. The bubble size represents the number of overlapping genes, and the color indicates the significance level (–log10 FDR-adjusted p-value). The top five enriched KEGG pathways are displayed. **(b)** GO Biological Process 2021 enrichment analysis for genes in Domain 2. Similar to panel (a), bubble size corresponds to gene counts, and color reflects adjusted p-value significance. The top five GO terms are shown. **(c)** Gene Set Enrichment Analysis (GSEA) for Domain 2. The left y-axis indicates the Enrichment Score (ES), the right y-axis shows the Ranked Metric, and the x-axis corresponds to the Gene Rank across all genes. The plot highlights pathways with significant enrichment. **(d)** Enrichment Network Map for Domain 2. Nodes represent enriched pathways, node size indicates the number of overlapping genes, and node color reflects enrichment significance (–log10 FDR). Edges denote gene overlap between pathways, and edge thickness corresponds to the number of shared genes. **(e)** Detailed visualization of six representative pathways in Domain 2. Each panel shows individual pathway-specific enrichment results, including gene coverage, enrichment scores, and significance metrics.

Supplementary Figure 30

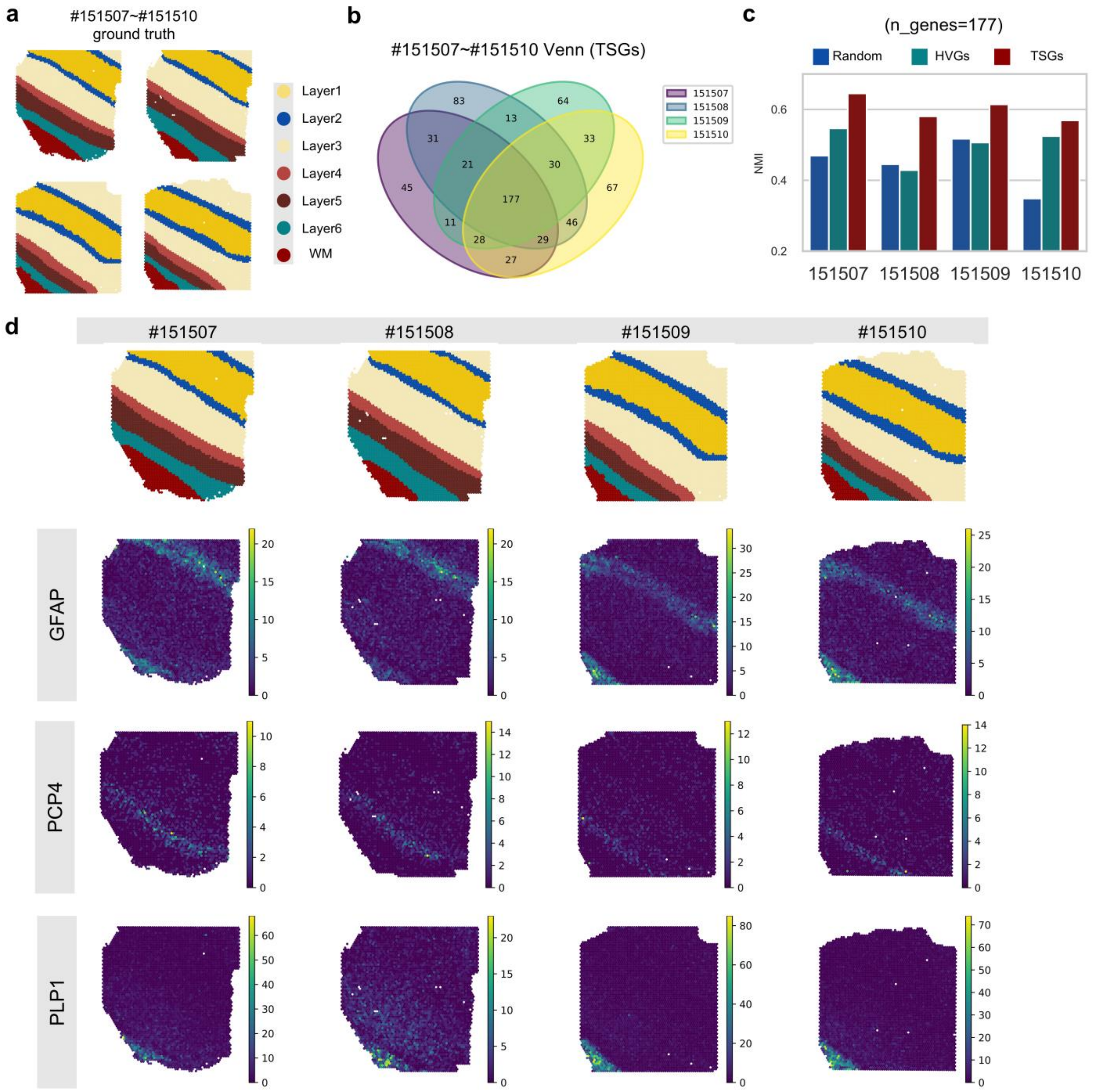

Supplementary Fig. 30 Cross-slice spatial consistency analysis of SAGE-selected genes in the DLPFC dataset (151507–151510). **(a)** Ground truth spatial domains of 4 adjacent DLPFC slices (151507–151510). The color legend on the right indicates the annotated cortical layers. **(b)** Venn diagram of topic-specific gene (TSG) sets identified by SAGE across the four slices, illustrating both shared and slice-specific genes. **(c)** Bar plot of clustering performance (NMI) across the same slices using three gene sets—Random (blue), HVGs (green), and TSGs (red)—with an equal gene number of 177 per set. TSGs consistently achieve higher NMI values, indicating superior spatial discriminability. **(d)** Spatial expression patterns of representative cross-slice consistent genes (GFAP, PCP4, and PLP1). The top row shows ground truth spatial domains for reference, while the following rows display the spatial distributions of each gene across the four slices, revealing clear and reproducible laminar expression patterns.

Supplementary Figure 31

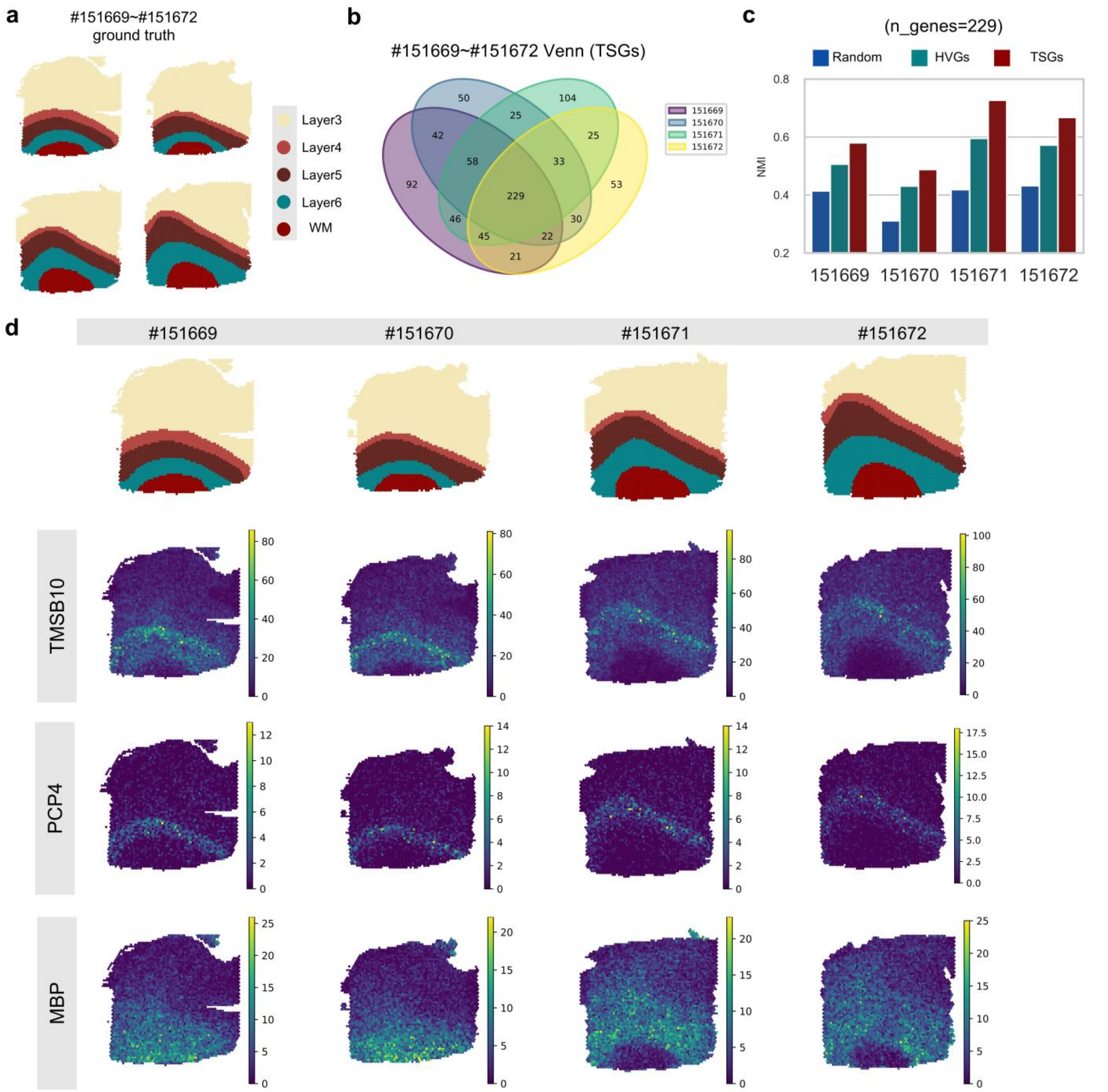

Supplementary Fig. 31 Cross-slice spatial consistency analysis of SAGE-selected genes in the DLPFC dataset (151669–151672). **(a)** Ground truth spatial domains of 4 adjacent DLPFC slices (151669–151672). The color legend on the right indicates the annotated cortical layers. **(b)** Venn diagram of topic-specific gene (TSG) sets identified by SAGE across the 4 slices, illustrating both shared and slice-specific genes. **(c)** Bar plot of clustering performance (NMI) across the same slices using three gene sets—Random (blue), HVGs (green), and TSGs (red)—with an equal gene number of 177 per set. TSGs consistently achieve higher NMI values, indicating superior spatial discriminability. **(d)** Spatial expression patterns of representative cross-slice consistent genes (TMSB10, PCP4, and MBP). The top row shows ground truth spatial domains for reference, while the following rows display the spatial distributions of each gene across the 4 slices, revealing clear and reproducible laminar expression patterns.

Supplementary Figure 32

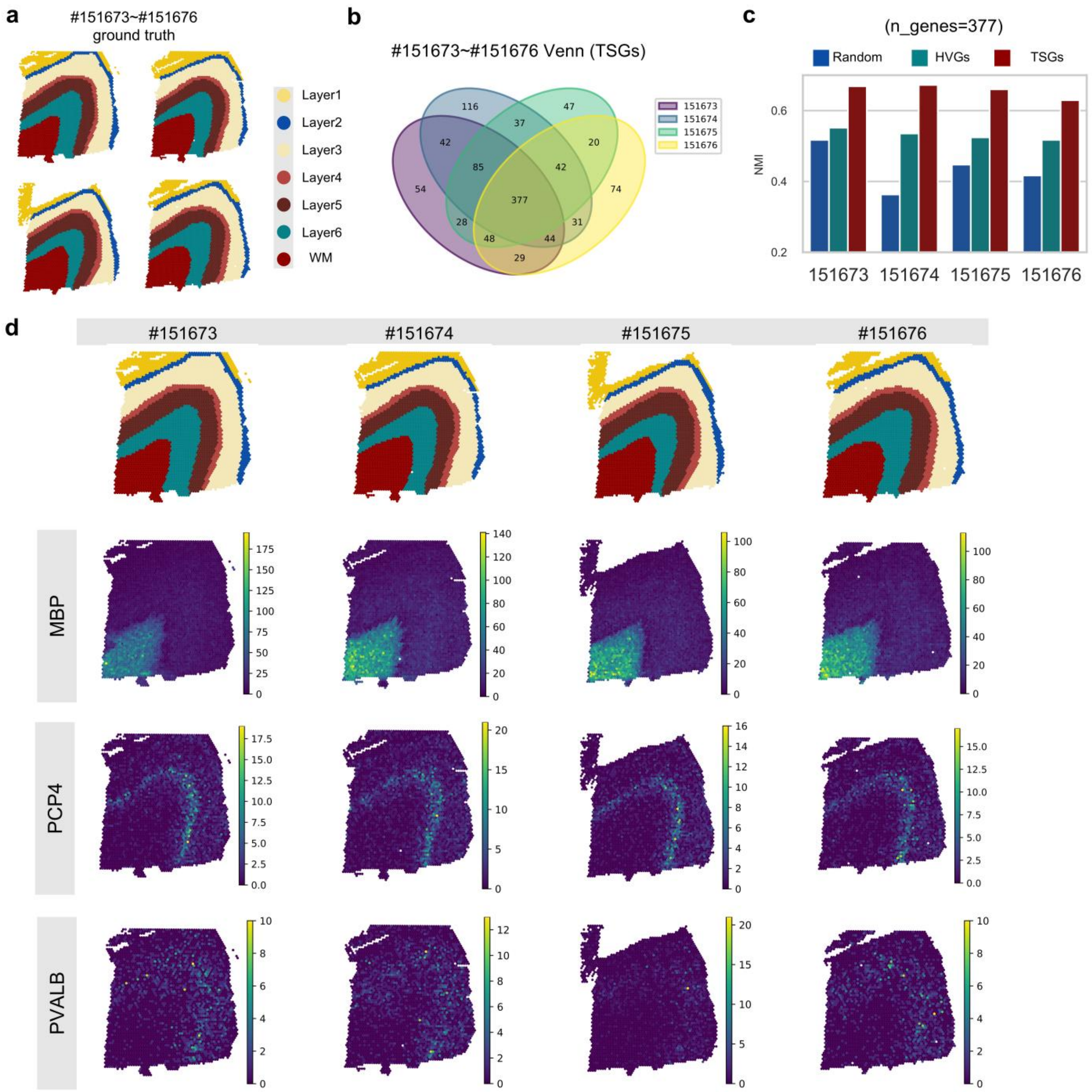

Supplementary Fig. 32 Cross-slice spatial consistency analysis of SAGE-selected genes in the DLPFC dataset (151673–151676). **(a)** Ground truth spatial domains of 4 adjacent DLPFC slices (151673–151676). The color legend on the right indicates the annotated cortical layers. **(b)** Venn diagram of topic-specific gene (TSG) sets identified by SAGE across the 4 slices, illustrating both shared and slice-specific genes. **(c)** Bar plot of clustering performance (NMI) across the same slices using three gene sets—Random (blue), HVGs (green), and TSGs (red)—with an equal gene number of 177 per set. TSGs consistently achieve higher NMI values, indicating superior spatial discriminability. **(d)** Spatial expression patterns of representative cross-slice consistent genes (MBP, PCP4, and PVALB). The top row shows ground truth spatial domains for reference, while the following rows display the spatial distributions of each gene across the 4 slices, revealing clear and reproducible laminar expression patterns.

Supplementary Figure 33

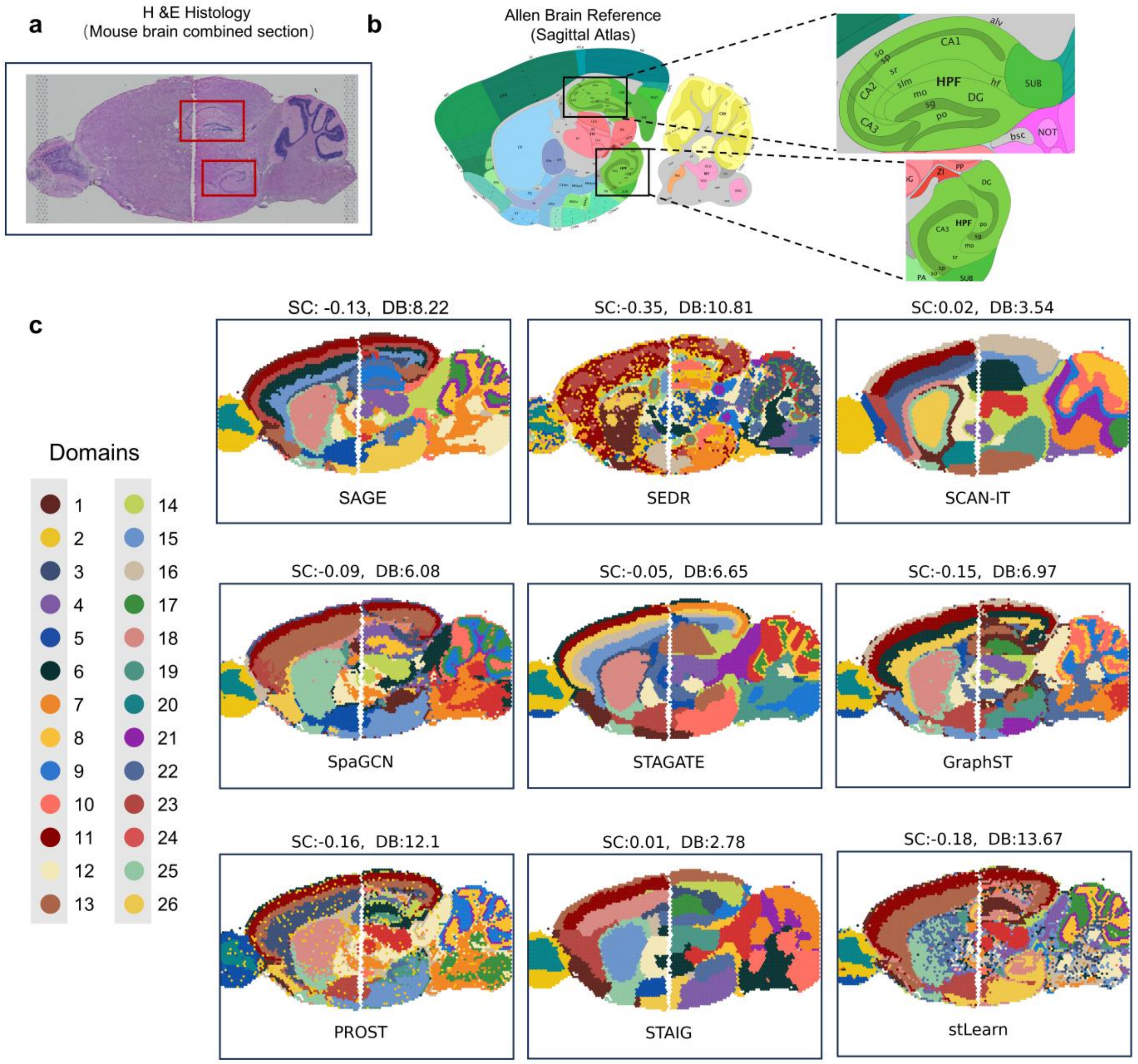

Supplementary Fig. 33 Spatial domain segmentation and comparison with H&E staining and the Allen Mouse Brain Atlas on a sagittal section of the mouse brain. **(a)** H&E-stained image of a sagittal section of the mouse brain, with red boxes indicating the dorsal and ventral regions of the hippocampal formation (HPF), including the CA and DG subregions. **(b)** Corresponding anatomical annotations from the Allen Mouse Brain Atlas. **(c)** Spatial domain segmentation results generated by nine computational methods: SEDR, stLearn, SCAN-IT, SpaGCN, STAGATE, GraphST, PROST, STAIG, and SAGE.

Supplementary Figure 34

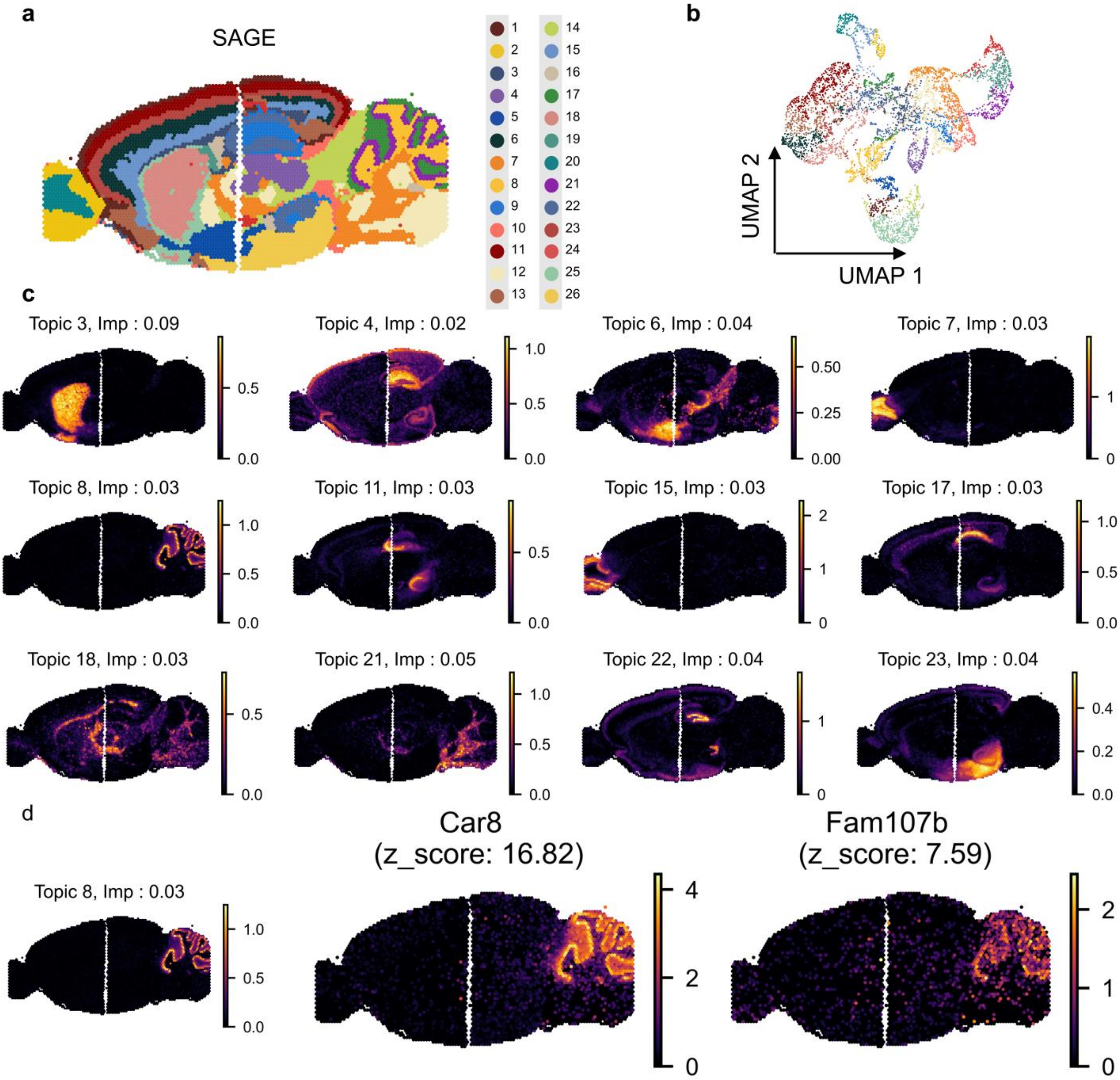

Supplementary Fig. 34 Spatial domain segmentation and gene co-expression topic analysis of the mouse brain sagittal section using SAGE. **(b)** UMAP visualization based on the spatial domains identified by SAGE, with colors corresponding to different spatial domains. **(c)** Gene co-expression topics identified by SAGE that exhibit significant spatial expression patterns, with statistical importance assessed using random forest–based importance scores (Imp). **(d)** Marker genes identified within Topic 8, a Purkinje cell layer–specific module, including *Car8* (z-score = 16.82) and *Fam107b* (z-score = 7.59). Source data are provided in the Source Data file.

Supplementary Figure 35

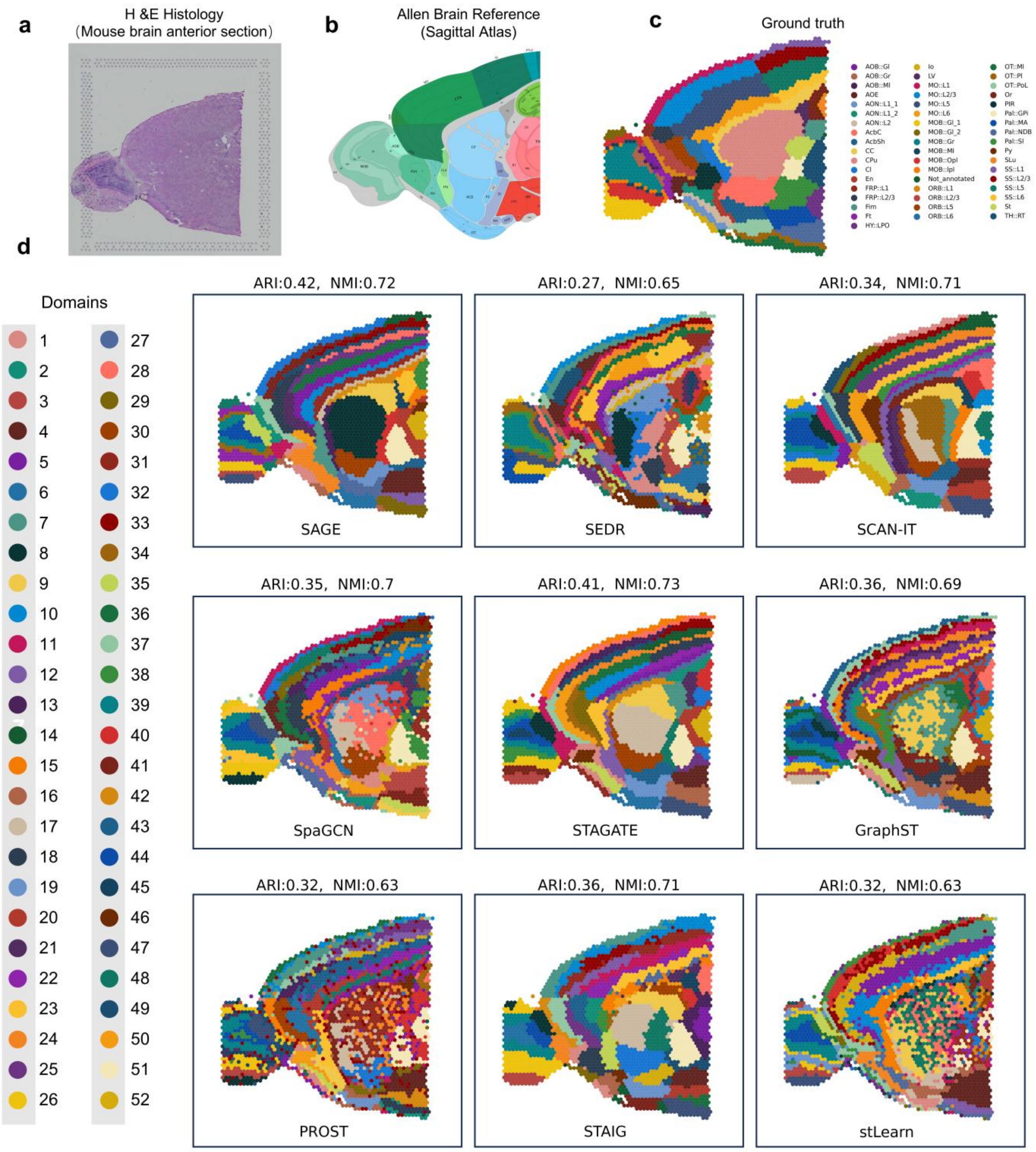

Supplementary Fig. 35 Spatial domain segmentation and comparison with H&E staining and the Allen Mouse Brain Atlas on the mouse forebrain sagittal section. **(a)** H&E-stained image of the mouse forebrain sagittal section. **(b)** Anatomical annotations from the Allen Mouse Brain Atlas. **(c)** Manual annotations provided by Long et al. **(d)** Spatial domain segmentation results from SAGE and other nine methods (SEDR, stLearn, SCAN-IT, SpaGCN, STAGATE, GraphST, PROST, STAIG, and SAGE).

Supplementary Figure 36

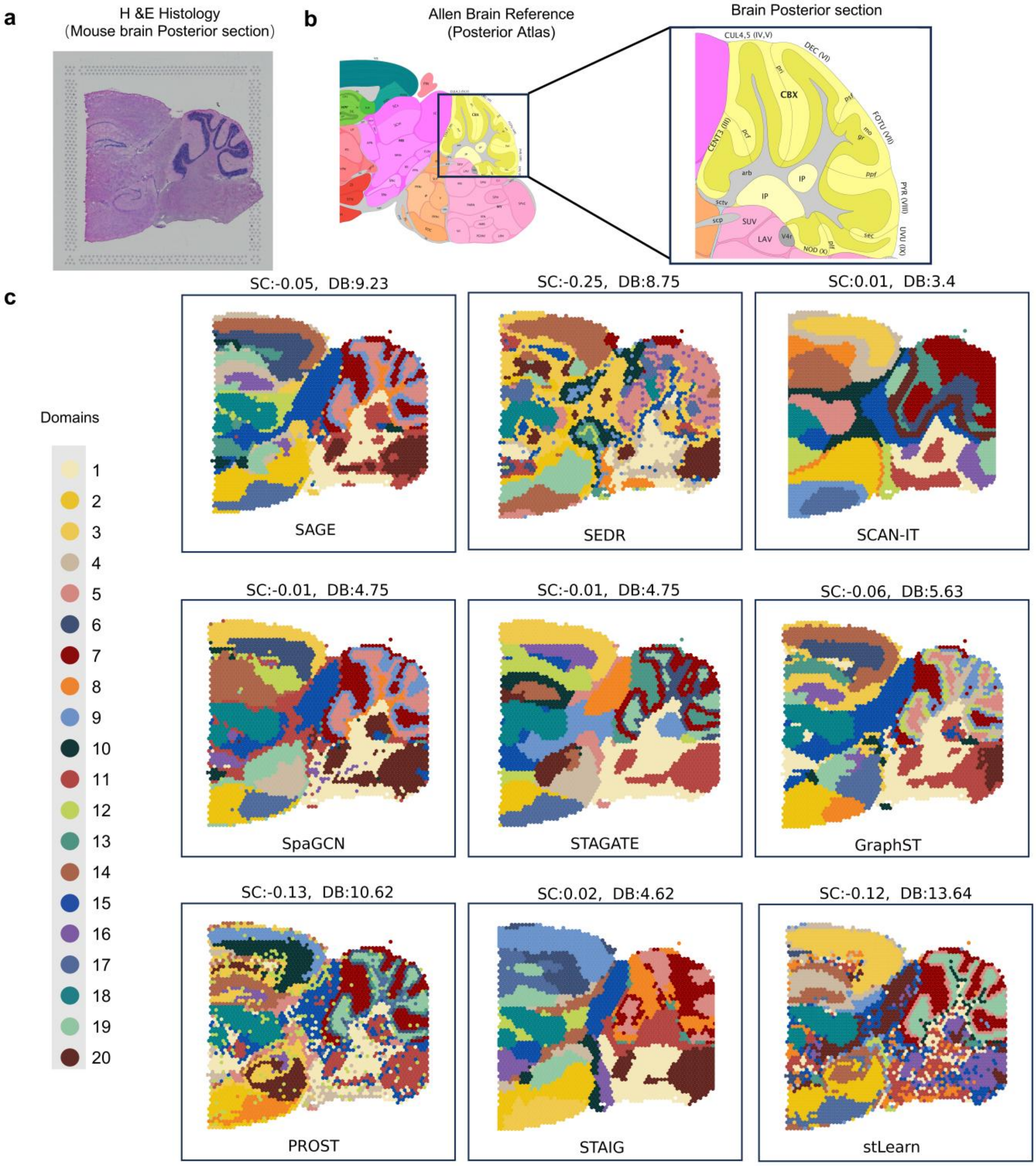

Supplementary Fig. 36 Spatial domain segmentation and comparison with H&E staining and the Allen Mouse Brain Atlas on the mouse hindbrain sagittal section. **(a)** H&E-stained image of the mouse hindbrain sagittal section. **(b)** Anatomical annotations from the Allen Mouse Brain Atlas. **(c)** Spatial domain segmentation results from SAGE and other nine methods (SEDR, stLearn, SCAN-IT, SpaGCN, STAGATE, GraphST, PROST, STAIG, and SAGE).

Supplementary Figure 37

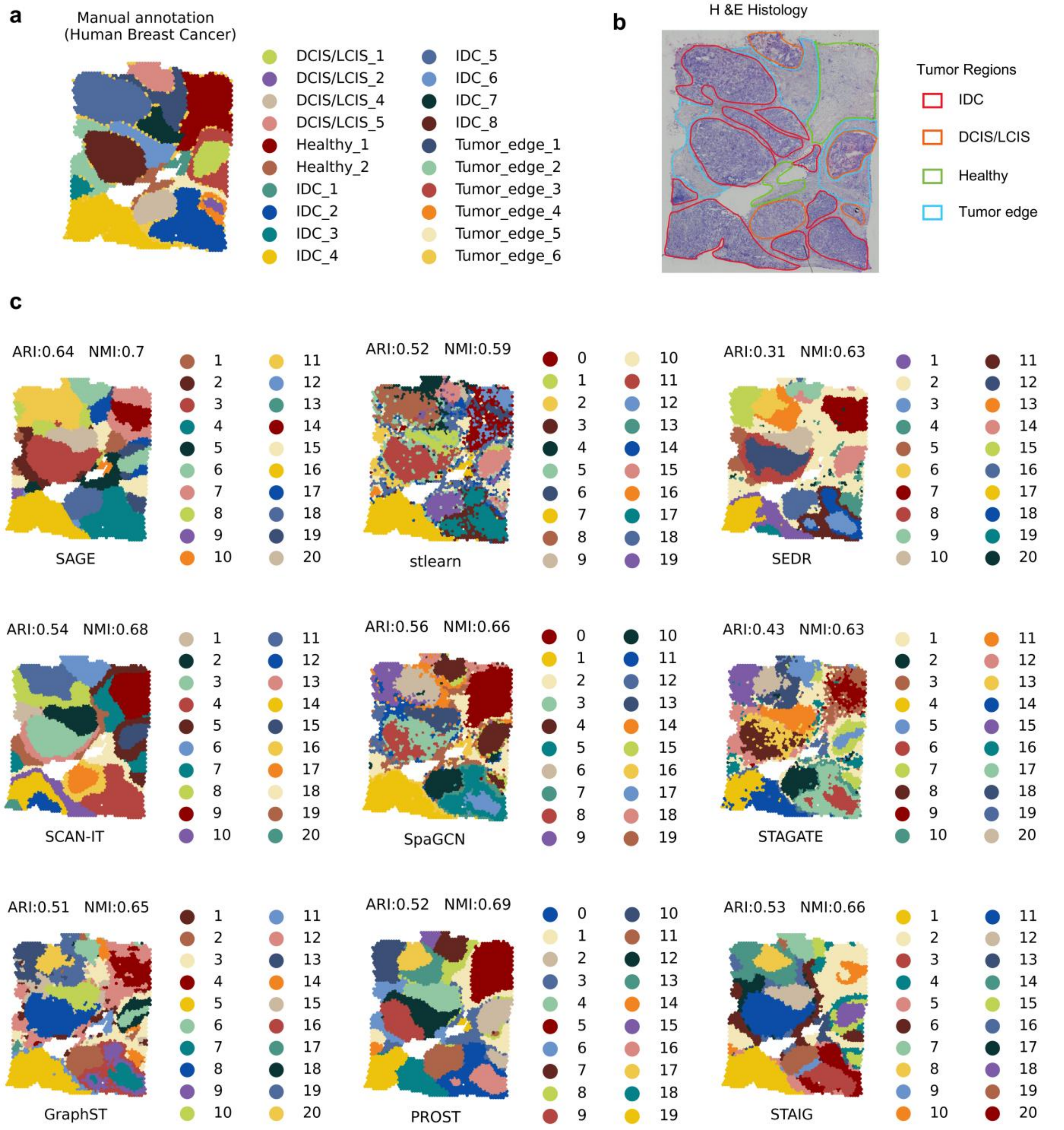

Supplementary Fig. 37 Comparison of spatial domain segmentation methods on human breast cancer tissue sections. **(a)** Manually annotated spatial domains (n = 20), provided by the Fu *et al*. **(b)** The tissue section comprises four major histopathological types: ductal/lobular carcinoma in situ (DCIS/LCIS), invasive ductal carcinoma (IDC), normal healthy tissue, and tumor edge regions. **(c)** Comparison of spatial domain segmentation results generated by nine spatial transcriptomics analysis methods: SEDR, stLearn, SCAN-IT, SpaGCN, STAGATE, GraphST, PROST, STAIG, and SAGE.

Supplementary Figure 38

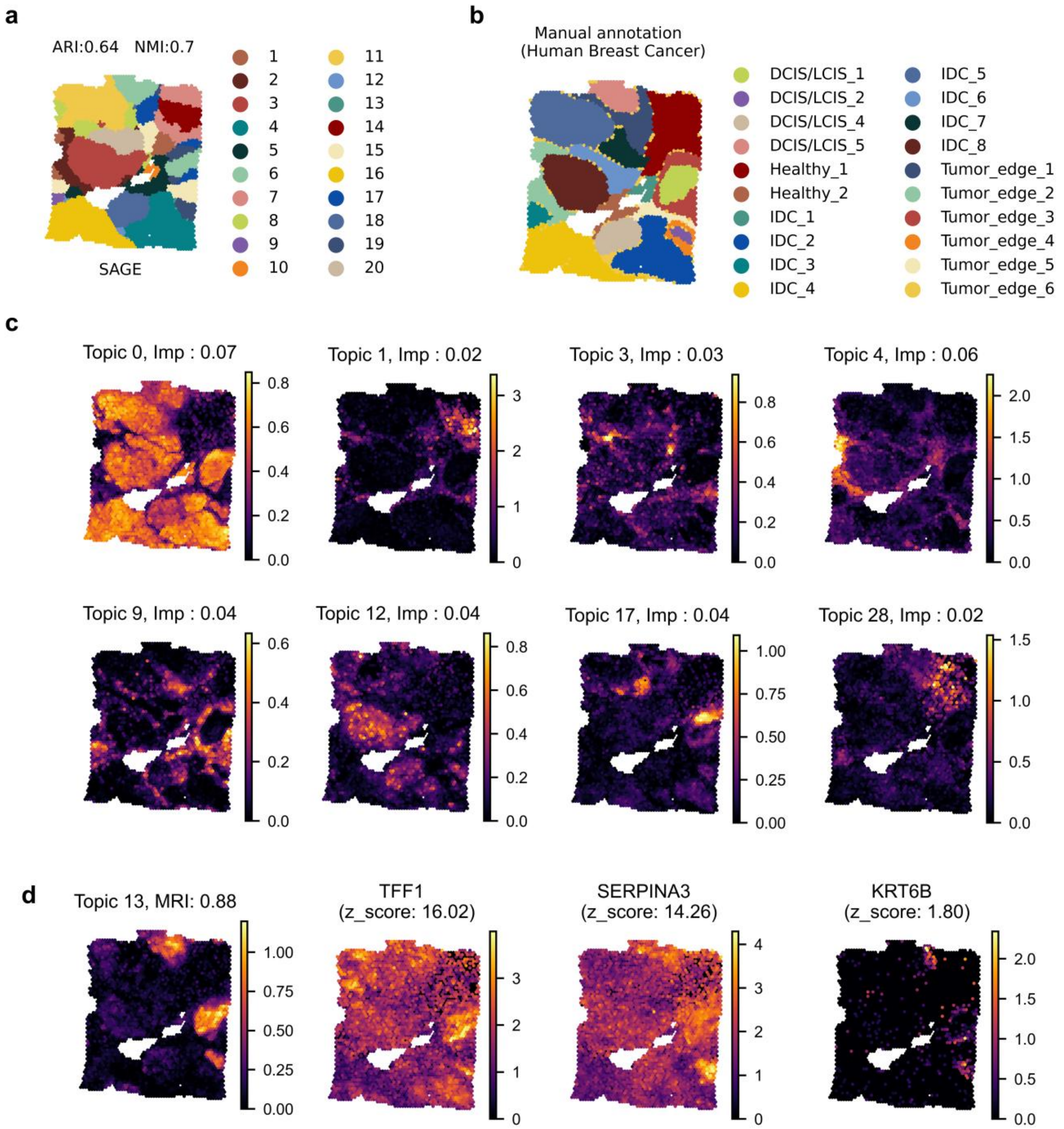

Supplementary Fig. 38 Spatial domain segmentation and identification of differentially gene co-expression topic in human breast cancer tissue by SAGE. **(a)** Spatial domain segmentation of breast cancer tissue obtained using the SAGE. **(b)** Reference manual annotations of spatial domains (n = 20), provided by Fu *et al.* **(c)** Spatially differentially gene co-expression topic identified by SAGE, with significance evaluated using random forest importance scores (Imp.). **(d)** Expression profiles of significantly upregulated marker genes in Topic 13 (specific to DCIS/LCIS_1, 2, 5) relative to DCIS/LCIS_4: *TFF1* (z-score = 16.02), *SERPINA3* (z-score = 14.26), and *KPT6B* (z-score = 1.8).

Supplementary Figure 39

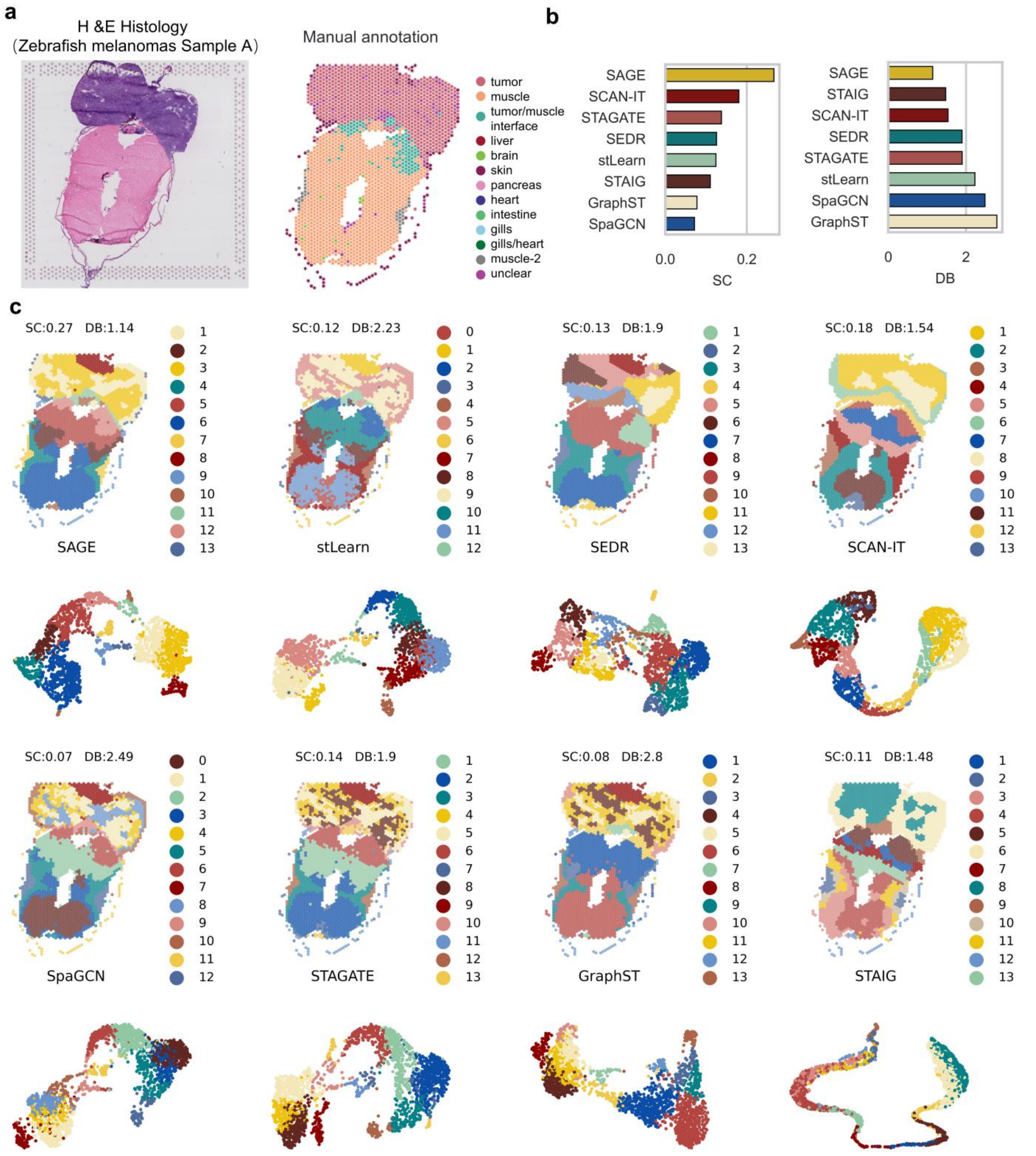

Supplementary Fig. 39 Spatial domain analysis evaluation of zebrafish melanomas sample A. **(a)** H&E staining image of sample A and manually annotated spatial domains (n=13), provided by Hunter *et al.* **(b)** Comparison of clustering quality evaluation metrics, including the SC and DB, between SAGE and other methods. **(c)** Comparison of spatial domain segmentation results and generated UMAP visualizations from eight methods (SEDR, stLearn, SCAN-IT, SpaGCN, STAGATE, GraphST, STAIG, and SAGE).

Supplementary Figure 40

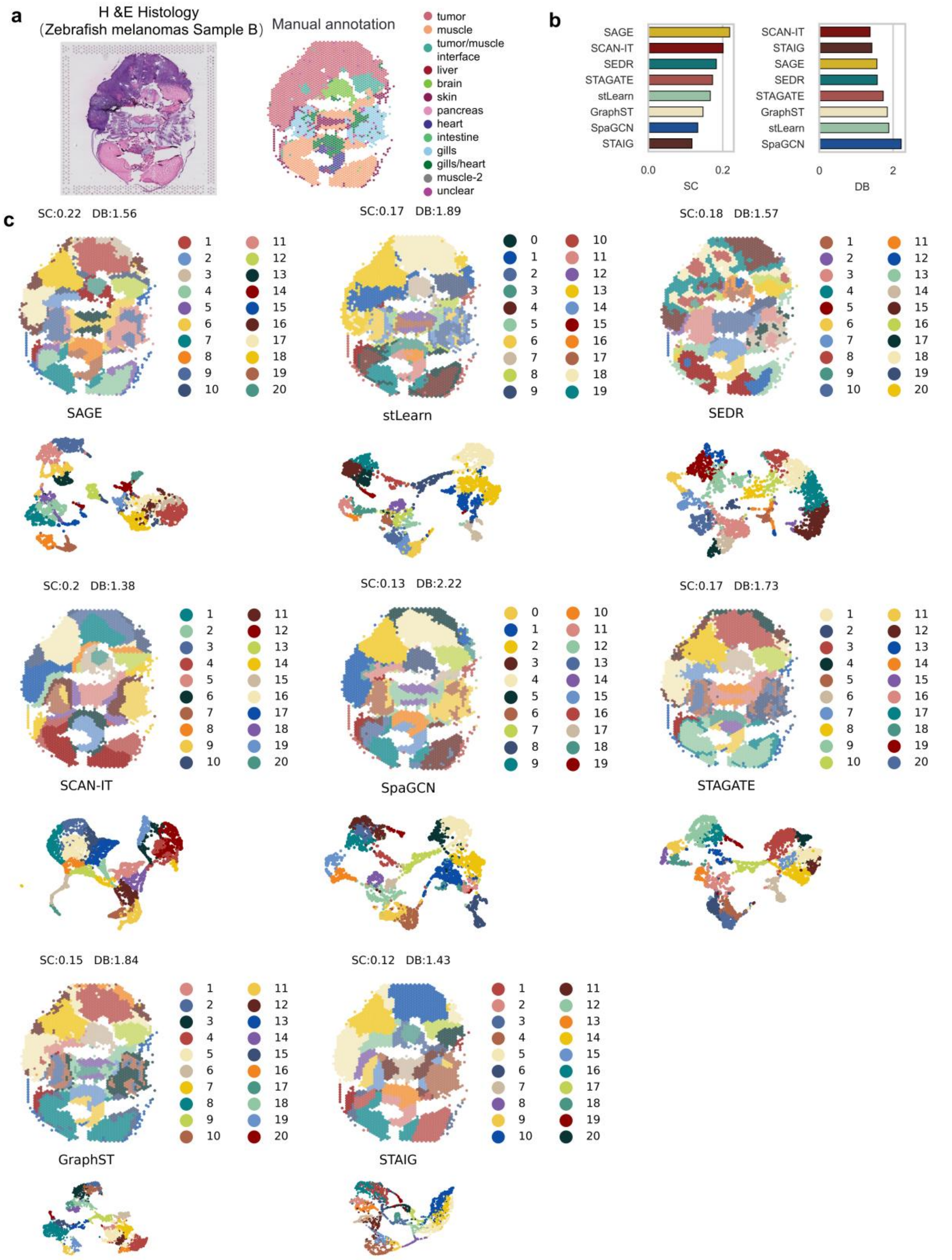

Supplementary Fig. 40 Spatial domain analysis evaluation of zebrafish melanomas sample B. **(a)** H&E staining image of sample B and manually annotated spatial domains (n=13), provided by Hunter *et al.* **(b)** Comparison of clustering quality evaluation metrics, including the SC and DB, between SAGE and other methods. **(c)** Comparison of spatial domain segmentation results (n =20) and generated UMAP visualizations from eight methods (SEDR, stLearn, SCAN-IT, SpaGCN, STAGATE, GraphST, STAIG, and SAGE).

Supplementary Figure 41

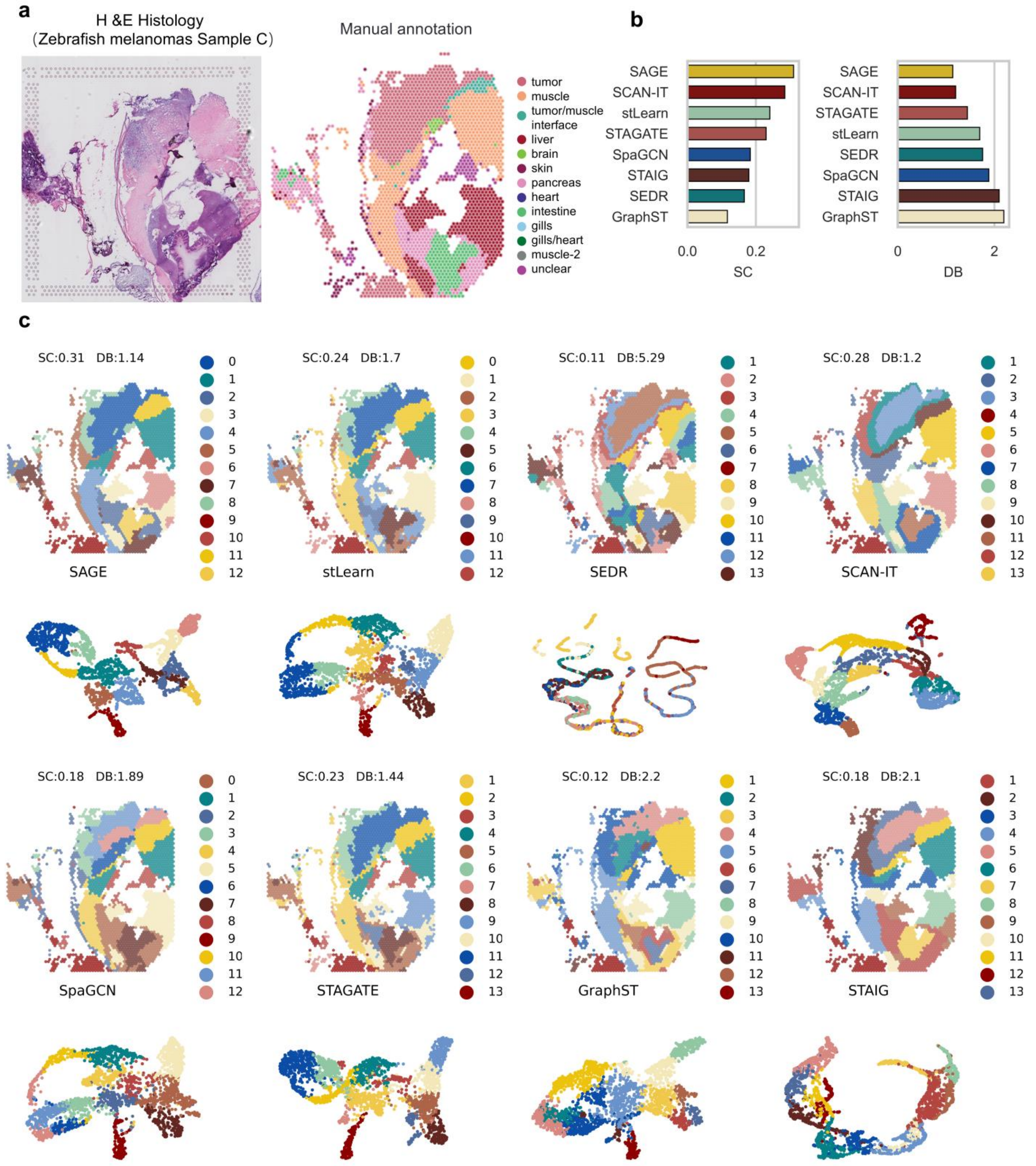

Supplementary Fig. 41 Spatial domain analysis evaluation of zebrafish melanomas sample C. **(a)** H&E staining image of sample C and manually annotated spatial domains (n=13), provided by Hunter *et al.* **(b)** Comparison of clustering quality evaluation metrics, including the SC and DB, between SAGE and other methods. **(c)** Comparison of spatial domain segmentation results and generated UMAP visualizations from eight methods (SEDR, stLearn, SCAN-IT, SpaGCN, STAGATE, GraphST, STAIG, and SAGE)

Supplementary Figure 42

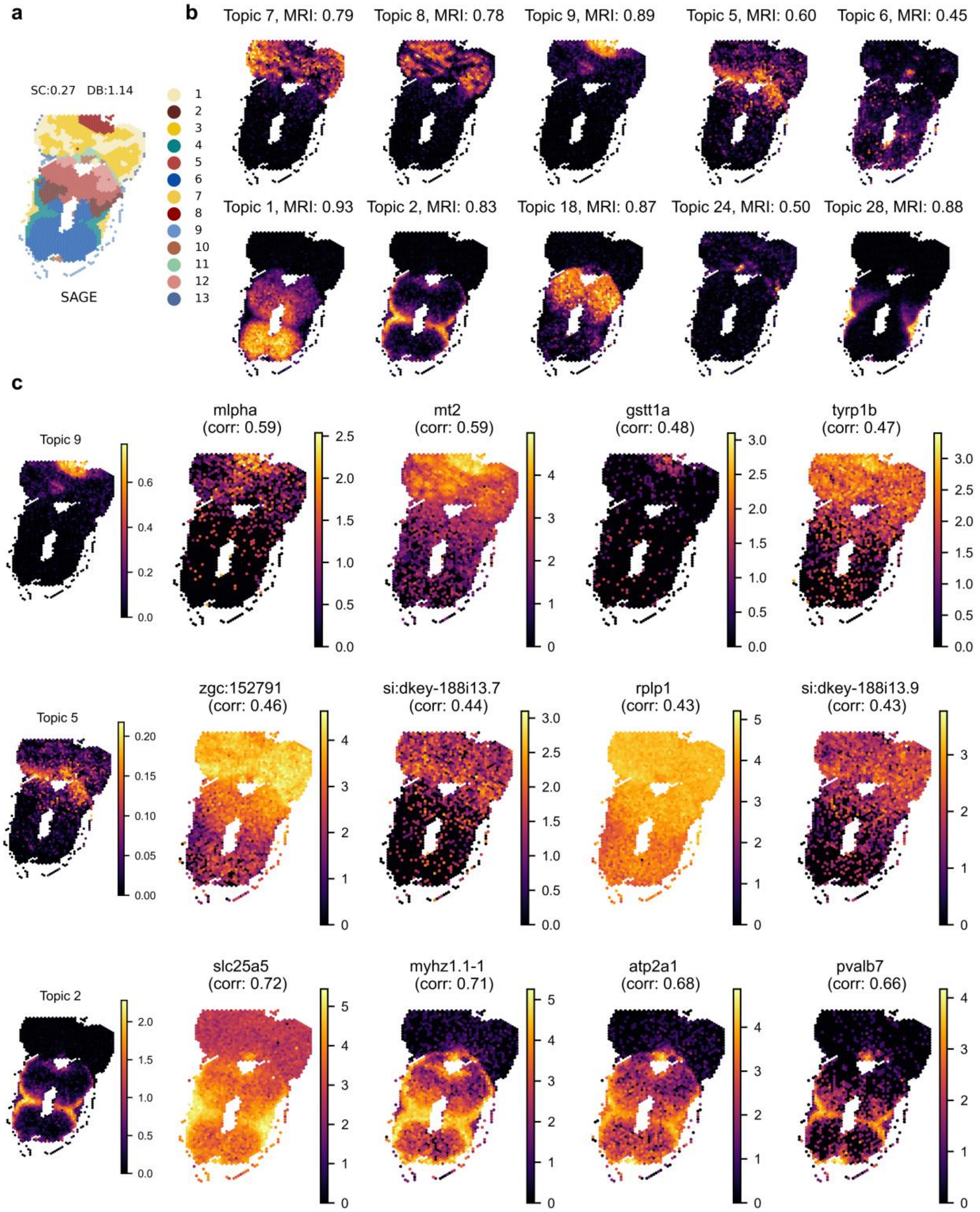

Supplementary Fig. 42 Spatial domain analysis and identification of gene co-expression topics by SAGE in zebrafish melanoma sample A. **(a)** Visualization of spatial domain segmentation results for zebrafish melanoma sample A obtained using SAGE. **(b)** Gene co-expression topics identified by SAGE show distinct spatial expression patterns. The spatial variability significance for each topic is quantified using the Moran's I score (MRI). **(c)** Spatial distributions of Topics 9, 5, and 2, along with their associated high-expression genes. The correlation (corr.) values represent Pearson correlation coefficients between topic probability maps and corresponding gene expression profiles.

Supplementary Figure 43

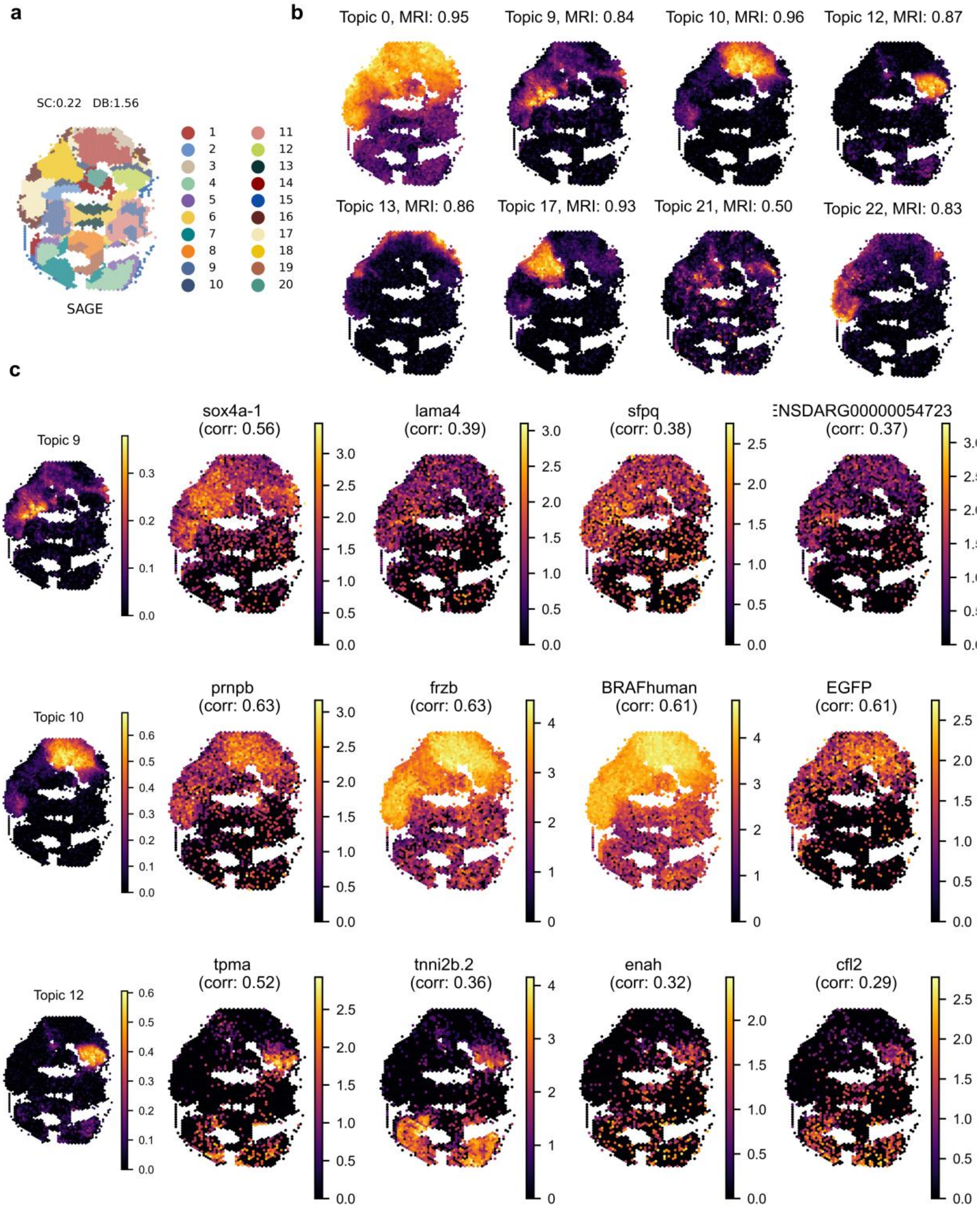

Supplementary Fig. 43 Spatial domain analysis and identification of gene co-expression topics by SAGE in zebrafish melanoma sample B. **(a)** Visualization of spatial domain segmentation results for zebrafish melanoma sample B obtained using SAGE. **(b)** Gene co-expression topics identified by SAGE show distinct spatial expression patterns. The spatial variability significance for each topic is quantified using the Moran's I score (MRI). **(c)** Spatial distributions of Topics 9, 10, and 12 in tumor-associated regions, along with their associated high-expression genes. The correlation (corr.) values represent Pearson correlation coefficients between topic probability maps and corresponding gene expression profiles.

Supplementary Figure 44

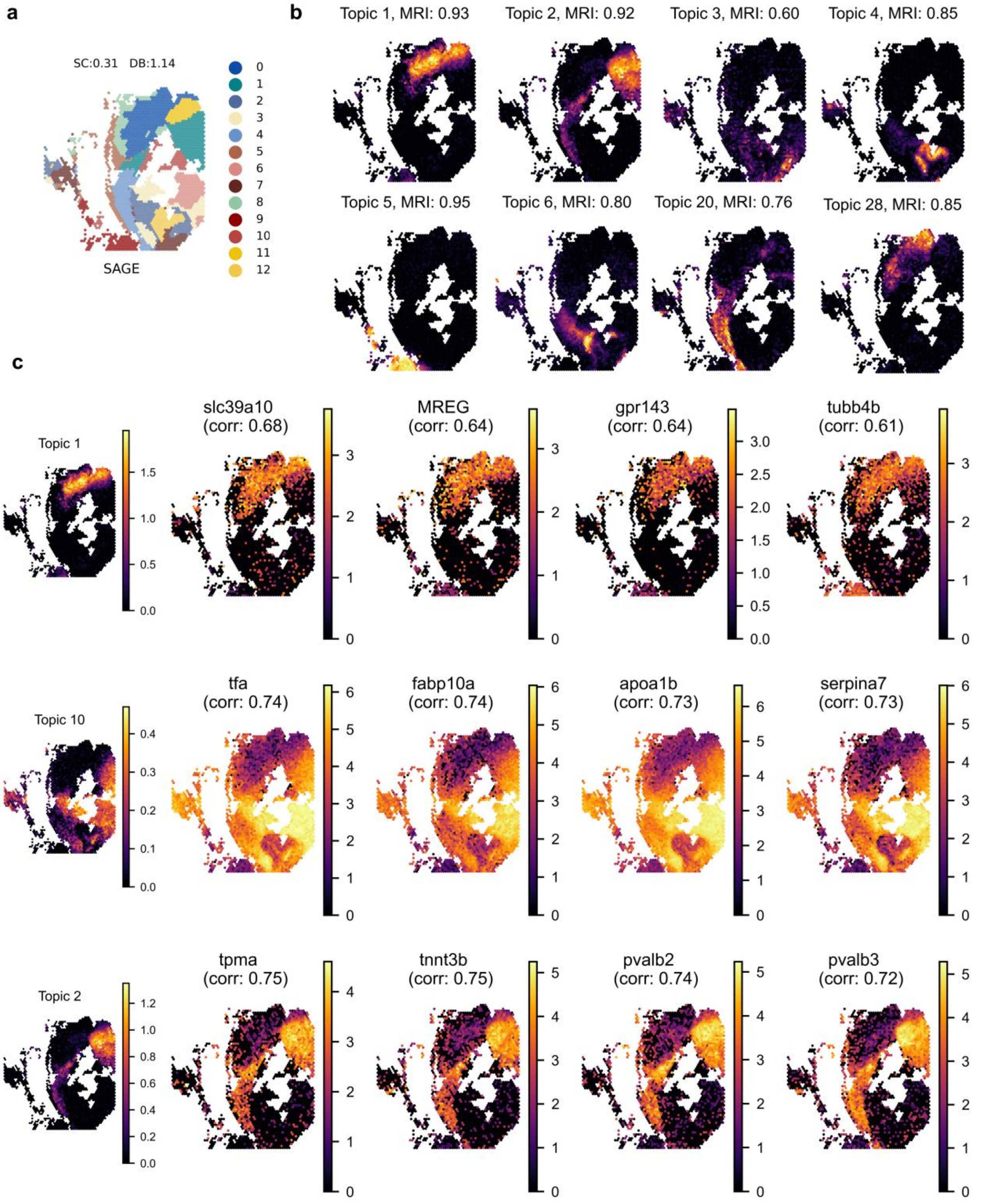

Supplementary Fig. 44 Spatial domain analysis and identification of gene co-expression topics by SAGE in zebrafish melanoma sample C. **(a)** Visualization of spatial domain segmentation results for zebrafish melanoma sample C obtained using SAGE. **(b)** Gene co-expression topics identified by SAGE show distinct spatial expression patterns. The spatial variability significance for each topic is quantified using the Moran's I score (MRI). **(c)** Spatial distributions of Topics 1, 2, and 10, along with their associated high-expression genes. The correlation (corr.) values represent Pearson correlation coefficients between topic probability maps and corresponding gene expression profiles.

Supplementary Figure 45

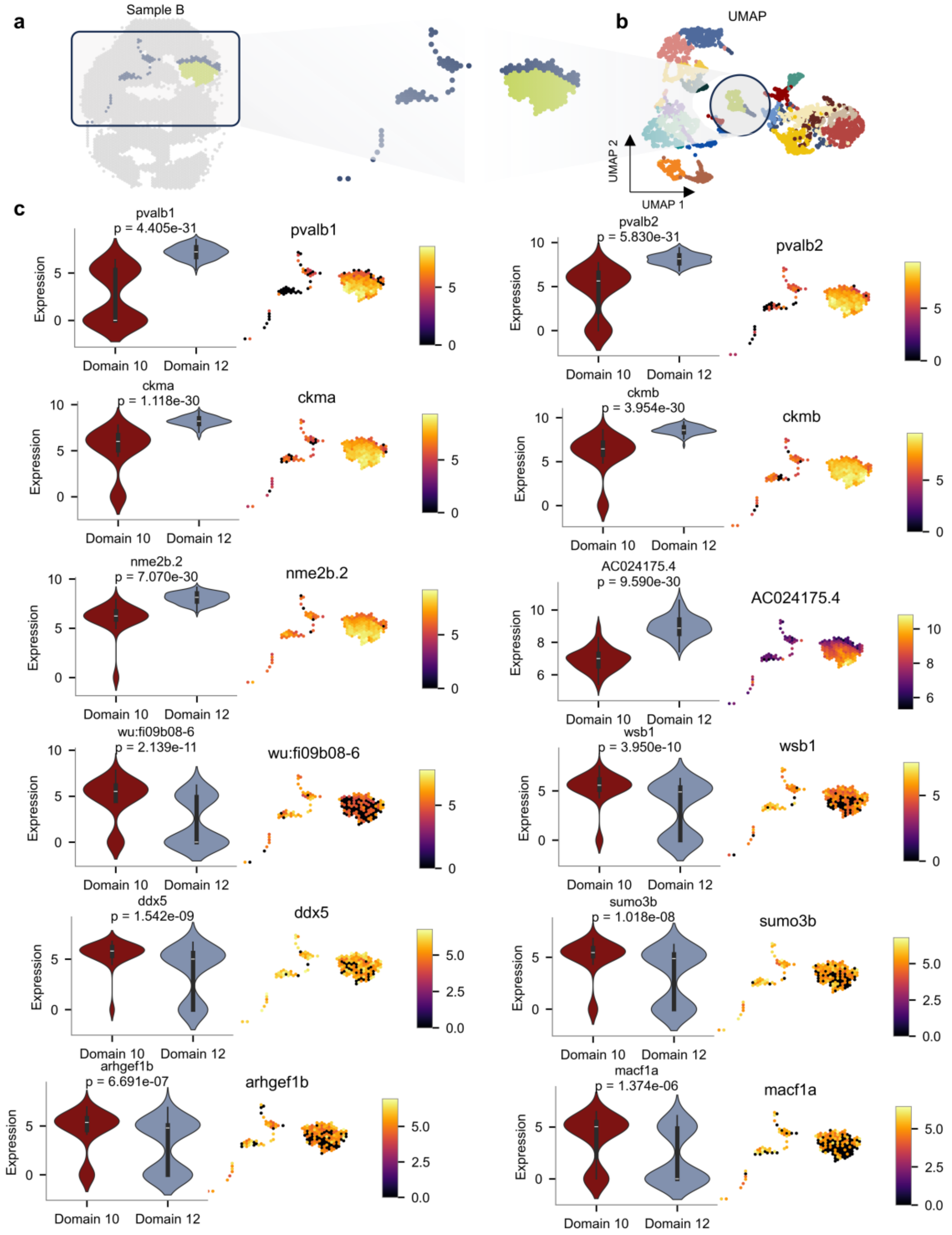

Supplementary Fig. 45 Identification of interface domains and spatially enriched genes in zebrafish melanoma sample B using SAGE. **(a)** Interface domains identified by SAGE in zebrafish melanoma sample B, revealing transitional boundaries between tumor and normal muscle tissue regions. **(b)** UMAP visualization based on spatial domain segmentation results from SAGE, with colors corresponding to different spatial domains. **(c)** Spatial visualization of significantly upregulated genes in Domain 12 and Domain 10. Genes enriched in Domain 12: *pvalb1* (p = 4.405e-31), *pvalb2* (p = 5.830e-31), *ckma* (p = 1.118e-30), *ckmb* (p = 3.954e-30), *nme2b.2* (p = 7.070e-30), *AC024175.4* (p = 9.590e-30). Genes enriched in Domain 10: *wu:fi09v08-6* (p = 2.139e-11), *wsb1* (p = 3.590e-10), *ddx5* (p = 1.542e-09), *sumo3b* (p = 1.018e-08), *arhgef1b* (p = 6.691e-07), *macf1a* (p = 1.374e-06). Source data are provided as a Source Data file.

Supplementary Figure 46

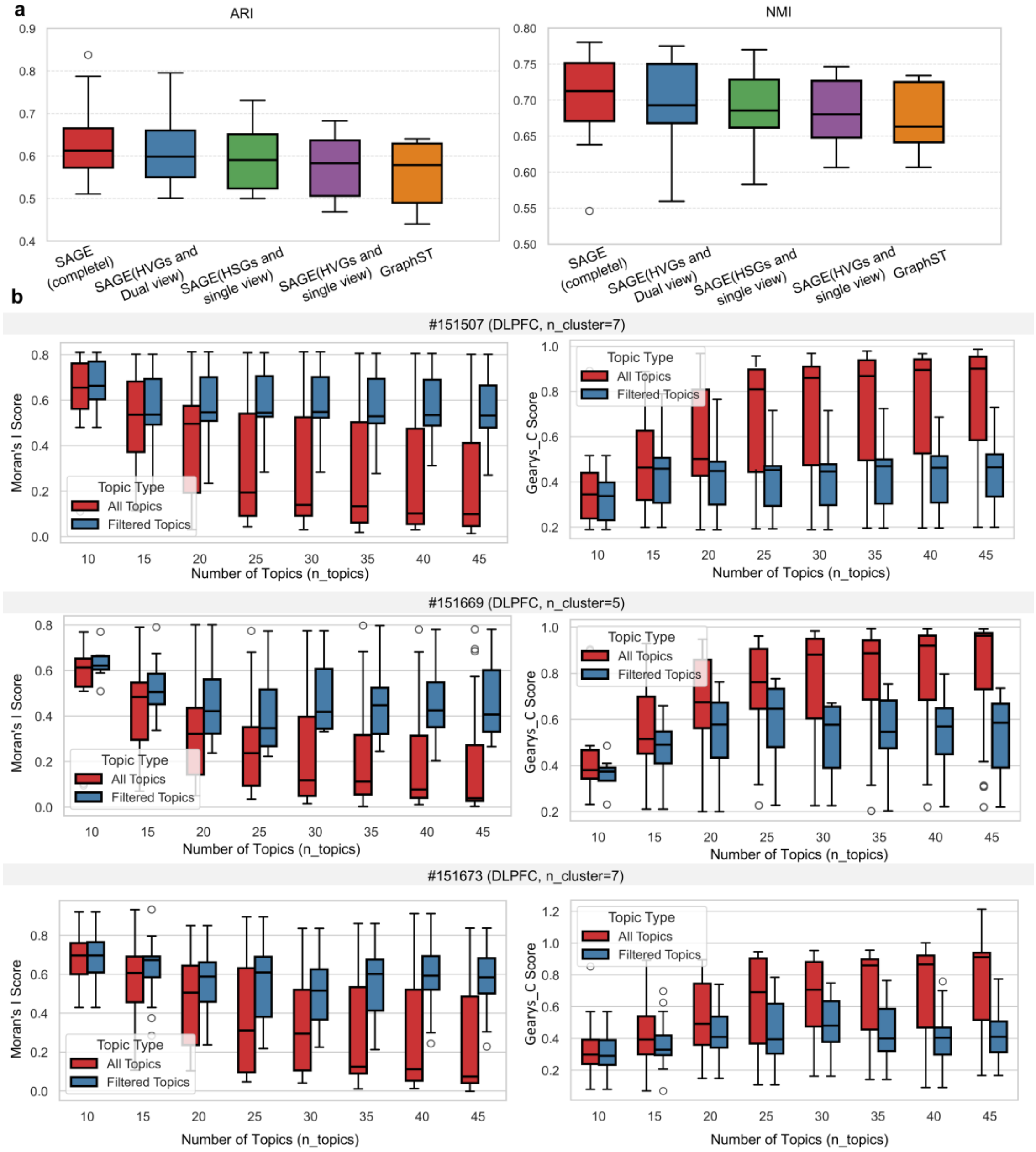

Supplementary Fig. 46 Ablation analysis of the STAIG framework and evaluation of topic selection stability. **(a)** Box plots of ARI and NMI for five model variants on the DLPFC dataset, designed to assess the contribution of the HSG-based gene selection strategy and the dual-view feature fusion module. From left to right, the configurations are: 1) the complete STAIG model with all components enabled; 2) SAGE without HSG, replaced by HVG selection while retaining the dual-view design; 3) SAGE with HSG retained but replacing the dual-view with a single-view structure; 4) a minimal SAGE version without HSG or dual-view; and 5) the baseline method GraphST. In each box plot, the center line indicates the median, box limits indicate the upper and lower quartiles, black dots represent scores from individual tissue slices, and whiskers span 1.5× the interquartile range. (**b**) Box plots of MRI and GC scores under different numbers of NMF topics (10, 15, 20, 25, 30, 35, 40, 45) on three representative tissue sections. The red boxes denote results using all topics, while the blue boxes represent those selected by the SAGE strategy. Box plot elements follow the same format as in (a). From top to bottom, the DLPFC slides shown are: 151507 (number of clusters = 7), 151669 (clusters = 5), and 151673 (clusters = 7).

Supplementary Figure 47

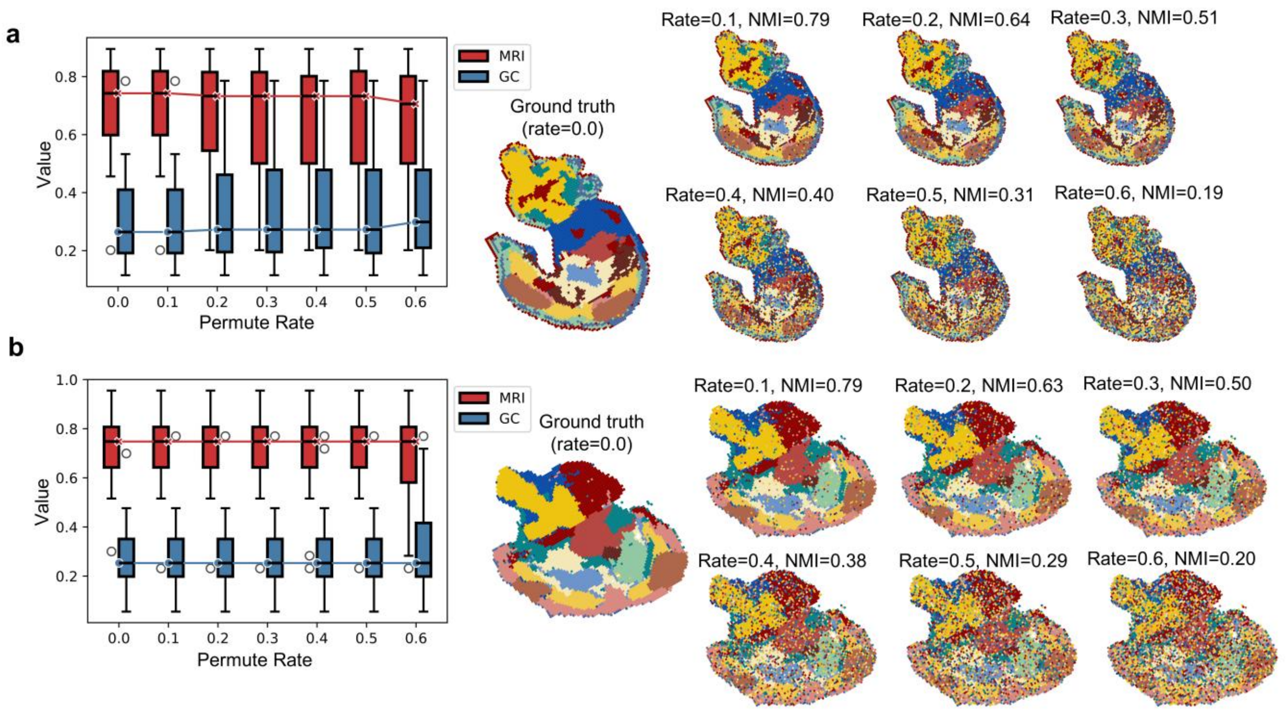

Supplementary Fig. 47 Effect of pre-clustering accuracy on the robustness of topic selection in the SAGE framework. **(a)** Boxplots showing the performance of selected topics under varying permutation rates (0.1, 0.2, 0.3, 0.4, 0.5, and 0.6) in the Data15 mouse embryo dataset, evaluated using Moran's *I* and Geary's C scores. Red represents Moran's *I*, and blue represents Geary's C. The center line of each box indicates the median; box edges represent the interquartile range (IQR); whiskers extend to 1.5× IQR, and black dots indicate individual slice scores. **(b)** Results on the Data16 mouse embryo dataset under the same permutation settings, using the same evaluation and visualization scheme as in (a).

Supplementary Figure 48

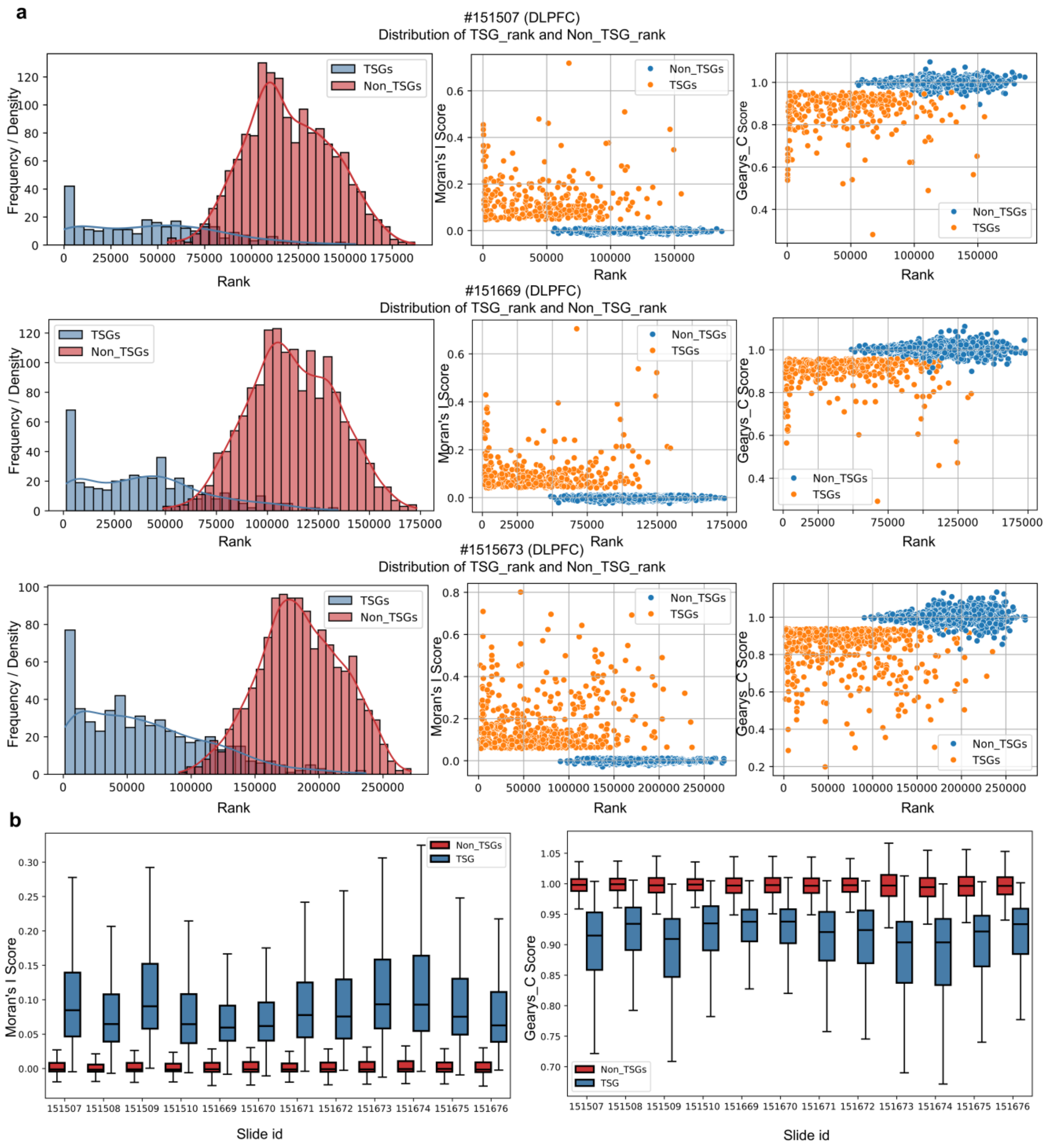

Supplementary Fig. 48 Spatial specificity analysis of TSGs and Non-TSGs in the DLPFC dataset. **(a)** Distribution of TSGs and Non-TSGs ranked by spatial specificity across three representative slices. The x-axis represents rank, and the y-axis shows frequency density. The distributions of both gene sets on the spatial specificity metrics Moran's *I* and Geary's C are shown (yellow for TSGs, blue for Non-TSGs). The slices from top to bottom are: 151507 (number of clusters = 7), 151669 (number of clusters = 5), and 151673 (number of clusters = 7). **(b)** Boxplots summarizing Moran's *I* (left) and Geary's C (right) scores of TSGs and Non-TSGs across all DLPFC slices. The x-axis denotes Slide IDs, with red representing Non-TSGs and blue representing TSGs. In the boxplots, the center line indicates the median, box edges represent the interquartile range, individual points correspond to scores of each slice, and whiskers extend to 1.5 times the interquartile range.

Supplementary Figure 49

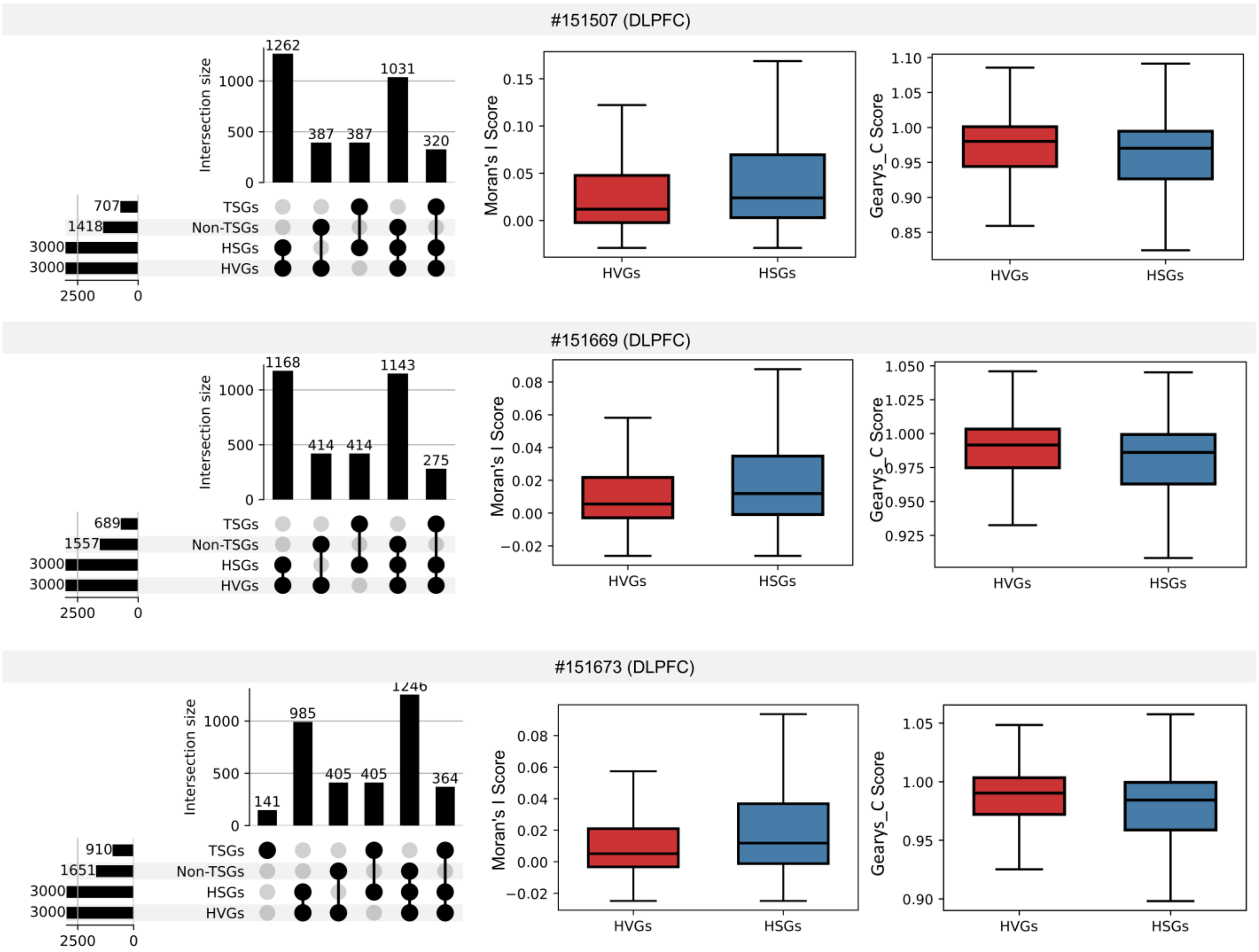

Supplementary Fig. 49 Intersection analysis of TSGs, Non-TSGs, HSGs, and HVGs in the DLPFC dataset and their performance on MRI and GC metrics. An UpSet plot is used to visualize the intersections and unique gene sets among TSGs, Non-TSGs, HSGs, and HVGs, providing a clear overview of their overlaps and distinctions. Additionally, boxplots compare the performance of HVGs and the HSGs on Moran's *I* and Geary's C metrics. In the boxplots, red indicates HVGs and blue indicates HSGs. The center line represents the median, box edges denote the interquartile range, black dots correspond to scores from individual slices, and whiskers represent values within 1.5 times the interquartile range. The slices are ordered from top to bottom as follows: 151507, 151669, and 151673.

Supplementary Figure 50

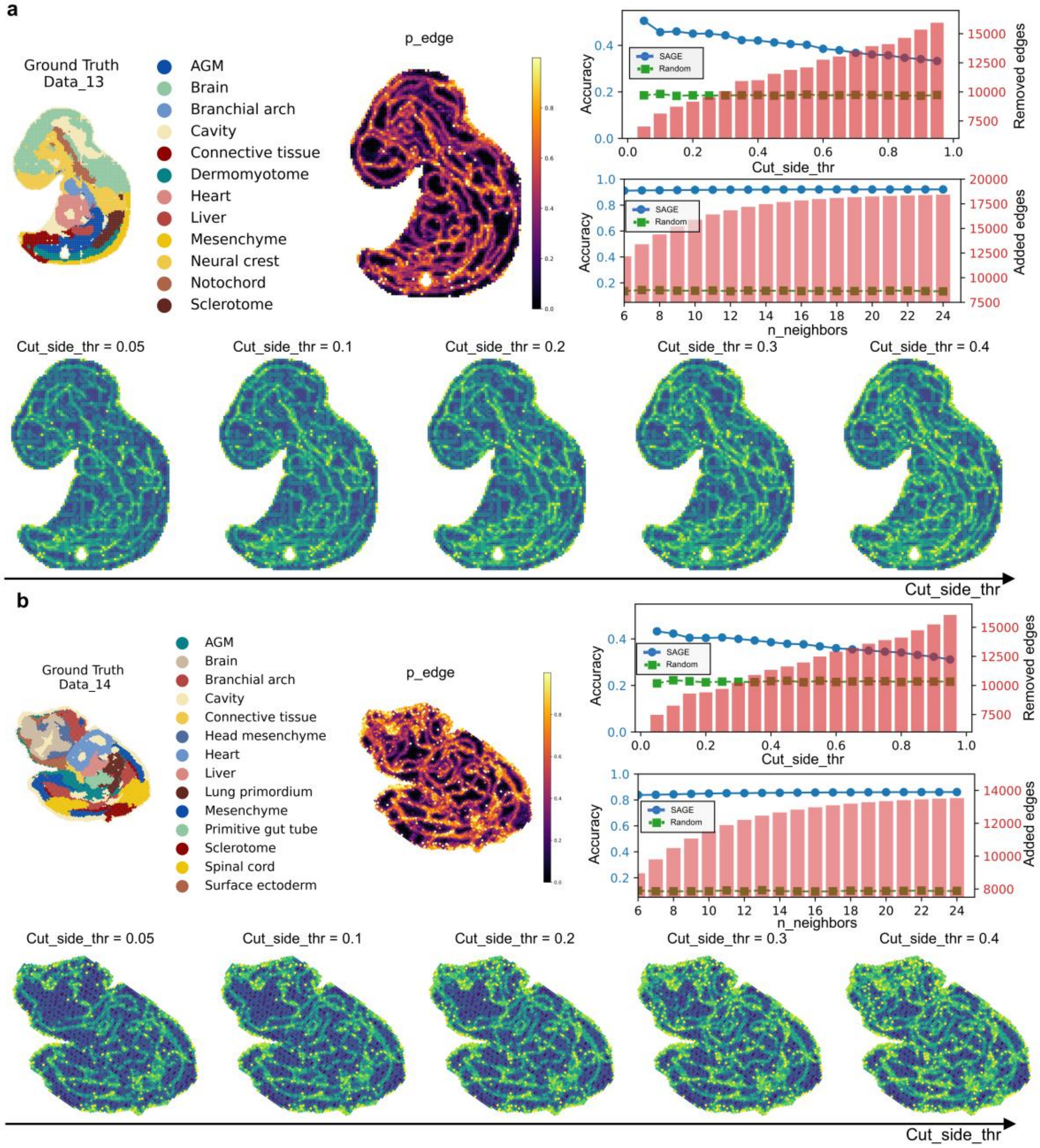

Supplementary Fig. 50 Accuracy analysis of graph topology augmentation and boundary visualization under edge removal and addition strategies on the Slide platform. **(a)** Results on the Data13 mouse embryo dataset. The left panel shows manual annotations of spatial domains along with the boundary probability map (brighter regions indicate a higher likelihood of boundary nodes). The upper right panel displays two dual-axis plots: the top plot shows edge removal accuracy (left axis, line chart; blue for the SAGE strategy, green for the random strategy) and the corresponding number of removed edges (right axis, bar chart); the bottom plot shows edge addition accuracy and the number of added edges under varying neighborhood size constraints, using the same color and axis conventions. The bottom row presents heatmaps of nodes involved in edge removal under different thresholds (0.05, 0.1, 0.2, 0.3, 0.4), where brighter colors indicate nodes with more removed edges. **(b)** Results on the Data14 mouse embryo dataset, following the same structure as in (a). The left panel shows manual annotations and boundary probability maps; the upper right panel compares edge removal accuracy and edge counts; the lower right panel illustrates edge addition accuracy and counts; and the bottom panel visualizes edge removal intensity under varying thresholds.

Supplementary Figure 51

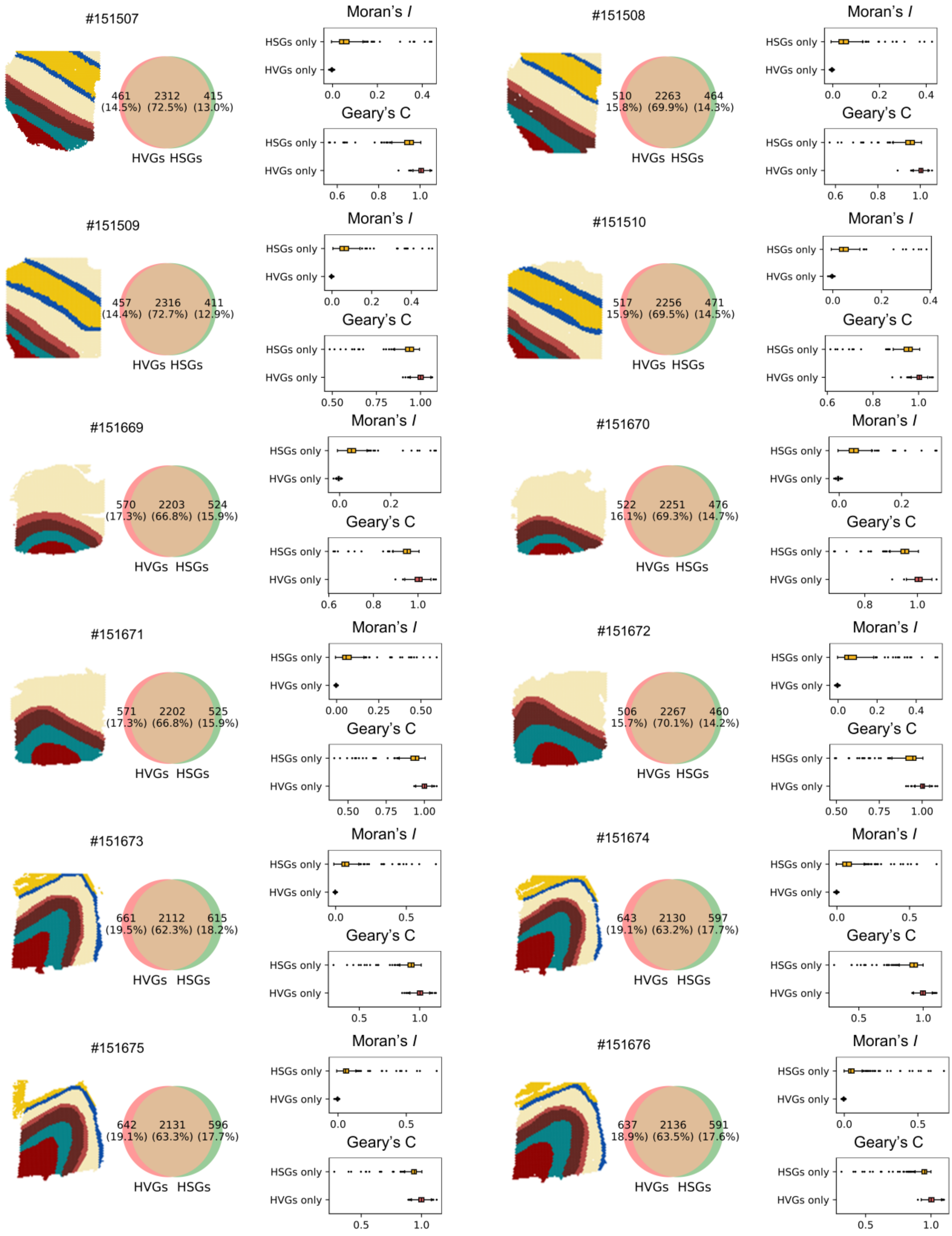

Supplementary Fig. 51 Comparison of HVGs and HSGs (by SAGE) gene selection strategies on the DLPFC dataset. Across 12 sections (151507–151673) of the DLPFC dataset, differences between genes selected by HVGs and those identified by the SAGE-based HSG strategy were compared. For each section, four panels are shown from top to

bottom and left to right: (1) manually annotated tissue domains (ground truth); (2) Venn diagram showing the overlap and distinction between HVG and HSG gene sets; (3–4) horizontal boxplots of Moran's I and Geary's C metrics computed for the non-overlapping gene subsets ("HVGs only" and "HSGs only"), where higher Moran's I and lower Geary's C values indicate stronger spatial autocorrelation.

Supplementary Figure 52

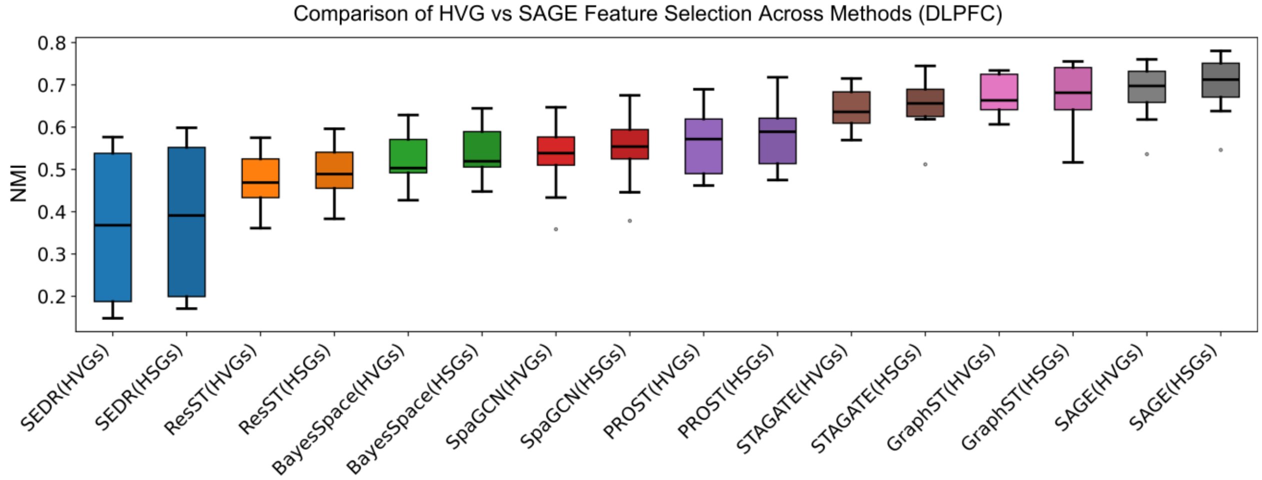

Supplementary Fig. 52 Comparison of HVGs vs SAGE feature selection across spatial analysis methods on the DLPFC dataset. Eight spatial transcriptomics methods (SEDR, BayesSpace, ResST, SpaGCN, PROST, STAGATE, GraphST, and SAGE) were evaluated on the DLPFC dataset using either HVGs or SAGE-based feature selection. Each method is represented by two adjacent boxplots, with lighter colors for HVGs and darker tones for SAGE. The y-axis denotes the Normalized Mutual Information (NMI) between the predicted spatial domains and manual annotations. Overall, SAGE-based feature selection yields higher NMI values for most methods, suggesting improved spatial domain delineation and feature representation.

Supplementary Figure 53

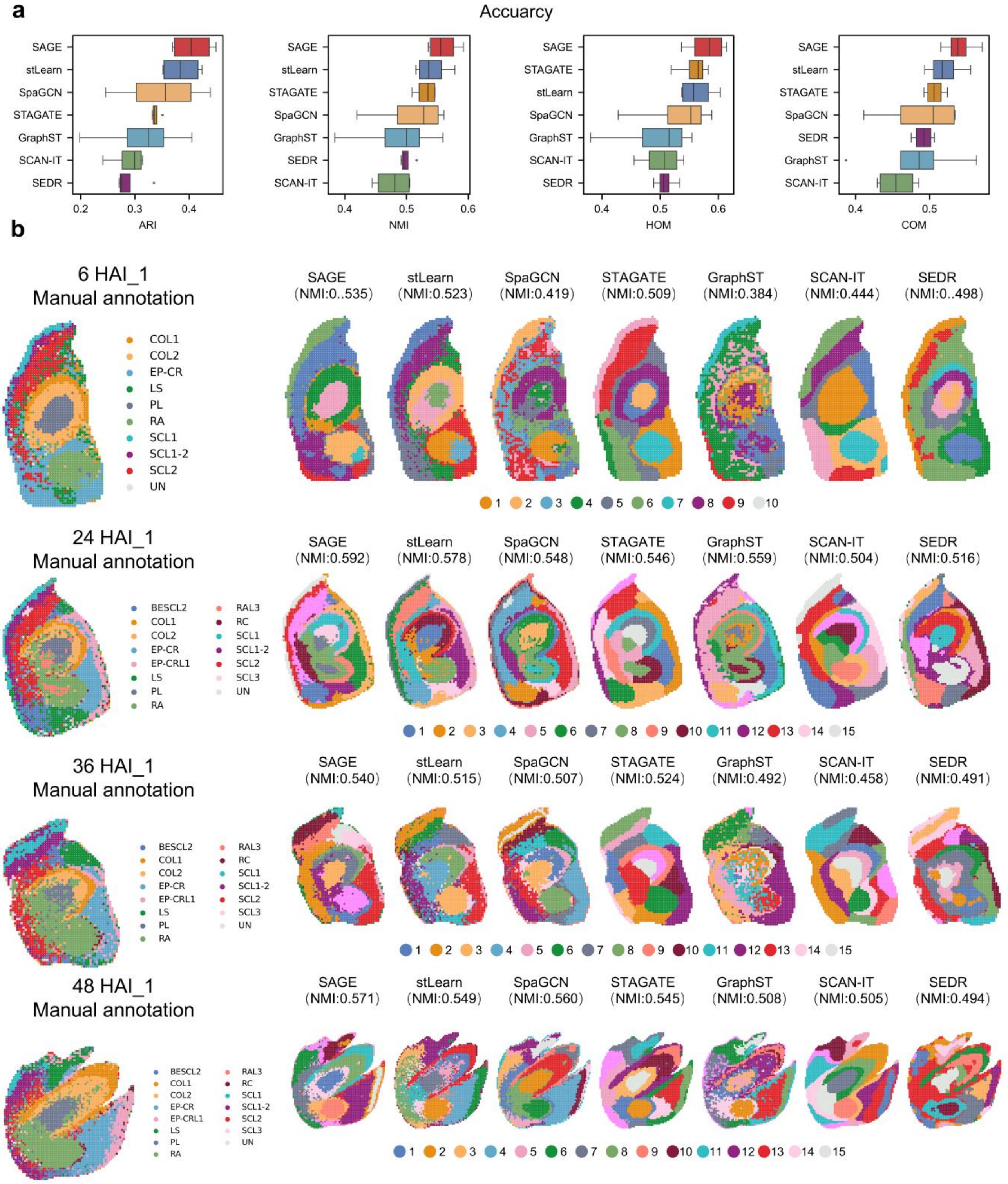

Supplementary Fig. 53 Comparison of method performance and spatial domain identification in rice embryonic cells during seed germination. (a) Boxplots of ARI, NMI, HOM, and COM for various methods, including SAGE, stLearn, SpaGCN, STAGATE, GraphST, SCAN-IT, and SEDR. SAGE outperforms others, particularly in NMI and HOM. (b) Spatial domain segmentation at 6, 24, 36, and 48 HAI. Manual annotations (right) serve as the reference. SAGE consistently provides the most accurate spatial partitioning, with higher NMI values compared to other methods, especially in early germination stages.

Supplementary Figure 54

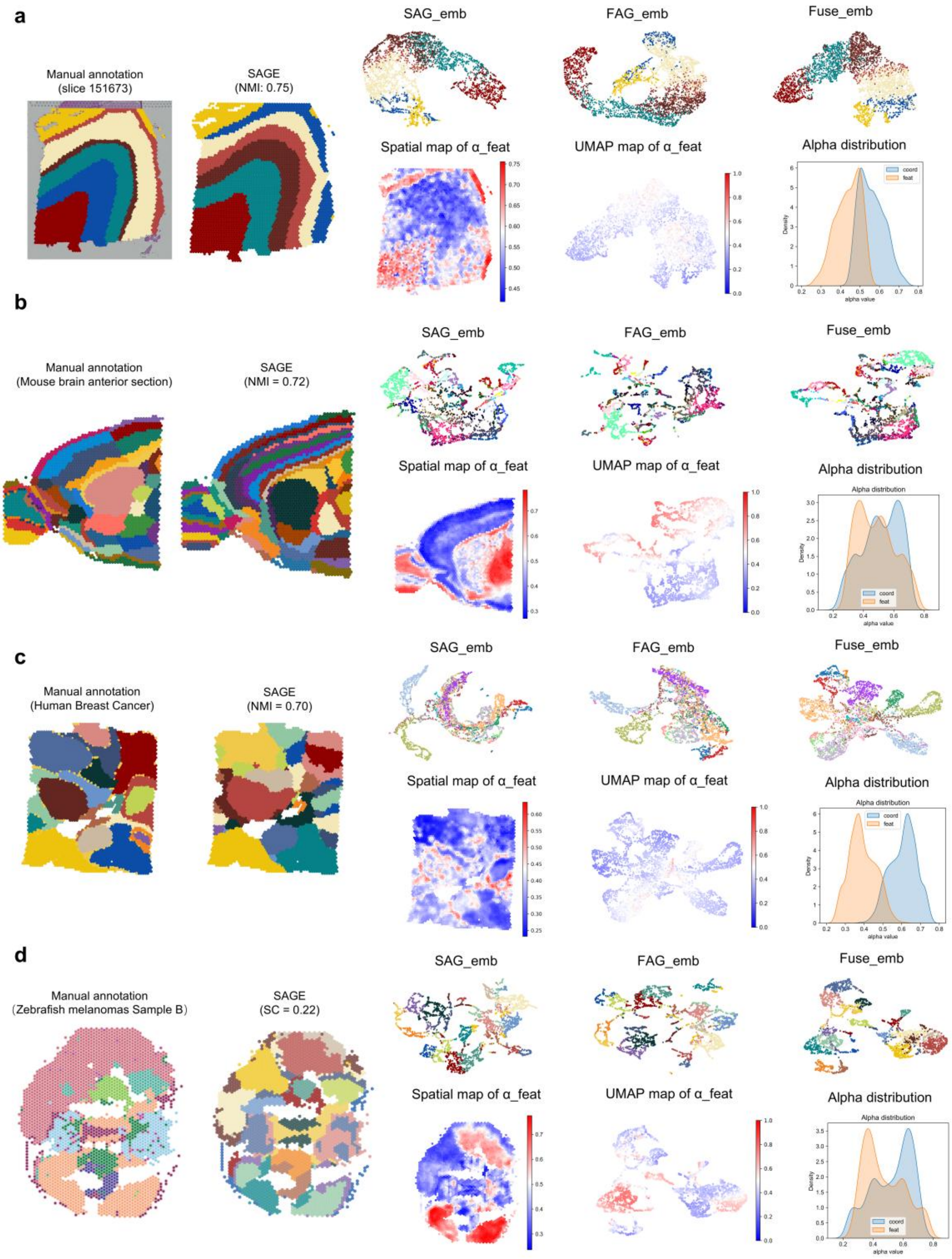

Supplementary Fig. 54 Dual-view embeddings and attention weights for four spatial transcriptomics datasets. (a–d) DLPFC slice 151673, mouse brain anterior section, human breast cancer, and zebrafish melanoma sample B. For each dataset, panels show manual anatomical annotations, SAGE-inferred spatial domains with clustering accuracy (NMI for annotated datasets or SC for zebrafish melanoma), UMAP embeddings from the spatial view (SAG_emb), feature view (FAG_emb), and fused view (Fuse_emb), as well as spatial and UMAP visualizations and distributions of the feature-view attention weight α_{feat} (with $\alpha_{\text{coord}} = 1 - \alpha_{\text{feat}}$), highlighting how SAGE integrates spatial and transcriptional information through dual-view fusion.

Supplementary Tables 1-28

Supplementary Table 1

Basic information about the datasets used in this study.

DataID	n_Data	Spatial technology	Size/Radius	n_Spots mean	n_Genes mean	Sparsity of examined data	Description	local reassignment
Data1~Data12	12	10× Visium	55 µm	3384	33538	0.94	DLPFC Maynard et al.	yes
Data13~Data21	9	Stereo-Seq	25 µm	5913	24238	0.87	Mouse embryo Chen et al.	yes
Data22~Data24	3	BaristaSeq	≤ 1 cell	1690	79	0.81	Mouse primary cortex Chen et al.	no
Data25~Data29	5	MERFISH	≤ 1 cell	5557	155	0.58	Hypothalamic preoptic region Chen et al.	no
Data30	1	osmFISH	≤ 1 cell	4839	33	0.31	Mouse somatosensory cortex Codeluppi et al.	no
Data31~Data33	3	STARmap	≤ 1 cell	1053	166	0.7	Mouse medial prefrontal cortex Wang et al.	no
Data34	1	STARmap*	≤ 1 cell	1207	1020	0.76	Mouse visual cortex Wang et al.	no
Data35	1	10× Visium	55 µm	3798	36601	0.84	HBC	yes
Data36	1	10× Visium	55 µm	2695	32285	0.81	Mouse Anterior brain section	no
Data37	1	10× Visium	55 µm	3355	32285	0.85	Mouse Posterior brain section	no
Data38	1	10× Visium	55 µm	6050	32285	0.83	Combined Mouse anterior and posterior brain sections	no
Data39	1	10× Visium	55 µm	2702	32285	0.81	Mouse Coronal brain section	no
Data40	1	10× Visium	55 µm	2179	32268	0.94	Zebrafish melanoma sample A (Hunter et al.)	yes
Data41	1	10× Visium	55 µm	2677	32268	0.94	Zebrafish melanoma sample B (Hunter et al.)	yes
Data42	1	10× Visium	55 µm	2425	32268	0.97	Zebrafish melanoma sample C (Hunter et al.)	yes
Data43	1	Stereo-Seq	25 µm	2223	23044	0.97	Rice embryonic sample 6 HAI_1 (Yao et al.)	no
Data44	1	Stereo-Seq	25 µm	2588	25320	0.98	Rice embryonic sample 24 HAI_1 (Yao et al.)	no
Data45	1	Stereo-Seq	25 µm	2669	24824	0.97	Rice embryonic sample 36 HAI_1 (Yao et al.)	no
Data46	1	Stereo-Seq	25 µm	2950	23824	0.98	Rice embryonic sample 48 HAI_1 (Yao et al.)	no

Supplementary Table 2

Detailed information about the datasets used in the benchmark of this study.

Data Index	Data_ID	Spatial technology	Size/Radius	Spots	Genes	Sparsity of examined data
Data_1	151507	10× Visium	55 μm	4226	33538	0.96
Data_2	151508	10× Visium	55 μm	4384	33538	0.96
Data_3	151509	10× Visium	55 μm	4789	33538	0.96
Data_4	151510	10× Visium	55 μm	4634	33538	0.96
Data_5	151669	10× Visium	55 μm	3661	33538	0.95
Data_6	151670	10× Visium	55 μm	3498	33538	0.95
Data_7	151671	10× Visium	55 μm	4110	33538	0.94
Data_8	151672	10× Visium	55 μm	4015	33538	0.95
Data_9	151673	10× Visium	55 μm	3639	33538	0.93
Data_10	151674	10× Visium	55 μm	3673	33538	0.92
Data_11	151675	10× Visium	55 μm	3592	33538	0.95
Data_12	151676	10× Visium	55 μm	3460	33538	0.94
Data_13	E9.5_E1S1.MOSTA	Stereo-Seq	≤ 1 cell	5913	25568	1
Data_14	E9.5_E2S1.MOSTA	Stereo-Seq	≤ 1 cell	5292	23756	1
Data_15	E9.5_E2S2.MOSTA	Stereo-Seq	≤ 1 cell	4356	24107	0.99
Data_16	E9.5_E2S3.MOSTA	Stereo-Seq	≤ 1 cell	5059	24238	0.99
Data_17	E9.5_E2S4.MOSTA	Stereo-Seq	≤ 1 cell	5797	23398	0.98
Data_18	E10.5_E1S1.MOSTA	Stereo-Seq	≤ 1 cell	18408	25201	0.98
Data_19	E10.5_E1S2.MOSTA	Stereo-Seq	≤ 1 cell	18647	25544	0.98
Data_20	E10.5_E1S3.MOSTA	Stereo-Seq	≤ 1 cell	18670	25647	0.98
Data_21	E10.5_E2S1.MOSTA	Stereo-Seq	≤ 1 cell	8494	22385	0.98
Data_22	Slice_1	BaristaSeq	≤ 1 cell	1525	79	0.81
Data_23	Slice_2	BaristaSeq	≤ 1 cell	2042	79	0.82
Data_24	Slice_3	BaristaSeq	≤ 1 cell	1690	79	0.86
Data_25	MERFISH_0.04	MERFISH	≤ 1 cell	5488	155	0.58
Data_26	MERFISH_0.09	MERFISH	≤ 1 cell	5557	155	0.57
Data_27	MERFISH_0.14	MERFISH	≤ 1 cell	5926	155	0.57
Data_28	MERFISH_0.19	MERFISH	≤ 1 cell	5803	155	0.58
Data_29	MERFISH_0.24	MERFISH	≤ 1 cell	5543	155	0.59
Data_30	osmfish	osmFISH	≤ 1 cell	4839	33	0.31
Data_31	20180417_BZ5_control	STARmap	≤ 1 cell	1049	166	0.69
Data_32	20180419_BZ9_control	STARmap	≤ 1 cell	1053	166	0.77
Data_33	20180424_BZ14_control	STARmap	≤ 1 cell	1088	166	0.7
Data_34	STARmap_20180505_BY3_1k	STARmap*	≤ 1 cell	1207	1020	0.77

Supplementary Table 3

Comparison of SAGE with 16 state-of-the-art spatial transcriptomics domain analysis methods in terms of NMI

Data Name	STAMP	GASTON	STAGATE	stLearn	PROST	SEDR	GraphST	BASS	BayesSpace	louvain	SpaGCN (HE)	SCAN-IT	CCST	SpaGCN	leiden	SpaceFlow	conST	SAGE
Data_1	0.46	0.63	0.52	0.61	0.66	0.51	0.69	0.65	0.63	0.21	0.51	0.66	0.57	0.55	0.22	0.63	0.50	**0.71**
Data_2	0.46	0.62	0.47	0.52	0.56	0.48	0.56	0.62	0.60	0.22	0.46	0.62	0.54	0.52	0.23	0.52	0.47	**0.68**
Data_3	0.45	0.65	0.55	0.61	0.62	0.58	0.63	0.63	0.60	0.34	0.52	0.65	0.55	0.55	0.29	0.52	0.53	**0.70**
Data_4	0.50	0.59	0.51	0.50	0.61	0.53	0.59	0.57	0.56	0.24	0.50	0.61	0.49	0.54	0.25	0.58	0.49	**0.65**
Data_5	0.39	0.58	0.36	0.51	0.44	0.58	0.59	0.59	0.61	0.16	0.39	0.38	0.42	0.48	0.16	0.46	0.53	**0.64**
Data_6	0.33	0.56	0.40	0.35	0.49	0.53	0.47	0.46	**0.55**	0.16	0.39	0.50	0.45	0.44	0.16	0.43	0.51	**0.55**
Data_7	0.39	0.57	0.46	0.58	0.68	0.66	0.68	0.68	0.69	0.24	0.54	0.55	0.55	0.62	0.24	0.50	0.62	**0.78**
Data_8	0.34	0.65	0.51	0.49	0.71	0.47	0.67	0.65	0.60	0.23	0.56	0.65	0.57	0.64	0.22	0.55	0.42	**0.76**
Data_9	0.46	0.72	0.57	0.55	0.62	0.63	0.70	0.71	0.69	0.30	0.48	0.60	0.48	0.49	0.31	0.45	0.67	**0.75**
Data_10	0.41	0.71	0.50	0.55	0.48	0.57	0.53	0.58	0.48	0.34	0.47	0.52	0.50	0.50	0.32	0.43	0.59	**0.76**
Data_11	0.46	0.55	0.55	0.57	0.61	0.54	0.64	0.61	0.68	0.35	0.47	0.51	0.50	0.53	0.34	0.47	0.40	**0.71**
Data_12	0.46	0.73	0.46	0.54	0.62	0.49	0.61	0.56	0.53	0.32	0.49	0.60	0.52	0.51	0.32	0.40	0.54	**0.73**
Data_13	0.46	0.45	NA	NA	0.53	0.51	0.56	0.44	NA	0.28	NA	0.53	NA	0.56	0.27	0.51	0.49	**0.57**
Data_14	0.53	0.43	NA	NA	0.56	0.53	0.55	0.53	NA	0.31	NA	0.49	NA	**0.59**	0.34	0.22	0.49	0.58
Data_15	0.55	0.47	NA	NA	0.58	0.58	0.58	0.58	NA	0.27	NA	0.51	NA	0.61	0.31	0.47	NA	**0.62**
Data_16	0.59	0.47	NA	NA	0.61	**0.67**	0.62	0.62	NA	0.37	NA	0.58	NA	0.65	0.37	0.44	0.58	0.65
Data_17	0.53	0.38	NA	NA	0.56	0.54	0.58	0.55	NA	0.40	NA	0.51	NA	**0.59**	0.39	0.26	0.49	**0.59**
Data_18	0.49	0.38	NA	NA	0.53	0.49	NA	0.47	NA	0.35	NA	0.45	NA	0.57	0.37	0.31	NA	**0.60**
Data_19	0.42	0.37	NA	NA	0.42	0.42	NA	0.39	NA	0.23	NA	0.41	NA	0.46	0.23	0.41	NA	**0.57**
Data_20	0.49	0.41	NA	NA	0.53	NA	NA	0.54	NA	0.29	NA	0.50	NA	0.55	0.33	0.37	NA	**0.60**
Data_21	0.58	0.43	NA	NA	0.58	0.63	0.60	NA	NA	0.30	NA	0.56	NA	0.64	0.33	0.35	0.53	**0.64**
Data_22	0.38	0.74	0.35	NA	0.67	0.04	0.35	0.71	NA	0.02	NA	0.73	0.63	0.31	0.06	0.69	0.04	**0.76**
Data_23	0.43	0.69	0.50	NA	0.75	0.06	0.42	0.82	NA	0.10	NA	0.73	0.63	0.27	0.10	0.72	0.05	**0.83**
Data_24	0.26	0.64	0.29	NA	0.45	0.05	0.34	NA	NA	0.06	NA	**0.70**	0.62	0.19	0.08	0.63	0.03	0.68
Data_25	0.20	0.42	0.09	NA	0.45	0.09	0.21	0.43	NA	0.15	NA	0.53	0.45	0.19	0.18	0.41	0.10	**0.56**
Data_26	0.31	0.39	0.30	NA	0.49	0.14	0.35	0.50	NA	0.17	NA	0.56	0.44	0.23	0.18	**0.60**	0.09	0.56
Data_27	0.34	0.43	0.29	NA	0.51	0.21	0.35	0.53	NA	0.17	NA	**0.57**	0.47	0.22	0.17	0.51	0.11	0.54
Data_28	0.33	0.51	0.20	NA	0.57	0.10	0.33	0.57	NA	0.17	NA	0.58	0.45	0.22	0.17	0.57	0.12	**0.63**
Data_29	0.32	0.55	0.14	NA	0.57	0.16	0.34	0.57	NA	0.17	NA	**0.65**	0.52	0.23	0.18	0.60	0.12	0.58
Data_30	0.48	0.49	0.52	NA	0.64	0.19	0.54	0.59	NA	0.32	NA	0.52	0.49	0.25	0.32	0.59	0.14	**0.69**
Data_31	0.40	0.55	0.60	NA	0.66	0.06	0.51	**0.80**	NA	0.09	NA	0.69	0.32	0.31	0.10	0.67	0.05	0.77
Data_32	0.37	0.63	0.45	NA	0.54	0.19	0.36	0.57	NA	0.06	NA	0.56	0.41	0.33	0.06	0.53	0.09	**0.62**
Data_33	0.29	0.58	0.56	NA	0.62	0.09	0.42	**0.71**	NA	0.04	NA	0.64	0.33	0.31	0.04	0.61	0.06	**0.71**
Data_34	0.28	0.61	0.40	NA	0.68	0.28	0.50	0.66	NA	0.11	NA	0.68	0.57	0.37	0.12	0.66	0.24	**0.72**

*NA in the table indicates that SDMbench did not provide the NMI metric results for that method on the given dataset, or that the method (SpaGCN (HE)) is not applicable to the specific spatial transcriptomics technology dataset.

Supplementary Table 4

Normalized rank scores of SAGE and 16 baseline methods based on NMI, reflecting performance ranks (0–1 scale, higher is better) across 34 datasets.

Data Name	STAMP	GASTON	STAGATE	stLearn	PROST	SEDR	GraphST	BASS	BayesSpace	louvain	SpaGCN (HE)	SCAN-IT	CCST	SpaGCN	leiden	SpaceFlow	conST	SAGE
Data_1	0.12	0.71	0.35	0.53	0.88	0.24	0.94	0.76	0.59	0	0.29	0.82	0.47	0.41	0.06	0.65	0.18	**1**
Data_2	0.12	0.94	0.29	0.41	0.65	0.35	0.71	0.82	0.76	0	0.18	0.88	0.59	0.47	0.06	0.53	0.24	**1**
Data_3	0.12	0.88	0.35	0.65	0.71	0.53	0.82	0.76	0.59	0.06	0.18	0.94	0.41	0.47	0	0.24	0.29	**1**
Data_4	0.24	0.82	0.41	0.29	0.88	0.47	0.76	0.65	0.59	0	0.35	0.94	0.12	0.53	0.06	0.71	0.18	**1**
Data_5	0.24	0.76	0.12	0.59	0.41	0.71	0.82	0.88	0.94	0.06	0.29	0.18	0.35	0.53	0	0.47	0.65	**1**
Data_6	0.12	**1**	0.29	0.18	0.65	0.82	0.59	0.53	0.94	0	0.24	0.71	0.47	0.41	0.06	0.35	0.76	0.88
Data_7	0.12	0.47	0.18	0.53	0.76	0.71	0.88	0.82	0.94	0	0.29	0.35	0.41	0.59	0.06	0.24	0.65	**1**
Data_8	0.12	0.76	0.35	0.29	0.94	0.24	0.88	0.82	0.59	0.06	0.47	0.71	0.53	0.65	0	0.41	0.18	**1**
Data_9	0.18	0.94	0.47	0.41	0.59	0.65	0.82	0.88	0.76	0	0.24	0.53	0.29	0.35	0.06	0.12	0.71	**1**
Data_10	0.12	0.94	0.53	0.71	0.29	0.76	0.65	0.82	0.35	0.06	0.24	0.59	0.47	0.41	0	0.18	0.88	**1**
Data_11	0.18	0.59	0.65	0.71	0.82	0.53	0.88	0.76	0.94	0.06	0.29	0.41	0.35	0.47	0	0.24	0.12	**1**
Data_12	0.18	0.94	0.24	0.59	0.88	0.35	0.82	0.71	0.53	0	0.29	0.76	0.47	0.41	0.06	0.12	0.65	**1**
Data_13	0.33	0.25	NA	NA	0.67	0.5	0.83	0.17	NA	0.08	NA	0.75	NA	0.92	0	0.58	0.42	**1**
Data_14	0.5	0.25	NA	NA	0.83	0.58	0.75	0.67	NA	0.08	NA	0.42	NA	**1**	0.17	0	0.33	0.92
Data_15	0.45	0.18	NA	NA	0.73	0.55	0.64	0.82	NA	0	NA	0.36	NA	0.91	0.09	0.27	NA	**1**
Data_16	0.5	0.25	NA	NA	0.58	**1**	0.67	0.75	NA	0	NA	0.33	NA	0.83	0.08	0.17	0.42	0.92
Data_17	0.5	0.08	NA	NA	0.75	0.58	0.83	0.67	NA	0.25	NA	0.42	NA	**1**	0.17	0	0.33	0.92
Data_18	0.6	0.3	NA	NA	0.8	0.7	NA	0.5	NA	0.1	NA	0.4	NA	0.9	0.2	0	NA	**1**
Data_19	0.7	0.2	NA	NA	0.6	0.8	NA	0.3	NA	0.1	NA	0.5	NA	0.9	0	0.4	NA	**1**
Data_20	0.44	0.33	NA	NA	0.67	NA	NA	0.78	NA	0	NA	0.56	NA	0.89	0.11	0.22	NA	**1**
Data_21	0.64	0.27	NA	NA	0.55	0.82	0.73	NA	NA	0	NA	0.45	NA	0.91	0.09	0.18	0.36	**1**
Data_22	0.5	0.93	0.36	NA	0.64	0.14	0.43	0.79	NA	0	NA	0.86	0.57	0.29	0.21	0.71	0.07	**1**
Data_23	0.43	0.64	0.5	NA	0.86	0.07	0.36	0.93	NA	0.14	NA	0.79	0.57	0.29	0.21	0.71	0	**1**
Data_24	0.38	0.85	0.46	NA	0.62	0.08	0.54	NA	NA	0.15	NA	**1**	0.69	0.31	0.23	0.77	0	0.92
Data_25	0.43	0.64	0	NA	0.79	0.07	0.5	0.71	NA	0.21	NA	0.93	0.86	0.36	0.29	0.57	0.14	**1**
Data_26	0.43	0.57	0.36	NA	0.71	0.07	0.5	0.79	NA	0.14	NA	0.86	0.64	0.29	0.21	**1**	0	0.93
Data_27	0.43	0.57	0.36	NA	0.71	0.21	0.5	0.86	NA	0.14	NA	**1**	0.64	0.29	0.07	0.79	0	0.93
Data_28	0.5	0.64	0.29	NA	0.86	0	0.43	0.71	NA	0.21	NA	0.93	0.57	0.36	0.14	0.79	0.07	**1**
Data_29	0.43	0.64	0.07	NA	0.71	0.14	0.5	0.79	NA	0.21	NA	**1**	0.57	0.36	0.29	0.93	0	0.86
Data_30	0.36	0.43	0.64	NA	0.93	0.07	0.71	0.86	NA	0.29	NA	0.57	0.5	0.14	0.21	0.79	0	**1**
Data_31	0.43	0.57	0.64	NA	0.71	0.07	0.5	**1**	NA	0.14	NA	0.86	0.36	0.29	0.21	0.79	0	0.93
Data_32	0.43	**1**	0.57	NA	0.71	0.21	0.36	0.86	NA	0	NA	0.79	0.5	0.29	0.07	0.64	0.14	0.93
Data_33	0.29	0.64	0.57	NA	0.79	0.21	0.5	0.93	NA	0.07	NA	0.86	0.43	0.36	0	0.71	0.14	**1**
Data_34	0.29	0.64	0.43	NA	0.93	0.21	0.5	0.71	NA	0	NA	0.86	0.57	0.36	0.07	0.79	0.14	**1**

*NA indicates missing values due to unavailability of the original metric or method incompatibility with specific datasets. The 'Mean score' row at the bottom represents the average normalized rank score for each method across all applicable datasets. A higher mean score indicates better overall performance under the given evaluation metric.

Supplementary Table 5

Comparison of SAGE with 16 state-of-the-art spatial transcriptomics domain analysis methods in terms of HOM

Data Name	STAMP	GASTON	STAGATE	stLearn	PROST	SEDR	GraphST	BASS	BayesSpace	louvain	SpaGCN (HE)	SCAN-IT	CCST	SpaGCN	leiden	SpaceFlow	conST	SAGE
Data_1	0.46	0.64	0.52	0.59	0.67	0.38	0.67	0.66	0.61	0.20	0.51	0.64	0.58	0.54	0.21	0.75	0.50	**0.72**
Data_2	0.48	0.61	0.49	0.49	0.58	0.36	0.57	0.62	0.60	0.23	0.47	0.64	0.57	0.54	0.23	0.64	0.47	**0.68**
Data_3	0.48	0.67	0.57	0.61	0.67	0.48	0.66	0.66	0.62	0.36	0.54	0.68	0.58	0.58	0.31	0.65	0.56	**0.73**
Data_4	0.53	0.63	0.54	0.51	0.65	0.39	0.62	0.61	0.57	0.25	0.53	0.66	0.52	0.56	0.27	0.72	0.53	**0.62**
Data_5	0.44	0.64	0.41	0.54	0.50	0.51	0.65	0.67	0.67	0.18	0.45	0.51	0.57	0.52	0.18	0.71	0.58	**0.69**
Data_6	0.39	0.60	0.48	0.39	0.57	0.46	0.52	0.54	0.63	0.19	0.46	0.67	0.62	0.51	0.19	0.70	0.60	**0.61**
Data_7	0.41	0.59	0.49	0.58	0.72	0.56	0.72	0.72	0.68	0.25	0.57	0.63	0.68	0.65	0.25	0.74	0.64	**0.76**
Data_8	0.36	0.67	0.52	0.48	0.74	0.38	0.69	0.65	0.59	0.24	0.59	0.72	0.67	0.67	0.23	0.78	0.43	**0.74**
Data_9	0.47	0.72	0.58	0.53	0.64	0.64	0.72	0.73	0.70	0.31	0.49	0.63	0.50	0.51	0.32	0.57	0.69	**0.77**
Data_10	0.42	0.70	0.51	0.55	0.49	0.54	0.52	0.59	0.49	0.33	0.47	0.54	0.52	0.51	0.33	0.52	0.60	**0.74**
Data_11	0.46	0.56	0.55	0.55	0.62	0.44	0.64	0.61	0.67	0.35	0.48	0.52	0.53	0.54	0.34	0.57	0.40	**0.70**
Data_12	0.46	0.73	0.47	0.53	0.62	0.38	0.62	0.56	0.53	0.32	0.50	0.63	0.55	0.52	0.33	0.50	0.55	**0.72**
Data_13	0.48	0.47	NA	NA	0.55	0.54	**0.59**	0.44	NA	0.28	NA	0.55	NA	**0.59**	0.27	0.58	0.51	**0.59**
Data_14	0.54	0.43	NA	NA	0.58	0.52	0.56	0.53	NA	0.29	NA	0.48	NA	**0.60**	0.34	0.25	0.49	0.59
Data_15	0.55	0.47	NA	NA	0.59	0.57	0.58	0.57	NA	0.26	NA	0.51	NA	**0.60**	0.32	0.55	NA	**0.60**
Data_16	0.60	0.48	NA	NA	0.61	**0.68**	0.62	0.63	NA	0.38	NA	0.57	NA	0.65	0.37	0.49	0.58	0.65
Data_17	0.56	0.40	NA	NA	0.58	0.54	**0.59**	0.56	NA	0.40	NA	0.52	NA	**0.59**	0.41	0.30	0.49	**0.59**
Data_18	0.52	0.41	NA	NA	0.58	0.52	NA	0.49	NA	0.37	NA	0.49	NA	0.60	0.40	0.38	NA	**0.64**
Data_19	0.44	0.38	NA	NA	0.44	0.42	NA	0.39	NA	0.23	NA	0.42	NA	0.46	0.24	0.52	NA	**0.57**
Data_20	0.51	0.43	NA	NA	0.55	NA	NA	0.54	NA	0.29	NA	0.50	NA	0.56	0.34	0.46	NA	**0.57**
Data_21	0.60	0.44	NA	NA	0.59	0.64	0.60	NA	NA	0.30	NA	0.58	NA	**0.65**	0.33	0.37	0.53	0.64
Data_22	0.39	0.73	0.36	NA	0.68	0.03	0.35	0.67	NA	0.02	NA	**0.73**	0.66	0.31	0.06	0.75	0.04	0.71
Data_23	0.45	0.70	0.52	NA	0.77	0.05	0.41	0.82	NA	0.11	NA	0.76	0.68	0.28	0.11	0.83	0.05	**0.83**
Data_24	0.27	0.66	0.29	NA	0.46	0.04	0.35	NA	NA	0.07	NA	**0.70**	0.68	0.19	0.08	0.68	0.03	0.68
Data_25	0.20	0.41	0.09	NA	0.47	0.08	0.14	0.35	NA	0.14	NA	0.55	0.53	0.19	0.18	0.35	0.10	0.56
Data_26	0.33	0.39	0.31	NA	0.50	0.12	0.26	0.43	NA	0.16	NA	**0.58**	0.52	0.23	0.17	0.57	0.09	0.55
Data_27	0.36	0.42	0.29	NA	0.51	0.18	0.26	0.44	NA	0.16	NA	**0.60**	0.53	0.21	0.16	0.47	0.12	0.52
Data_28	0.34	0.49	0.20	NA	0.58	0.09	0.21	0.47	NA	0.16	NA	0.59	0.51	0.21	0.16	0.50	0.12	**0.63**
Data_29	0.33	0.55	0.14	NA	0.57	0.12	0.23	0.47	NA	0.16	NA	0.68	0.59	0.22	0.16	0.53	0.12	0.55
Data_30	0.50	0.49	0.55	NA	0.67	0.19	0.55	0.58	NA	0.30	NA	0.52	0.49	0.25	0.30	0.63	0.15	**0.68**
Data_31	0.42	0.58	0.61	NA	0.68	0.06	0.48	**0.81**	NA	0.08	NA	0.74	0.30	0.32	0.10	**0.81**	0.05	0.75
Data_32	0.41	0.57	0.49	NA	0.58	0.19	0.38	0.60	NA	0.05	NA	0.61	0.44	0.36	0.06	**0.67**	0.10	0.56
Data_33	0.32	0.60	0.59	NA	0.68	0.10	0.46	0.73	NA	0.04	NA	**0.79**	0.34	0.34	0.04	0.78	0.07	0.75
Data_34	0.29	0.60	0.40	NA	0.66	0.28	0.51	0.66	NA	0.10	NA	0.67	0.55	0.38	0.11	**0.73**	0.24	0.68

*NA in the table indicates that SDMbench did not provide the HOM metric results for that method on the given dataset, or that the method (SpaGCN (HE)) is not applicable to the specific spatial transcriptomics technology dataset.

Supplementary Table 6

Normalized rank scores of SAGE and 16 baseline methods based on HOM, reflecting performance ranks (0–1 scale, higher is better) across 34 datasets.

Data Name	STAMP	GASTON	STAGATE	stLearn	PROST	SEDR	GraphST	BASS	BayesSpace	louvain	SpaGCN (HE)	SCAN-IT	CCST	SpaGCN	leiden	SpaceFlow	conST	SAGE
Data_1	0.18	0.65	0.35	0.53	0.82	0.12	0.88	0.76	0.59	0	0.29	0.71	0.47	0.41	0.06	**1**	0.24	0.94
Data_2	0.29	0.76	0.35	0.41	0.65	0.12	0.59	0.82	0.71	0	0.18	0.88	0.53	0.47	0.06	0.94	0.24	**1**
Data_3	0.18	0.88	0.35	0.53	0.82	0.12	0.71	0.76	0.59	0.06	0.24	0.94	0.47	0.41	0	0.65	0.29	**1**
Data_4	0.41	0.82	0.47	0.18	0.88	0.12	0.76	0.65	0.59	0	0.29	0.94	0.24	0.53	0.06	**1**	0.35	0.71
Data_5	0.18	0.71	0.12	0.53	0.29	0.41	0.76	0.88	0.82	0.06	0.24	0.35	0.59	0.47	0	**1**	0.65	0.94
Data_6	0.12	0.71	0.35	0.18	0.59	0.29	0.47	0.53	0.88	0	0.24	0.94	0.82	0.41	0.06	**1**	0.65	0.76
Data_7	0.12	0.41	0.18	0.35	0.88	0.24	0.76	0.82	0.71	0.06	0.29	0.47	0.65	0.59	0	0.94	0.53	**1**
Data_8	0.12	0.71	0.35	0.29	0.94	0.18	0.76	0.53	0.47	0.06	0.41	0.82	0.59	0.65	0	**1**	0.24	0.88
Data_9	0.12	0.88	0.47	0.35	0.65	0.59	0.82	0.94	0.76	0	0.18	0.53	0.24	0.29	0.06	0.41	0.71	**1**
Data_10	0.12	0.94	0.41	0.76	0.29	0.71	0.47	0.82	0.24	0	0.18	0.65	0.59	0.35	0.06	0.53	0.88	**1**
Data_11	0.24	0.65	0.53	0.59	0.82	0.18	0.88	0.76	0.94	0.06	0.29	0.35	0.41	0.47	0	0.71	0.12	**1**
Data_12	0.18	**1**	0.24	0.47	0.82	0.12	0.76	0.71	0.53	0	0.35	0.88	0.65	0.41	0.06	0.29	0.59	0.94
Data_13	0.33	0.25	NA	NA	0.67	0.5	0.83	0.17	NA	0.08	NA	0.58	NA	**1**	0	0.75	0.42	0.92
Data_14	0.67	0.25	NA	NA	0.83	0.5	0.75	0.58	NA	0.08	NA	0.33	NA	**1**	0.17	0	0.42	0.92
Data_15	0.36	0.18	NA	NA	0.82	0.55	0.73	0.64	NA	0	NA	0.27	NA	**1**	0.09	0.45	NA	0.91
Data_16	0.5	0.17	NA	NA	0.58	**1**	0.67	0.75	NA	0.08	NA	0.33	NA	0.83	0	0.25	0.42	0.92
Data_17	0.58	0.08	NA	NA	0.75	0.5	0.83	0.67	NA	0.17	NA	0.42	NA	**1**	0.25	0	0.33	0.92
Data_18	0.7	0.3	NA	NA	0.8	0.6	NA	0.5	NA	0	NA	0.4	NA	0.9	0.2	0.1	NA	**1**
Data_19	0.7	0.2	NA	NA	0.6	0.5	NA	0.3	NA	0	NA	0.4	NA	0.8	0.1	0.9	NA	**1**
Data_20	0.56	0.22	NA	NA	0.78	NA	NA	0.67	NA	0	NA	0.44	NA	0.89	0.11	0.33	NA	**1**
Data_21	0.64	0.27	NA	NA	0.55	0.82	0.73	NA	NA	0	NA	0.45	NA	**1**	0.09	0.18	0.36	0.91
Data_22	0.5	0.93	0.43	NA	0.71	0.07	0.36	0.64	NA	0	NA	0.86	0.57	0.29	0.21	**1**	0.14	0.79
Data_23	0.43	0.64	0.5	NA	0.79	0.07	0.36	0.86	NA	0.14	NA	0.71	0.57	0.29	0.21	0.93	0	**1**
Data_24	0.38	0.69	0.46	NA	0.62	0.08	0.54	NA	NA	0.15	NA	**1**	0.85	0.31	0.23	0.92	0	0.77
Data_25	0.5	0.71	0.07	NA	0.79	0	0.21	0.64	NA	0.29	NA	0.93	0.86	0.43	0.36	0.57	0.14	**1**
Data_26	0.5	0.57	0.43	NA	0.71	0.07	0.36	0.64	NA	0.14	NA	**1**	0.79	0.29	0.21	0.93	0	0.86
Data_27	0.5	0.57	0.43	NA	0.79	0.21	0.36	0.64	NA	0.14	NA	**1**	0.93	0.29	0.07	0.71	0	0.86
Data_28	0.5	0.64	0.29	NA	0.86	0	0.36	0.57	NA	0.21	NA	0.93	0.79	0.43	0.14	0.71	0.07	**1**
Data_29	0.5	0.71	0.14	NA	0.86	0	0.43	0.57	NA	0.21	NA	**1**	0.93	0.36	0.29	0.64	0.07	0.79
Data_30	0.5	0.43	0.71	NA	0.93	0.07	0.64	0.79	NA	0.21	NA	0.57	0.36	0.14	0.29	0.86	0	**1**
Data_31	0.43	0.57	0.64	NA	0.71	0.07	0.5	**1**	NA	0.14	NA	0.79	0.29	0.36	0.21	0.93	0	0.86
Data_32	0.43	0.71	0.57	NA	0.79	0.21	0.36	0.86	NA	0	NA	0.93	0.5	0.29	0.07	**1**	0.14	0.64
Data_33	0.29	0.64	0.57	NA	0.71	0.21	0.5	0.79	NA	0	NA	**1**	0.36	0.43	0.07	0.93	0.14	0.86
Data_34	0.29	0.64	0.43	NA	0.79	0.21	0.5	0.71	NA	0	NA	0.86	0.57	0.36	0.07	**1**	0.14	0.93

*NA indicates missing values due to unavailability of the original metric or method incompatibility with specific datasets. The 'Mean score' row at the bottom represents the average normalized rank score for each method across all applicable datasets. A higher mean score indicates better overall performance under the given evaluation metric.

Supplementary Table 7

Comparison of SAGE with 16 state-of-the-art spatial transcriptomics domain analysis methods in terms of COM

Data Name	STAMP	GASTON	STAGATE	stLearn	PROST	SEDR	GraphST	BASS	BayesSpace	louvain	SpaGCN (HE)	SCAN-IT	CCST	SpaGCN	leiden	SpaceFlow	conST	SAGE
Data_1	0.45	0.62	0.51	0.63	0.65	**0.78**	0.70	0.65	0.64	0.24	0.51	0.67	0.55	0.56	0.22	0.54	0.49	0.70
Data_2	0.44	0.63	0.46	0.55	0.54	**0.70**	0.56	0.61	0.60	0.22	0.45	0.60	0.51	0.51	0.22	0.44	0.46	0.68
Data_3	0.42	0.62	0.52	0.61	0.59	**0.73**	0.60	0.60	0.57	0.32	0.49	0.62	0.52	0.53	0.27	0.43	0.50	0.68
Data_4	0.46	0.56	0.48	0.49	0.57	**0.84**	0.56	0.54	0.55	0.23	0.48	0.57	0.45	0.53	0.24	0.48	0.45	0.68
Data_5	0.35	0.53	0.32	0.49	0.39	**0.67**	0.55	0.53	0.56	0.14	0.35	0.31	0.33	0.44	0.14	0.34	0.49	0.59
Data_6	0.28	0.52	0.35	0.32	0.42	**0.62**	0.43	0.40	0.50	0.14	0.34	0.39	0.35	0.39	0.14	0.31	0.45	0.50
Data_7	0.36	0.55	0.44	0.58	0.64	**0.81**	0.65	0.65	0.69	0.23	0.51	0.48	0.47	0.58	0.23	0.37	0.61	0.80
Data_8	0.33	0.63	0.50	0.50	0.68	0.60	0.64	0.65	0.60	0.22	0.54	0.59	0.50	0.62	0.21	0.43	0.42	**0.79**
Data_9	0.45	0.72	0.56	0.56	0.60	0.62	0.68	0.69	0.67	0.30	0.46	0.58	0.46	0.48	0.30	0.37	0.65	**0.73**
Data_10	0.40	0.73	0.50	0.55	0.47	0.59	0.53	0.57	0.47	0.34	0.46	0.51	0.48	0.48	0.32	0.36	0.59	**0.77**
Data_11	0.46	0.54	0.56	0.59	0.60	0.69	0.64	0.61	0.70	0.35	0.47	0.51	0.47	0.52	0.34	0.40	0.40	**0.72**
Data_12	0.45	0.72	0.46	0.55	0.61	0.69	0.61	0.56	0.53	0.32	0.48	0.58	0.50	0.51	0.32	0.34	0.53	**0.74**
Data_13	0.44	0.43	NA	NA	0.50	0.49	0.53	0.44	NA	0.28	NA	0.51	NA	0.54	0.26	0.46	0.48	**0.56**
Data_14	0.52	0.42	NA	NA	0.55	0.53	0.54	0.54	NA	0.33	NA	0.51	NA	**0.58**	0.33	0.19	0.48	**0.58**
Data_15	0.56	0.47	NA	NA	0.58	0.59	0.59	0.60	NA	0.28	NA	0.51	NA	0.61	0.31	0.42	NA	**0.64**
Data_16	0.58	0.46	NA	NA	0.60	**0.66**	0.61	0.62	NA	0.35	NA	0.59	NA	0.64	0.36	0.39	0.57	**0.66**
Data_17	0.51	0.37	NA	NA	0.54	0.54	0.57	0.55	NA	0.39	NA	0.50	NA	**0.58**	0.38	0.22	0.48	**0.58**
Data_18	0.46	0.35	NA	NA	0.50	0.47	NA	0.45	NA	0.34	NA	0.42	NA	0.54	0.34	0.26	NA	**0.56**
Data_19	0.40	0.36	NA	NA	0.40	0.42	NA	0.40	NA	0.24	NA	0.40	NA	0.46	0.22	0.34	NA	**0.52**
Data_20	0.47	0.40	NA	NA	0.51	NA	NA	0.53	NA	0.29	NA	0.49	NA	0.53	0.32	0.31	NA	**0.57**
Data_21	0.57	0.42	NA	NA	0.57	0.62	0.60	NA	NA	0.29	NA	0.55	NA	**0.64**	0.32	0.34	0.53	**0.64**
Data_22	0.37	0.75	0.34	NA	0.66	0.06	0.36	0.76	NA	0.02	NA	0.73	0.59	0.31	0.06	0.64	0.04	**0.80**
Data_23	0.41	0.69	0.48	NA	0.73	0.09	0.43	0.81	NA	0.10	NA	0.71	0.58	0.26	0.10	0.63	0.04	**0.83**
Data_24	0.25	0.63	0.28	NA	0.44	0.06	0.34	NA	NA	0.06	NA	**0.70**	0.57	0.18	0.08	0.58	0.03	0.68
Data_25	0.19	0.42	0.09	NA	0.44	0.11	0.37	0.54	NA	0.16	NA	0.51	0.40	0.18	0.19	0.48	0.10	**0.56**
Data_26	0.30	0.39	0.29	NA	0.49	0.18	0.58	0.61	NA	0.18	NA	0.54	0.39	0.23	0.20	**0.62**	0.09	0.58
Data_27	0.32	0.45	0.28	NA	0.50	0.27	**0.57**	0.66	NA	0.19	NA	0.55	0.41	0.22	0.18	0.56	0.11	0.55
Data_28	0.32	0.53	0.20	NA	0.57	0.13	**0.73**	0.72	NA	0.19	NA	0.57	0.41	0.22	0.19	0.65	0.12	0.63
Data_29	0.31	0.56	0.14	NA	0.56	0.25	**0.71**	0.72	NA	0.19	NA	0.63	0.47	0.23	0.19	0.69	0.11	0.61
Data_30	0.46	0.48	0.50	NA	0.62	0.19	0.53	0.59	NA	0.35	NA	0.52	0.49	0.24	0.35	0.55	0.13	**0.70**
Data_31	0.38	0.53	0.60	NA	0.63	0.06	0.54	0.79	NA	0.12	NA	0.64	0.34	0.30	0.10	0.58	0.04	**0.79**
Data_32	0.34	0.69	0.41	NA	0.51	0.19	0.35	0.54	NA	0.07	NA	0.52	0.39	0.31	0.07	0.44	0.09	**0.70**
Data_33	0.26	0.56	0.54	NA	0.57	0.08	0.40	0.69	NA	0.05	NA	0.54	0.32	0.28	0.03	0.51	0.06	**0.68**
Data_34	0.28	0.63	0.40	NA	0.70	0.28	0.50	0.66	NA	0.12	NA	0.69	0.58	0.37	0.12	0.61	0.23	**0.77**

*NA in the table indicates that SDMbench did not provide the COM metric results for that method on the given dataset, or that the method (SpaGCN (HE)) is not applicable to the specific spatial transcriptomics technology dataset.

Supplementary Table 8

Normalized rank scores of SAGE and 16 baseline methods based on COM, reflecting performance ranks (0–1 scale, higher is better) across 34 datasets.

Data Name	STAMP	GASTON	STAGATE	stLearn	PROST	SEDR	GraphST	BASS	BayesSpace	louvain	SpaGCN (HE)	SCAN-IT	CCST	SpaGCN	leiden	SpaceFlow	conST	SAGE
Data_1	0.12	0.53	0.24	0.59	0.76	**1**	0.88	0.71	0.65	0.06	0.29	0.82	0.41	0.47	0	0.35	0.18	0.94
Data_2	0.18	0.88	0.29	0.59	0.53	**1**	0.65	0.82	0.76	0	0.24	0.71	0.47	0.41	0.06	0.12	0.35	0.94
Data_3	0.12	0.88	0.35	0.76	0.59	**1**	0.71	0.65	0.53	0.06	0.24	0.82	0.41	0.47	0	0.18	0.29	0.94
Data_4	0.24	0.76	0.29	0.47	0.82	**1**	0.71	0.59	0.65	0	0.35	0.88	0.18	0.53	0.06	0.41	0.12	0.94
Data_5	0.41	0.71	0.18	0.65	0.47	**1**	0.82	0.76	0.88	0.06	0.35	0.12	0.24	0.53	0	0.29	0.59	0.94
Data_6	0.12	0.94	0.35	0.24	0.65	**1**	0.71	0.59	0.82	0	0.29	0.53	0.41	0.47	0.06	0.18	0.76	0.88
Data_7	0.12	0.47	0.24	0.53	0.71	**1**	0.82	0.76	0.88	0	0.41	0.35	0.29	0.59	0.06	0.18	0.65	0.94
Data_8	0.12	0.76	0.41	0.29	0.94	0.65	0.82	0.88	0.59	0.06	0.47	0.53	0.35	0.71	0	0.24	0.18	**1**
Data_9	0.18	0.94	0.41	0.47	0.59	0.65	0.82	0.88	0.76	0	0.29	0.53	0.24	0.35	0.06	0.12	0.71	**1**
Data_10	0.18	0.94	0.53	0.71	0.29	0.88	0.65	0.76	0.35	0.06	0.24	0.59	0.41	0.47	0	0.12	0.82	**1**
Data_11	0.24	0.53	0.59	0.65	0.71	0.88	0.82	0.76	0.94	0.06	0.29	0.41	0.35	0.47	0	0.18	0.12	**1**
Data_12	0.18	0.94	0.24	0.59	0.82	0.88	0.76	0.65	0.47	0	0.29	0.71	0.35	0.41	0.06	0.12	0.53	**1**
Data_13	0.33	0.17	NA	NA	0.67	0.58	0.83	0.25	NA	0.08	NA	0.75	NA	0.92	0	0.42	0.5	**1**
Data_14	0.5	0.25	NA	NA	0.83	0.58	0.75	0.67	NA	0.08	NA	0.42	NA	0.92	0.17	0	0.33	**1**
Data_15	0.45	0.27	NA	NA	0.55	0.73	0.64	0.82	NA	0	NA	0.36	NA	0.91	0.09	0.18	NA	**1**
Data_16	0.42	0.25	NA	NA	0.58	**1**	0.67	0.75	NA	0	NA	0.5	NA	0.83	0.08	0.17	0.33	0.92
Data_17	0.5	0.08	NA	NA	0.58	0.67	0.83	0.75	NA	0.25	NA	0.42	NA	**1**	0.17	0	0.33	0.92
Data_18	0.6	0.3	NA	NA	0.8	0.7	NA	0.5	NA	0.2	NA	0.4	NA	0.9	0.1	0	NA	**1**
Data_19	0.5	0.3	NA	NA	0.7	0.8	NA	0.4	NA	0.1	NA	0.6	NA	0.9	0	0.2	NA	**1**
Data_20	0.44	0.33	NA	NA	0.67	NA	NA	0.89	NA	0	NA	0.56	NA	0.78	0.22	0.11	NA	**1**
Data_21	0.55	0.27	NA	NA	0.64	0.82	0.73	NA	NA	0	NA	0.45	NA	0.91	0.09	0.18	0.36	**1**
Data_22	0.5	0.86	0.36	NA	0.71	0.14	0.43	0.93	NA	0	NA	0.79	0.57	0.29	0.21	0.64	0.07	**1**
Data_23	0.36	0.71	0.5	NA	0.86	0.07	0.43	0.93	NA	0.14	NA	0.79	0.57	0.29	0.21	0.64	0	**1**
Data_24	0.38	0.85	0.46	NA	0.62	0.15	0.54	NA	NA	0.08	NA	1	0.69	0.31	0.23	0.77	0	0.92
Data_25	0.43	0.64	0	NA	0.71	0.14	0.5	0.93	NA	0.21	NA	0.86	0.57	0.29	0.36	0.79	0.07	**1**
Data_26	0.43	0.57	0.36	NA	0.64	0.07	0.79	0.93	NA	0.14	NA	0.71	0.5	0.29	0.21	**1**	0	0.86
Data_27	0.43	0.57	0.36	NA	0.64	0.29	0.93	**1**	NA	0.14	NA	0.71	0.5	0.21	0.07	0.86	0	0.79
Data_28	0.43	0.57	0.29	NA	0.64	0.07	**1**	0.93	NA	0.21	NA	0.71	0.5	0.36	0.14	0.86	0	0.79
Data_29	0.43	0.57	0.07	NA	0.64	0.36	0.93	**1**	NA	0.14	NA	0.79	0.5	0.29	0.21	0.86	0	0.71
Data_30	0.36	0.43	0.57	NA	0.93	0.07	0.71	0.86	NA	0.29	NA	0.64	0.5	0.14	0.21	0.79	0	**1**
Data_31	0.43	0.5	0.71	NA	0.79	0.07	0.57	0.93	NA	0.21	NA	0.86	0.36	0.29	0.14	0.64	0	**1**
Data_32	0.36	0.93	0.57	NA	0.71	0.21	0.43	0.86	NA	0.07	NA	0.79	0.5	0.29	0	0.64	0.14	**1**
Data_33	0.29	0.79	0.64	NA	0.86	0.21	0.5	**1**	NA	0.07	NA	0.71	0.43	0.36	0	0.57	0.14	0.93
Data_34	0.21	0.71	0.43	NA	0.93	0.29	0.5	0.79	NA	0.07	NA	0.86	0.57	0.36	0	0.64	0.14	**1**

*NA indicates missing values due to unavailability of the original metric or method incompatibility with specific datasets. The 'Mean score' row at the bottom represents the average normalized rank score for each method across all applicable datasets. A higher mean score indicates better overall performance under the given evaluation metric.

Supplementary Table 9

Comparison of methods based on mean rank scores of accuracy-related metrics (NMI, HOM, COM)

Data Name	STAMP	GASTON	STAGATE	stLearn	PROST	SEDR	GraphST	BASS	BayesSpace	louvain	SpaGCN (HE)	SCAN-IT	CCST	SpaGCN	leiden	SpaceFlow	conST	SAGE
Data_1	0.14	0.63	0.31	0.55	0.82	0.45	0.9	0.74	0.61	0.02	0.29	0.78	0.45	0.43	0.04	0.67	0.2	0.96
Data_2	0.2	0.86	0.31	0.47	0.61	0.49	0.65	0.82	0.74	0	0.2	0.82	0.53	0.45	0.06	0.53	0.28	0.98
Data_3	0.14	0.88	0.35	0.65	0.71	0.55	0.75	0.72	0.57	0.06	0.22	0.9	0.43	0.45	0	0.36	0.29	0.98
Data_4	0.3	0.8	0.39	0.31	0.86	0.53	0.74	0.63	0.61	0	0.33	0.92	0.18	0.53	0.06	0.71	0.22	0.88
Data_5	0.28	0.73	0.14	0.59	0.39	0.71	0.8	0.84	0.88	0.06	0.29	0.22	0.39	0.51	0	0.59	0.63	0.96
Data_6	0.12	0.88	0.33	0.2	0.63	0.7	0.59	0.55	0.88	0	0.26	0.73	0.57	0.43	0.06	0.51	0.72	0.84
Data_7	0.12	0.45	0.2	0.47	0.78	0.65	0.82	0.8	0.84	0.02	0.33	0.39	0.45	0.59	0.04	0.45	0.61	0.98
Data_8	0.12	0.74	0.37	0.29	0.94	0.36	0.82	0.74	0.55	0.06	0.45	0.69	0.49	0.67	0	0.55	0.2	0.96
Data_9	0.16	0.92	0.45	0.41	0.61	0.63	0.82	0.9	0.76	0	0.24	0.53	0.26	0.33	0.06	0.22	0.71	1
Data_10	0.14	0.94	0.49	0.73	0.29	0.78	0.59	0.8	0.31	0.04	0.22	0.61	0.49	0.41	0.02	0.28	0.86	1
Data_11	0.22	0.59	0.59	0.65	0.78	0.53	0.86	0.76	0.94	0.06	0.29	0.39	0.37	0.47	0	0.38	0.12	1
Data_12	0.18	0.96	0.24	0.55	0.84	0.45	0.78	0.69	0.51	0	0.31	0.78	0.49	0.41	0.06	0.18	0.59	0.98
Data_13	0.33	0.22	NA	NA	0.67	0.53	0.83	0.2	NA	0.08	NA	0.69	NA	0.95	0	0.58	0.45	0.97
Data_14	0.56	0.25	NA	NA	0.83	0.55	0.75	0.64	NA	0.08	NA	0.39	NA	0.97	0.17	0	0.36	0.95
Data_15	0.42	0.21	NA	NA	0.7	0.61	0.67	0.76	NA	0	NA	0.33	NA	0.94	0.09	0.3	NA	0.97
Data_16	0.47	0.22	NA	NA	0.58	1	0.67	0.75	NA	0.03	NA	0.39	NA	0.83	0.05	0.2	0.39	0.92
Data_17	0.53	0.08	NA	NA	0.69	0.58	0.83	0.7	NA	0.22	NA	0.42	NA	1	0.2	0	0.33	0.92
Data_18	0.63	0.3	NA	NA	0.8	0.67	NA	0.5	NA	0.1	NA	0.4	NA	0.9	0.17	0.03	NA	1
Data_19	0.63	0.23	NA	NA	0.63	0.7	NA	0.33	NA	0.07	NA	0.5	NA	0.87	0.03	0.5	NA	1
Data_20	0.48	0.29	NA	NA	0.71	NA	NA	0.78	NA	0	NA	0.52	NA	0.85	0.15	0.22	NA	1
Data_21	0.61	0.27	NA	NA	0.58	0.82	0.73	NA	NA	0	NA	0.45	NA	0.94	0.09	0.18	0.36	0.97
Data_22	0.5	0.91	0.38	NA	0.69	0.12	0.41	0.79	NA	0	NA	0.84	0.57	0.29	0.21	0.78	0.09	0.93
Data_23	0.41	0.66	0.5	NA	0.84	0.07	0.38	0.91	NA	0.14	NA	0.76	0.57	0.29	0.21	0.76	0	1
Data_24	0.38	0.8	0.46	NA	0.62	0.1	0.54	NA	NA	0.13	NA	1	0.74	0.31	0.23	0.82	0	0.87
Data_25	0.45	0.66	0.02	NA	0.76	0.07	0.4	0.76	NA	0.24	NA	0.91	0.76	0.36	0.34	0.64	0.12	1
Data_26	0.45	0.57	0.38	NA	0.69	0.07	0.55	0.79	NA	0.14	NA	0.86	0.64	0.29	0.21	0.98	0	0.88
Data_27	0.45	0.57	0.38	NA	0.71	0.24	0.6	0.83	NA	0.14	NA	0.9	0.69	0.26	0.07	0.79	0	0.86
Data_28	0.48	0.62	0.29	NA	0.79	0.02	0.6	0.74	NA	0.21	NA	0.86	0.62	0.38	0.14	0.79	0.05	0.93
Data_29	0.45	0.64	0.09	NA	0.74	0.17	0.62	0.79	NA	0.19	NA	0.93	0.67	0.34	0.26	0.81	0.02	0.79
Data_30	0.41	0.43	0.64	NA	0.93	0.07	0.69	0.84	NA	0.26	NA	0.59	0.45	0.14	0.24	0.81	0	1
Data_31	0.43	0.55	0.66	NA	0.74	0.07	0.52	0.98	NA	0.16	NA	0.84	0.34	0.31	0.19	0.79	0	0.93
Data_32	0.41	0.88	0.57	NA	0.74	0.21	0.38	0.86	NA	0.02	NA	0.84	0.5	0.29	0.05	0.76	0.14	0.86
Data_33	0.29	0.69	0.59	NA	0.79	0.21	0.5	0.91	NA	0.05	NA	0.86	0.41	0.38	0.02	0.74	0.14	0.93
Data_34	0.26	0.66	0.43	NA	0.88	0.24	0.5	0.74	NA	0.02	NA	0.86	0.57	0.36	0.05	0.81	0.14	0.98

* NA indicates missing values due to unavailability of the original metric or method incompatibility with specific datasets. Accuracy evaluation of SAGE and 14 baseline methods based on the mean normalized rank scores across three metrics: NMI, HOM, and COM. Each score represents the average rank score of a method across the three metrics for a given dataset. Higher scores indicate greater agreement with manual annotations.

Supplementary Table 10

Comparison of SAGE with 16 state-of-the-art spatial transcriptomics domain analysis methods in terms of CHAOS

Data Name	STAMP	GASTON	STAGATE	stLearn	PROST	SEDR	GraphST	BASS	BayesSpace	louvain	SpaGCN (HE)	SCAN-IT	CCST	SpaGCN	leiden	SpaceFlow	conST	SAGE
Data_1	0.063	0.056	0.058	0.059	**0.056**	0.058	0.057	0.057	**0.056**	0.063	0.062	**0.056**	**0.056**	0.060	0.065	0.057	0.062	**0.056**
Data_2	0.062	0.055	0.057	0.058	0.056	0.056	0.057	0.056	0.056	0.064	0.062	**0.055**	**0.055**	0.060	0.064	0.056	0.059	**0.055**
Data_3	0.059	0.054	0.054	0.055	**0.054**	0.055	0.055	**0.054**	**0.054**	0.061	0.058	**0.054**	**0.054**	0.057	0.061	**0.054**	0.057	**0.054**
Data_4	0.062	0.055	0.056	0.058	**0.055**	**0.055**	0.058	0.056	0.056	0.063	0.060	0.055	0.055	0.059	0.063	0.056	0.060	0.056
Data_5	0.068	0.061	0.063	0.065	**0.061**	0.062	0.062	0.062	0.062	0.069	0.068	**0.061**	**0.061**	0.065	0.069	0.063	0.063	**0.061**
Data_6	0.068	0.060	0.061	0.064	**0.060**	0.061	0.063	0.062	0.061	0.069	0.067	**0.060**	**0.060**	0.065	0.070	0.061	0.064	**0.060**
Data_7	0.063	0.058	**0.058**	0.061	**0.058**	**0.058**	0.059	**0.058**	0.059	0.065	0.063	**0.058**	**0.058**	0.061	0.065	0.060	0.059	**0.058**
Data_8	0.066	0.060	0.062	0.064	**0.060**	**0.060**	0.061	**0.060**	**0.060**	0.067	0.064	**0.060**	**0.060**	0.062	0.067	0.061	0.063	**0.060**
Data_9	0.066	0.060	**0.061**	0.063	**0.061**	0.062	**0.061**	**0.061**	**0.061**	0.067	0.067	**0.061**	**0.061**	0.065	0.069	0.064	**0.061**	**0.061**
Data_10	0.066	0.060	0.061	0.063	**0.060**	0.061	0.061	**0.060**	0.061	0.066	0.065	**0.060**	**0.060**	0.064	0.067	0.063	0.062	**0.060**
Data_11	0.068	0.061	0.062	0.063	0.061	0.062	0.062	0.061	0.062	0.067	0.066	**0.060**	**0.060**	0.065	0.068	0.063	0.069	**0.060**
Data_12	0.068	0.062	0.064	0.065	0.063	0.063	0.065	0.063	0.066	0.072	0.069	0.063	**0.062**	0.067	0.071	0.067	0.064	0.063
Data_13	0.038	0.036	NA	NA	0.037	**0.036**	**0.036**	0.037	NA	0.043	NA	0.035	NA	0.039	0.043	0.037	0.040	0.038
Data_14	0.051	0.046	NA	NA	0.048	0.046	0.047	0.047	NA	0.049	NA	**0.045**	NA	0.049	0.051	0.080	0.048	0.047
Data_15	0.052	0.048	NA	NA	**0.049**	0.048	0.049	0.049	NA	0.061	NA	0.047	NA	0.052	0.064	0.052	NA	0.049
Data_16	0.050	0.047	NA	NA	0.048	0.048	0.048	0.047	NA	0.053	NA	**0.046**	NA	0.049	0.053	0.056	0.048	0.048
Data_17	0.052	0.046	NA	NA	0.046	0.045	0.048	0.047	NA	0.051	NA	**0.045**	NA	0.048	0.052	0.065	0.047	0.047
Data_18	0.022	0.019	NA	NA	0.020	0.019	NA	0.020	NA	0.022	NA	**0.019**	NA	0.020	0.023	0.025	NA	**0.019**
Data_19	0.022	0.018	NA	NA	0.019	0.019	NA	0.019	NA	0.024	NA	**0.018**	NA	0.020	0.025	0.020	NA	0.019
Data_20	0.023	0.018	NA	NA	0.019	NA	NA	0.019	NA	0.024	NA	**0.018**	NA	0.020	0.023	0.025	NA	0.019
Data_21	0.038	0.033	NA	NA	0.032	0.034	0.033	NA	NA	0.045	NA	**0.031**	NA	0.036	0.044	0.041	0.035	0.033
Data_22	0.070	0.052	0.073	NA	0.051	0.086	0.063	**0.052**	NA	0.096	NA	0.054	0.051	0.081	0.100	0.056	0.106	**0.052**
Data_23	0.064	0.047	0.056	NA	0.048	0.082	0.056	0.049	NA	0.088	NA	0.048	**0.046**	0.076	0.088	0.051	0.093	0.048
Data_24	0.075	0.050	0.070	NA	0.053	0.096	0.063	NA	NA	0.107	NA	0.050	**0.049**	0.088	0.096	0.052	0.103	0.051
Data_25	0.045	0.029	0.055	NA	0.030	0.051	0.032	0.030	NA	0.057	NA	0.029	**0.028**	0.050	0.057	0.029	0.062	0.029
Data_26	0.044	0.030	0.046	NA	0.029	0.048	0.031	0.030	NA	0.055	NA	0.030	**0.028**	0.050	0.056	0.030	0.060	0.030
Data_27	0.043	0.028	0.044	NA	**0.028**	0.047	0.030	0.029	NA	0.053	NA	0.029	**0.028**	0.046	0.054	**0.028**	0.058	**0.028**
Data_28	0.043	0.028	0.046	NA	0.028	0.046	0.028	0.029	NA	0.053	NA	0.029	**0.027**	0.048	0.053	0.028	0.059	0.028
Data_29	0.046	0.029	0.052	NA	0.029	0.039	0.030	0.029	NA	0.055	NA	0.029	**0.028**	0.049	0.055	0.029	0.062	0.029
Data_30	0.026	0.020	0.024	NA	0.020	0.044	0.024	0.021	NA	0.033	NA	0.029	0.027	0.037	0.034	0.031	0.050	**0.020**
Data_31	0.087	0.069	0.074	NA	0.069	0.106	0.075	0.071	NA	0.089	NA	**0.069**	0.074	0.089	0.100	0.072	0.108	0.070
Data_32	0.091	0.073	0.079	NA	**0.073**	0.107	0.077	0.076	NA	0.092	NA	0.075	0.077	0.090	0.100	0.075	0.110	**0.073**
Data_33	0.096	0.073	0.077	NA	**0.073**	0.109	0.079	0.075	NA	0.092	NA	0.074	0.079	0.094	0.103	0.076	0.113	**0.073**
Data_34	0.097	0.063	0.079	NA	**0.063**	0.108	0.075	0.065	NA	0.105	NA	0.066	0.069	0.092	0.116	0.071	0.113	**0.063**

*NA in the table indicates that SDMbench did not provide the CHAOS metric results for that method on the given dataset, or that the method (SpaGCN (HE)) is not applicable to the specific spatial transcriptomics technology dataset.

Supplementary Table 11

Normalized rank scores of SAGE and 16 baseline methods based on CHAOS, reflecting performance ranks (0–1 scale, higher is better) across 34 datasets.

Data Name	STAMP	GASTON	STAGATE	stLearn	PROST	SEDR	GraphST	BASS	BayesSpace	louvain	SpaGCN (HE)	SCAN-IT	CCST	SpaGCN	leiden	SpaceFlow	conST	SAGE
Data_1	0.06	0.76	0.41	0.35	0.88	0.47	0.65	0.53	0.71	0.12	0.18	0.94	1	0.29	0	0.59	0.24	0.82
Data_2	0.12	0.88	0.47	0.35	0.65	0.59	0.41	0.53	0.76	0	0.18	0.94	0.82	0.24	0.06	0.71	0.29	1
Data_3	0.12	0.82	0.53	0.35	0.94	0.47	0.41	0.65	0.71	0	0.18	0.76	0.88	0.29	0.06	0.59	0.24	1
Data_4	0.12	1	0.53	0.35	0.76	0.88	0.41	0.59	0.47	0.06	0.18	0.94	0.82	0.29	0	0.71	0.24	0.65
Data_5	0.12	0.94	0.35	0.24	0.88	0.53	0.59	0.65	0.71	0.06	0.18	0.76	0.82	0.29	0	0.47	0.41	1
Data_6	0.12	1	0.65	0.35	0.88	0.53	0.41	0.47	0.71	0.06	0.18	0.94	0.82	0.24	0	0.59	0.29	0.76
Data_7	0.12	0.76	0.59	0.29	1	0.71	0.53	0.65	0.47	0	0.18	0.94	0.82	0.24	0.06	0.35	0.41	0.88
Data_8	0.12	0.88	0.41	0.24	0.94	0.59	0.53	0.71	0.65	0	0.18	0.82	0.76	0.35	0.06	0.47	0.29	1
Data_9	0.18	1	0.76	0.35	0.88	0.41	0.53	0.47	0.71	0.06	0.12	0.94	0.82	0.24	0	0.29	0.65	0.59
Data_10	0.06	0.94	0.53	0.29	0.71	0.47	0.59	0.82	0.65	0.12	0.18	0.88	0.76	0.24	0	0.35	0.41	1
Data_11	0.12	0.76	0.47	0.41	0.82	0.65	0.59	0.71	0.53	0.18	0.24	0.88	1	0.29	0.06	0.35	0	0.94
Data_12	0.18	0.94	0.59	0.41	0.76	0.71	0.47	0.65	0.35	0	0.12	0.88	1	0.24	0.06	0.29	0.53	0.82
Data_13	0.33	0.92	NA	NA	0.58	0.75	0.83	0.67	NA	0	NA	1	NA	0.25	0.08	0.5	0.17	0.42
Data_14	0.08	0.83	NA	NA	0.42	0.92	0.67	0.75	NA	0.25	NA	1	NA	0.33	0.17	0	0.5	0.58
Data_15	0.27	0.91	NA	NA	0.55	0.82	0.64	0.73	NA	0.09	NA	1	NA	0.36	0	0.18	NA	0.45
Data_16	0.25	0.92	NA	NA	0.5	0.58	0.67	0.83	NA	0.08	NA	1	NA	0.33	0.17	0	0.42	0.75
Data_17	0.17	0.83	NA	NA	0.75	0.92	0.42	0.67	NA	0.25	NA	1	NA	0.33	0.08	0	0.5	0.58
Data_18	0.3	0.9	NA	NA	0.5	0.8	NA	0.7	NA	0.2	NA	1	NA	0.4	0.1	0	NA	0.6
Data_19	0.2	1	NA	NA	0.7	0.8	NA	0.6	NA	0.1	NA	0.9	NA	0.3	0	0.4	NA	0.5
Data_20	0.33	0.89	NA	NA	0.67	NA	NA	0.78	NA	0.11	NA	1	NA	0.44	0.22	0	NA	0.56
Data_21	0.27	0.82	NA	NA	0.91	0.55	0.73	NA	NA	0	NA	1	NA	0.36	0.09	0.18	0.45	0.64
Data_22	0.43	0.86	0.36	NA	0.93	0.21	0.5	0.71	NA	0.14	NA	0.64	1	0.29	0.07	0.57	0	0.79
Data_23	0.36	0.93	0.43	NA	0.79	0.21	0.5	0.64	NA	0.14	NA	0.71	1	0.29	0.07	0.57	0	0.86
Data_24	0.38	0.85	0.46	NA	0.62	0.15	0.54	NA	NA	0	NA	0.92	1	0.31	0.23	0.69	0.08	0.77
Data_25	0.43	0.71	0.21	NA	0.57	0.29	0.5	0.64	NA	0.14	NA	0.79	1	0.36	0.07	0.86	0	0.93
Data_26	0.43	0.86	0.36	NA	0.93	0.29	0.5	0.57	NA	0.14	NA	0.71	1	0.21	0.07	0.79	0	0.64
Data_27	0.43	0.86	0.36	NA	0.79	0.21	0.5	0.57	NA	0.14	NA	0.64	1	0.29	0.07	0.71	0	0.93
Data_28	0.43	0.86	0.29	NA	0.71	0.36	0.93	0.5	NA	0.07	NA	0.57	1	0.21	0.14	0.64	0	0.79
Data_29	0.36	0.86	0.21	NA	0.93	0.43	0.5	0.79	NA	0.14	NA	0.64	1	0.29	0.07	0.57	0	0.71
Data_30	0.57	0.86	0.64	NA	0.93	0.07	0.71	0.79	NA	0.29	NA	0.43	0.5	0.14	0.21	0.36	0	1
Data_31	0.36	0.93	0.57	NA	1	0.07	0.43	0.71	NA	0.29	NA	0.86	0.5	0.21	0.14	0.64	0	0.79
Data_32	0.29	0.86	0.43	NA	1	0.07	0.57	0.64	NA	0.21	NA	0.79	0.5	0.36	0.14	0.71	0	0.93
Data_33	0.21	0.86	0.57	NA	0.93	0.07	0.5	0.71	NA	0.36	NA	0.79	0.43	0.29	0.14	0.64	0	1
Data_34	0.29	1	0.43	NA	0.86	0.14	0.5	0.79	NA	0.21	NA	0.71	0.64	0.36	0	0.57	0.07	0.93

*NA indicates missing values due to unavailability of the original metric or method incompatibility with specific datasets. The 'Mean score' row at the bottom represents the average normalized rank score for each method across all applicable datasets. A higher mean score indicates better overall performance under the given evaluation metric.

Supplementary Table 12

Comparison of SAGE with 16 state-of-the-art spatial transcriptomics domain analysis methods in terms of PAS

Data Name	STAMP	GASTON	STAGATE	stLearn	PROST	SEDR	GraphST	BASS	BayesSpace	louvain	SpaGCN (HE)	SCAN-IT	CCST	SpaGCN	leiden	SpaceFlow	conST	SAGE
Data_1	0.319	0.013	0.112	0.089	**0.008**	0.013	0.050	0.027	0.032	0.493	0.248	0.009	0.009	0.131	0.611	0.055	0.322	0.018
Data_2	0.328	0.011	0.145	0.068	0.014	**0.010**	0.120	0.034	0.031	0.614	0.253	0.012	**0.010**	0.147	0.599	0.062	0.208	**0.010**
Data_3	0.317	0.023	0.078	0.085	**0.006**	0.015	0.100	0.027	0.031	0.515	0.249	0.009	0.012	0.159	0.602	0.153	0.305	0.011
Data_4	0.290	0.006	0.105	0.201	0.012	**0.010**	0.125	0.044	0.044	0.592	0.298	0.009	0.008	0.216	0.613	0.054	0.310	0.017
Data_5	0.261	0.020	0.104	0.132	0.015	0.013	0.074	0.026	0.022	0.443	0.295	0.017	0.012	0.130	0.499	0.071	0.064	**0.010**
Data_6	0.346	0.013	0.075	0.150	**0.003**	0.008	0.084	0.030	0.024	0.424	0.232	0.010	0.011	0.132	0.423	0.098	0.167	0.026
Data_7	0.339	0.024	0.072	0.123	**0.004**	0.010	0.058	0.026	0.025	0.425	0.173	0.008	0.012	0.086	0.378	0.135	0.068	0.010
Data_8	0.402	0.010	0.092	0.136	**0.005**	0.044	0.063	0.020	0.024	0.514	0.185	0.010	0.014	0.086	0.530	0.157	0.318	0.007
Data_9	0.316	0.013	0.055	0.118	0.020	0.066	0.080	0.038	0.046	0.417	0.243	0.015	**0.009**	0.154	0.438	0.253	0.049	0.018
Data_10	0.334	0.017	0.062	0.110	0.015	0.039	0.100	0.029	0.038	0.299	0.176	0.015	**0.010**	0.118	0.409	0.198	0.116	0.015
Data_11	0.317	0.020	0.067	0.130	**0.006**	0.050	0.134	0.028	0.052	0.401	0.254	0.012	0.014	0.128	0.417	0.169	0.404	0.022
Data_12	0.298	0.017	0.123	0.139	**0.007**	0.025	0.119	0.029	0.070	0.476	0.255	0.017	0.010	0.154	0.499	0.230	0.084	0.020
Data_13	0.173	0.033	NA	NA	**0.054**	0.055	0.072	0.144	NA	0.398	NA	0.013	NA	0.130	0.427	0.198	0.078	0.102
Data_14	0.181	0.053	NA	NA	0.104	0.073	0.146	0.096	NA	0.311	NA	**0.028**	NA	0.182	0.403	0.944	0.111	0.202
Data_15	0.238	0.049	NA	NA	0.093	0.096	0.157	**0.079**	NA	0.570	NA	0.042	NA	0.190	0.601	0.339	NA	0.157
Data_16	0.201	0.047	NA	NA	0.079	0.048	0.091	0.072	NA	0.433	NA	**0.027**	NA	0.145	0.386	0.557	0.102	0.101
Data_17	0.259	0.059	NA	NA	0.120	0.082	0.137	0.094	NA	0.440	NA	**0.030**	NA	0.199	0.490	0.711	0.213	0.175
Data_18	0.177	0.032	NA	NA	0.090	0.080	NA	0.083	NA	0.273	NA	**0.017**	NA	0.137	0.310	0.473	NA	0.145
Data_19	0.215	0.020	NA	NA	0.123	0.071	NA	0.096	NA	0.424	NA	**0.017**	NA	0.156	0.485	0.227	NA	0.252
Data_20	0.235	0.041	NA	NA	0.126	NA	NA	0.120	NA	0.481	NA	**0.024**	NA	0.198	0.443	0.517	NA	0.352
Data_21	0.248	0.114	NA	NA	0.083	0.118	0.152	NA	NA	0.598	NA	**0.017**	NA	0.234	0.577	0.552	0.174	0.184
Data_22	0.394	0.024	0.413	NA	0.028	0.287	0.279	0.019	NA	0.893	NA	0.028	**0.012**	0.444	0.847	0.066	0.909	0.018
Data_23	0.350	0.024	0.216	NA	**0.008**	0.271	0.239	0.029	NA	0.869	NA	0.033	0.016	0.604	0.868	0.099	0.912	0.024
Data_24	0.528	0.041	0.461	NA	0.093	0.445	0.356	NA	NA	0.937	NA	0.020	**0.016**	0.599	0.863	0.079	0.958	0.045
Data_25	0.584	0.053	0.702	NA	0.025	0.472	0.109	0.034	NA	0.614	NA	0.028	**0.005**	0.635	0.629	0.032	0.848	0.037
Data_26	0.491	0.064	0.499	NA	0.029	0.375	0.075	0.031	NA	0.573	NA	0.027	**0.005**	0.572	0.580	0.032	0.859	0.055
Data_27	0.422	0.034	0.497	NA	0.024	0.308	0.066	0.022	NA	0.497	NA	0.024	**0.004**	0.523	0.523	0.025	0.862	0.032
Data_28	0.444	0.044	0.586	NA	0.026	0.512	0.033	0.026	NA	0.555	NA	0.026	**0.006**	0.609	0.564	0.025	0.808	0.035
Data_29	0.509	0.036	0.661	NA	0.022	0.295	0.035	0.019	NA	0.602	NA	0.029	**0.005**	0.610	0.601	0.025	0.855	0.049
Data_30	0.295	0.047	0.164	NA	**0.016**	0.744	0.197	0.030	NA	0.422	NA	0.091	0.233	0.663	0.440	0.119	0.848	0.043
Data_31	0.316	0.022	0.076	NA	**0.011**	0.407	0.125	0.040	NA	0.285	NA	0.024	0.121	0.370	0.576	0.047	0.765	0.024
Data_32	0.292	0.037	0.116	NA	**0.010**	0.363	0.152	0.083	NA	0.334	NA	0.032	0.079	0.292	0.393	0.050	0.616	0.015
Data_33	0.431	0.028	0.076	NA	**0.004**	0.616	0.196	0.042	NA	0.330	NA	0.020	0.132	0.405	0.687	0.053	0.720	0.027
Data_34	0.558	0.041	0.313	NA	**0.014**	0.611	0.258	0.036	NA	0.787	NA	0.030	0.100	0.452	0.838	0.179	0.662	0.027

*NA in the table indicates that SDMbench did not provide the PAS metric results for that method on the given dataset, or that the method (SpaGCN (HE)) is not applicable to the specific spatial transcriptomics technology dataset.

Supplementary Table 13

Normalized rank scores of SAGE and 16 baseline methods based on PAS, reflecting performance ranks (0–1 scale, higher is better) across 34 datasets.

Data Name	STAMP	GASTON	STAGATE	stLearn	PROST	SEDR	GraphST	BASS	BayesSpace	louvain	SpaGCN (HE)	SCAN-IT	CCST	SpaGCN	leiden	SpaceFlow	conST	SAGE
Data_1	0.18	0.82	0.35	0.41	1	0.76	0.53	0.65	0.59	0.06	0.24	0.88	0.94	0.29	0	0.47	0.12	0.71
Data_2	0.12	0.82	0.35	0.47	0.71	0.88	0.41	0.59	0.65	0	0.18	0.76	0.94	0.29	0.06	0.53	0.24	1
Data_3	0.12	0.71	0.53	0.47	1	0.76	0.41	0.65	0.59	0.06	0.24	0.94	0.82	0.29	0	0.35	0.18	0.88
Data_4	0.24	1	0.47	0.35	0.76	0.82	0.41	0.65	0.59	0.06	0.18	0.88	0.94	0.29	0	0.53	0.12	0.71
Data_5	0.18	0.71	0.35	0.24	0.82	0.88	0.41	0.59	0.65	0.06	0.12	0.76	0.94	0.29	0	0.47	0.53	1
Data_6	0.12	0.76	0.53	0.29	1	0.94	0.47	0.59	0.71	0	0.18	0.88	0.82	0.35	0.06	0.41	0.24	0.65
Data_7	0.12	0.71	0.41	0.29	1	0.82	0.53	0.59	0.65	0	0.18	0.94	0.76	0.35	0.06	0.24	0.47	0.88
Data_8	0.12	0.82	0.41	0.35	1	0.59	0.53	0.71	0.65	0.06	0.24	0.88	0.76	0.47	0	0.29	0.18	0.94
Data_9	0.12	0.94	0.53	0.35	0.76	0.47	0.41	0.71	0.65	0.06	0.24	0.88	1	0.29	0	0.18	0.59	0.82
Data_10	0.06	0.76	0.53	0.41	0.82	0.59	0.47	0.71	0.65	0.12	0.24	0.88	1	0.29	0	0.18	0.35	0.94
Data_11	0.18	0.82	0.53	0.41	1	0.65	0.35	0.71	0.59	0.12	0.24	0.94	0.88	0.47	0	0.29	0.06	0.76
Data_12	0.12	0.88	0.41	0.35	1	0.71	0.47	0.65	0.59	0.06	0.18	0.82	0.94	0.29	0	0.24	0.53	0.76
Data_13	0.25	0.92	NA	NA	0.83	0.75	0.67	0.33	NA	0.08	NA	1	NA	0.42	0	0.17	0.58	0.5
Data_14	0.42	0.92	NA	NA	0.67	0.83	0.5	0.75	NA	0.17	NA	1	NA	0.33	0.08	0	0.58	0.25
Data_15	0.27	0.91	NA	NA	0.73	0.64	0.55	0.82	NA	0.09	NA	1	NA	0.36	0	0.18	NA	0.45
Data_16	0.25	0.92	NA	NA	0.67	0.83	0.58	0.75	NA	0.08	NA	1	NA	0.33	0.17	0	0.42	0.5
Data_17	0.25	0.92	NA	NA	0.67	0.83	0.58	0.75	NA	0.17	NA	1	NA	0.42	0.08	0	0.33	0.5
Data_18	0.3	0.9	NA	NA	0.6	0.8	NA	0.7	NA	0.2	NA	1	NA	0.5	0.1	0	NA	0.4
Data_19	0.4	0.9	NA	NA	0.6	0.8	NA	0.7	NA	0.1	NA	1	NA	0.5	0	0.3	NA	0.2
Data_20	0.44	0.89	NA	NA	0.67	NA	NA	0.78	NA	0.11	NA	1	NA	0.56	0.22	0	NA	0.33
Data_21	0.27	0.82	NA	NA	0.91	0.73	0.64	NA	NA	0	NA	1	NA	0.36	0.09	0.18	0.55	0.45
Data_22	0.36	0.79	0.29	NA	0.64	0.43	0.5	0.86	NA	0.07	NA	0.71	1	0.21	0.14	0.57	0	0.93
Data_23	0.29	0.79	0.5	NA	1	0.36	0.43	0.71	NA	0.07	NA	0.64	0.93	0.21	0.14	0.57	0	0.86
Data_24	0.31	0.85	0.38	NA	0.62	0.46	0.54	NA	NA	0.08	NA	0.92	1	0.23	0.15	0.69	0	0.77
Data_25	0.36	0.57	0.07	NA	0.93	0.43	0.5	0.71	NA	0.29	NA	0.86	1	0.14	0.21	0.79	0	0.64
Data_26	0.36	0.57	0.29	NA	0.86	0.43	0.5	0.79	NA	0.14	NA	0.93	1	0.21	0.07	0.71	0	0.64
Data_27	0.36	0.57	0.29	NA	0.86	0.43	0.5	0.93	NA	0.21	NA	0.79	1	0.14	0.07	0.71	0	0.64
Data_28	0.43	0.5	0.14	NA	0.79	0.36	0.64	0.71	NA	0.29	NA	0.86	1	0.07	0.21	0.93	0	0.57
Data_29	0.36	0.57	0.07	NA	0.86	0.43	0.64	0.93	NA	0.21	NA	0.71	1	0.14	0.29	0.79	0	0.5
Data_30	0.36	0.79	0.57	NA	1	0.07	0.5	0.93	NA	0.29	NA	0.71	0.43	0.14	0.21	0.64	0	0.86
Data_31	0.29	0.93	0.57	NA	1	0.14	0.43	0.71	NA	0.36	NA	0.79	0.5	0.21	0.07	0.64	0	0.86
Data_32	0.29	0.79	0.5	NA	1	0.14	0.43	0.57	NA	0.21	NA	0.86	0.64	0.36	0.07	0.71	0	0.93
Data_33	0.21	0.79	0.57	NA	1	0.14	0.43	0.71	NA	0.36	NA	0.93	0.5	0.29	0.07	0.64	0	0.86
Data_34	0.29	0.71	0.43	NA	1	0.21	0.5	0.79	NA	0.07	NA	0.86	0.64	0.36	0	0.57	0.14	0.93

*NA indicates missing values due to unavailability of the original metric or method incompatibility with specific datasets. The 'Mean score' row at the bottom represents the average normalized rank score for each method across all applicable datasets. A higher mean score indicates better overall performance under the given evaluation metric.

Supplementary Table 14

Comparison of SAGE with 16 state-of-the-art spatial transcriptomics domain analysis methods in terms of ASW

Data Name	STAMP	GASTON	STAGATE	stLearn	PROST	SEDR	GraphST	BASS	BayesSpace	louvain	SpaGCN (HE)	SCAN-IT	CCST	SpaGCN	leiden	SpaceFlow	conST	SAGE
Data_1	0.488	0.529	0.515	0.508	0.521	**0.545**	0.515	0.513	0.494	0.476	0.500	0.543	0.550	0.520	0.439	0.488	0.502	0.529
Data_2	0.490	0.521	0.508	0.493	0.559	**0.562**	0.505	0.533	0.526	0.470	0.504	0.534	0.514	0.525	0.470	0.507	0.497	0.521
Data_3	0.473	0.483	0.461	0.459	0.483	0.483	0.463	0.484	0.481	0.442	0.470	0.504	**0.545**	0.481	0.450	0.458	0.463	0.483
Data_4	0.480	0.452	0.488	0.452	0.518	0.484	0.465	0.476	0.477	0.433	0.467	0.540	**0.563**	0.479	0.444	0.458	0.478	0.452
Data_5	0.504	0.541	0.520	0.506	0.566	0.543	0.538	0.569	0.547	0.482	0.503	**0.602**	0.570	0.544	0.477	0.491	0.570	0.541
Data_6	0.511	0.540	0.538	0.537	0.530	0.551	0.527	0.548	0.537	0.477	0.519	**0.564**	0.548	0.539	0.483	0.471	0.537	0.540
Data_7	0.509	0.555	0.537	0.523	0.578	0.583	0.559	0.559	0.556	0.489	0.530	**0.617**	0.523	0.554	0.485	0.466	0.521	0.555
Data_8	0.513	0.553	0.541	0.527	**0.579**	0.568	0.564	0.560	0.556	0.483	0.525	0.561	0.564	0.546	0.487	0.466	0.500	0.553
Data_9	0.508	0.528	0.515	0.495	0.511	0.497	0.524	0.525	0.505	0.523	0.495	0.524	**0.644**	0.531	0.511	0.460	0.518	0.528
Data_10	0.488	0.540	0.538	0.527	0.559	0.526	0.513	0.557	0.583	0.517	0.516	0.608	**0.631**	0.548	0.507	0.469	0.507	0.540
Data_11	0.500	0.497	0.531	0.523	0.569	0.530	0.520	0.530	0.499	0.505	0.506	**0.605**	0.553	0.544	0.501	0.476	0.484	0.497
Data_12	0.498	0.514	0.521	0.516	0.552	**0.601**	0.527	0.544	0.545	0.494	0.508	0.529	0.570	0.531	0.494	0.456	0.507	0.514
Data_13	0.413	0.459	NA	NA	0.479	0.458	0.522	0.434	NA	0.373	NA	**0.593**	NA	0.491	0.352	0.433	0.441	0.459
Data_14	0.441	0.406	NA	NA	0.451	0.435	0.439	0.364	NA	0.434	NA	**0.505**	NA	0.456	0.405	0.379	0.416	0.406
Data_15	0.418	0.430	NA	NA	0.452	0.400	0.427	0.412	NA	0.434	NA	**0.507**	NA	0.432	0.411	0.404	NA	0.430
Data_16	0.416	0.420	NA	NA	0.449	0.470	0.412	0.467	NA	0.397	NA	0.476	NA	0.423	0.399	0.369	0.402	0.420
Data_17	0.447	0.400	NA	NA	0.428	0.380	0.428	0.433	NA	0.400	NA	**0.481**	NA	0.393	0.394	0.353	0.356	0.400
Data_18	0.402	0.433	NA	NA	0.411	0.357	NA	0.381	NA	0.407	NA	**0.499**	NA	0.374	0.397	0.347	NA	0.432
Data_19	0.395	0.463	NA	NA	0.396	0.390	NA	0.413	NA	0.450	NA	**0.476**	NA	0.390	0.444	0.355	NA	0.462
Data_20	0.375	0.426	NA	NA	0.369	NA	NA	0.299	NA	0.407	NA	**0.458**	NA	0.339	0.308	0.323	NA	0.425
Data_21	0.418	0.387	NA	NA	0.422	0.430	0.357	NA	NA	0.371	NA	**0.526**	NA	0.413	0.352	0.400	0.343	0.387
Data_22	0.492	0.553	0.479	NA	0.561	0.415	0.480	0.603	NA	0.467	NA	0.587	**0.593**	0.492	0.434	0.552	0.465	0.553
Data_23	0.481	0.571	0.491	NA	0.547	0.402	0.464	**0.575**	NA	0.448	NA	0.571	0.561	0.472	0.454	0.522	0.462	0.571
Data_24	0.450	0.568	0.435	NA	0.482	0.424	0.471	NA	NA	0.444	NA	0.557	**0.599**	0.453	0.439	0.511	0.459	0.568
Data_25	0.447	0.439	0.412	NA	0.461	0.475	0.432	0.477	NA	0.433	NA	0.464	**0.639**	0.419	0.394	0.469	0.446	0.439
Data_26	0.438	0.443	0.421	NA	0.424	0.404	0.468	0.486	NA	0.435	NA	0.458	**0.643**	0.422	0.410	0.459	0.445	0.443
Data_27	0.456	0.410	0.379	NA	0.430	0.416	0.442	0.491	NA	0.431	NA	0.506	**0.644**	0.407	0.430	0.478	0.443	0.410
Data_28	0.451	0.457	0.394	NA	0.448	0.484	0.421	0.499	NA	0.423	NA	0.514	**0.650**	0.410	0.423	0.494	0.455	0.457
Data_29	0.443	0.443	0.408	NA	0.457	0.420	0.422	0.506	NA	0.422	NA	0.513	**0.652**	0.410	0.422	0.529	0.459	0.443
Data_30	0.426	0.411	0.448	NA	0.486	0.407	0.447	0.457	NA	0.436	NA	0.498	0.432	0.413	0.438	0.451	0.418	0.411
Data_31	0.509	0.616	0.558	NA	0.591	0.490	0.537	**0.624**	NA	0.452	NA	0.601	0.515	0.528	0.449	0.554	0.474	0.616
Data_32	0.502	0.625	0.537	NA	0.570	0.508	0.553	0.543	NA	0.450	NA	0.594	0.542	0.510	0.445	0.517	0.473	**0.625**
Data_33	0.495	0.603	0.572	NA	0.574	0.465	0.492	**0.614**	NA	0.462	NA	0.580	0.539	0.500	0.457	0.549	0.470	0.603
Data_34	0.444	0.572	0.469	NA	0.557	0.437	0.509	0.571	NA	0.452	NA	**0.592**	0.543	0.460	0.423	0.511	0.439	0.572

*NA in the table indicates that SDMbench did not provide the ASW metric results for that method on the given dataset, or that the method (SpaGCN (HE)) is not applicable to the specific spatial transcriptomics technology dataset.

Supplementary Table 15

Normalized rank scores of SAGE and 16 baseline methods based on ASW, reflecting performance ranks (0–1 scale, higher is better) across 34 datasets.

Data Name	STAMP	GASTON	STAGATE	stLearn	PROST	SEDR	GraphST	BASS	BayesSpace	louvain	SpaGCN (HE)	SCAN-IT	CCST	SpaGCN	leiden	SpaceFlow	conST	SAGE
Data_1	0.18	0.82	0.53	0.41	0.71	0.94	0.59	0.47	0.24	0.06	0.29	0.88	1	0.65	0	0.12	0.35	0.76
Data_2	0.12	0.88	0.47	0.18	0.94	1	0.35	0.76	0.71	0.06	0.29	0.82	0.53	0.65	0	0.41	0.24	0.59
Data_3	0.53	0.24	0.29	0.18	0.82	0.76	0.41	0.88	0.59	0	0.47	0.94	1	0.65	0.06	0.12	0.35	0.71
Data_4	0.65	0.88	0.76	0.18	0.82	0.71	0.29	0.41	0.47	0	0.35	0.94	1	0.59	0.06	0.24	0.53	0.12
Data_5	0.24	0.71	0.35	0.29	0.76	0.53	0.41	0.82	0.65	0.06	0.18	1	0.94	0.59	0	0.12	0.88	0.47
Data_6	0.18	0.88	0.59	0.47	0.35	0.94	0.29	0.82	0.41	0.06	0.24	1	0.76	0.65	0.12	0	0.53	0.71
Data_7	0.18	0.82	0.47	0.35	0.88	0.94	0.71	0.76	0.65	0.12	0.41	1	0.29	0.53	0.06	0	0.24	0.59
Data_8	0.24	0.94	0.41	0.35	1	0.88	0.82	0.65	0.59	0.06	0.29	0.71	0.76	0.47	0.12	0	0.18	0.53
Data_9	0.29	0.35	0.53	0.12	0.47	0.18	0.76	0.82	0.24	0.65	0.06	0.71	1	0.94	0.41	0	0.59	0.88
Data_10	0.06	0.12	0.59	0.53	0.82	0.47	0.29	0.76	0.88	0.41	0.35	0.94	1	0.71	0.24	0	0.18	0.65
Data_11	0.24	0.94	0.71	0.53	0.88	0.65	0.47	0.59	0.18	0.35	0.41	1	0.82	0.76	0.29	0	0.06	0.12
Data_12	0.18	0.35	0.53	0.47	0.88	1	0.59	0.76	0.82	0.06	0.29	0.65	0.94	0.71	0.12	0	0.24	0.41
Data_13	0.17	0.92	NA	NA	0.67	0.5	0.83	0.33	NA	0.08	NA	1	NA	0.75	0	0.25	0.42	0.58
Data_14	0.67	0.92	NA	NA	0.75	0.5	0.58	0	NA	0.42	NA	1	NA	0.83	0.17	0.08	0.33	0.25
Data_15	0.36	1	NA	NA	0.82	0	0.45	0.27	NA	0.73	NA	0.91	NA	0.64	0.18	0.09	NA	0.55
Data_16	0.42	1	NA	NA	0.67	0.83	0.33	0.75	NA	0.08	NA	0.92	NA	0.58	0.17	0	0.25	0.5
Data_17	0.83	1	NA	NA	0.58	0.17	0.67	0.75	NA	0.5	NA	0.92	NA	0.25	0.33	0	0.08	0.42
Data_18	0.5	1	NA	NA	0.7	0.1	NA	0.3	NA	0.6	NA	0.9	NA	0.2	0.4	0	NA	0.8
Data_19	0.3	1	NA	NA	0.4	0.1	NA	0.5	NA	0.7	NA	0.9	NA	0.2	0.6	0	NA	0.8
Data_20	0.56	1	NA	NA	0.44	NA	NA	0	NA	0.67	NA	0.89	NA	0.33	0.11	0.22	NA	0.78
Data_21	0.64	0.82	NA	NA	0.73	0.91	0.18	NA	NA	0.27	NA	1	NA	0.55	0.09	0.45	0	0.36
Data_22	0.5	0.79	0.29	NA	0.71	0	0.36	1	NA	0.21	NA	0.86	0.93	0.43	0.07	0.57	0.14	0.64
Data_23	0.43	0.79	0.5	NA	0.64	0	0.29	1	NA	0.07	NA	0.86	0.71	0.36	0.14	0.57	0.21	0.93
Data_24	0.31	0.85	0.08	NA	0.62	0	0.54	NA	NA	0.23	NA	0.77	1	0.38	0.15	0.69	0.46	0.92
Data_25	0.57	0.21	0.07	NA	0.64	0.86	0.29	0.93	NA	0.36	NA	0.71	1	0.14	0	0.79	0.5	0.43
Data_26	0.5	0.29	0.14	NA	0.36	0	0.86	0.93	NA	0.43	NA	0.71	1	0.21	0.07	0.79	0.64	0.57
Data_27	0.64	0.71	0	NA	0.29	0.21	0.5	0.86	NA	0.43	NA	0.93	1	0.07	0.36	0.79	0.57	0.14
Data_28	0.43	0.64	0	NA	0.36	0.71	0.14	0.86	NA	0.21	NA	0.93	1	0.07	0.29	0.79	0.5	0.57
Data_29	0.5	0.71	0	NA	0.57	0.14	0.21	0.79	NA	0.29	NA	0.86	1	0.07	0.36	0.93	0.64	0.43
Data_30	0.29	0.86	0.64	NA	0.93	0	0.57	0.79	NA	0.43	NA	1	0.36	0.14	0.5	0.71	0.21	0.07
Data_31	0.29	0.86	0.64	NA	0.71	0.21	0.5	1	NA	0.07	NA	0.79	0.36	0.43	0	0.57	0.14	0.93
Data_32	0.21	0.93	0.5	NA	0.79	0.29	0.71	0.64	NA	0.07	NA	0.86	0.57	0.36	0	0.43	0.14	1
Data_33	0.36	0.86	0.64	NA	0.71	0.14	0.29	1	NA	0.07	NA	0.79	0.5	0.43	0	0.57	0.21	0.93
Data_34	0.21	0.79	0.43	NA	0.71	0.07	0.5	0.86	NA	0.29	NA	1	0.64	0.36	0	0.57	0.14	0.93

* NA indicates missing values due to unavailability of the original metric or method incompatibility with specific datasets. The 'Mean score' row at the bottom represents the average normalized rank score for each method across all applicable datasets. A higher mean score indicates better overall performance under the given evaluation metric.

Supplementary Table 16

Comparison of methods based on mean rank scores of spatial consistency metrics (CHAOS, PAS, ASW)

Data Name	STAMP	GASTON	STAGATE	stLearn	PROST	SEDR	GraphST	BASS	BayesSpace	louvain	SpaGCN (HE)	SCAN-IT	CCST	SpaGCN	leiden	SpaceFlow	conST	SAGE
Data_1	0.14	0.8	0.43	0.39	0.86	0.72	0.59	0.55	0.51	0.08	0.24	0.9	0.98	0.41	0	0.39	0.24	0.76
Data_2	0.12	0.86	0.43	0.33	0.77	0.82	0.39	0.63	0.71	0.02	0.22	0.84	0.76	0.39	0.04	0.55	0.26	0.86
Data_3	0.26	0.59	0.45	0.33	0.92	0.66	0.41	0.73	0.63	0.02	0.3	0.88	0.9	0.41	0.04	0.35	0.26	0.86
Data_4	0.34	0.96	0.59	0.29	0.78	0.8	0.37	0.55	0.51	0.04	0.24	0.92	0.92	0.39	0.02	0.49	0.3	0.49
Data_5	0.18	0.79	0.35	0.26	0.82	0.65	0.47	0.69	0.67	0.06	0.16	0.84	0.9	0.39	0	0.35	0.61	0.82
Data_6	0.14	0.88	0.59	0.37	0.74	0.8	0.39	0.63	0.61	0.04	0.2	0.94	0.8	0.41	0.06	0.33	0.35	0.71
Data_7	0.14	0.76	0.49	0.31	0.96	0.82	0.59	0.67	0.59	0.04	0.26	0.96	0.62	0.37	0.06	0.2	0.37	0.78
Data_8	0.16	0.88	0.41	0.31	0.98	0.69	0.63	0.69	0.63	0.04	0.24	0.8	0.76	0.43	0.06	0.25	0.22	0.82
Data_9	0.2	0.76	0.61	0.27	0.7	0.35	0.57	0.67	0.53	0.26	0.14	0.84	0.94	0.49	0.14	0.16	0.61	0.76
Data_10	0.06	0.61	0.55	0.41	0.78	0.51	0.45	0.76	0.73	0.22	0.26	0.9	0.92	0.41	0.08	0.18	0.31	0.86
Data_11	0.18	0.84	0.57	0.45	0.9	0.65	0.47	0.67	0.43	0.22	0.3	0.94	0.9	0.51	0.12	0.21	0.04	0.61
Data_12	0.16	0.72	0.51	0.41	0.88	0.81	0.51	0.69	0.59	0.04	0.2	0.78	0.96	0.41	0.06	0.18	0.43	0.66
Data_13	0.25	0.92	NA	NA	0.69	0.67	0.78	0.44	NA	0.05	NA	1	NA	0.47	0.03	0.31	0.39	0.5
Data_14	0.39	0.89	NA	NA	0.61	0.75	0.58	0.5	NA	0.28	NA	1	NA	0.5	0.14	0.03	0.47	0.36
Data_15	0.3	0.94	NA	NA	0.7	0.49	0.55	0.61	NA	0.3	NA	0.97	NA	0.45	0.06	0.15	NA	0.48
Data_16	0.31	0.95	NA	NA	0.61	0.75	0.53	0.78	NA	0.08	NA	0.97	NA	0.41	0.17	0	0.36	0.58
Data_17	0.42	0.92	NA	NA	0.67	0.64	0.56	0.72	NA	0.31	NA	0.97	NA	0.33	0.16	0	0.3	0.5
Data_18	0.37	0.93	NA	NA	0.6	0.57	NA	0.57	NA	0.33	NA	0.97	NA	0.37	0.2	0	NA	0.6
Data_19	0.3	0.97	NA	NA	0.57	0.57	NA	0.6	NA	0.3	NA	0.93	NA	0.33	0.2	0.23	NA	0.5
Data_20	0.44	0.93	NA	NA	0.59	NA	NA	0.52	NA	0.3	NA	0.96	NA	0.44	0.18	0.07	NA	0.56
Data_21	0.39	0.82	NA	NA	0.85	0.73	0.52	NA	NA	0.09	NA	1	NA	0.42	0.09	0.27	0.33	0.48
Data_22	0.43	0.81	0.31	NA	0.76	0.21	0.45	0.86	NA	0.14	NA	0.74	0.98	0.31	0.09	0.57	0.05	0.79
Data_23	0.36	0.84	0.48	NA	0.81	0.19	0.41	0.78	NA	0.09	NA	0.74	0.88	0.29	0.12	0.57	0.07	0.88
Data_24	0.33	0.85	0.31	NA	0.62	0.2	0.54	NA	NA	0.1	NA	0.87	1	0.31	0.18	0.69	0.18	0.82
Data_25	0.45	0.5	0.12	NA	0.71	0.53	0.43	0.76	NA	0.26	NA	0.79	1	0.21	0.09	0.81	0.17	0.67
Data_26	0.43	0.57	0.26	NA	0.72	0.24	0.62	0.76	NA	0.24	NA	0.78	1	0.21	0.07	0.76	0.21	0.62
Data_27	0.48	0.71	0.22	NA	0.65	0.28	0.5	0.79	NA	0.26	NA	0.79	1	0.17	0.17	0.74	0.19	0.57
Data_28	0.43	0.67	0.14	NA	0.62	0.48	0.57	0.69	NA	0.19	NA	0.79	1	0.12	0.21	0.79	0.17	0.64
Data_29	0.41	0.71	0.09	NA	0.79	0.33	0.45	0.84	NA	0.21	NA	0.74	1	0.17	0.24	0.76	0.21	0.55
Data_30	0.41	0.84	0.62	NA	0.95	0.05	0.59	0.84	NA	0.34	NA	0.71	0.43	0.14	0.31	0.57	0.07	0.64
Data_31	0.31	0.91	0.59	NA	0.9	0.14	0.45	0.81	NA	0.24	NA	0.81	0.45	0.28	0.07	0.62	0.05	0.86
Data_32	0.26	0.86	0.48	NA	0.93	0.17	0.57	0.62	NA	0.16	NA	0.84	0.57	0.36	0.07	0.62	0.05	0.95
Data_33	0.26	0.84	0.59	NA	0.88	0.12	0.41	0.81	NA	0.26	NA	0.84	0.48	0.34	0.07	0.62	0.07	0.93
Data_34	0.26	0.83	0.43	NA	0.86	0.14	0.5	0.81	NA	0.19	NA	0.86	0.64	0.36	0	0.57	0.12	0.93

* NA indicates missing values due to unavailability of the original metric or method incompatibility with specific datasets. Spatial consistency evaluation based on the mean normalized rank scores of CHAOS, PAS, and ASW. These metrics reflect the structural compactness and spatial coherence of clusters. For each dataset-method pair, the consistency score is the average of rank scores across the three metrics. Higher values represent better spatial organization.

Supplementary Table 17

Comparison of SAGE with 16 state-of-the-art spatial transcriptomics domain analysis methods in terms of Moran’s I (MRI)

Data Name	STAMP	GASTON	STAGATE	stLearn	PROST	SEDR	GraphST	BASS	BayesSpace	louvain	SpaGCN (HE)	SCAN-IT	CCST	SpaGCN	leiden	SpaceFlow	conST	SAGE
Data_1	0.431	0.431	0.336	0.278	**0.446**	0.374	0.268	0.331	0.278	0.335	0.305	0.310	0.314	0.296	0.335	0.294	0.328	0.430
Data_2	0.448	0.448	0.336	0.249	**0.453**	0.349	0.313	0.255	0.343	0.336	0.301	0.292	0.275	0.282	0.289	0.333	0.339	0.368
Data_3	0.381	0.381	0.311	0.342	**0.413**	0.378	0.307	0.342	0.304	0.304	0.318	0.303	0.361	0.310	0.316	0.307	0.311	0.369
Data_4	0.333	0.333	0.288	0.288	**0.403**	0.354	0.299	0.285	0.279	0.298	0.312	0.295	0.316	0.303	0.306	0.274	0.307	0.345
Data_5	0.611	0.611	0.303	0.299	0.354	0.307	0.262	0.276	0.235	0.249	0.246	0.261	0.250	0.272	0.237	0.235	0.281	**0.370**
Data_6	0.552	0.552	0.237	0.269	0.351	0.285	0.253	0.236	0.252	0.236	0.240	0.238	0.203	0.245	0.252	0.229	0.221	**0.370**
Data_7	0.388	0.388	0.359	0.268	0.314	**0.391**	0.359	0.354	0.326	0.380	0.357	0.357	0.287	0.357	0.359	0.282	0.335	0.317
Data_8	0.251	0.251	0.298	0.281	0.302	0.340	0.248	0.259	0.283	0.334	0.265	0.277	0.260	0.264	**0.357**	0.266	0.285	0.281
Data_9	0.444	0.444	0.401	0.403	0.508	0.399	0.380	0.387	0.380	**0.466**	**0.466**	0.385	0.441	0.397	0.392	0.375	0.444	0.444
Data_10	0.531	0.531	0.473	0.455	**0.551**	0.468	0.445	0.477	0.477	0.455	0.434	0.478	0.465	0.460	0.455	0.429	0.475	0.537
Data_11	0.539	0.539	0.400	0.374	0.376	0.433	0.401	0.399	0.467	0.442	0.373	0.409	0.310	0.385	0.442	0.306	0.439	**0.491**
Data_12	0.491	0.491	0.392	0.389	0.495	**0.510**	0.346	0.346	0.360	0.447	0.374	0.396	0.300	0.379	0.447	0.329	0.441	0.495
Data_13	0.539	0.539	NA	NA	0.554	0.552	0.504	0.523	NA	0.513	NA	0.520	NA	0.507	0.529	0.520	**0.561**	0.539
Data_14	0.608	0.608	NA	NA	**0.611**	0.451	0.354	0.426	NA	0.672	NA	0.385	NA	0.366	0.354	0.268	0.535	0.608
Data_15	0.426	0.426	NA	NA	**0.559**	0.323	0.286	0.345	NA	0.289	NA	0.292	NA	0.298	0.273	0.268	NA	0.426
Data_16	0.594	0.594	NA	NA	0.603	0.634	0.639	0.556	NA	0.682	NA	0.583	NA	0.641	**0.667**	0.544	0.637	0.594
Data_17	0.545	0.545	NA	NA	**0.573**	0.370	0.369	0.295	NA	0.406	NA	0.274	NA	0.295	0.263	0.258	0.445	0.410
Data_18	0.610	0.610	NA	NA	0.619	0.285	NA	0.263	NA	0.272	NA	0.264	NA	0.273	0.250	0.254	NA	**0.635**
Data_19	0.516	0.516	NA	NA	0.629	0.356	NA	0.244	NA	0.349	NA	0.294	NA	0.333	0.359	0.266	NA	**0.642**
Data_20	0.581	0.581	NA	NA	0.594	NA	NA	0.306	NA	0.232	NA	0.309	NA	0.303	0.301	0.247	NA	**0.615**
Data_21	0.430	0.430	NA	NA	0.448	0.497	0.490	NA	NA	0.542	NA	0.447	NA	0.442	**0.539**	0.414	0.468	0.519
Data_22	0.094	0.094	0.075	NA	0.092	0.044	0.094	0.107	NA	0.035	NA	0.108	0.103	0.069	0.044	0.071	0.059	0.122
Data_23	0.076	0.076	0.097	NA	0.078	0.057	0.080	0.087	NA	0.078	NA	0.098	0.086	0.080	0.078	0.066	0.048	**0.154**
Data_24	0.061	0.062	0.078	NA	0.070	0.032	0.054	NA	NA	0.048	NA	0.053	0.051	0.057	0.048	0.075	0.037	**0.083**
Data_25	0.216	0.216	0.192	NA	0.206	0.181	0.205	0.230	NA	0.157	NA	0.206	0.192	0.191	0.169	0.240	0.196	**0.246**
Data_26	0.211	0.211	0.232	NA	0.215	0.217	0.259	**0.271**	NA	0.137	NA	0.234	0.213	0.185	0.152	0.238	0.209	0.242
Data_27	0.222	0.222	0.229	NA	0.222	0.201	0.239	**0.241**	NA	0.167	NA	0.215	0.194	0.198	0.153	0.207	0.203	0.225
Data_28	0.209	0.209	0.203	NA	0.206	0.181	0.215	**0.245**	NA	0.165	NA	0.199	0.190	0.181	0.165	0.236	0.201	0.181
Data_29	0.214	0.214	0.172	NA	0.208	0.187	0.222	**0.245**	NA	0.147	NA	0.194	0.193	0.161	0.147	0.212	0.186	0.220
Data_30	0.191	0.191	0.207	NA	0.190	0.167	0.187	0.171	NA	0.215	NA	0.251	0.188	0.183	0.189	0.189	0.173	**0.305**
Data_31	0.175	0.175	0.198	NA	0.181	0.141	0.211	0.202	NA	0.157	NA	0.189	0.207	0.192	0.159	0.152	0.103	**0.262**
Data_32	0.180	0.180	0.236	NA	0.185	0.215	0.236	**0.242**	NA	0.227	NA	0.200	0.230	0.236	0.186	0.196	0.207	0.221
Data_33	0.148	0.148	0.224	NA	0.167	0.154	0.224	0.214	NA	0.132	NA	0.190	0.211	0.221	0.132	0.197	0.114	**0.231**
Data_34	0.194	0.194	0.229	NA	0.184	0.187	0.255	0.245	NA	0.186	NA	0.253	0.220	0.192	0.155	0.180	0.187	**0.277**

*NA in the table indicates that SDMbench did not provide the MRI metric results for that method on the given dataset, or that the method (SpaGCN (HE)) is not applicable to the specific spatial transcriptomics technology dataset.

Supplementary Table 18

Normalized rank scores of SAGE and 16 baseline methods based on Moran's I (MRI), reflecting performance ranks (0–1 scale, higher is better) across 34 datasets.

Data Name	STAMP	GASTON	STAGATE	stLearn	PROST	SEDR	GraphST	BASS	BayesSpace	louvain	SpaGCN (HE)	SCAN-IT	CCST	SpaGCN	leiden	SpaceFlow	conST	SAGE
Data_1	0.88	0.94	0.71	0.12	1	0.76	0	0.53	0.06	0.65	0.29	0.35	0.41	0.24	0.59	0.18	0.47	0.82
Data_2	0.88	0.94	0.53	0	1	0.76	0.41	0.06	0.71	0.59	0.35	0.29	0.12	0.18	0.24	0.47	0.65	0.82
Data_3	0.88	0.94	0.41	0.65	1	0.82	0.18	0.59	0.12	0.06	0.53	0	0.71	0.29	0.47	0.24	0.35	0.76
Data_4	0.76	0.82	0.18	0.24	1	0.94	0.41	0.12	0.06	0.35	0.65	0.29	0.71	0.47	0.53	0	0.59	0.88
Data_5	0.94	1	0.71	0.65	0.82	0.76	0.41	0.53	0	0.24	0.18	0.35	0.29	0.47	0.12	0.06	0.59	0.88
Data_6	0.94	1	0.29	0.71	0.82	0.76	0.65	0.18	0.59	0.24	0.41	0.35	0	0.47	0.53	0.12	0.06	0.88
Data_7	0.88	0.94	0.65	0	0.18	1	0.76	0.41	0.29	0.82	0.47	0.59	0.12	0.53	0.71	0.06	0.35	0.24
Data_8	0.06	0.12	0.76	0.53	0.82	0.94	0	0.18	0.65	0.88	0.35	0.47	0.24	0.29	1	0.41	0.71	0.59
Data_9	0.76	0.82	0.47	0.53	1	0.41	0.12	0.24	0.06	0.88	0.94	0.18	0.59	0.35	0.29	0	0.65	0.71
Data_10	0.82	0.88	0.53	0.29	1	0.47	0.12	0.71	0.65	0.18	0.06	0.76	0.41	0.35	0.24	0	0.59	0.94
Data_11	0.94	1	0.41	0.18	0.24	0.59	0.47	0.35	0.82	0.76	0.12	0.53	0.06	0.29	0.71	0	0.65	0.88
Data_12	0.76	0.82	0.47	0.41	0.94	1	0.18	0.12	0.24	0.71	0.29	0.53	0	0.35	0.65	0.06	0.59	0.88
Data_13	0.67	0.75	NA	NA	0.92	0.83	0	0.42	NA	0.17	NA	0.25	NA	0.08	0.5	0.33	1	0.58
Data_14	0.75	0.83	NA	NA	0.92	0.5	0.17	0.42	NA	1	NA	0.33	NA	0.25	0.08	0	0.58	0.67
Data_15	0.82	0.91	NA	NA	1	0.55	0.18	0.64	NA	0.27	NA	0.36	NA	0.45	0.09	0	NA	0.73
Data_16	0.33	0.42	NA	NA	0.5	0.58	0.75	0.08	NA	1	NA	0.17	NA	0.83	0.92	0	0.67	0.25
Data_17	0.83	0.92	NA	NA	1	0.5	0.42	0.25	NA	0.58	NA	0.17	NA	0.33	0.08	0	0.75	0.67
Data_18	0.7	0.8	NA	NA	0.9	0.6	NA	0.2	NA	0.4	NA	0.3	NA	0.5	0	0.1	NA	1
Data_19	0.7	0.8	NA	NA	0.9	0.5	NA	0	NA	0.4	NA	0.2	NA	0.3	0.6	0.1	NA	1
Data_20	0.67	0.78	NA	NA	0.89	NA	NA	0.44	NA	0	NA	0.56	NA	0.33	0.22	0.11	NA	1
Data_21	0.09	0.18	NA	NA	0.45	0.73	0.64	NA	NA	1	NA	0.36	NA	0.27	0.91	0	0.55	0.82
Data_22	0.57	0.64	0.43	NA	0.5	0.14	0.71	0.86	NA	0	NA	0.93	0.79	0.29	0.07	0.36	0.21	1
Data_23	0.21	0.29	0.86	NA	0.5	0.07	0.64	0.79	NA	0.43	NA	0.93	0.71	0.57	0.36	0.14	0	1
Data_24	0.62	0.69	0.92	NA	0.77	0	0.46	NA	NA	0.15	NA	0.38	0.31	0.54	0.23	0.85	0.08	1
Data_25	0.71	0.79	0.36	NA	0.57	0.14	0.5	0.86	NA	0	NA	0.64	0.29	0.21	0.07	0.93	0.43	1
Data_26	0.29	0.36	0.64	NA	0.5	0.57	0.93	1	NA	0	NA	0.71	0.43	0.14	0.07	0.79	0.21	0.86
Data_27	0.57	0.71	0.86	NA	0.64	0.29	0.93	1	NA	0.07	NA	0.5	0.14	0.21	0	0.43	0.36	0.79
Data_28	0.71	0.79	0.57	NA	0.64	0.14	0.86	1	NA	0.07	NA	0.43	0.36	0.21	0	0.93	0.5	0.29
Data_29	0.71	0.79	0.21	NA	0.57	0.36	0.93	1	NA	0.07	NA	0.5	0.43	0.14	0	0.64	0.29	0.86
Data_30	0.64	0.71	0.79	NA	0.57	0	0.29	0.07	NA	0.86	NA	0.93	0.36	0.21	0.5	0.43	0.14	1
Data_31	0.36	0.43	0.71	NA	0.5	0.07	0.93	0.79	NA	0.21	NA	0.57	0.86	0.64	0.29	0.14	0	1
Data_32	0	0.07	0.93	NA	0.14	0.5	0.86	1	NA	0.64	NA	0.36	0.71	0.79	0.21	0.29	0.43	0.57
Data_33	0.21	0.29	0.93	NA	0.43	0.36	0.86	0.71	NA	0.14	NA	0.5	0.64	0.79	0.07	0.57	0	1
Data_34	0.5	0.57	0.71	NA	0.14	0.29	0.93	0.79	NA	0.21	NA	0.86	0.64	0.43	0	0.07	0.36	1

*NA indicates missing values due to unavailability of the original metric or method incompatibility with specific datasets. The 'Mean score' row at the bottom represents the average normalized rank score for each method across all applicable datasets. A higher mean score indicates better overall performance under the given evaluation metric.

Supplementary Table 19

Comparison of SAGE with 16 state-of-the-art spatial transcriptomics domain analysis methods in terms of Geary’s C (GC)

Data Name	STAMP	GASTON	STAGATE	stLearn	PROST	SEDR	GraphST	BASS	BayesSpace	louvain	SpaGCN (HE)	SCAN-IT	CCST	SpaGCN	leiden	SpaceFlow	conST	SAGE
Data_1	0.567	0.567	0.664	0.722	**0.555**	0.626	0.733	0.670	0.723	0.666	0.694	0.691	0.686	0.704	0.666	0.706	0.672	0.568
Data_2	0.553	0.553	0.662	0.751	**0.545**	0.648	0.687	0.745	0.655	0.663	0.698	0.707	0.725	0.718	0.710	0.666	0.660	0.634
Data_3	0.620	0.620	0.689	0.658	**0.588**	0.623	0.695	0.658	0.697	0.697	0.681	0.698	0.639	0.691	0.684	0.694	0.690	0.631
Data_4	0.665	0.665	0.713	0.710	**0.597**	0.646	0.702	0.716	0.721	0.704	0.688	0.705	0.684	0.699	0.695	0.726	0.694	0.654
Data_5	0.389	0.389	0.694	0.698	0.647	0.692	0.737	0.722	0.765	0.750	0.753	0.737	0.748	0.726	0.762	0.764	0.718	**0.630**
Data_6	0.447	0.447	0.763	0.733	0.650	0.714	0.748	0.765	0.747	0.765	0.761	0.762	0.797	0.756	0.747	0.773	0.779	**0.631**
Data_7	0.612	0.612	0.641	0.732	0.687	**0.610**	0.642	0.646	0.676	0.622	0.645	0.644	0.714	0.644	0.642	0.719	0.666	0.679
Data_8	0.750	0.750	0.703	0.721	0.697	0.663	0.752	0.741	0.716	0.668	0.735	0.724	0.739	0.736	**0.648**	0.735	0.716	0.719
Data_9	0.558	0.558	0.597	0.595	**0.492**	0.599	0.621	0.613	0.619	0.533	0.533	0.615	0.557	0.602	0.605	0.623	0.554	0.558
Data_10	0.473	0.473	0.529	0.544	**0.450**	0.532	0.555	0.519	0.522	0.544	0.564	0.521	0.535	0.539	0.544	0.570	0.526	0.471
Data_11	0.462	0.462	0.595	0.627	0.624	0.568	0.595	0.598	0.532	0.555	0.625	0.589	0.688	0.614	0.555	0.691	0.556	**0.509**
Data_12	0.509	0.509	0.608	0.612	0.506	**0.490**	0.656	0.656	0.636	0.553	0.625	0.602	0.697	0.620	0.553	0.671	0.558	0.506
Data_13	0.452	0.452	NA	NA	**0.436**	0.449	0.495	0.479	NA	0.491	NA	0.482	NA	0.493	0.474	0.481	0.440	0.452
Data_14	0.379	0.379	NA	NA	0.377	0.556	0.649	0.580	NA	**0.332**	NA	0.620	NA	0.640	0.654	0.741	0.465	0.379
Data_15	0.567	0.567	NA	NA	0.442	0.679	0.719	0.656	NA	0.716	NA	0.711	NA	0.704	0.727	0.734	NA	0.567
Data_16	0.412	0.412	NA	NA	0.406	0.366	0.361	0.444	NA	**0.319**	NA	0.418	NA	0.359	0.334	0.457	0.363	0.412
Data_17	0.449	0.449	NA	NA	0.425	0.634	0.629	0.705	NA	0.599	NA	0.731	NA	0.708	0.749	0.750	0.559	0.585
Data_18	0.387	0.387	NA	NA	0.379	0.717	NA	0.742	NA	0.731	NA	0.740	NA	0.729	0.757	0.752	NA	**0.356**
Data_19	0.477	0.477	NA	NA	0.367	0.645	NA	0.756	NA	0.652	NA	0.707	NA	0.669	0.641	0.736	NA	**0.346**
Data_20	0.424	0.424	NA	NA	0.406	NA	NA	0.694	NA	0.775	NA	0.697	NA	0.702	0.700	0.762	NA	**0.384**
Data_21	0.567	0.567	NA	NA	0.546	0.501	0.508	NA	NA	0.458	NA	0.552	NA	0.557	**0.461**	0.587	0.531	0.476
Data_22	0.895	0.895	0.923	NA	0.902	0.949	0.915	0.893	NA	0.961	NA	0.892	0.896	0.929	0.949	0.926	0.940	**0.883**
Data_23	0.905	0.905	0.902	NA	0.905	0.943	0.918	0.899	NA	0.920	NA	0.898	0.907	0.918	0.920	0.928	0.950	**0.842**
Data_24	0.916	0.916	0.919	NA	0.920	0.970	0.942	NA	NA	0.950	NA	0.944	0.946	0.940	0.948	0.926	0.957	**0.915**
Data_25	0.794	0.794	0.807	NA	0.799	0.817	0.793	0.767	NA	0.842	NA	0.793	0.807	0.809	0.831	0.761	0.803	**0.758**
Data_26	0.787	0.787	0.764	NA	0.799	0.780	0.738	**0.726**	NA	0.860	NA	0.764	0.789	0.813	0.844	0.759	0.787	0.755
Data_27	0.783	0.783	0.770	NA	0.791	0.797	0.757	**0.756**	NA	0.831	NA	0.783	0.805	0.799	0.846	0.793	0.796	0.778
Data_28	0.801	0.801	0.793	NA	0.804	0.819	0.782	**0.753**	NA	0.834	NA	0.803	0.808	0.816	0.834	0.761	0.797	0.820
Data_29	0.795	0.795	0.826	NA	0.799	0.810	0.779	0.756	NA	0.849	NA	0.804	0.804	0.837	0.849	0.784	0.811	0.781
Data_30	0.803	0.803	0.788	NA	0.807	0.825	0.810	0.822	NA	0.778	NA	0.742	0.807	0.814	0.810	0.804	0.822	**0.705**
Data_31	0.857	0.857	0.799	NA	0.816	0.858	0.789	0.799	NA	0.832	NA	0.807	0.791	0.802	0.832	0.846	0.900	**0.752**
Data_32	0.802	0.802	**0.764**	NA	0.800	0.782	0.765	0.754	NA	0.769	NA	0.796	0.771	0.765	0.814	0.800	0.791	0.774
Data_33	0.835	0.835	0.771	NA	0.820	0.844	0.771	0.785	NA	0.867	NA	0.809	0.786	0.782	0.867	0.803	0.886	**0.767**
Data_34	0.786	0.786	0.769	NA	0.806	0.811	0.743	0.753	NA	0.814	NA	0.744	0.779	0.804	0.833	0.816	0.811	**0.725**

*NA in the table indicates that SDMbench did not provide the GC metric results for that method on the given dataset, or that the method (SpaGCN (HE)) is not applicable to the specific spatial transcriptomics technology dataset.

Supplementary Table 20

Normalized rank scores of SAGE and 16 baseline methods based on Geary's C (GC), reflecting performance ranks (0–1 scale, higher is better) across 34 datasets.

Data Name	STAMP	GASTON	STAGATE	stLearn	PROST	SEDR	GraphST	BASS	BayesSpace	louvain	SpaGCN (HE)	SCAN-IT	CCST	SpaGCN	leiden	SpaceFlow	conST	SAGE
Data_1	0.88	0.94	0.71	0.12	1	0.76	0	0.53	0.06	0.65	0.29	0.35	0.41	0.24	0.59	0.18	0.47	0.82
Data_2	0.88	0.94	0.59	0	1	0.76	0.41	0.06	0.71	0.53	0.35	0.29	0.12	0.18	0.24	0.47	0.65	0.82
Data_3	0.88	0.94	0.41	0.65	1	0.82	0.18	0.59	0.12	0.06	0.53	0	0.71	0.29	0.47	0.24	0.35	0.76
Data_4	0.76	0.82	0.18	0.24	1	0.94	0.41	0.12	0.06	0.35	0.65	0.29	0.71	0.47	0.53	0	0.59	0.88
Data_5	0.94	1	0.71	0.65	0.82	0.76	0.35	0.53	0	0.24	0.18	0.41	0.29	0.47	0.12	0.06	0.59	0.88
Data_6	0.94	1	0.29	0.71	0.82	0.76	0.53	0.18	0.65	0.24	0.41	0.35	0	0.47	0.59	0.12	0.06	0.88
Data_7	0.88	0.94	0.76	0	0.18	1	0.71	0.41	0.29	0.82	0.47	0.53	0.12	0.59	0.65	0.06	0.35	0.24
Data_8	0.06	0.12	0.76	0.53	0.82	0.94	0	0.18	0.71	0.88	0.41	0.47	0.24	0.29	1	0.35	0.65	0.59
Data_9	0.65	0.71	0.47	0.53	1	0.41	0.06	0.24	0.12	0.88	0.94	0.18	0.76	0.35	0.29	0	0.82	0.59
Data_10	0.82	0.88	0.53	0.29	1	0.47	0.12	0.76	0.65	0.24	0.06	0.71	0.41	0.35	0.18	0	0.59	0.94
Data_11	0.94	1	0.41	0.12	0.24	0.59	0.47	0.35	0.82	0.76	0.18	0.53	0.06	0.29	0.71	0	0.65	0.88
Data_12	0.76	0.82	0.47	0.41	0.94	1	0.18	0.12	0.24	0.71	0.29	0.53	0	0.35	0.65	0.06	0.59	0.88
Data_13	0.67	0.75	NA	NA	1	0.83	0	0.42	NA	0.17	NA	0.25	NA	0.08	0.5	0.33	0.92	0.58
Data_14	0.75	0.83	NA	NA	0.92	0.5	0.17	0.42	NA	1	NA	0.33	NA	0.25	0.08	0	0.58	0.67
Data_15	0.82	0.91	NA	NA	1	0.55	0.18	0.64	NA	0.27	NA	0.36	NA	0.45	0.09	0	NA	0.73
Data_16	0.33	0.42	NA	NA	0.5	0.58	0.75	0.08	NA	1	NA	0.17	NA	0.83	0.92	0	0.67	0.25
Data_17	0.83	0.92	NA	NA	1	0.42	0.5	0.33	NA	0.58	NA	0.17	NA	0.25	0.08	0	0.75	0.67
Data_18	0.7	0.8	NA	NA	0.9	0.6	NA	0.2	NA	0.4	NA	0.3	NA	0.5	0	0.1	NA	1
Data_19	0.7	0.8	NA	NA	0.9	0.5	NA	0	NA	0.4	NA	0.2	NA	0.3	0.6	0.1	NA	1
Data_20	0.67	0.78	NA	NA	0.89	NA	NA	0.56	NA	0	NA	0.44	NA	0.22	0.33	0.11	NA	1
Data_21	0.09	0.18	NA	NA	0.45	0.73	0.64	NA	NA	1	NA	0.36	NA	0.27	0.91	0	0.55	0.82
Data_22	0.71	0.79	0.43	NA	0.57	0.14	0.5	0.86	NA	0	NA	0.93	0.64	0.29	0.07	0.36	0.21	1
Data_23	0.57	0.64	0.79	NA	0.71	0.07	0.43	0.86	NA	0.29	NA	0.93	0.5	0.36	0.21	0.14	0	1
Data_24	0.85	0.92	0.77	NA	0.69	0	0.46	NA	NA	0.15	NA	0.38	0.31	0.54	0.23	0.62	0.08	1
Data_25	0.57	0.64	0.36	NA	0.5	0.14	0.71	0.86	NA	0	NA	0.79	0.29	0.21	0.07	0.93	0.43	1
Data_26	0.36	0.43	0.64	NA	0.21	0.57	0.93	1	NA	0	NA	0.71	0.29	0.14	0.07	0.79	0.5	0.86
Data_27	0.57	0.64	0.86	NA	0.5	0.29	0.93	1	NA	0.07	NA	0.71	0.14	0.21	0	0.43	0.36	0.79
Data_28	0.57	0.64	0.79	NA	0.43	0.21	0.86	1	NA	0.07	NA	0.5	0.36	0.29	0	0.93	0.71	0.14
Data_29	0.64	0.71	0.21	NA	0.57	0.36	0.93	1	NA	0.07	NA	0.5	0.43	0.14	0	0.79	0.29	0.86
Data_30	0.64	0.71	0.79	NA	0.5	0	0.36	0.14	NA	0.86	NA	0.93	0.43	0.21	0.29	0.57	0.07	1
Data_31	0.14	0.21	0.71	NA	0.5	0.07	0.93	0.79	NA	0.43	NA	0.57	0.86	0.64	0.36	0.29	0	1
Data_32	0.07	0.14	0.93	NA	0.29	0.5	0.86	1	NA	0.71	NA	0.36	0.64	0.79	0	0.21	0.43	0.57
Data_33	0.29	0.36	0.93	NA	0.43	0.21	0.86	0.71	NA	0.14	NA	0.5	0.64	0.79	0.07	0.57	0	1
Data_34	0.5	0.57	0.71	NA	0.36	0.29	0.93	0.79	NA	0.14	NA	0.86	0.64	0.43	0	0.07	0.21	1

*NA indicates missing values due to unavailability of the original metric or method incompatibility with specific datasets. The 'Mean score' row at the bottom represents the average normalized rank score for each method across all applicable datasets. A higher mean score indicates better overall performance under the given evaluation metric.

Supplementary Table 21

Comparison of methods based on mean Rank Scores of biological relevance metrics (Moran’s I, Geary’s C)

Data Name	STAMP	GASTON	STAGATE	stLearn	PROST	SEDR	GraphST	BASS	BayesSpace	louvain	SpaGCN (HE)	SCAN-IT	CCST	SpaGCN	leiden	SpaceFlow	conST	SAGE
Data_1	0.88	0.94	0.71	0.12	1	0.76	0	0.53	0.06	0.65	0.29	0.35	0.41	0.24	0.59	0.18	0.47	0.82
Data_2	0.88	0.94	0.56	0	1	0.76	0.41	0.06	0.71	0.56	0.35	0.29	0.12	0.18	0.24	0.47	0.65	0.82
Data_3	0.88	0.94	0.41	0.65	1	0.82	0.18	0.59	0.12	0.06	0.53	0	0.71	0.29	0.47	0.24	0.35	0.76
Data_4	0.76	0.82	0.18	0.24	1	0.94	0.41	0.12	0.06	0.35	0.65	0.29	0.71	0.47	0.53	0	0.59	0.88
Data_5	0.94	1	0.71	0.65	0.82	0.76	0.38	0.53	0	0.24	0.18	0.38	0.29	0.47	0.12	0.06	0.59	0.88
Data_6	0.94	1	0.29	0.71	0.82	0.76	0.59	0.18	0.62	0.24	0.41	0.35	0	0.47	0.56	0.12	0.06	0.88
Data_7	0.88	0.94	0.71	0	0.18	1	0.73	0.41	0.29	0.82	0.47	0.56	0.12	0.56	0.68	0.06	0.35	0.24
Data_8	0.06	0.12	0.76	0.53	0.82	0.94	0	0.18	0.68	0.88	0.38	0.47	0.24	0.29	1	0.38	0.68	0.59
Data_9	0.71	0.76	0.47	0.53	1	0.41	0.09	0.24	0.09	0.88	0.94	0.18	0.68	0.35	0.29	0	0.73	0.65
Data_10	0.82	0.88	0.53	0.29	1	0.47	0.12	0.73	0.65	0.21	0.06	0.73	0.41	0.35	0.21	0	0.59	0.94
Data_11	0.94	1	0.41	0.15	0.24	0.59	0.47	0.35	0.82	0.76	0.15	0.53	0.06	0.29	0.71	0	0.65	0.88
Data_12	0.76	0.82	0.47	0.41	0.94	1	0.18	0.12	0.24	0.71	0.29	0.53	0	0.35	0.65	0.06	0.59	0.88
Data_13	0.67	0.75	NA	NA	0.96	0.83	0	0.42	NA	0.17	NA	0.25	NA	0.08	0.5	0.33	0.96	0.58
Data_14	0.75	0.83	NA	NA	0.92	0.5	0.17	0.42	NA	1	NA	0.33	NA	0.25	0.08	0	0.58	0.67
Data_15	0.82	0.91	NA	NA	1	0.55	0.18	0.64	NA	0.27	NA	0.36	NA	0.45	0.09	0	NA	0.73
Data_16	0.33	0.42	NA	NA	0.5	0.58	0.75	0.08	NA	1	NA	0.17	NA	0.83	0.92	0	0.67	0.25
Data_17	0.83	0.92	NA	NA	1	0.46	0.46	0.29	NA	0.58	NA	0.17	NA	0.29	0.08	0	0.75	0.67
Data_18	0.7	0.8	NA	NA	0.9	0.6	NA	0.2	NA	0.4	NA	0.3	NA	0.5	0	0.1	NA	1
Data_19	0.7	0.8	NA	NA	0.9	0.5	NA	0	NA	0.4	NA	0.2	NA	0.3	0.6	0.1	NA	1
Data_20	0.67	0.78	NA	NA	0.89	NA	NA	0.5	NA	0	NA	0.5	NA	0.28	0.28	0.11	NA	1
Data_21	0.09	0.18	NA	NA	0.45	0.73	0.64	NA	NA	1	NA	0.36	NA	0.27	0.91	0	0.55	0.82
Data_22	0.64	0.72	0.43	NA	0.53	0.14	0.6	0.86	NA	0	NA	0.93	0.72	0.29	0.07	0.36	0.21	1
Data_23	0.39	0.46	0.82	NA	0.6	0.07	0.54	0.82	NA	0.36	NA	0.93	0.6	0.46	0.28	0.14	0	1
Data_24	0.73	0.8	0.84	NA	0.73	0	0.46	NA	NA	0.15	NA	0.38	0.31	0.54	0.23	0.73	0.08	1
Data_25	0.64	0.72	0.36	NA	0.53	0.14	0.6	0.86	NA	0	NA	0.72	0.29	0.21	0.07	0.93	0.43	1
Data_26	0.32	0.4	0.64	NA	0.35	0.57	0.93	1	NA	0	NA	0.71	0.36	0.14	0.07	0.79	0.35	0.86
Data_27	0.57	0.68	0.86	NA	0.57	0.29	0.93	1	NA	0.07	NA	0.6	0.14	0.21	0	0.43	0.36	0.79
Data_28	0.64	0.72	0.68	NA	0.54	0.17	0.86	1	NA	0.07	NA	0.46	0.36	0.25	0	0.93	0.6	0.21
Data_29	0.68	0.75	0.21	NA	0.57	0.36	0.93	1	NA	0.07	NA	0.5	0.43	0.14	0	0.72	0.29	0.86
Data_30	0.64	0.71	0.79	NA	0.53	0	0.32	0.11	NA	0.86	NA	0.93	0.4	0.21	0.4	0.5	0.11	1
Data_31	0.25	0.32	0.71	NA	0.5	0.07	0.93	0.79	NA	0.32	NA	0.57	0.86	0.64	0.32	0.21	0	1
Data_32	0.04	0.11	0.93	NA	0.21	0.5	0.86	1	NA	0.68	NA	0.36	0.68	0.79	0.1	0.25	0.43	0.57
Data_33	0.25	0.32	0.93	NA	0.43	0.28	0.86	0.71	NA	0.14	NA	0.5	0.64	0.79	0.07	0.57	0	1
Data_34	0.5	0.57	0.71	NA	0.25	0.29	0.93	0.79	NA	0.17	NA	0.86	0.64	0.43	0	0.07	0.28	1

* NA indicates missing values due to unavailability of the original metric or method incompatibility with specific datasets. Evaluation of biological relevance using the mean normalized rank scores of Moran’s I and Geary’s C, which assess the spatial autocorrelation of marker gene expression. Scores closer to 1 indicate stronger biological signal alignment in clustering results.

Supplementary Table 22

Comparison of ranking scores of SAGE with 16 state-of-the-art spatial transcriptomics domain analysis methods based on eight metrics (NMI, HOM, COM, CHAOS, PAS, ASW, MRI, GC).

Methods	NMI	HOM	COM	Mean	CHAOS	PAS	ASW	Mean	MRI	GC	Mean	Total
SAGE	**0.97**	**0.91**	**0.95**	**0.94**	0.78	0.70	0.59	0.69	**0.82**	**0.81**	**0.82**	**2.45**
BASS	0.74	0.69	0.78	0.74	0.66	0.71	0.69	0.69	0.51	0.53	0.52	1.94
PROST	0.72	0.73	0.69	0.71	0.78	0.85	0.68	0.77	0.70	0.70	0.70	2.18
BayesSpace	0.71	0.65	0.69	0.68	0.62	0.63	0.53	0.59	0.35	0.37	0.36	1.64
SCAN-IT	0.69	0.70	0.64	0.68	0.85	**0.88**	**0.88**	**0.87**	0.46	0.47	0.46	2.01
GraphST	0.66	0.60	0.71	0.66	0.56	0.50	0.48	0.51	0.50	0.49	0.50	1.67
GASTON	0.61	0.57	0.59	0.59	**0.88**	0.80	0.76	0.81	0.61	0.62	0.62	2.02
SpaGCN	0.53	0.53	0.52	0.53	0.29	0.31	0.46	0.35	0.38	0.37	0.38	1.26
SpaceFlow	0.47	0.69	0.39	0.52	0.45	0.41	0.32	0.39	0.26	0.26	0.26	1.17
CCST	0.50	0.58	0.44	0.51	0.84	0.86	0.81	0.84	0.41	0.40	0.41	1.75
stLearn	0.49	0.43	0.54	0.49	0.33	0.37	0.34	0.35	0.35	0.34	0.35	1.18
SEDR	0.41	0.29	0.57	0.42	0.48	0.58	0.45	0.50	0.51	0.51	0.51	1.44
STAGATE	0.38	0.39	0.38	0.38	0.46	0.40	0.41	0.42	0.60	0.61	0.61	1.41
STAMP	0.35	0.38	0.34	0.36	0.25	0.26	0.37	0.29	0.70	0.70	0.70	1.35
SpaGCN(HE)	0.28	0.26	0.31	0.28	0.17	0.20	0.30	0.22	0.38	0.39	0.39	0.89
conST	0.27	0.28	0.26	0.27	0.21	0.21	0.33	0.25	0.43	0.44	0.44	0.96
leiden	0.10	0.11	0.10	0.10	0.08	0.08	0.16	0.11	0.35	0.34	0.35	0.55
louvain	0.08	0.07	0.08	0.08	0.12	0.13	0.27	0.17	0.40	0.40	0.40	0.65

Supplementary Table 23

Comparison of Standard Deviations (SD) Across Different Methods

Metric_SD	STAMP	GASTON	BASS	BayesSpace	CCST	GraphST	PROST	SCAN-IT	SEDR	STAGATE	SpaGCN	SpaGCN(HE)	SpaceFlow	conST	leiden	louvain	stLearn	SAGE
NMI_SD	0.168	0.272	0.175	0.174	0.144	0.286	0.173	0.171	0.199	0.084	0.078	0.236	0.147	0.257	0.090	0.294	0.263	0.042
HOM_SD	0.182	0.254	0.159	0.172	0.151	0.259	0.192	0.171	0.193	0.082	0.070	0.254	0.207	0.265	0.099	0.325	0.245	0.096
COM_SD	0.144	0.260	0.172	0.157	0.139	0.362	0.157	0.176	0.182	0.083	0.071	0.196	0.122	0.255	0.094	0.304	0.256	0.072
CHAOS_SD	0.134	0.074	0.135	0.056	0.162	0.270	0.125	0.099	0.131	0.097	0.031	0.147	0.189	0.062	0.068	0.252	0.210	0.178
PAS_SD	0.106	0.126	0.148	0.071	0.147	0.254	0.080	0.119	0.039	0.100	0.040	0.102	0.180	0.116	0.082	0.269	0.224	0.223
ASW_SD	0.189	0.255	0.236	0.147	0.186	0.371	0.201	0.273	0.230	0.225	0.110	0.103	0.241	0.238	0.162	0.315	0.211	0.260
MRI_SD	0.268	0.271	0.219	0.252	0.277	0.301	0.320	0.332	0.306	0.345	0.230	0.234	0.260	0.197	0.295	0.291	0.255	0.208
GC_SD	0.258	0.264	0.214	0.256	0.276	0.302	0.320	0.330	0.312	0.348	0.224	0.239	0.251	0.195	0.298	0.289	0.262	0.222

*This table presents the standard deviations (SD) of various spatial transcriptomics analysis methods (STAMP, GASTON, BASS, BayesSpace, CCST, SAGE, GraphST, PROST, SCAN-IT, SEDR, STAGATE, SpaGCN, SpaGCN (HE), SpaceFlow, conST, leiden, louvain, stLearn) across multiple evaluation metrics. Standard deviation measures the stability of each method across different experiments or datasets, with lower values indicating more stable results.

Supplementary Table 24

Median values of eight evaluation metrics for 16 methods on the DLPFC dataset.

median_metric	STAGATE	stLearn	PROST	SEDR	GraphST	BASS	BayesSpace	louvain	SpaGCN (HE)	SCAN-IT	CCST	SpaGCN	leiden	SpaceFlow	conST	SAGE
Median NMI	0.505	0.545	0.615	0.535	0.620	0.615	0.600	0.240	0.485	0.600	0.510	0.525	0.245	0.485	0.520	**0.710**
Median HOM	0.515	0.535	0.630	0.450	0.645	0.635	0.615	0.250	0.495	0.635	0.570	0.540	0.260	0.675	0.555	**0.720**
Median COM	0.490	0.550	0.595	0.690	0.605	0.605	0.585	0.235	0.475	0.575	0.475	0.515	0.235	0.385	0.490	**0.710**
Median CHAOS	0.061	0.063	0.060	0.061	0.061	0.060	0.061	0.067	0.065	0.060	0.060	0.063	0.067	0.061	0.062	**0.060**
Median PAS	0.085	0.127	**0.008**	0.014	0.092	0.029	0.032	0.460	0.249	0.011	0.011	0.132	0.499	0.144	0.188	0.016
Median ASW	0.521	0.512	0.556	0.544	0.522	0.539	0.532	0.483	0.505	0.552	**0.558**	0.535	0.484	0.468	0.505	0.529
Median MRI	0.336	0.294	**0.408**	0.376	0.310	0.337	0.315	0.336	0.315	0.307	0.305	0.307	0.346	0.300	0.332	0.370
Median GC	0.663	0.704	**0.593**	0.625	0.691	0.664	0.687	0.665	0.685	0.695	0.693	0.695	0.657	0.700	0.669	0.631

Supplementary Table 25

Marker Genes in Human Dorsolateral Prefrontal Cortex (DLPFC) Across Cortical Layers and White Matter

Layer	Marker Genes	Description
WM	MOBP, MBP	MOBP: Myelin-associated oligodendrocytes basic protein; marker of mature oligodendrocytes, enriched in white matter.<br>MBP: Myelin basic protein; structural component of the myelin sheath.
L6	TLE4, KRT17	TLE4: Transcriptional repressor enriched in deep-layer (L6) excitatory neurons.<br>KRT17: Intermediate filament protein recently identified in L6 via spatial transcriptomics.
L5	BCL11B, TRABD2A, PCP4	BCL11B (CTIP2): Transcription factor marking L5 corticospinal-projecting neurons.<br>TRABD2A: Preferentially expressed in L5 excitatory neurons, linked to cortical development.<br>PCP4: Purkinje cell protein 4; calcium-binding protein enriched in deep-layer pyramidal neurons including L5.
L4	RORB, PVALB	RORB: Nuclear receptor and classic marker for L4 excitatory neurons; involved in sensory information processing.<br>PVALB: Parvalbumin; calcium-binding protein marking fast-spiking interneurons highly enriched in L4.
L3	FREM3	FREM3: ECM-related protein enriched in L3; possibly involved in extracellular matrix interactions.
L2	HPCAL1	HPCAL1: Neuronal calcium sensor protein enriched in upper-layer excitatory neurons (especially L2).
L1	AQP4, RELN, FABP7	AQP4: Astrocytic water channel protein enriched in L1. RELN: ECM glycoprotein secreted by Cajal-Retzius cells in L1.<br>FABP7: Fatty acid-binding protein 7; marker of radial glia-like astrocytes, enriched in superficial layers including L1.

*This table lists widely recognized marker genes for each cortical layer (L1–L6) and the white matter (WM) in the human dorsolateral prefrontal cortex (DLPFC). These genes have been validated in spatial transcriptomic studies to be specifically enriched in their respective layers and are commonly used for evaluating the performance of spatial clustering methods in capturing laminar organization.

Supplementary Table 26

Ablation Study Results Using Different Gene Sets (NMI and MRI Metrics, n = 12 Slices)

Slides	NMI			MRI		
	Random	HVGs	HSGs	Random	HVGs	HSGs
151507	0.618	0.670	**0.714**	0.013	0.022	**0.025**
151508	0.607	0.527	**0.678**	0.014	0.019	**0.022**
151509	0.655	0.604	**0.701**	0.016	0.025	**0.029**
151510	0.499	0.622	**0.649**	0.015	0.021	**0.024**
151669	0.568	0.609	**0.638**	0.013	0.019	**0.023**
151670	0.443	0.542	**0.546**	0.014	0.018	**0.022**
151671	0.573	0.703	**0.780**	0.015	0.024	**0.030**
151672	0.569	0.674	**0.761**	0.015	0.023	**0.027**
151673	0.650	0.666	**0.749**	0.023	0.045	**0.053**
151674	0.531	0.631	**0.758**	0.021	0.046	**0.054**
151675	0.616	0.626	**0.711**	0.019	0.036	**0.043**
151676	0.579	0.622	**0.728**	0.018	0.034	**0.039**

*This table presents the results of an ablation study based on SAGE, using different gene sets (Random, HVGs, HSGs) across 12 human dorsolateral prefrontal cortex (DLPFC) tissue slices. The evaluation metrics include NMI and MRI. Results show that HSGs yielded the best performance in most slices, indicating their superior representativeness for spatial structure reconstruction.

Supplementary Table 27

Cross-slice Consistency Analysis of SAGE-identified Gene Sets (NMI Metric, n = 12 DLPFC Slices)

Slides	NMI		
	Random_equal	HVGs_equal	Common_TSG
151507	0.470	0.547	**0.645**
151508	0.446	0.429	**0.580**
151509	0.517	0.507	**0.614**
151510	0.348	0.525	**0.569**
151669	0.414	0.507	**0.580**
151670	0.311	0.431	**0.488**
151671	0.419	0.596	**0.727**
151672	0.432	0.572	**0.668**
151673	0.517	0.552	**0.669**
151674	0.363	0.535	**0.673**
151675	0.447	0.524	**0.660**
151676	0.417	0.517	**0.630**

*This table presents the results of a multi-slice consistency analysis that assesses the robustness and reproducibility of SAGE-selected genes across adjacent DLPFC tissue sections. 12 slices are grouped into 3 multi-slice regions: (151507–151510), (151669–151672), and (151673–151676), corresponding to common TSG sets of 177, 229, and 377 genes, respectively. To ensure fair comparison, we construct three gene sets for each group: Random_equal, HVGs_equal, and Common_TSG, where the first two contain the same number of genes as the corresponding Common_TSG set. The Normalized Mutual Information (NMI) values show that Common_TSG consistently achieves higher clustering performance across slices, indicating that SAGE identifies spatially informative genes that remain stable and biologically consistent across neighboring tissue sections.

Supplementary Table 28

Clustering Quality Assessment of Multiple Methods on Zebrafish Melanoma Slices (Metrics: SC and DB)

	SC								DB							
Samples	SCAM-IT	STAGATE	stLearn	SEDR	STAIG	SpaGCN	GraphST	SAGE	SCAM-IT	STAGATE	stLearn	SEDR	STAIG	SpaGCN	GraphST	SAGE
Sample A	0.181	0.138	0.124	0.126	0.111	0.072	0.078	**0.267**	1.378	1.735	1.885	1.571	**1.425**	2.222	1.845	1.564
Sample B	0.201	0.173	0.167	0.183	0.117	0.133	0.147	**0.219**	1.200	1.444	1.697	1.758	2.100	1.887	2.197	**1.139**
Sample C	0.285	0.230	0.241	0.166	0.179	0.184	0.117	**0.310**	1.372	1.694	1.937	1.743	1.668	2.200	2.279	**1.282**

*This table presents the clustering performance of eight spatial domain segmentation methods—SCAM-IT, STAGATE, stLearn, SEDR, STAIG, SpaGCN, GraphST, and SAGE—on three zebrafish melanoma tissue sections (Sample A, Sample B, Sample C), evaluated using the Silhouette Coefficient (SC) and the Davies–Bouldin Index (DB). The Silhouette Coefficient measures the compactness and separation of clusters, with higher values indicating better-defined spatial domains. The Davies–Bouldin Index assesses the average similarity between each cluster and its most similar counterpart, where lower values indicate superior clustering performance. Overall, SAGE achieves the highest SC scores and relatively low DB values across most samples, demonstrating its superior ability in capturing biologically meaningful and spatially coherent domains.

Supplementary Note

Supplementary Data 1–13 provide quantitative results supporting the main conclusions of this study. These include spatial domain-specific transcriptional signatures, differentially expressed genes, pathway and GO enrichment analyses, and gene-level feature metrics across multiple datasets. Specifically, Supplementary Data 1 presents the gene-by-topic z-score matrix for the DLPFC, highlighting laminar- and cell-type-specific patterns. Supplementary Data 2–4 report domain-specific DE genes, KEGG/GO enrichment, and GSEA for domain 5 in slice 151673. Supplementary Data 5 lists topics-specific genes (TSGs) across all 12 DLPFC slices, enabling cross-slice spatial consistency analysis. Supplementary Data 6 shows the mouse brain z-score matrix, illustrating cell-type-specific expression (e.g., granule, Purkinje, and molecular layer interneurons). Supplementary Data 7–10 summarize breast cancer topic/cluster signatures, top-ranked genes, and pathway enrichment, revealing subtype-specific programs and microenvironment activation. Supplementary Data 11–13 contain DE genes and GO enrichment for zebrafish melanoma at the tumor–muscle interface, highlighting muscle and tumor-associated transcriptional programs. Supplementary Data 14 contain SAGE and six state-of-the-art spatial domain analysis methods on the rice embryo spatiotemporal dataset.

Supplementary Data 1. Standardized gene-by-topic z-score matrix derived from SAGE on the DLPFC dataset. This file presents the standardized gene expression profiles across 17 topics inferred by the SAGE model applied to the DLPFC spatial transcriptomic dataset. Each row corresponds to a gene, and each column represents a topic (indexed by topic number). The matrix values are z-scores, indicating the relative enrichment or depletion of each gene within each topic. Higher positive z-scores suggest stronger topic-specific expression. Notably, genes such as *MOBP* (z-score = 12.22), *PCP4* (19.88), *PVALB* (11.28), *NEFM* (11.31), and *HPCAL1* (12.34) show distinct laminar or cell-type-specific expression patterns. These patterns are consistent with known biological annotations, validating the interpretability and biological relevance of the topic model.

Supplementary Data 2. Differentially expressed genes in domain 5 of slice 151673 (DLPFC). This table lists the genes that are significantly upregulated or downregulated in domain 5, as identified by SAGE analysis of the 151673 slice. Each row corresponds to a gene, and the Score column represents the differential expression metric derived from SAGE, reflecting the relative contribution or enrichment of each gene within this spatial domain. Positive scores indicate upregulated genes, while negative scores indicate downregulated genes. Notable genes with high scores include ENC1 (Score = 18.83), MT-CO1 (Score = 17.76), MT-ATP6 (Score = 16.08), HOPX (Score = 15.59), MT-CYB (Score = 15.57), and YWHAH (Score = 14.58), suggesting key roles in cellular metabolism, neuronal activity, and domain-specific functions. This dataset provides a quantitative basis for interpreting the biological characteristics of domain 5 within the DLPFC.

Supplementary Data 3. Pathway enrichment analysis of genes upregulated in domain 5 of slice 151673 (DLPFC). This file presents the results of functional enrichment analysis performed on genes significantly upregulated in domain 5, using KEGG_2021_Human and GO_Biological_Process_2021 gene sets. Each row corresponds to a pathway or biological process, with columns indicating Gene_set, Term, Overlap, p-value, Adjusted P-value, Odds Ratio, Combined Score, and the list of contributing genes. The analysis highlights pathways and processes that are specifically enriched in this domain, reflecting the molecular and functional characteristics of domain 5, including protein synthesis, energy metabolism, synaptic signaling, and other neuron-specific biological processes.

Supplementary Data 4. Gene set enrichment analysis (GSEA) results for pre-ranked genes in domain 5 of slice 151673 in the DLPFC dataset. This file presents the results of pre-ranked GSEA performed using GSEApy. Each row corresponds to a gene set (e.g., KEGG, GO, Hallmark), and the table includes the enrichment statistics: Enrichment Score (ES), Normalized Enrichment Score (NES), p-value, FDR q-value, Rank at Max, and the list of genes contributing to the enrichment. Positive ES values indicate gene sets enriched in upregulated genes, while negative ES values indicate enrichment in downregulated genes. This dataset allows identification of biological pathways and processes that are specifically enriched in domain 5, facilitating the interpretation of its molecular and functional characteristics.

Supplementary Data 5. Detailed transcriptional and feature information of topics-specific genes (TSGs) identified by SAGE across 12 DLPFC tissue slices (151507–151676). This file presents the TSGs detected by SAGE in the DLPFC dataset, along with associated gene-level statistics. Each row corresponds to a gene, and columns include gene_ids, feature_types, genome, n_cells, n_counts, highly_variable, highly_variable_rank, means, variances, variances_norm, genes_mri, and tsgs_rank. The dataset provides information on gene expression levels, variability metrics, and importance scores for all identified TSGs, enabling assessment of their contribution to spatial domain reconstruction and cross-slice spatial consistency.

Supplementary Data 6. Standardized gene-by-topic z-score matrix derived from SAGE on the mouse brain dataset. This file presents the standardized gene expression profiles across 27 topics inferred by the SAGE model applied to the mouse brain spatial transcriptomic dataset. Each row corresponds to a gene, and each column represents a topic (indexed by topic number). The matrix values are z-scores, indicating the relative enrichment or depletion of each gene within each topic. Higher positive z-scores suggest stronger topic-specific expression. Notable examples include *Gabra6* ($z = 8.66$ in a CBX-related topic) marks granule cells in the cerebellar granular layer (GCL). *Grid2* ($z = 7.57$) is associated with Purkinje cells in the Purkinje cell layer (PCL). *Pvalb* ($z = 16.69$) highlights molecular layer (ML) interneurons.

Supplementary Data 7. Standardized gene-by-topic z-score matrix derived from SAGE on the breast cancer tissue dataset. This file presents the standardized gene expression profiles across 27 topics inferred by the SAGE model applied to the breast cancer tissue spatial transcriptomic dataset. Each row corresponds to a gene, and each column represents a topic (indexed by topic number). The matrix values are z-scores, indicating the relative enrichment or depletion of each gene within each topic. Higher positive z-scores suggest stronger topic-specific expression. Notably, several biologically meaningful topics emerge from this decomposition: (1) Topic 2, 5, 16, and 19 show high spatial concordance with IDC (Invasive Ductal Carcinoma) subtypes 4, 2, 6, and 5, respectively, indicating subtype-specific transcriptional programs. (2) Topic 13 reveals a distinct gene co-expression pattern enriched in DCIS/LCIS subtypes 1, 2, and 5, but not subtype 4. This topic is characterized by the co-upregulation of luminal epithelial marker *TFF1*, basal-like gene *KRT6B*, and microenvironment regulator *SERPINA3* (see Supplementary Fig. 31d). These findings suggest a mixed luminal–basal phenotype with signatures of a tumor-promoting microenvironment, potentially indicating pre-invasive states with invasive potential.

Supplementary Data 8. Top-ranked genes of Cluster 14 identified by SAGE in breast cancer tissue. This table lists the top-ranked genes in Cluster 14, a spatial domain identified by SAGE analysis of breast cancer tissue. The genes are ranked by their SAGE-derived scores, reflecting their contribution to the transcriptional program of this cluster. Notably, Cluster 14 is enriched for extracellular matrix (ECM) remodeling and mesenchymal transition genes (e.g., *COL1A2*, *BGN*, *VIM*), indicative of an activated precancerous microenvironment.

Supplementary Data 9. Top-ranked genes of Cluster 7 identified by SAGE in breast cancer tissue. This table presents the top-ranked genes in Cluster 7, a transcriptionally defined spatial domain identified by SAGE in breast cancer tissue. Genes such as *PBX1*, *CDH1*, and *KRT19* highlight an epithelial and luminal lineage-associated signature, suggesting Cluster 7 corresponds to a well-differentiated tumor or normal epithelial region.

Supplementary Data 10. Pathway enrichment analysis of domain 14 upregulated genes identified by SAGE in breast cancer tissue. This table summarizes the results of pathway enrichment analysis performed on genes significantly upregulated in Cluster 14 using KEGG_2021_Human and MSigDB_Hallmark_2020 databases. Notably, enriched pathways include extracellular matrix (ECM) remodeling, focal adhesion, phagosome, and regulation of actin cytoskeleton, reflecting mesenchymal and stromal activation. These findings support the presence of an activated precancerous microenvironment within Cluster 14.

Supplementary Data 11. Differentially expressed genes in domain 12 of the tumor–muscle interface (Zebrafish melanoma sample B). This table lists genes significantly upregulated in Domain 12, located near normal muscle tissue at the tumor–muscle interface in zebrafish melanoma sample B. SAGE analysis revealed strong expression of muscle-associated genes such as pvalb1, myhc4, ckma, and tpma, consistent with skeletal muscle contraction and stress response. Genes are ranked by significance, with associated scores, log fold-changes, and adjusted p-values provided.

Supplementary Data 12. GO biological process enrichment of genes upregulated in Domain 12 (Zebrafish melanoma sample B). This table summarizes Gene Ontology (GO) biological processes significantly enriched among genes upregulated in Domain 12 at the tumor–muscle interface. Enrichment analysis highlights processes related to striated and skeletal muscle contraction, filament assembly, and muscle tissue morphogenesis, reflecting the muscle-like transcriptional profile of this domain. Key enriched terms include “striated muscle contraction,” “skeletal muscle thin filament assembly,” and “muscle contraction,” consistent with high expression of muscle structural genes such as tpma, tnnt3b, mybpha, and myom2a. For each term, overlap size, p-values, combined scores, and contributing genes are listed.

Supplementary Data 13. GO biological process enrichment of genes upregulated in Domain 10 (zebrafish melanoma sample B). This table summarizes GO biological processes significantly enriched among genes upregulated in Domain 10 at the tumor-facing side of the tumor–muscle interface. Enriched terms highlight tumor microenvironment–associated activities, including mRNA splicing and RNA processing, post-transcriptional regulation, neural crest/stem cell–like programs, and signaling and cytoskeletal features such as melanosome organization. For each GO term, the overlap, statistical significance, and contributing genes are listed.

Supplementary Data 14. Benchmarking SAGE and six state-of-the-art spatial domain analysis methods on the rice embryo spatiotemporal dataset. This table reports NMI, HOM, COM, ARI, PAS, ASW, and CHAOS scores for GraphST, SAGE, SCAN-IT, SEDR, SpaGCN, STAGATE, and stLearn on four spatial sections (6HAI_1, 24HAI_1, 36HAI_1, and 48HAI_1) from the Spatiotemporal Transcriptomic Atlas of Rice Embryonic Cells. These metrics correspond to Figure R9 and Supplementary Fig. 53 and extend Table R3 by providing the full set of accuracy- and continuity-related benchmark scores, showing that SAGE consistently attains the highest or near-highest accuracy (NMI, HOM, COM, ARI) while maintaining competitive spatial continuity (ASW, CHAOS, PAS).

Supplementary Figure 54. Across the four representative datasets (DLPFC slice 151673, mouse brain anterior section, human breast cancer, and zebrafish melanoma sample B), the dual-view embeddings in Supplementary Fig. 54 show a consistent pattern. The spatial-view embedding (SAG_emb) preserves large-scale anatomical geometry and laminar or lobular continuity, but functionally defined subpopulations that are spatially dispersed or intermingled tend to appear fragmented. In contrast, the feature-view embedding (FAG_emb) groups spots with similar transcriptional programs into compact clusters, even when they are spatially distant, at the cost of blurring some fine spatial transitions and boundaries. The fused embedding (Fuse_emb) combines the strengths of both views: spatially contiguous structures remain coherent and their boundaries are preserved, while transcriptionally coherent cell states become more compact and better separated in the latent space, leading to improved agreement with manual or expert annotations. In the DLPFC slice, the fused embedding maintains laminar organization while sharpening separation between cortical layers; in the mouse brain anterior section, it aligns well with known cortical and subcortical nuclei; in the human breast cancer section, it preserves tumor architecture while further resolving stromal and immune niches at the tumor edge; and in zebrafish melanoma, it retains tumor–normal separation while revealing transcriptionally distinct tumor and microenvironmental subdomains. The learned attention weights α_{coord} and α_{feat} ($\alpha_{\text{coord}} + \alpha_{\text{feat}} = 1$) further indicate that SAGE adaptively balances the spatial and feature views across tissue regions, rather than simply averaging two graphs, thereby providing an intuitive visualization of how spatial and functional information are integrated through dual-view fusion.
